# Supplementary material for: Conformational restriction shapes the inhibition of a multidrug efflux adaptor protein
Source: Nat Commun. 2023 Jul 18;14:3900. doi: 10.1038/s41467-023-39615-x (PMC10354078; doi:10.1038/s41467-023-39615-x)
Supplement: Supplementary file 1 — Supplementary Information [file 41467_2023_39615_MOESM1_ESM.pdf]

Supplementary Information

Conformational restriction shapes the inhibition of a multidrug efflux adaptor protein

Benjamin Russell Lewis[1], Muhammad R. Uddin[2], Mohammad Moniruzzaman[2], Katie M. Kuo[3], Anna J. Higgins[4], Laila M. N. Shah[1], Frank Sobott[4], Jerry M. Parks[5], Dietmar Hammerschmid[1], James C. Gumbart[3,6,*], Helen I. Zgurskaya[2,*], Eamonn Reading[1,*]

1. Department of Chemistry, Britannia House, 7 Trinity Street, King's College London, London, SE1 1DB, UK
2. Department of Chemistry and Biochemistry, University of Oklahoma, 101 Stephenson Parkway, Norman, Oklahoma 73019, USA
3. School of Chemistry and Biochemistry, Georgia Institute of Technology, 837 State Street NW, Atlanta, Georgia 30332, USA
4. School of Molecular and Cellular Biology & Astbury Centre for Structural Molecular Biology, University of Leeds, Leeds, UK
5. Bioscience Division, Oak Ridge National Laboratory, 1 Bethel Valley Rd, Oak Ridge, TN, 37831, USA
6. School of Physics, Georgia Institute of Technology, 837 State Street NW, Atlanta, Georgia 30332, USA

[*]Correspondence to: eamonn.reading@kcl.ac.uk, elenaz@ou.edu, gumbart@physics.gatech.edu

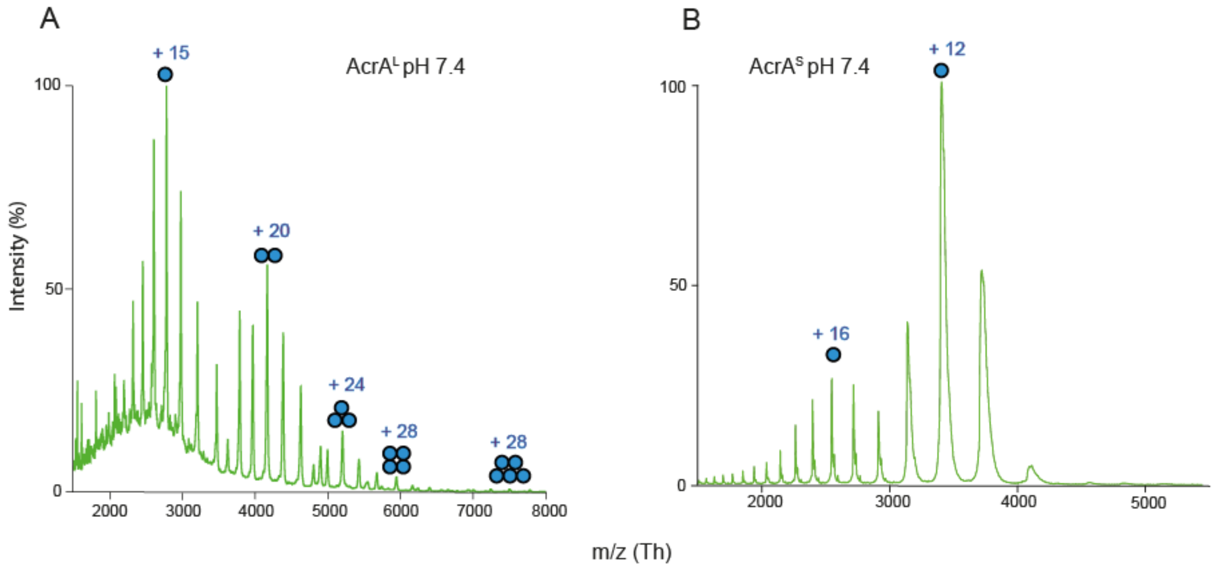

Figure S1. Native-MS of AcrA constructs at pH 7.4. A. Native-MS characterisation of AcrA^L construct at pH 7.4. Protein buffer exchanged to 100 mM ammonium acetate prior to MS, in the presence of 2 x critical micelle concentration (CMC) of DDM at 0.03 %. AcrA^L presents as a mix of oligomers up until pentamers. **B.** Native-MS characterisation of AcrA^S construct at pH 7.4. Protein buffer exchanged to 100 mM ammonium acetate prior to MS. AcrA^S presents as a monomer. See Supplementary Table 1 for masses.

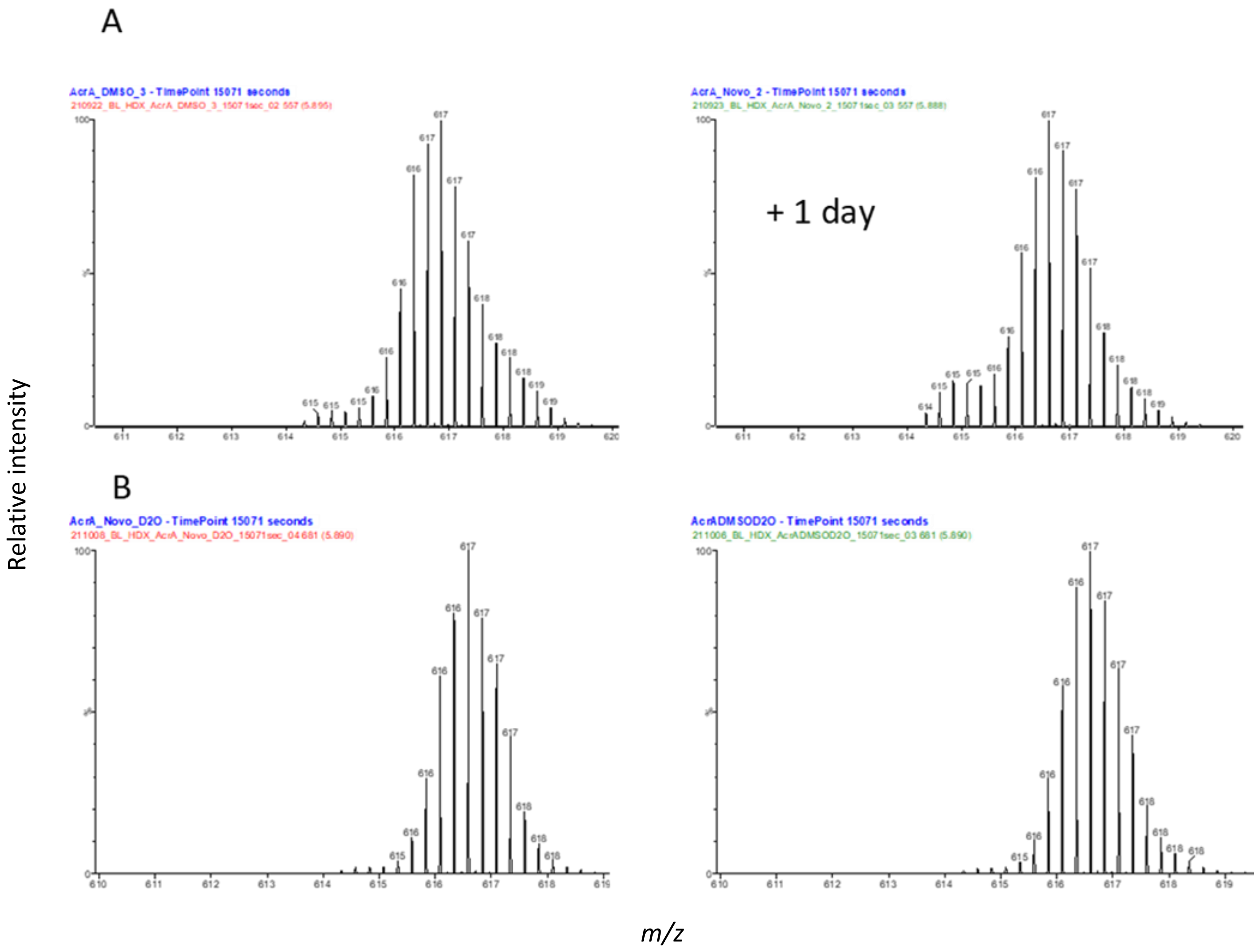

Figure S2. Optimization of HDX-MS conditions. A. One peptide selected throughout, after labelling in deuterated buffer for 4 hours. This represents two datasets for one protein taken one day after each other. There is an area in the low m/z (614-616 *m/z*) which is present, which represents protein aggregation/carryover. **B.** The same peptide and two subsequent datasets taken one day after each other after optimization steps. This was the addition of a SEC 'clean-up' stage of the protein sample before experiments and increasing pepsin washes from 2 to 3.

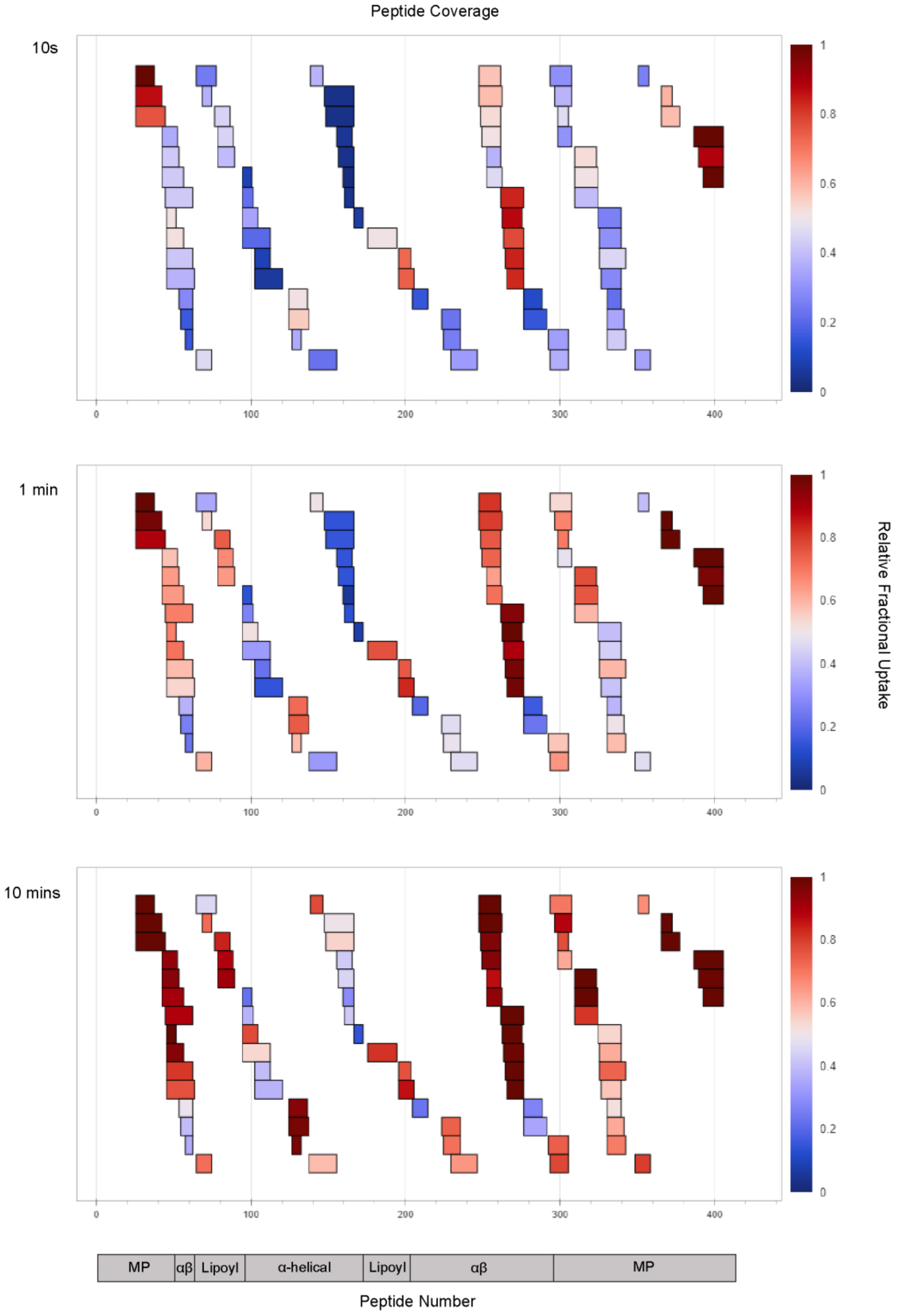

Figure S3. Relative fractional uptake of AcrA^S at pH 6.0. Relative fractional deuterium uptake (RFU) analysis of AcrA^S at pH 6.0 for three time points (pH 7.4 corrected). RFU was normalized using a MaxD control for AcrA^s (see methods and Source Data file). Areas which take up near-maximal deuteration at the earliest timepoints (10s) are indicative of protein regions which have no measurable secondary structure and are likely intrinsically disordered. Plotted using Pyhdx.[1]

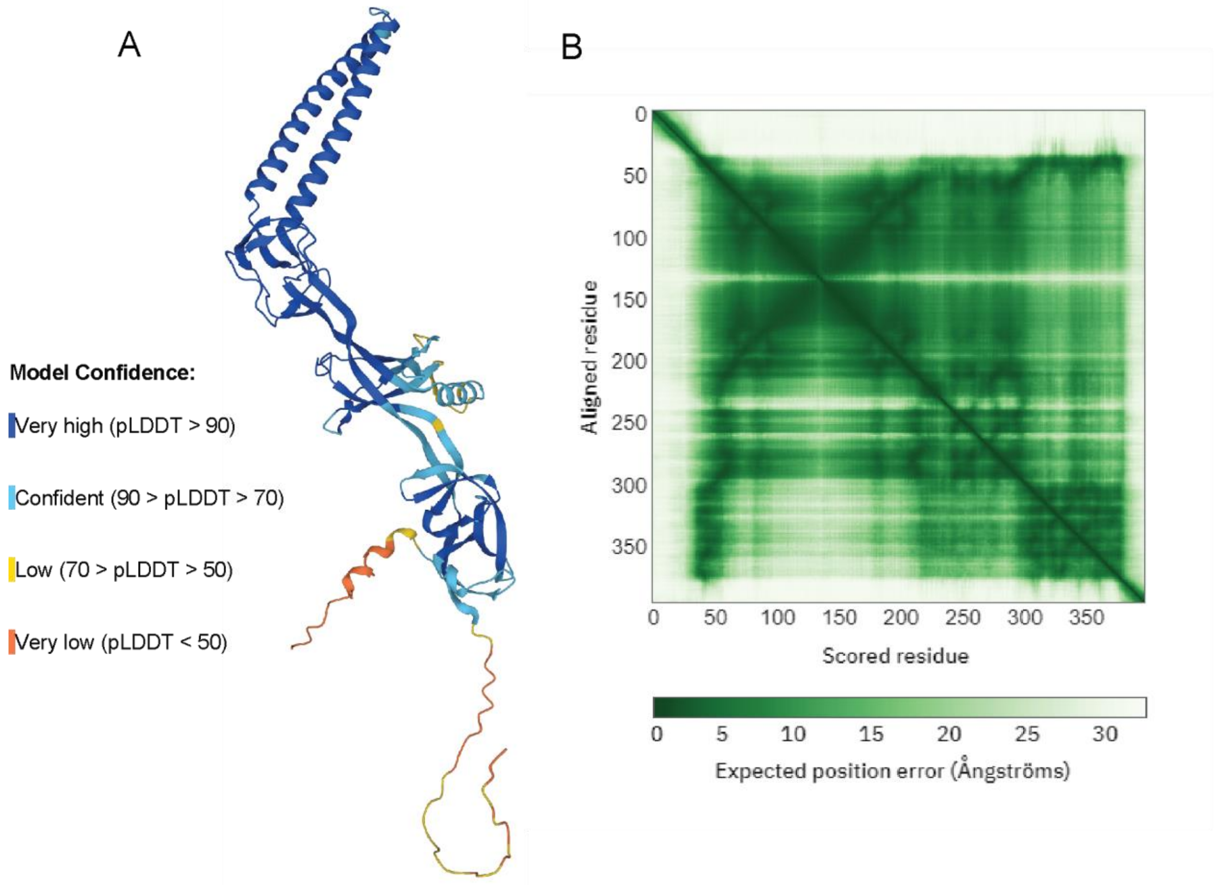

Figure S4. AlphaFold2 prediction of AcrA structure. A. Predicted structure of AcrA. Coloured coded based on the per-residue confidence score (pLDDT). A very low confidence score (<50) can indicate unstructured regions. **B.** Predicted aligned error plot. The colour position at (x, y) indicates Alphafold's expected position error at residue X, when the predicted and true structures are aligned on residue y. The colour bar represents how confident AlphaFold2's prediction is – dark green is high confidence, light green is low confidence.[2,3]

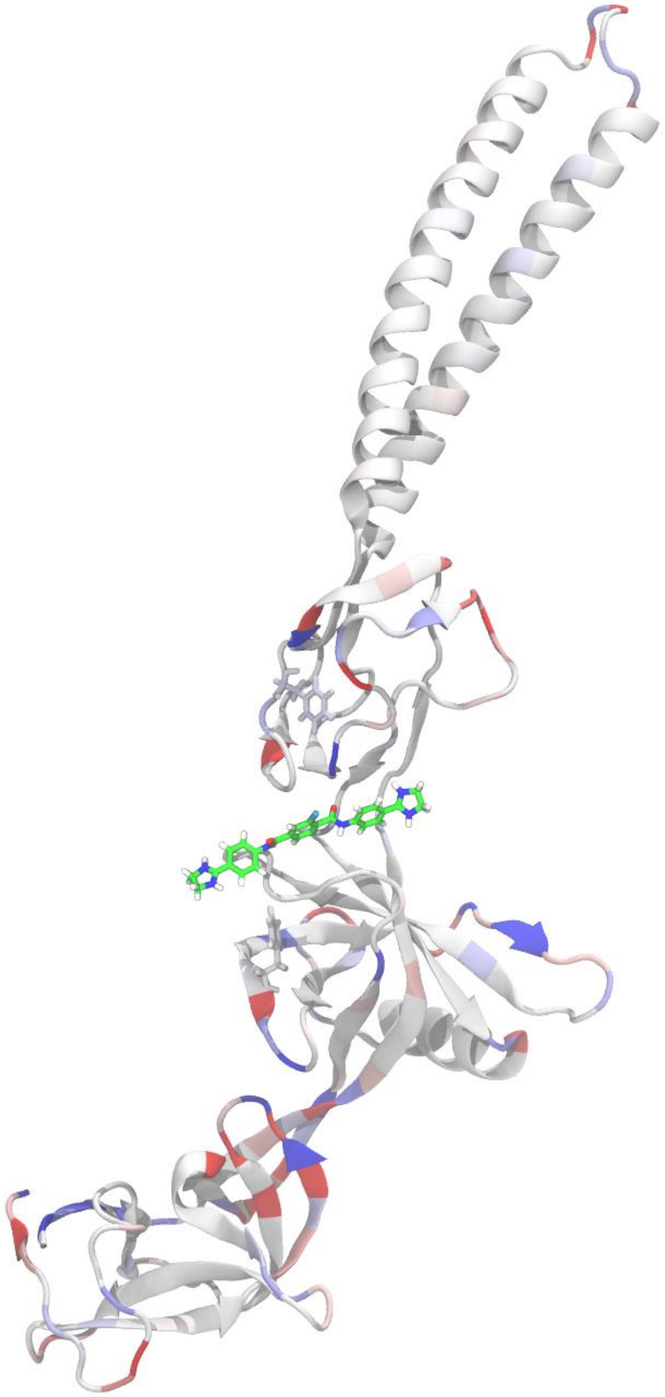

Figure S5. Solvent accessible surface area (SASA) from MD simulations of an AcrA^{S}. AcrA coloured according to the difference in SASA between the last 70 ns of 100-ns simulations of the bound and apo states, averaged over four replicas for each. Red indicates that the SASA is greater in the bound state while blue indicates it is greater in the apo state (the colour range is from -1 Å^2 to 1 Å^2). F81 and F254 are shown as sticks above and below the ligand.

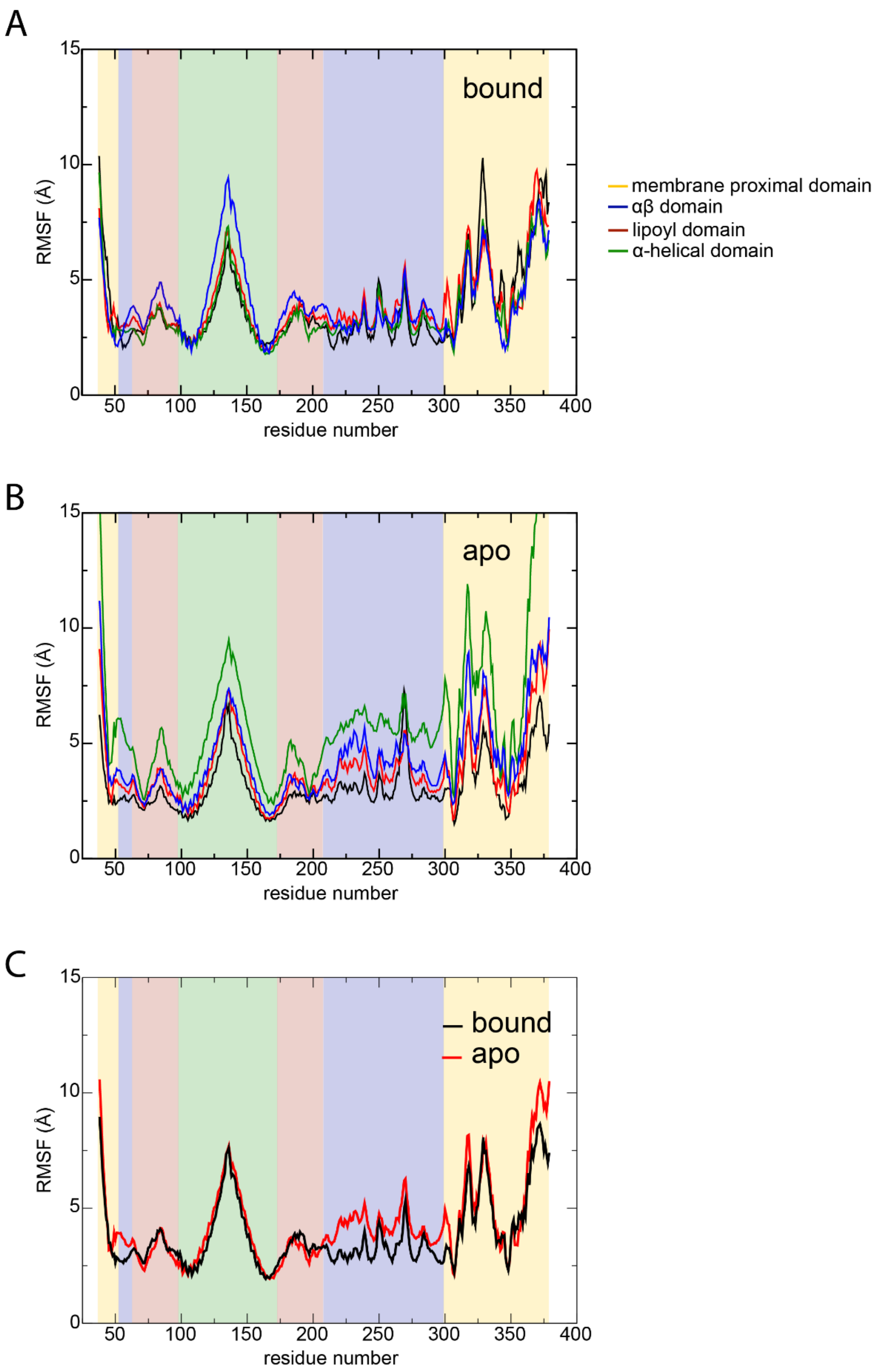

Figure S6. Root-mean-square fluctuations (RMSF) from MD simulations of an AcrA^{S}. RMSF of AcrA from four independent simulations for the (A) bound and (B) apo states. RMSF was calculated over the last 70 ns of each 100-ns simulation. (C) Average from the four replicas for the bound (black) and apo (red) states. The shading indicates the domains of AcrA as indicated.

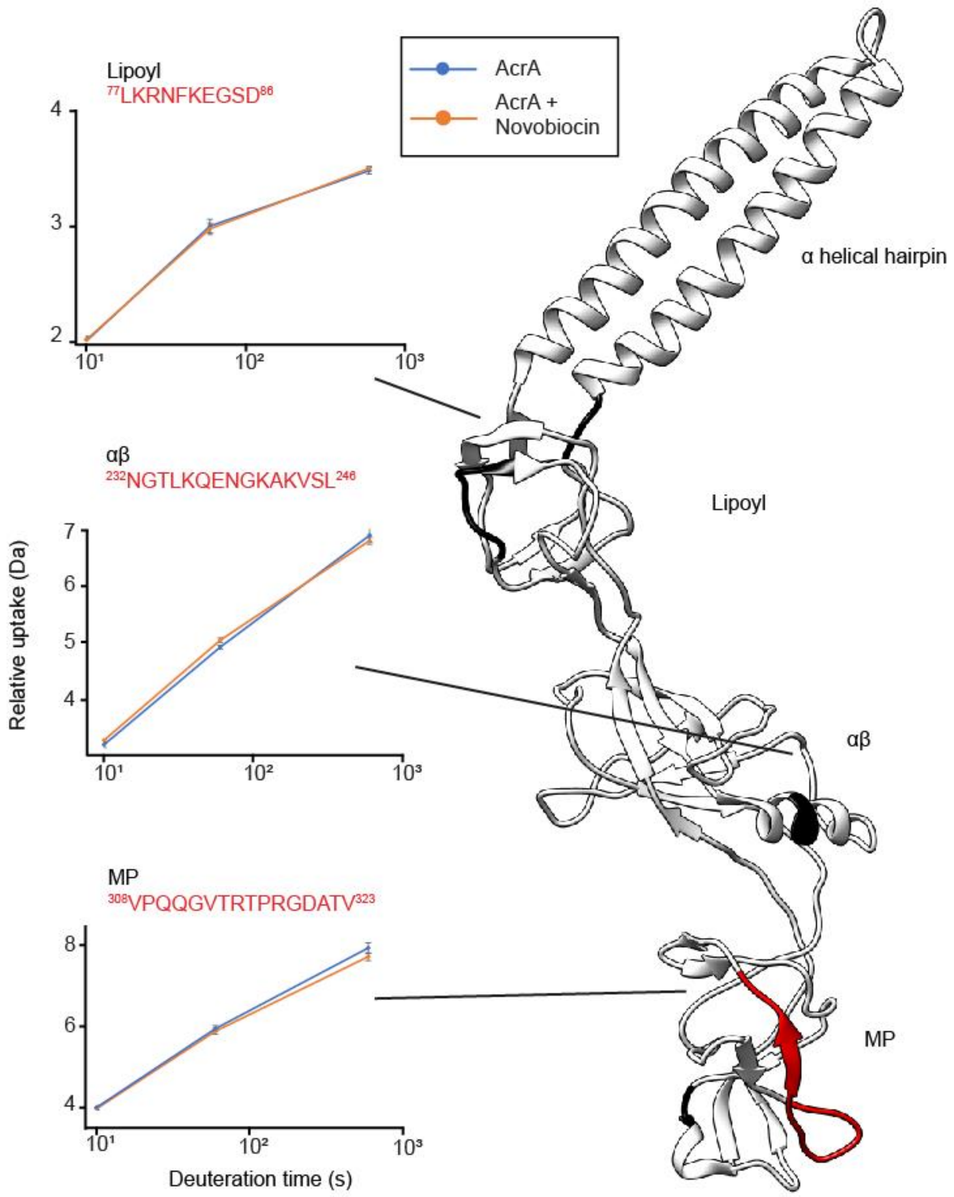

Figure S7. The effect of novobiocin on AcrA^S structural dynamics. ΔHDX for ((AcrA^S + novobiocin) – AcrA^S) for the latest time point is painted onto the AcrA structure (PDB:5O66) using HDeXplosion and Chimera.[4–6] We defined significance to be ≥ 0.34 Da change (see Methods) with a P-value ≤ 0.01 in a two-tailed Welch's *t*-test (*n* = 4 technical replicates). White areas represent regions with insignificant ΔHDX, and black areas represent regions with no peptide coverage. Three peptide uptake plots are shown, in areas that saw significant protection with NSC 60339. Uptake plots are the average deuterium uptake and error bars indicate the standard deviation (n = 4 technical replicates). Source data provided as a Source Data file.

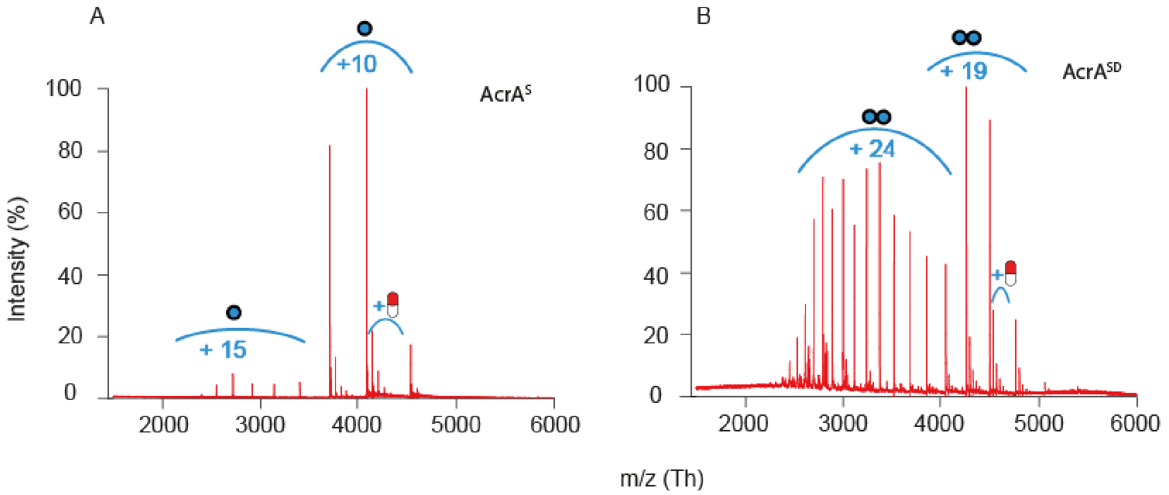

Figure S8. Native-MS of AcrA$^{S/SD}$ constructs and novobiocin at pH 6.0. A. Native-MS characterisation of AcrA^S construct with novobiocin at pH 6.0. Protein buffer exchanged to 100 mM ammonium acetate prior to MS and protein diluted to 10 µM. Novobiocin added to a concentration of 30 µM, 5% DMSO final. Satellite peaks representing drug binding can be seen adjacent to peaks in the lower charge state distribution. **B.** Native-MS characterisation of AcrA^{SD} construct with novobiocin at pH 6.0. Protein buffer exchanged to 100 mM ammonium acetate prior to MS and diluted to 10 µM. Novobiocin added to a concentration of 100 µM, 10% DMSO final. Satellite peaks representing drug binding can be seen adjacent to peaks in the lower charge state distribution. See Supplementary Table 2 for masses.

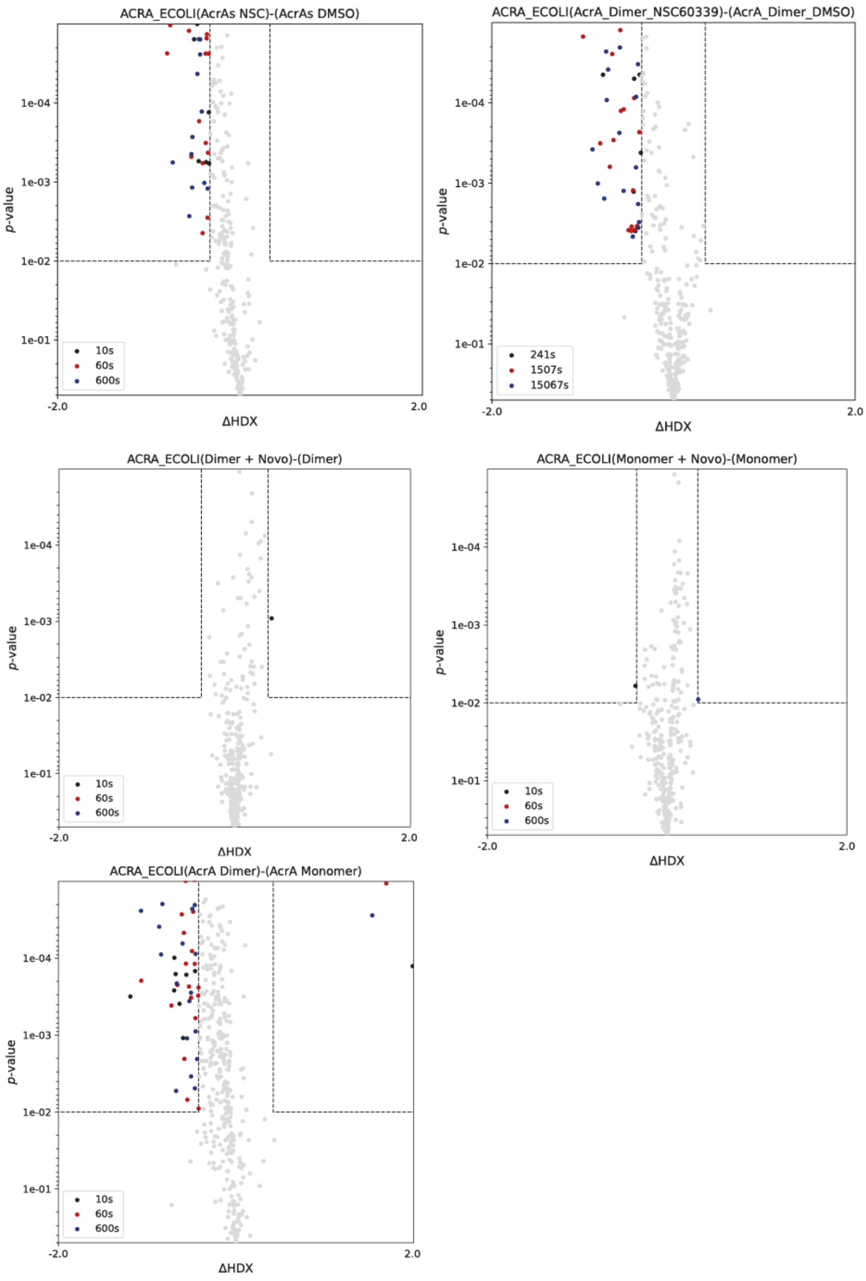

Figure S9. Volcano plots for HDX-MS experiments. ΔHDX cutoff values were calculated using the standard deviation of deuterated peptides for time points performed in quadruplets. We defined significance to be ≥ ΔHDX cutoff (Da) with a P-value ≤ 0.01 in a two-sided Welch's *t*-test (*n* = ≥ 4 technical replicates). Gray plots are insignificant, coloured spots represent significantly changed peptides at a certain time point. See methods for more detail.

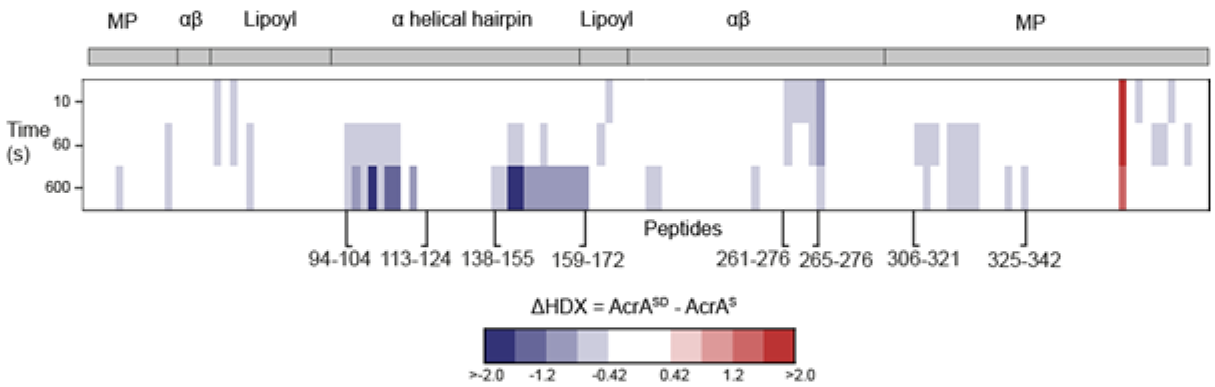

Figure S10. Effects of dimerization on AcrA. Chiclet plot displaying the differential HDX (ΔHDX) plots for $AcrA^{SD}$ - $AcrA^{S}$ for all time points collected. Blue signifies areas with decreased HDX between states. We defined significance to be ≥ 0.42 Da change (see Methods) with a P-value ≤ 0.01 in a two-sided Welch's *t*-test (*n* = 4 technical replicates). White areas represent regions with insignificant ΔHDX. All supporting HDX-MS peptide data can be found in the Source Data file.

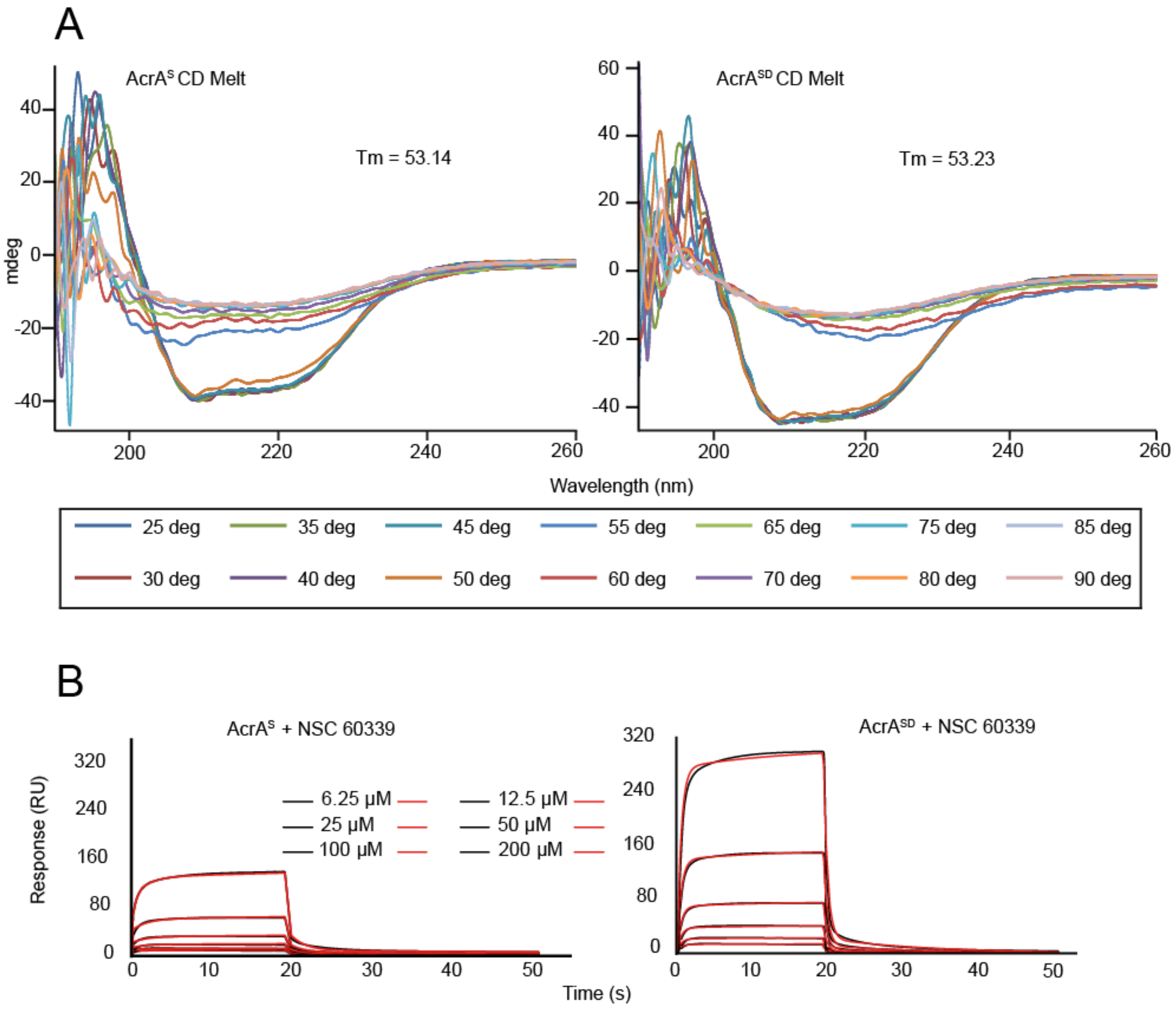

Figure S11. Biophysical characterisation of AcrA^{S} and AcrA^{SD}. A. Circular dichroism thermal melts of AcrA^{S} and AcrA^{SD}. Proteins diluted to 0.4 mg ml^{-1} and CD measured from 190-260 nm in a 0.5 mm pathlength cell, from 25 ˚C to 90 ˚C, at 5 ˚C intervals. Tm calculated using the values at 222 nm. **B.** Surface plasmon resonance of AcrA^{S} and AcrA^{SD} + NSC 60339. The sensorgrams (black lines) were collected at indicated concentrations of NSC 60339.

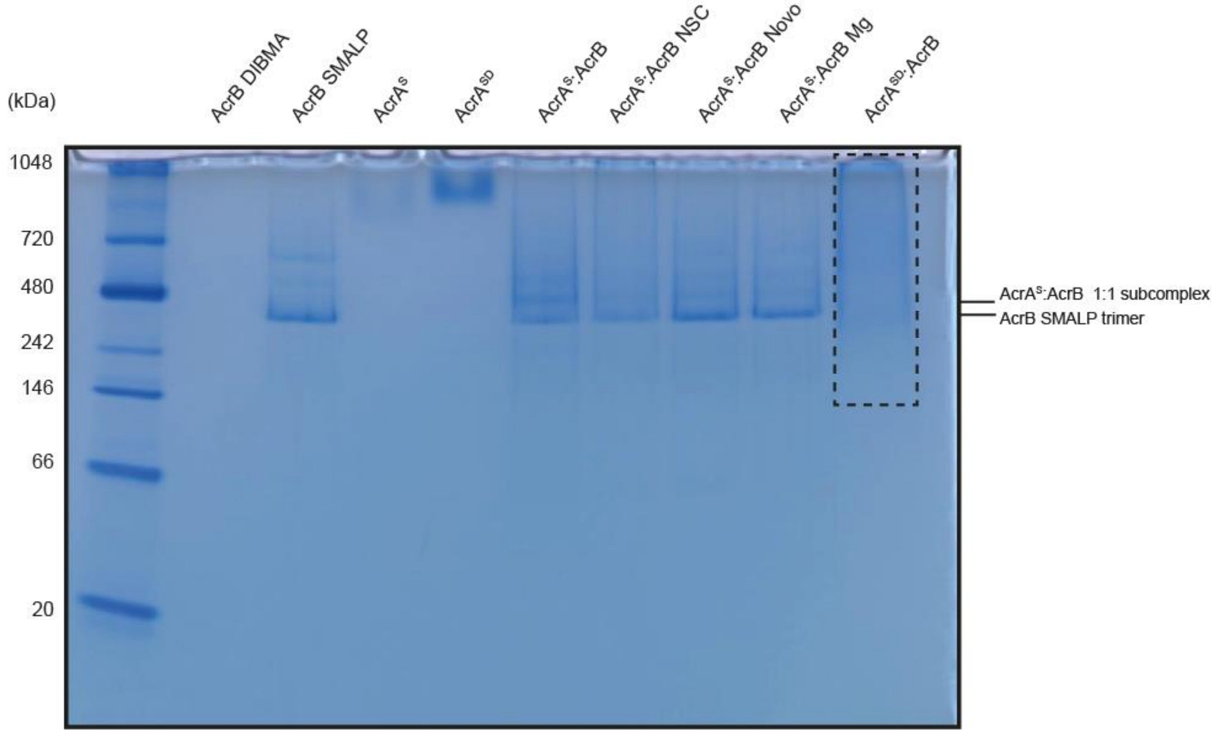

Figure S12. SMA-PAGE of AcrA constructs and AcrB SMALPs. Proteins loaded at 2 µM, NSC 60339 at 500 µM, novobiocin at 30 µM, Mg^{2+} at 1 mM. AcrA^{S}:AcrB subcomplex can be seen at 1:1 ratio. AcrA^{SD}:AcrB shows various different stoichiometries, which is highlighted by the dotted box. Gel ran as described in the methods. This gel was representative of results seen (n =3).

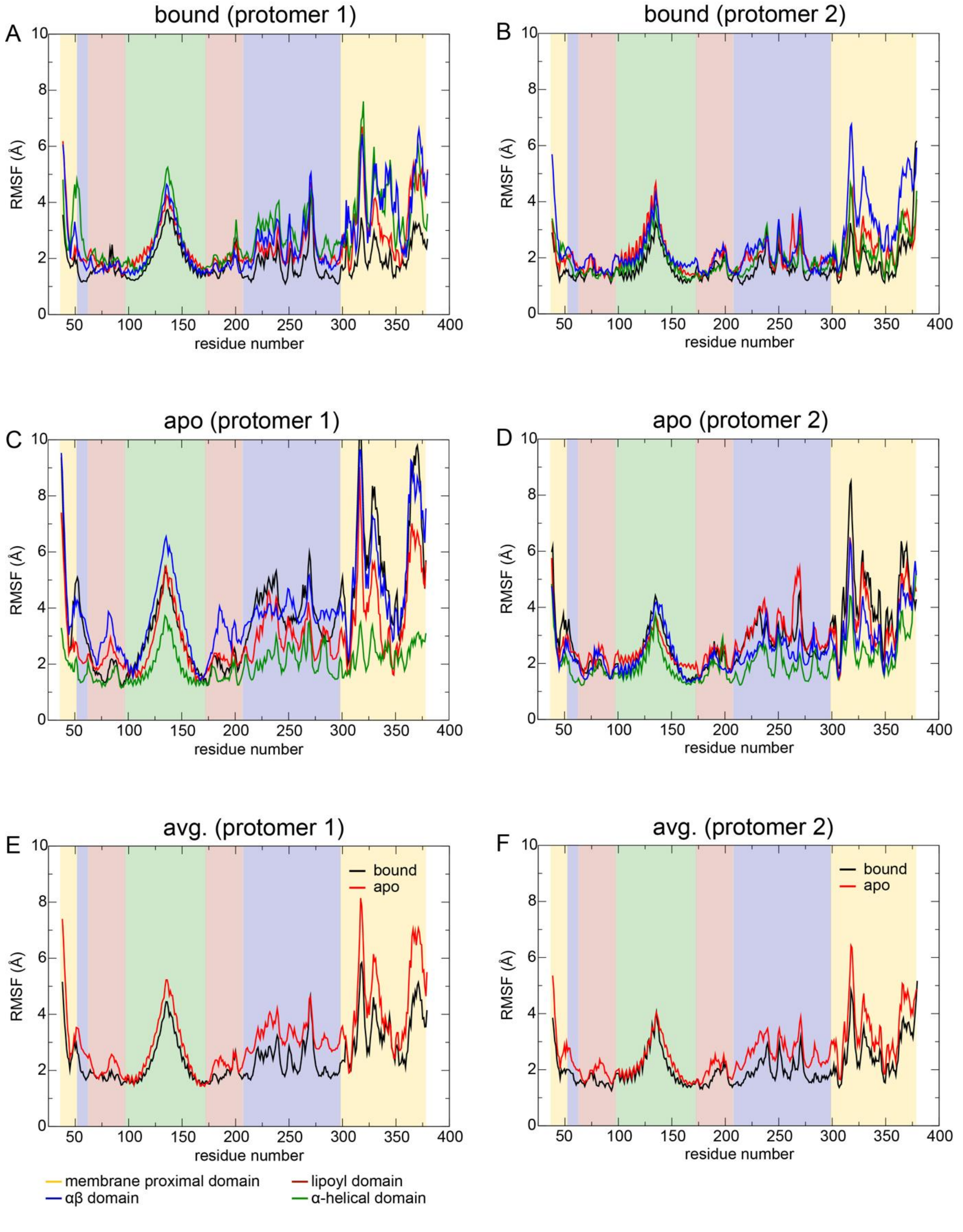

Figure S13. RMSF from MD simulations of AcrA^{SD}. RMSF of AcrA^{SD} from four independent simulations for the (A,B) bound and (C,D) apo states. RMSF was calculated over the last 70 ns of each 100-ns simulation. (E,F) Average from the four replicas for the bound (black) and apo (red) states. The shading indicates the domains of AcrA as indicated. The first column (A, C, E) is for protomer 1 and the second column (B, D, F) is for protomer 2.

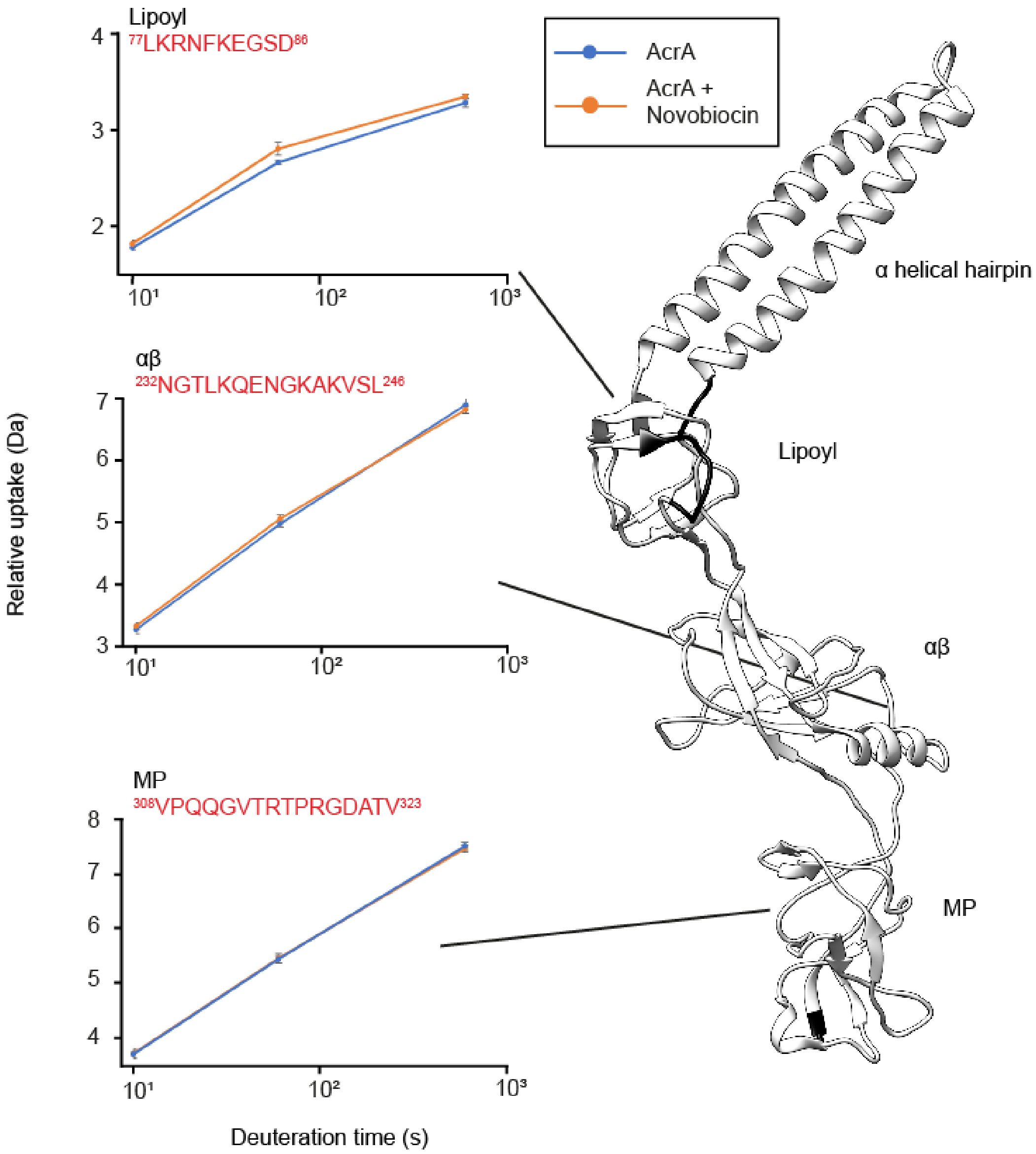

Figure S14. The effect of novobiocin on AcrA^{SD} structural dynamics. ΔHDX for ((AcrA^{SD} + novobiocin) – AcrA^{SD}) for the latest time point is painted onto the AcrA structure (PDB:5O66) using HDeXplosion and Chimera.[4–6] Blue signifies areas with decreased HDX between states. We defined significance to be ≥ 0.38 Da change (see Methods) using a P-value ≤ 0.01 in a two-sided Welch's *t*-test (*n* = 4 technical replicates). White areas represent regions with insignificant ΔHDX, and black areas represent regions with no peptide coverage. Three peptide uptake plots are shown. Uptake plots are the average deuterium uptake and error bars indicate the standard deviation (n = 4 technical replicates). Source data provided as a Source Data file.

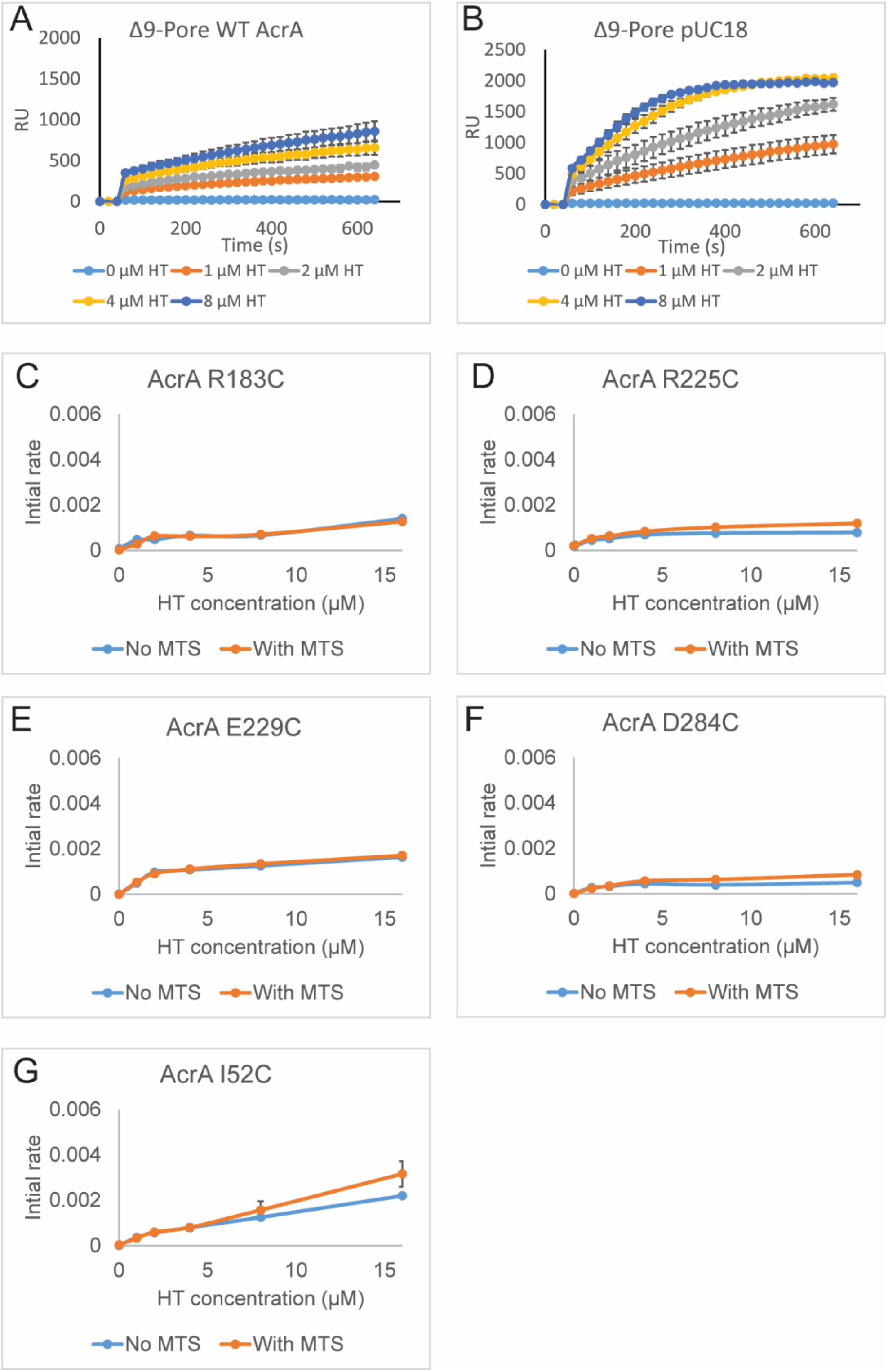

Figure S15. The effect of Cys-reactive MTS probe on the efficiency of AcrAB-TolC. *E. coli* Δ9-Pore cells producing AcrAB-TolC complex carrying the indicated AcrA variants were split into two aliquots and one of the aliquots was treated with a Cys-reactive probe MTS. After incubation for 15 min at 37C, cells were washed and the intracellular accumulation of Hoechst was analysed as described previously.[49] A, B: Representative time courses of HT accumulation in Δ9-Pore cells with WT AcrA (A) and empty vector (B). C-G: Kinetic data were fitted into a burst-single exponential decay function and the calculated initial rates of Hoechst accumulation (µM/s) were plotted as a function of the externally added concentration of Hoechst. Error bars represent standard deviation (n = 3 independent measurements) for all.

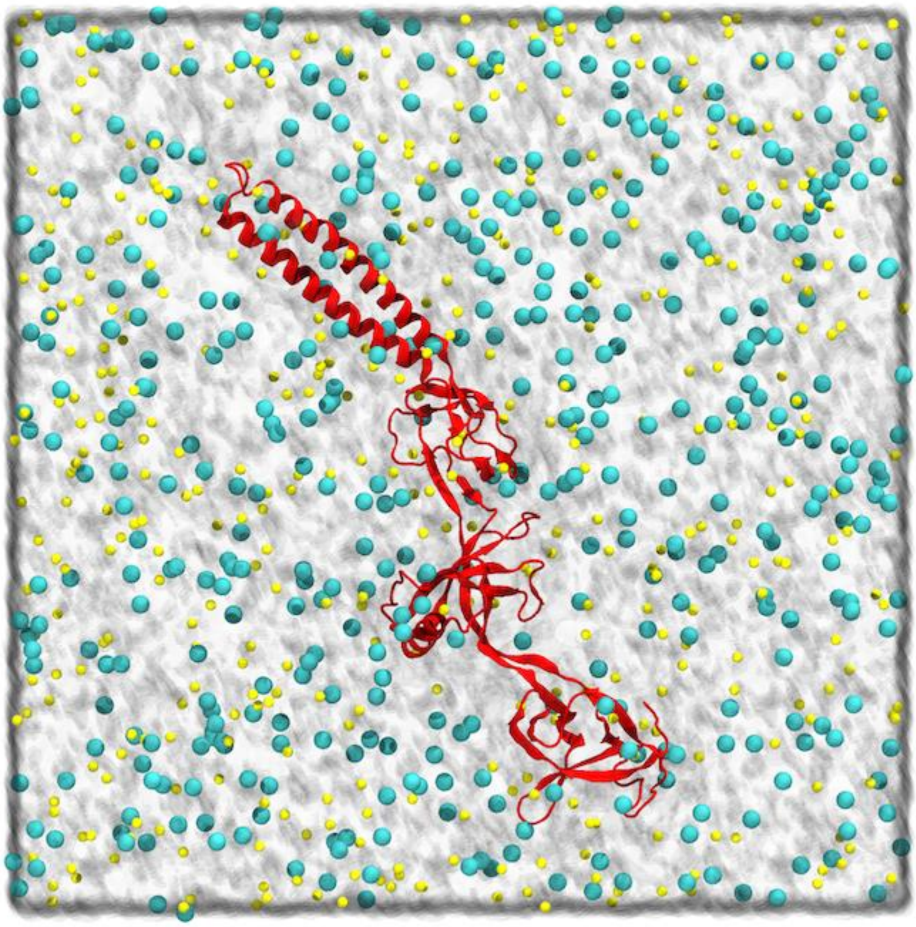

Figure S16. Full system used for simulation of AcrA^S. A cubic water box, 170 Å on a side, was used for simulations to allow for tumbling of AcrA without the need for orientational restraints. In addition to protein, the system contains 156674 water molecules and 442 Na^+ and 443 Cl^- ions.

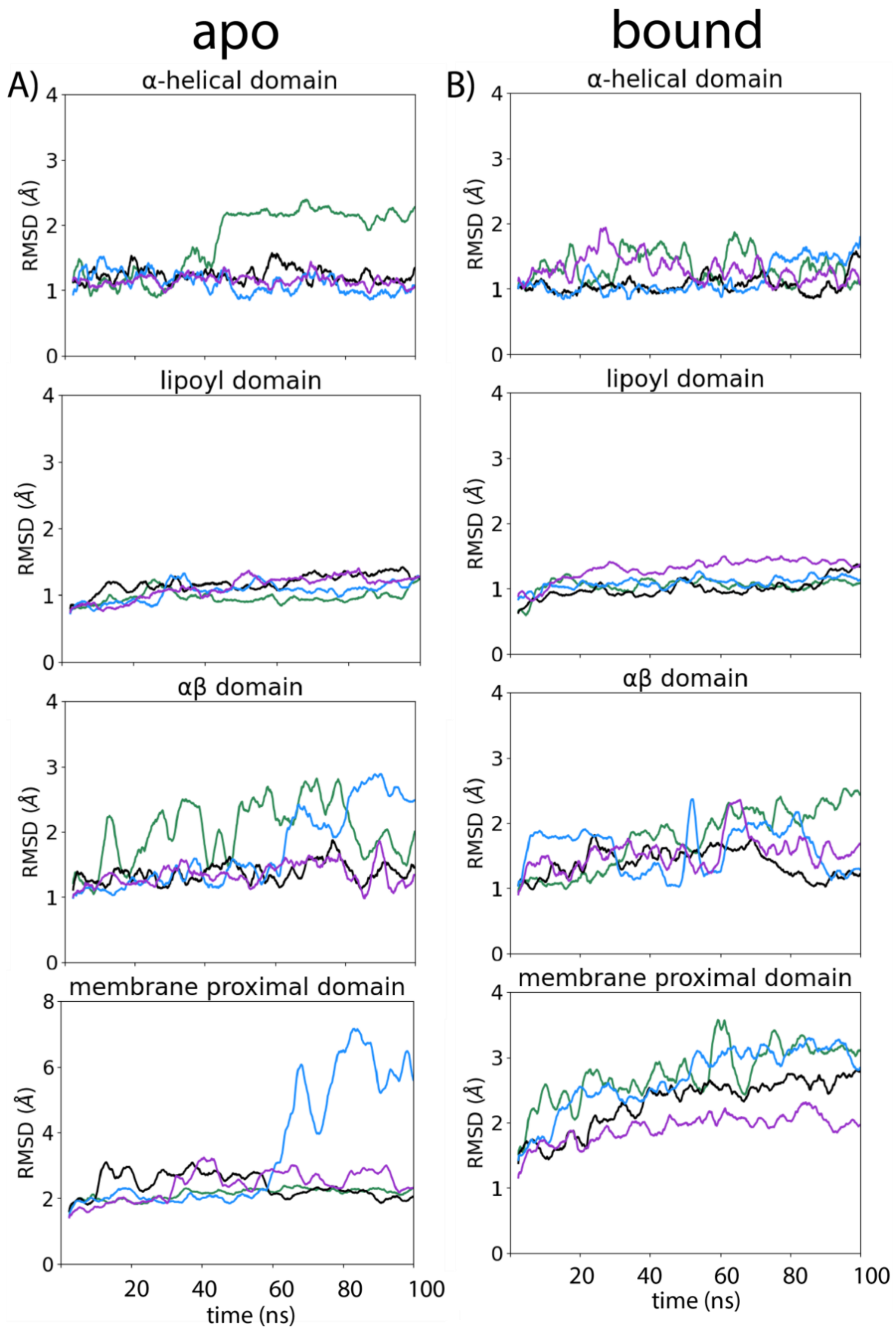

Figure S17. Root-mean-square deviation (RMSD) from MD simulations of an AcrA^S. RMSD was calculated for each of the four domains of AcrA independently as labelled from four independent simulations, indicated by different colours. **A.** AcrA^S for the apo state. **B.** AcrA^S for the bound state.

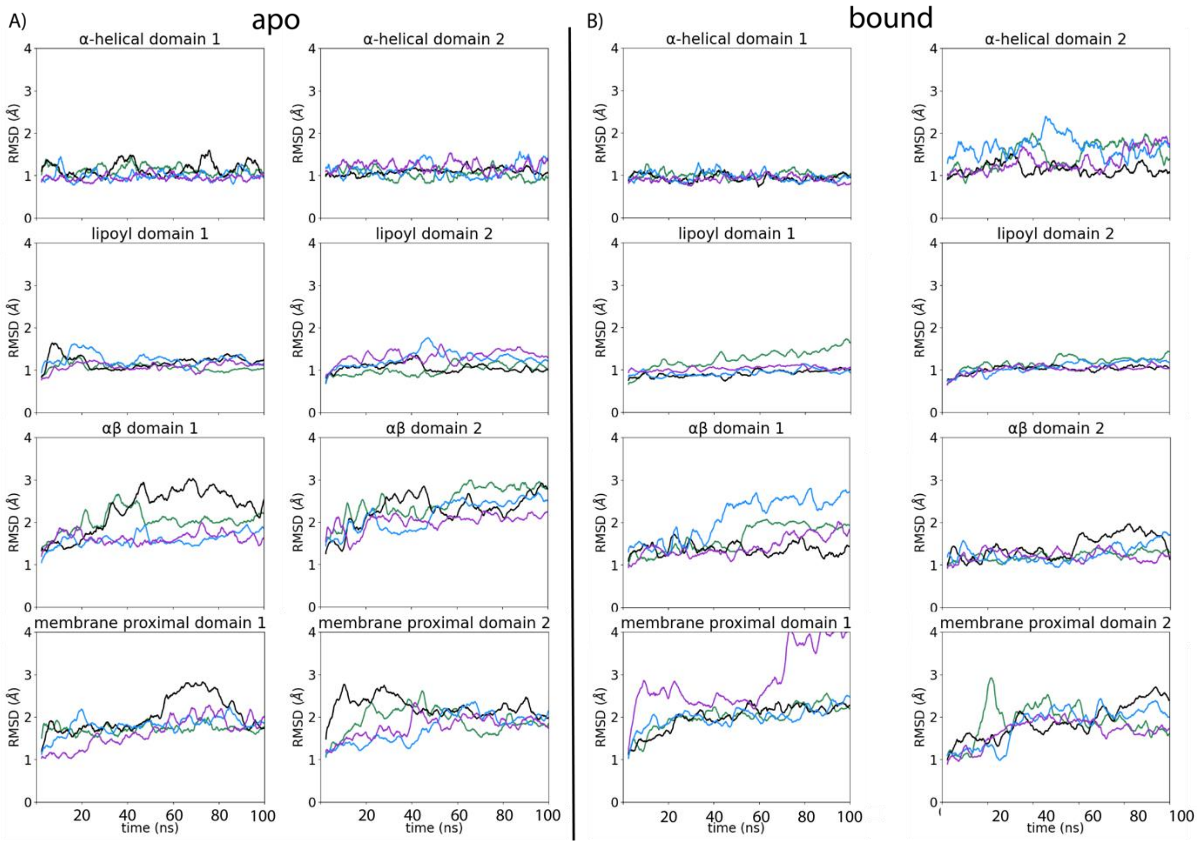

Figure S18. Root-mean-square deviation (RMSD) from MD simulations of AcrA^{SD}. RMSD was calculated for each of the four domains of AcrA independently as labelled from four independent simulations, indicated by different colours, for the apo (left) and bound (right) states. Domain 1 and domain 2 indicate which protomer was analysed. . **A.** AcrA^{S} for the apo state. **B.** AcrA^{S} for the bound state.

Table S1. Native-MS masses table 1. Reported is the standard error of the mean within a single spectrum. Positive mass differences can be attributed to salt and/or detergent adducts.

	Measured mass (Da)	Standard Error (± Da)	Theoretical mass†,* (Da)	Mass difference (Da)
**AcrA^l pH 6.0 Monomer**	41,627	8	41,624	3
**AcrA^l pH 6.0 Dimer**	83,221	2	83,248	-27
**AcrA^l pH 7.4 Monomer**	41,632	7	41,624	8
**AcrA^l pH 7.4 Dimer**	83,274	8	83,248	26
**AcrA^l pH 7.4 Trimer**	124,879	10	124,872	7
**AcrA^l pH 7.4 Tetramer**	166,485	25	166,496	-11
**AcrA^l pH 7.4 Pentamer**	210,023	33	208,120	1903
**AcrA^s pH 6.0 Monomer**	40,849	2	40,817	32
**AcrA^s pH 7.4 Monomer**	40,846	2	40,817	29
***AcrA^{SD} pH 6.0**	81,005	4	80,987	18

†*The theoretical masses for AcrA^l were calculated for AcrA modified with N-acyl-S-diacylglycerol containing two palmitoyl residues and one oleoyl residue.*

**Theoretical masses for AcrA^{SD} construct were amended for fMet processing.*

Table S2. Native-MS masses table 2. Reported is the standard error of the mean within a single spectrum. Positive mass differences can be attributed to salt and/or detergent adducts. Novobiocin mass = 613 Da.

	Measured mass (Da)	Standard Error (± Da)	Mass difference (Da)
**AcrA^S pH 6.0**	40,841	2	-
**+ Novobiocin pH 6.0**	41,458	3	617
**+ Novobiocin pH 6.0 (x2)**	42,067	4	1226
***AcrA^{SD} pH 6.0**	81,000	3	-
**+ Novobiocin pH 6.0**	81,613	10	613
**+ Novobiocin pH 6.0 (x2)**	82,228	4	1228
**+ Novobiocin pH 6.0 (x3)**	82,954	2	1954

Table S3. Minimal inhibitory concentrations of Δ9-Pore cells

Strains	SDS (µg/ml)	NOV (µg/ml)	ERY (µg/ml)	Van (µg/ml)	MTS (µM)
**Δ9-Pore pUC18**	8-16	<1	<1	8-16	>200
**Δ9-Pore p151**	500-1000	64-128	32-64	8	>200
**Δ9-Pore p151 $AcrA_{L50C}B$**	1000->1000	128	32-64	8	>200
**Δ9-Pore p151 $AcrA_{I52C}B$**	1000	128	32-64	8	>200
**Δ9-Pore p151 $AcrA_{T205C}B$**	1000->1000	128	32-64	16	>200
**Δ9-Pore p151 $AcrA_{R225C}B$**	1000	64	32	16	>200
**Δ9-Pore p151 $AcrA_{E229C}B$**	1000	64	32	16	>200
**Δ9-Pore p151 $AcrA_{N232C}B$**	500	64	32	8	>200
**Δ9-Pore p151 $AcrA_{D284C}B$**	>1000	64	32	8	>200
**Δ9-Pore p151 $AcrA_{R183C}B$**	>1000	64	32	16	>200

Table 4. Primers

Protein		Primer	Sequence 5'-3'
**AcrA^{SD}**			
	AcrA (1)	F1	GTCCGCCC ATG GCA GACGACAAACAGGCCCAACAAGGTGGC
		R1	GTCCGCGGATCC**ACCAGAAGAATTACC**AGACTTGGACTGTTC AGGCTGAGC
	AcrA (2)	F1	GCGCGC GGATCC GAC GAC AAA CAG GCC CAA CAA GGT
		R1	GCGGGG CTCGAG AGACTTGGACTGTTCAGGCTGAGCA

Supplementary References

1. Smit JH, Krishnamurthy S, Srinivasu BY, Parakra R, Karamanou S, Economou A. Probing Universal Protein Dynamics Using Hydrogen-Deuterium Exchange Mass Spectrometry-Derived Residue-Level Gibbs Free Energy. *Anal Chem*. 2021;93(38):12840-12847. doi:10.1021/acs.analchem.1c02155
2. Jumper J, Evans R, Pritzel A, et al. Highly accurate protein structure prediction with AlphaFold. *Nature*. 2021;596(7873):583-589. doi:10.1038/s41586-021-03819-2
3. Varadi M, Anyango S, Deshpande M, et al. AlphaFold Protein Structure Database: Massively expanding the structural coverage of protein-sequence space with high-accuracy models. *Nucleic Acids Res*. 2022;50(D1):D439-D444. doi:10.1093/nar/gkab1061
4. Wang Z, Fan G, Hryc CF, et al. An allosteric transport mechanism for the AcrAB-TolC multidrug efflux pump. *Elife*. 2017;6:1-19. doi:10.7554/eLife.24905
5. Zhang N, Yu X, Zhang X, Arcy SD. Structural bioinformatics HD-eXplosion : visualization of hydrogen – deuterium exchange data as chiclet and volcano plots with statistical filtering. 2021;37(13):1926-1927. doi:10.1093/bioinformatics/btaa892
6. Pettersen EF, Goddard TD, Huang CC, et al. UCSF Chimera — A Visualization System for Exploratory Research and Analysis. *J Comput Chem*. 2004;25:1605-1612. doi:10.1002/jcc.20084
