# Supplementary material for: Conformational restriction shapes the inhibition of a multidrug efflux adaptor protein
Source: Nat Commun. 2023 Jul 18;14:3900. doi: 10.1038/s41467-023-39615-x (PMC10354078; doi:10.1038/s41467-023-39615-x)
Supplement: Supplementary file 3 — Description of Additional Supplementary Information Files [file 41467_2023_39615_MOESM3_ESM.docx]

**Description of Additional Supplementary Information File**

File Name: Supplementary Data 1

Description: HDX-MS summary tables

File Name: Supplementary Data 2

Description: MD simulations data containing the trajectories as PDB files and parameters for NSC 60339 binding

File Name: Supplementary Data 3

Description: Uptake plots (uptake plots are the average deuterium uptake and error bars indicate the standard deviation) from the HDX-MS experiments
