# Supplementary figures and images for: Conformational restriction shapes the inhibition of a multidrug efflux adaptor protein
Source: Nat Commun. 2023 Jul 18;14:3900. doi: 10.1038/s41467-023-39615-x (PMC10354078; doi:10.1038/s41467-023-39615-x)

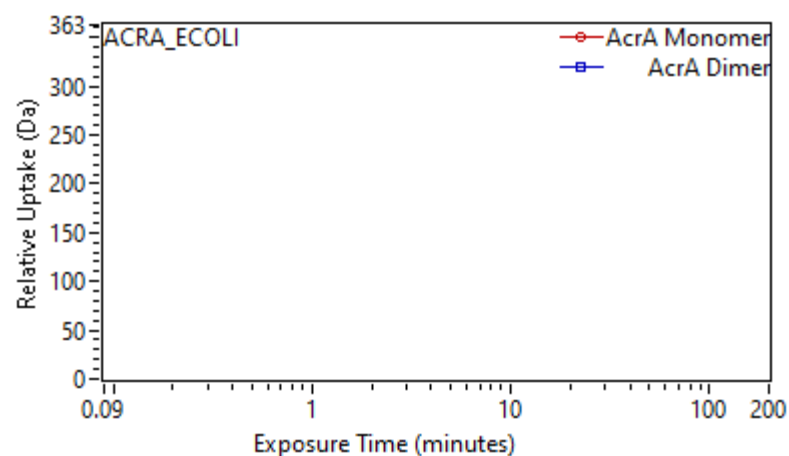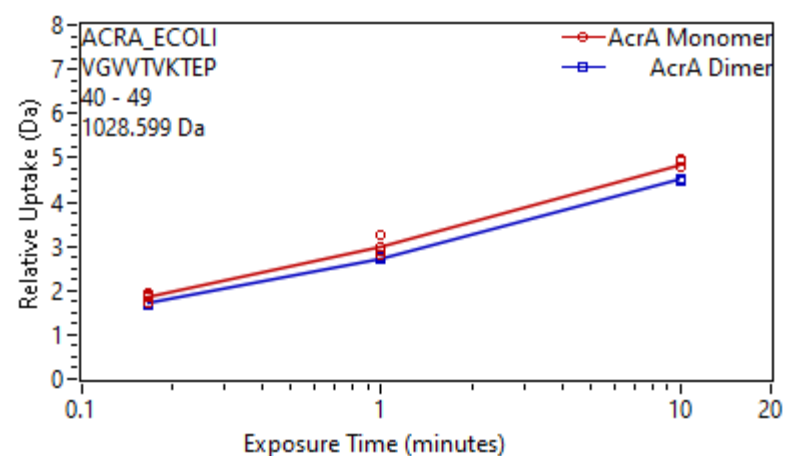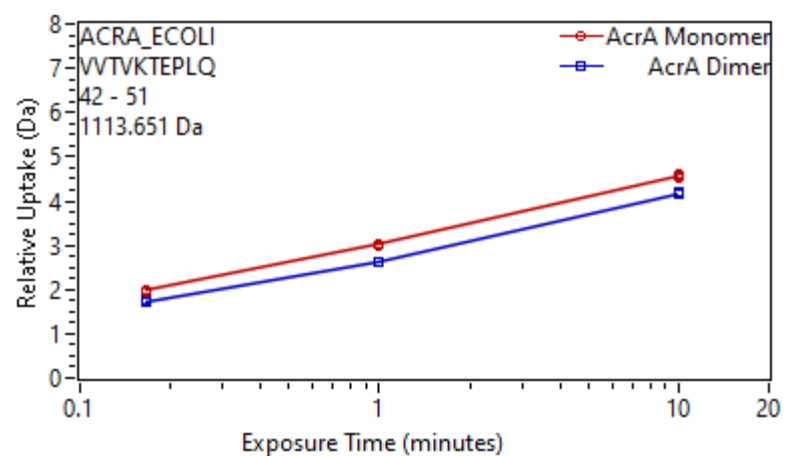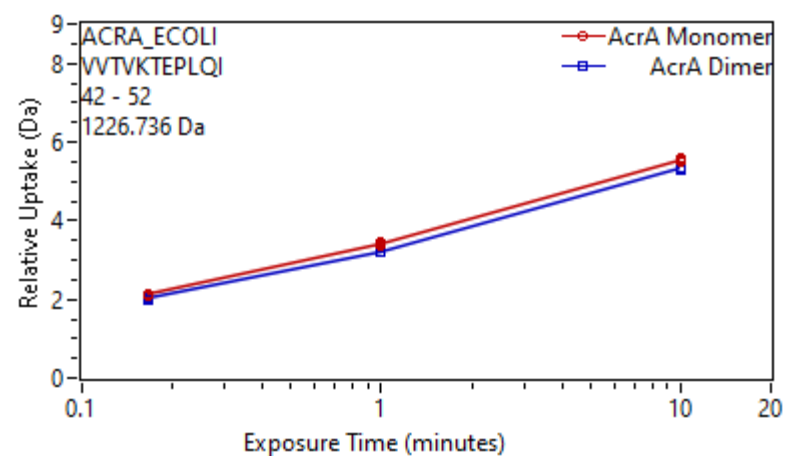

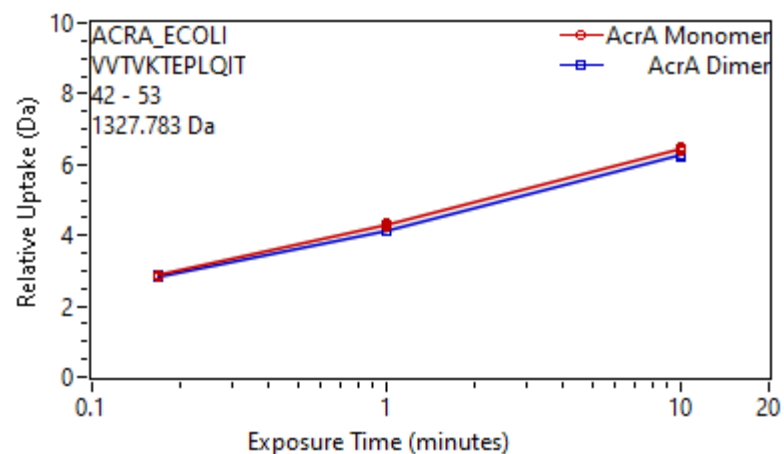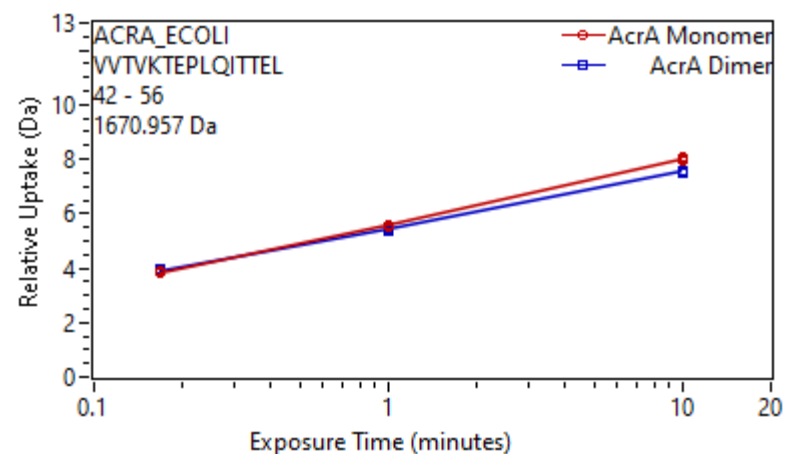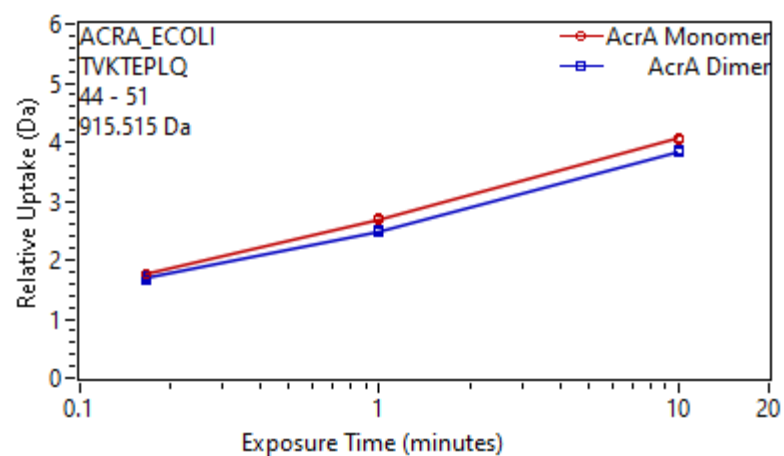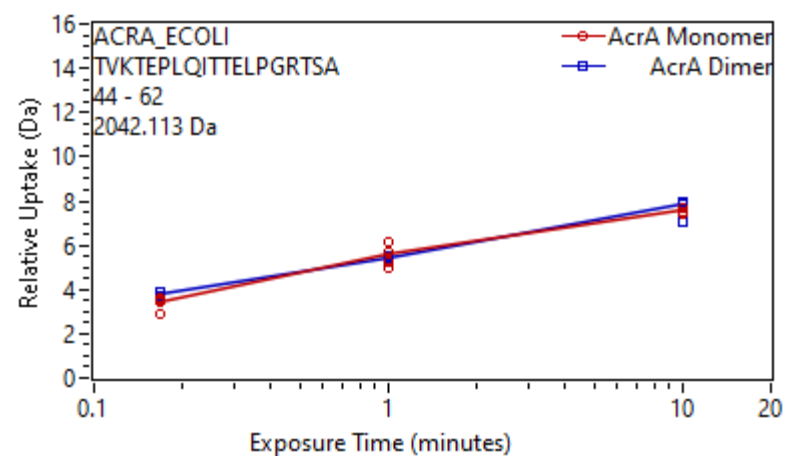

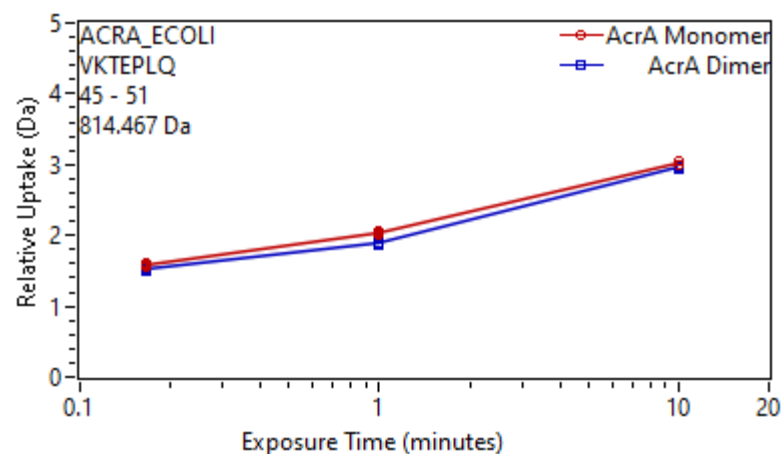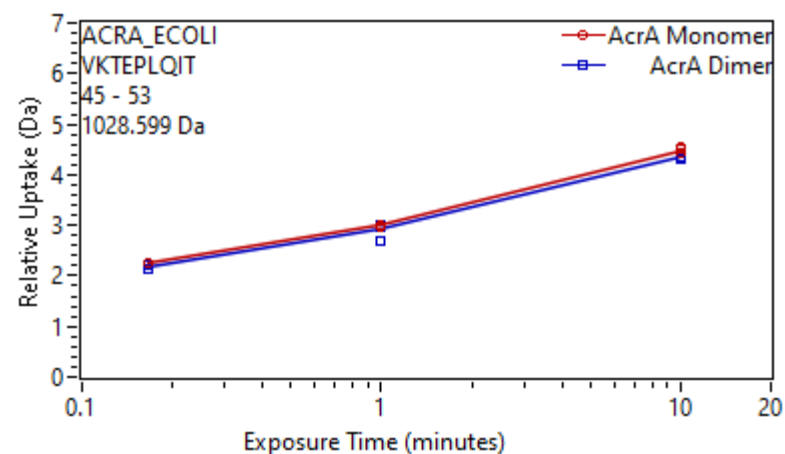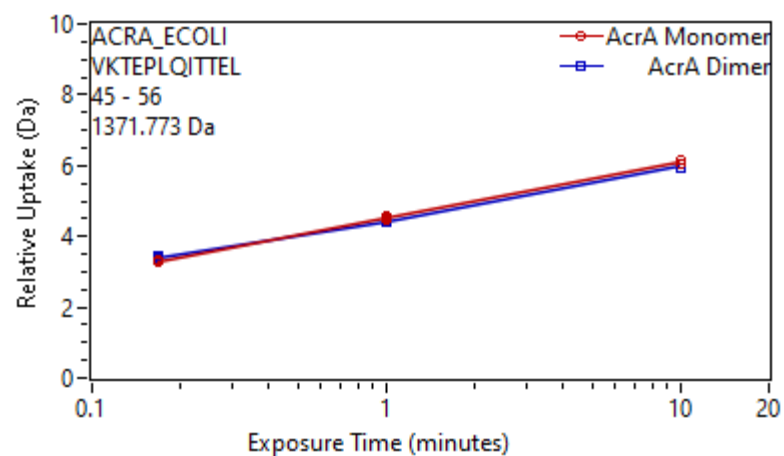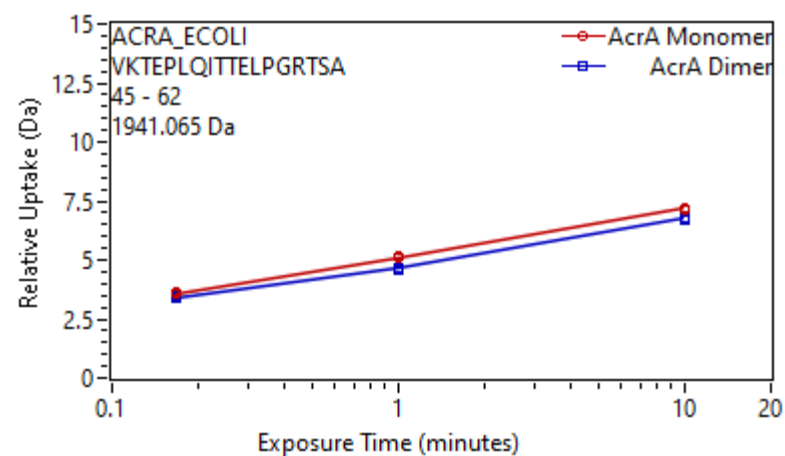

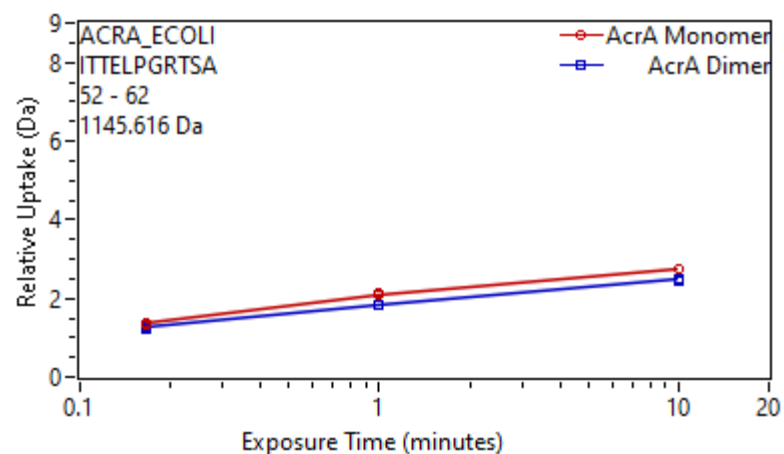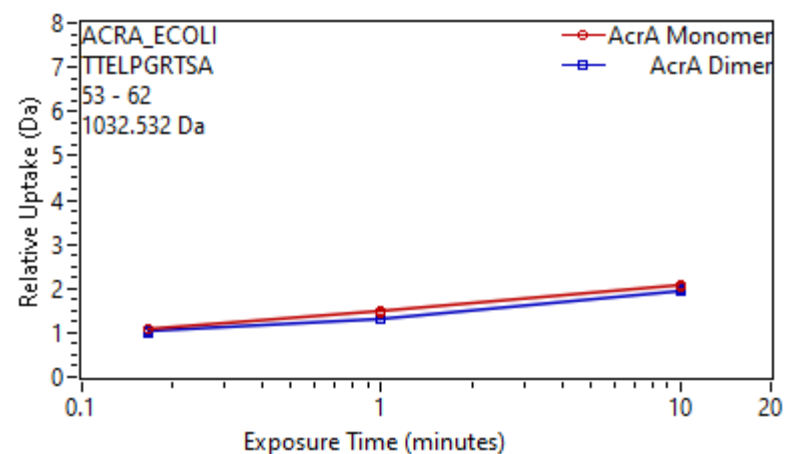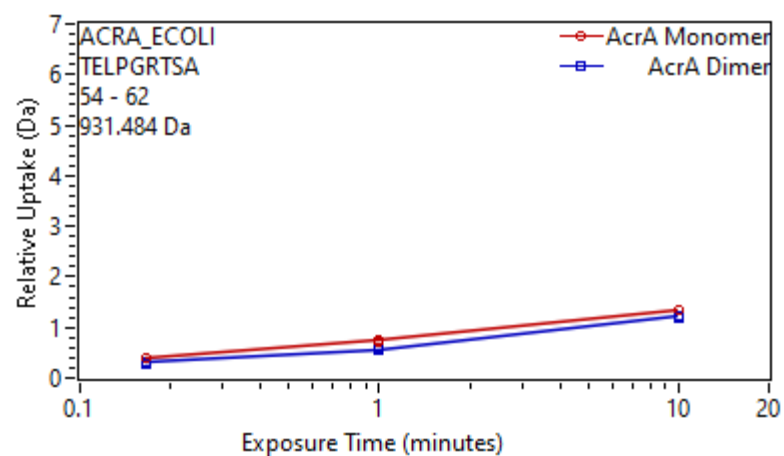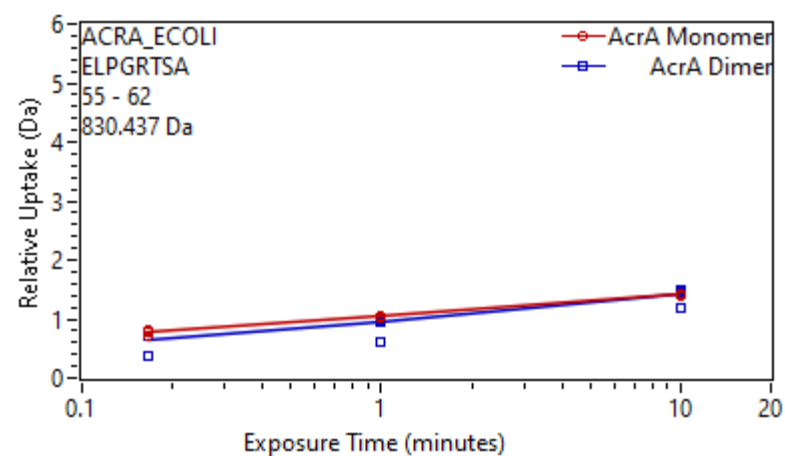

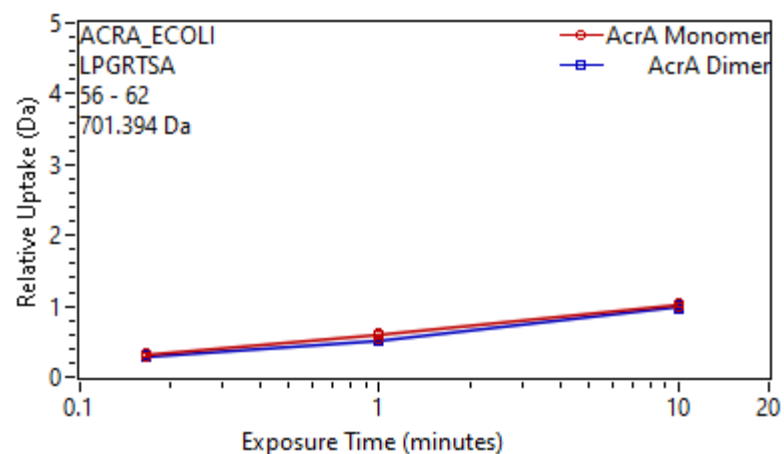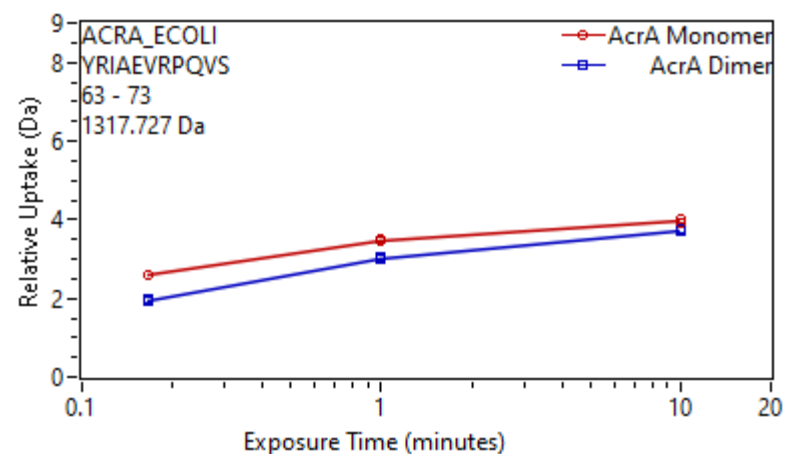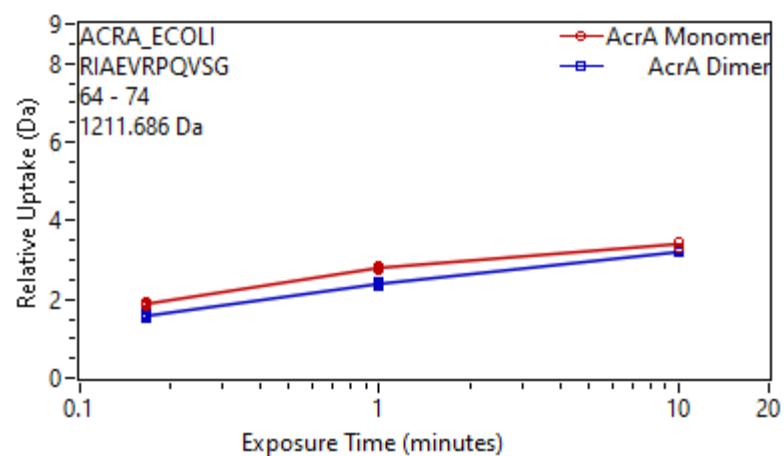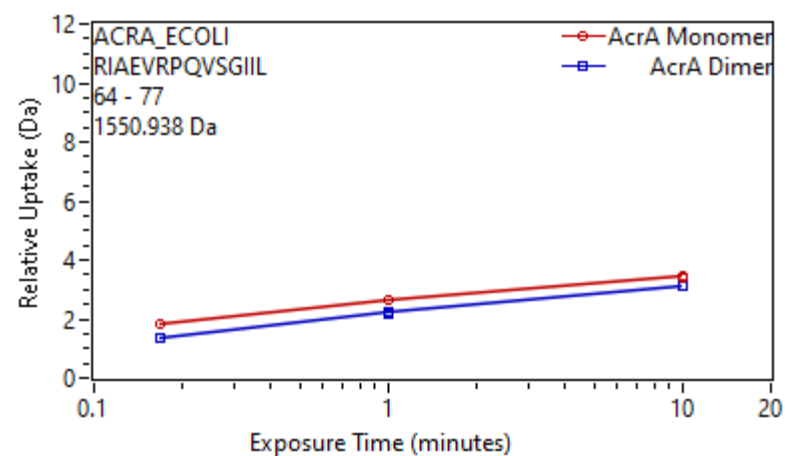

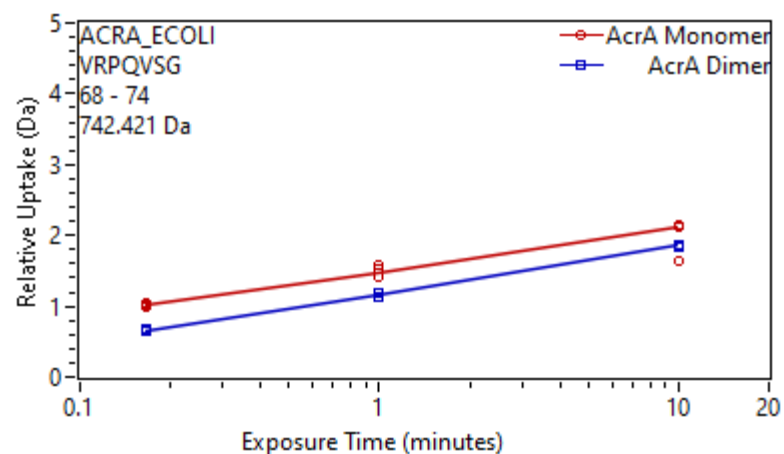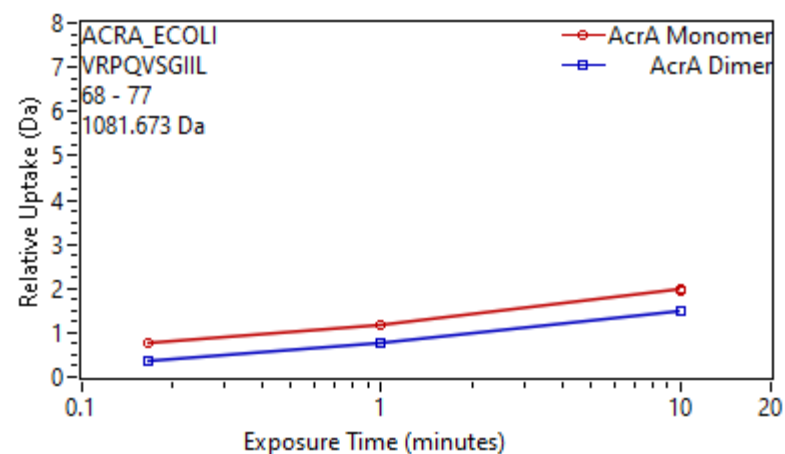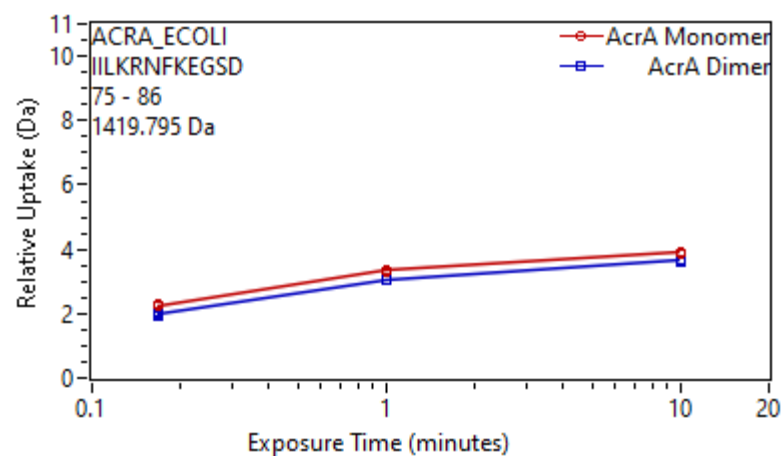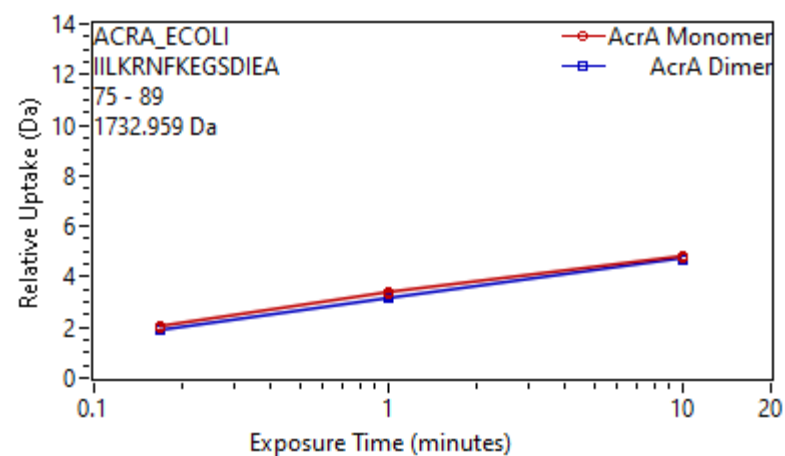

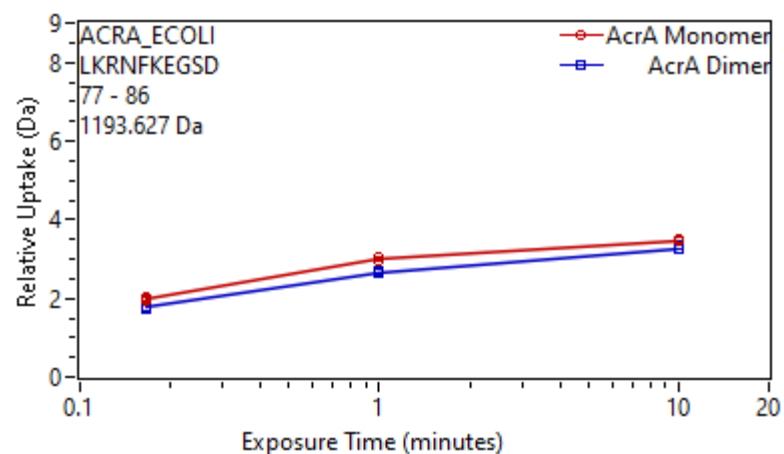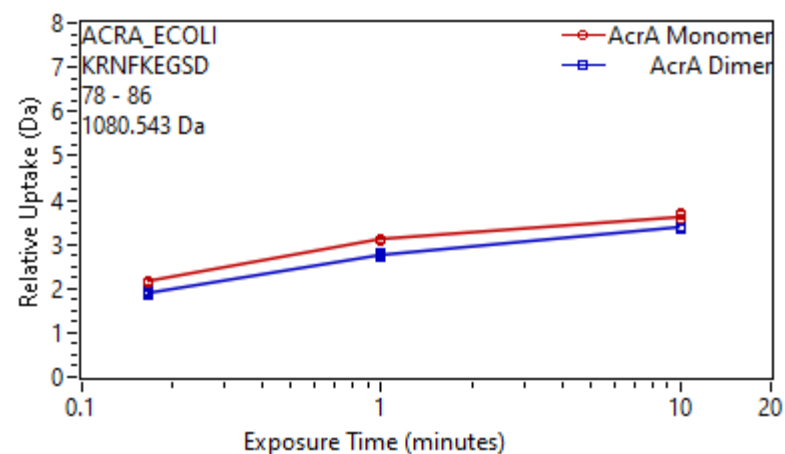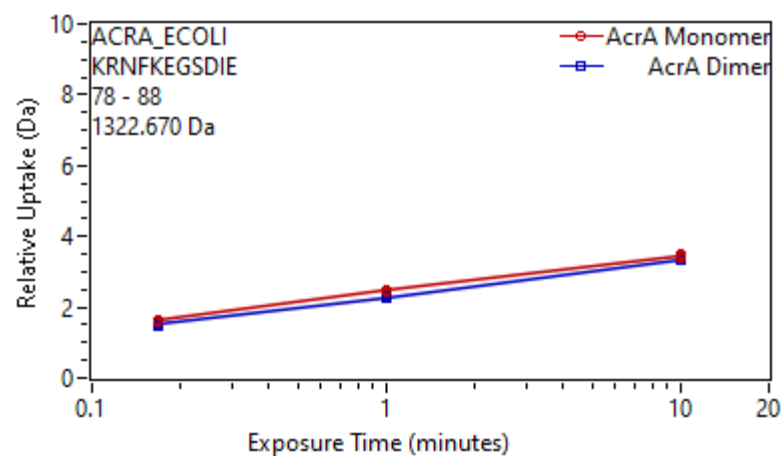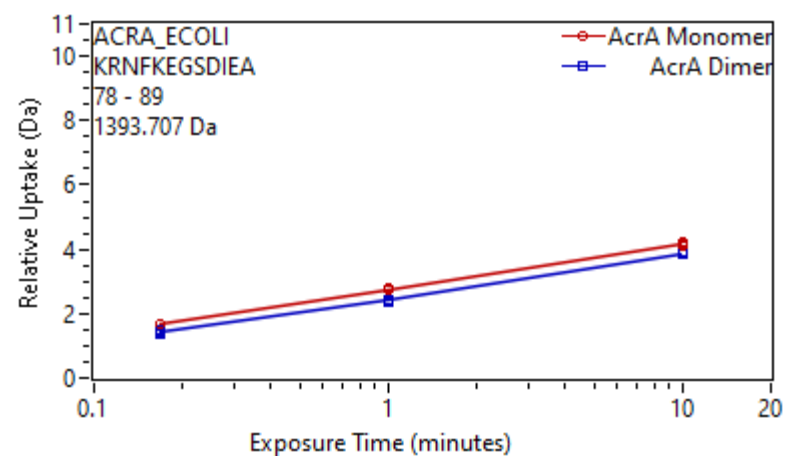

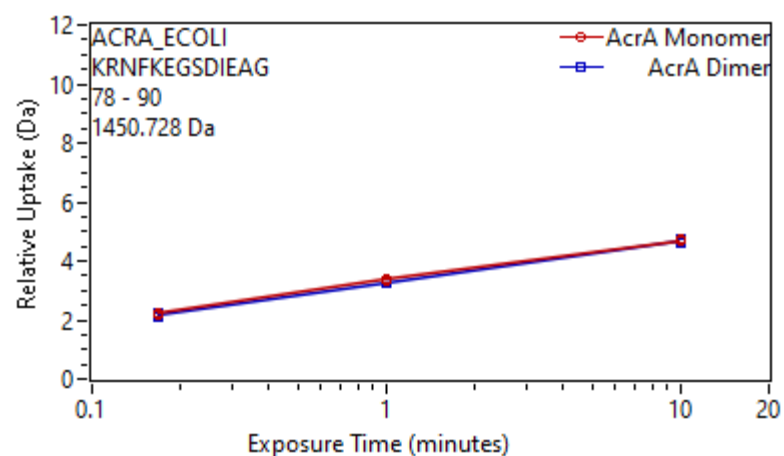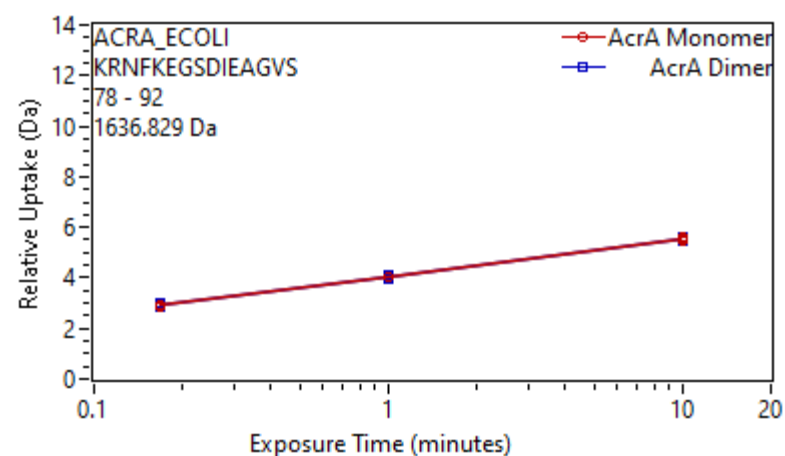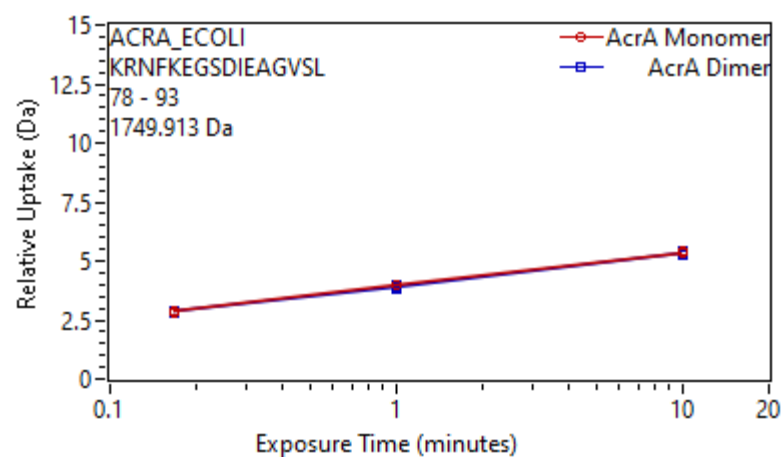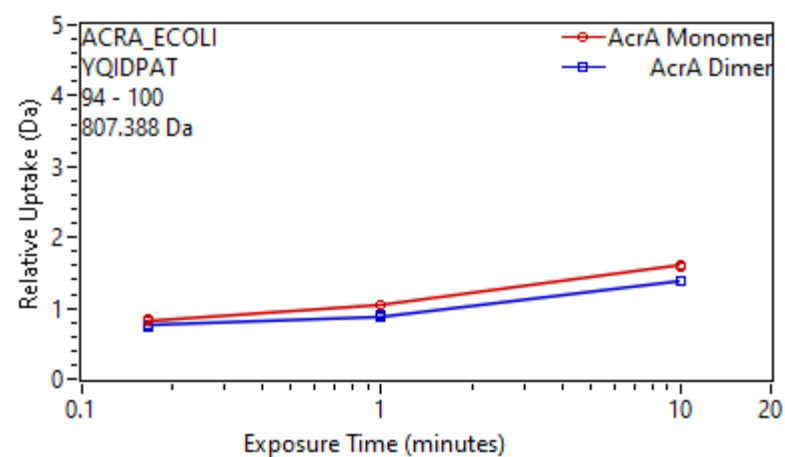

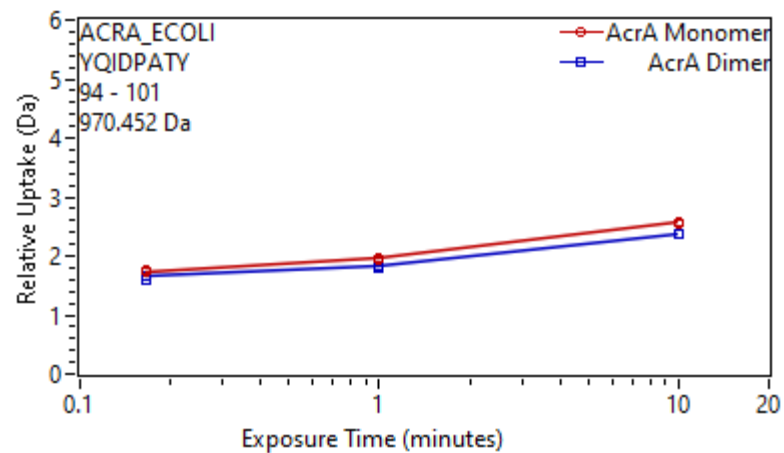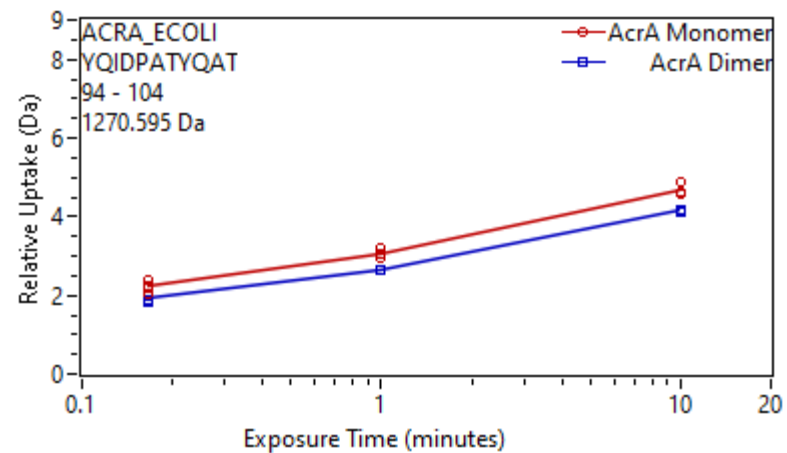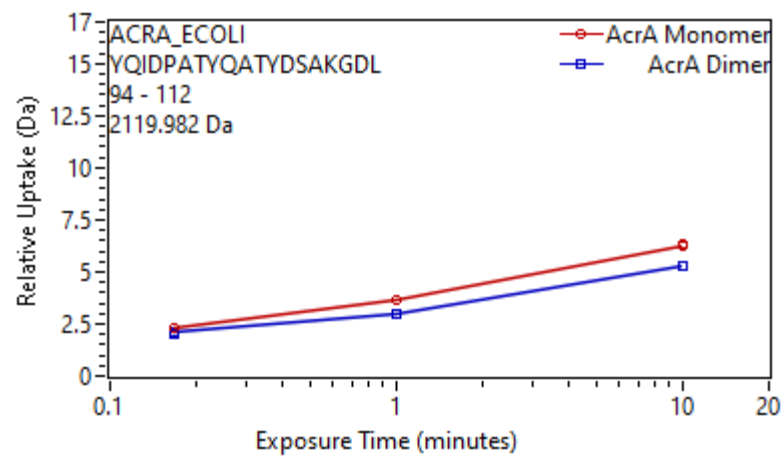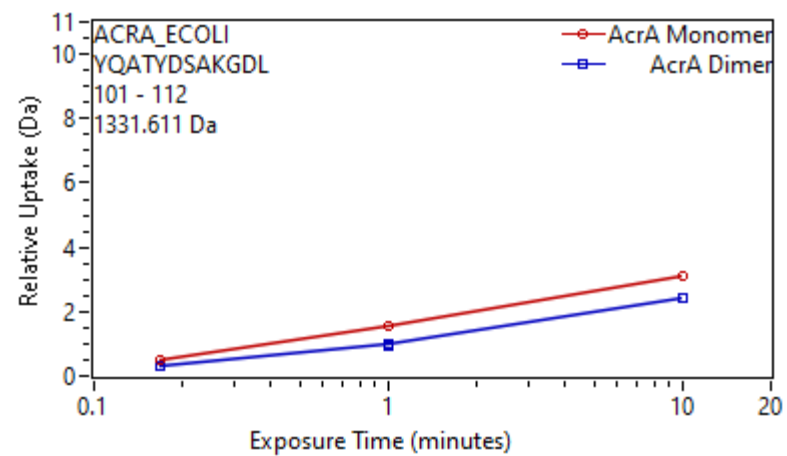

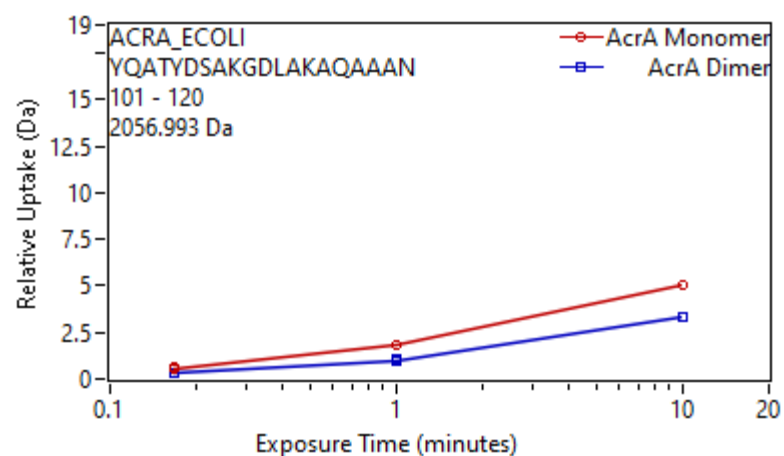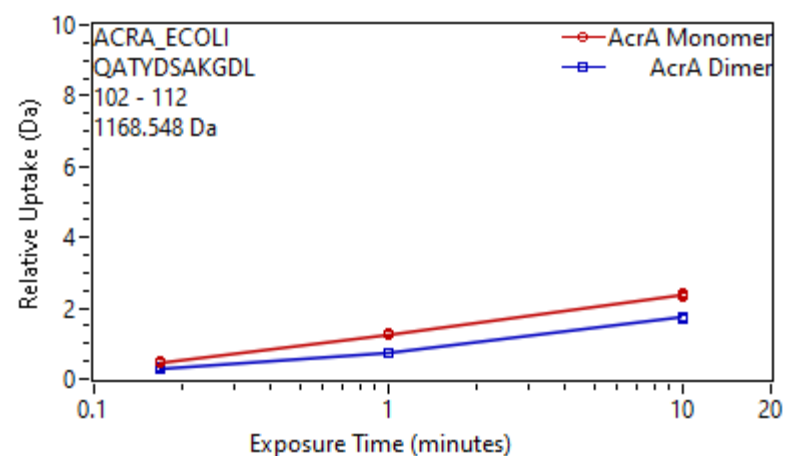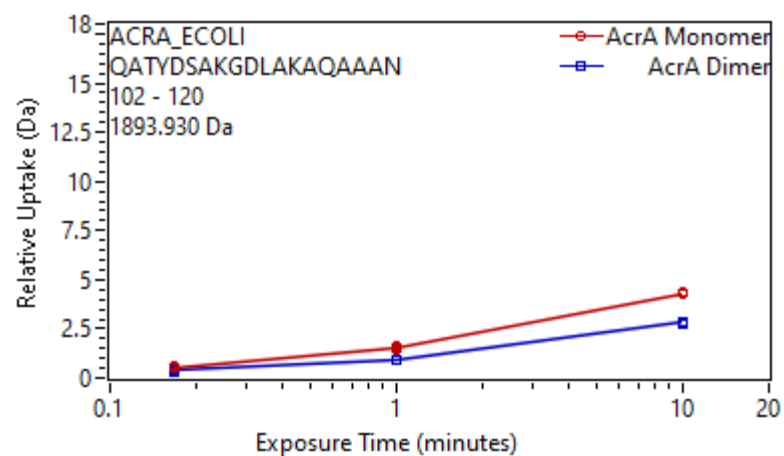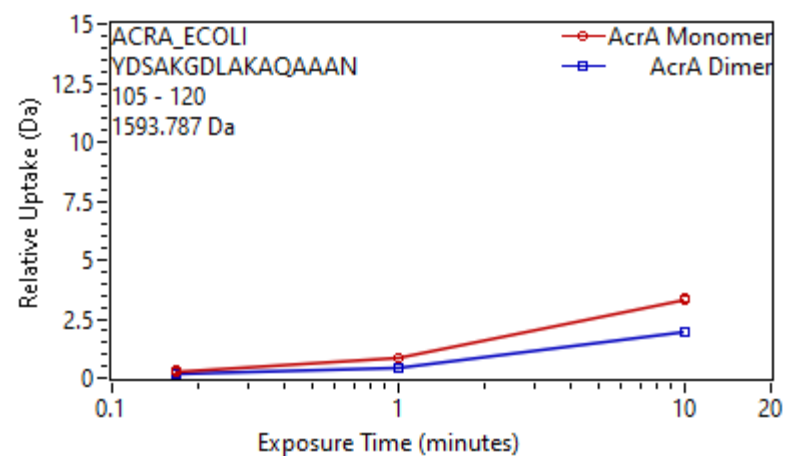

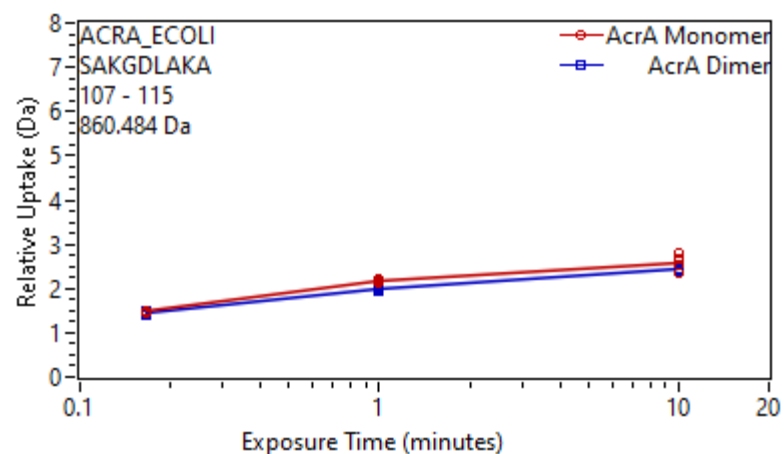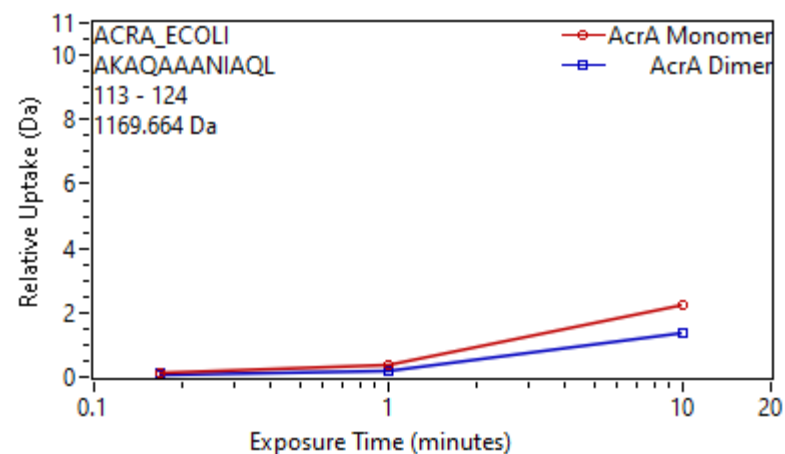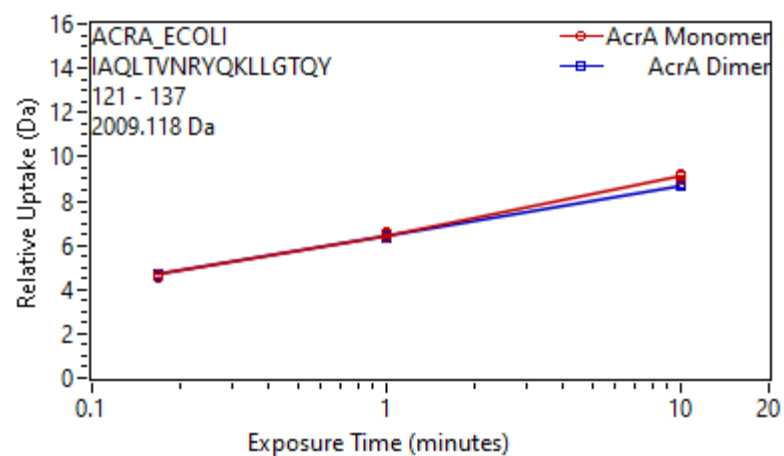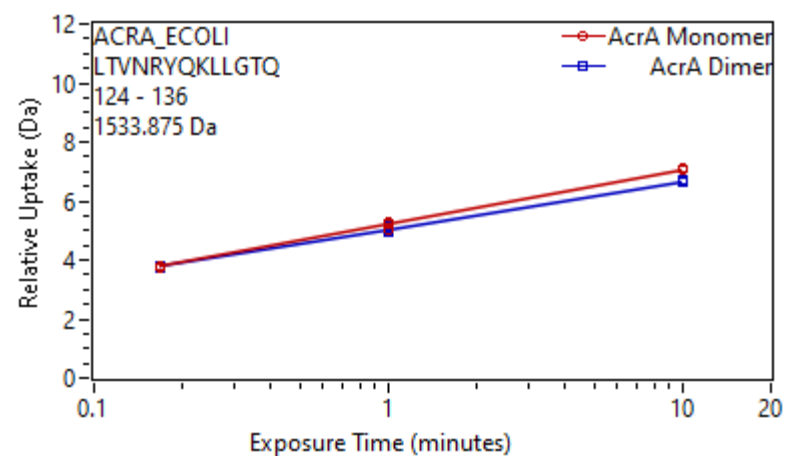

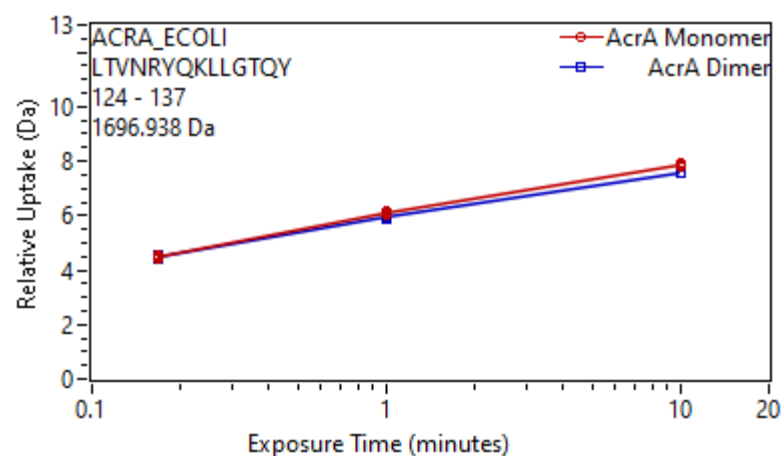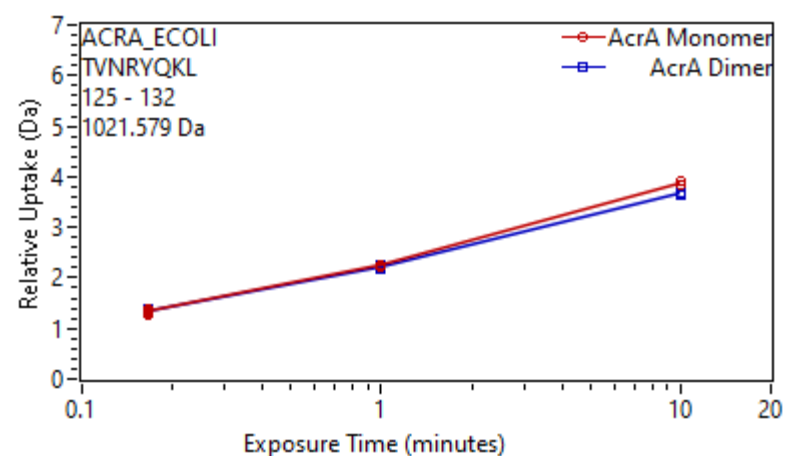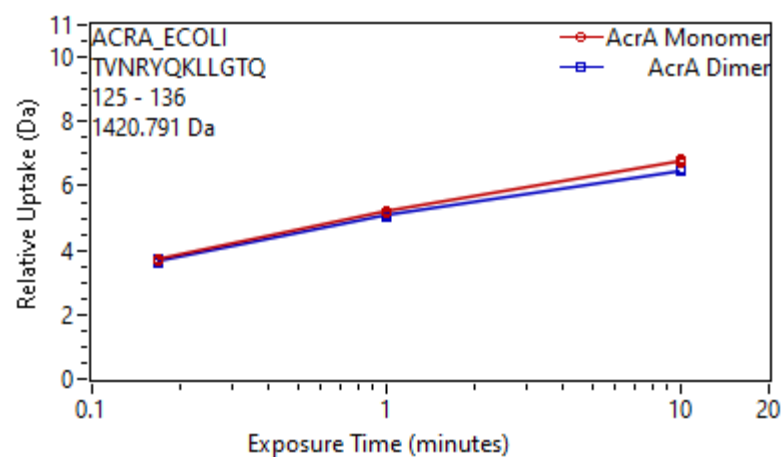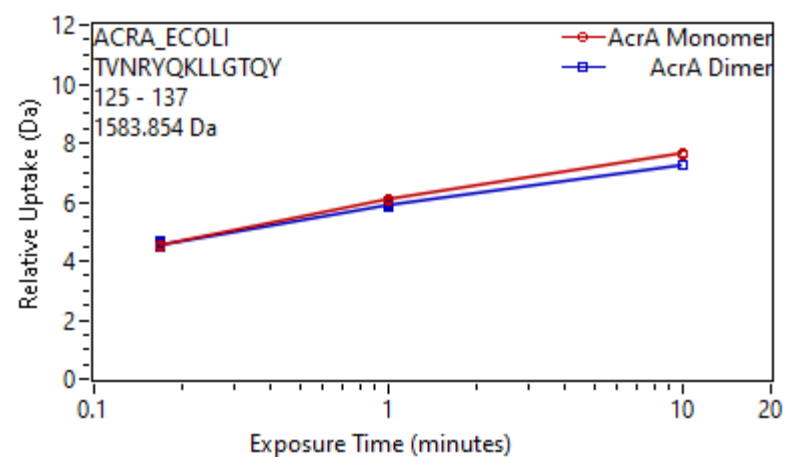

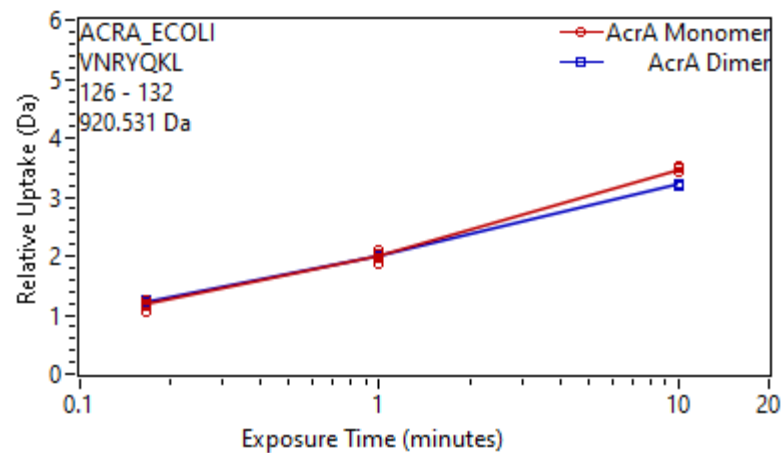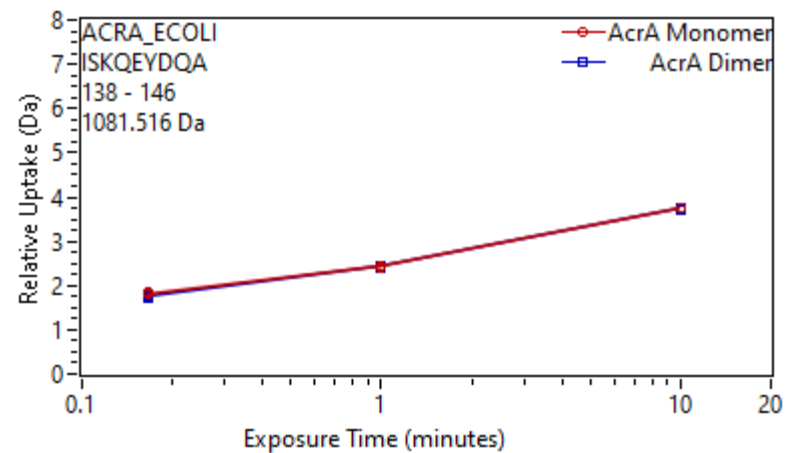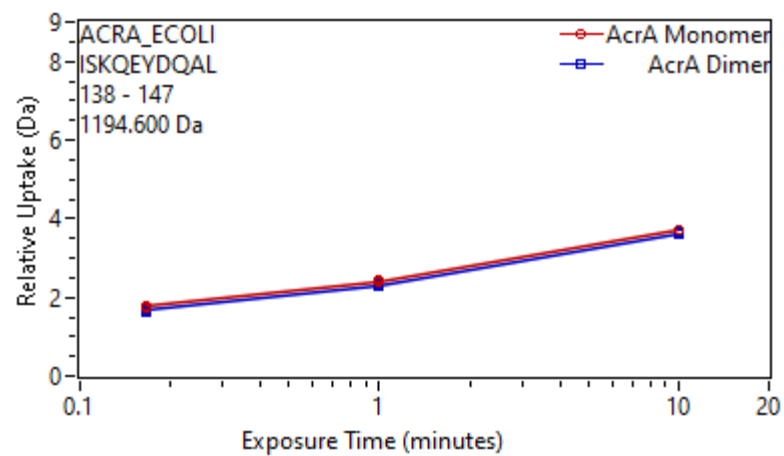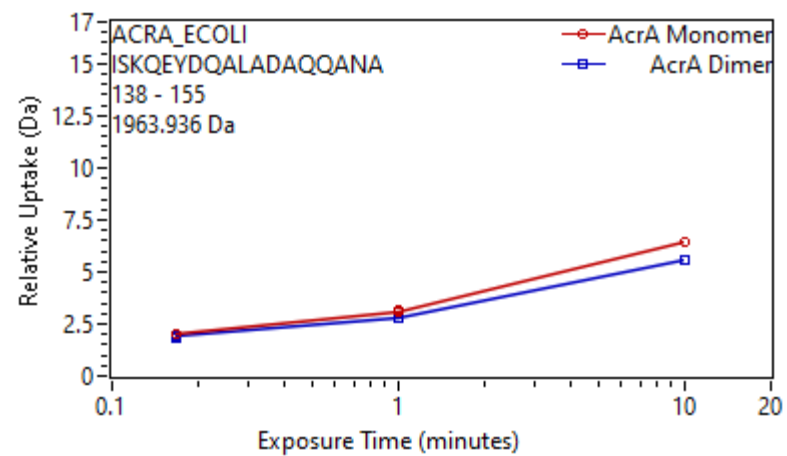

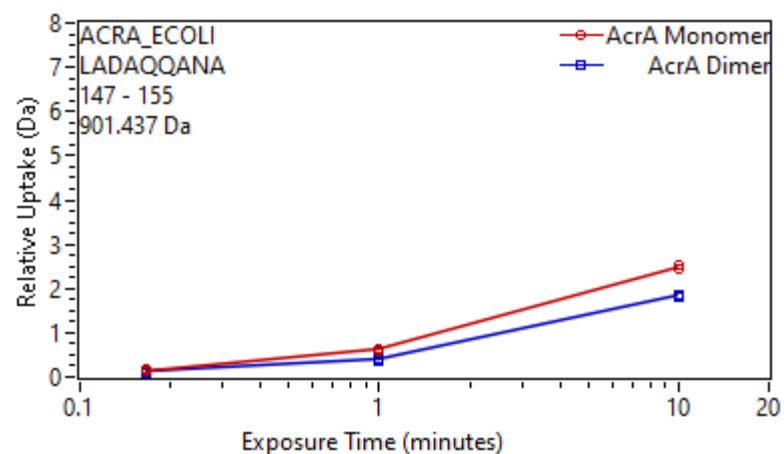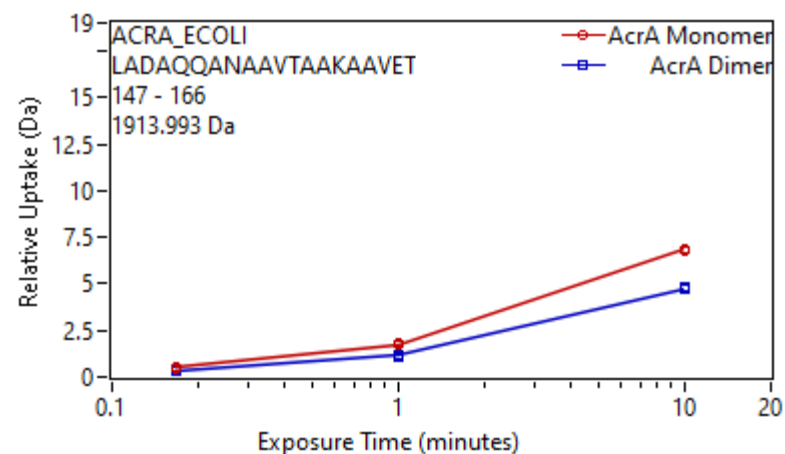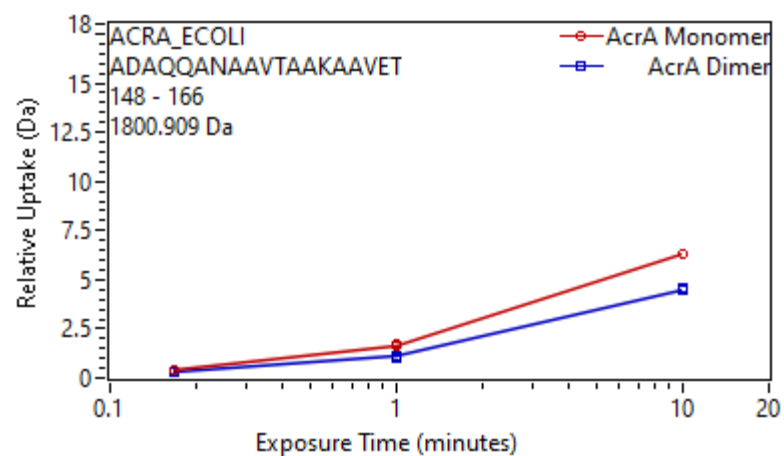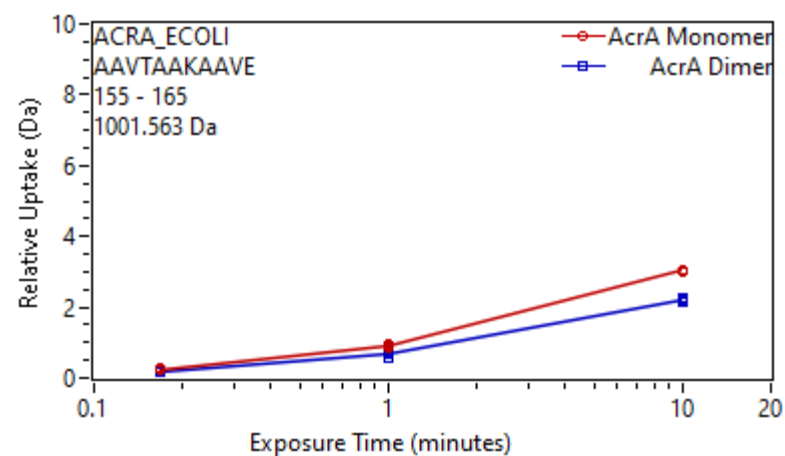

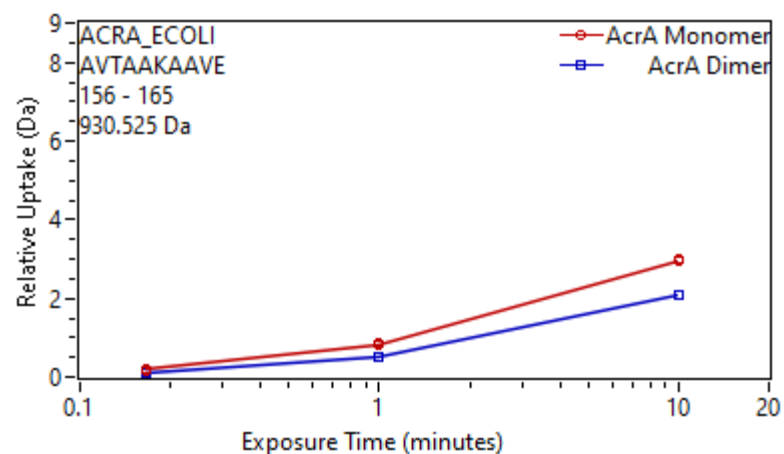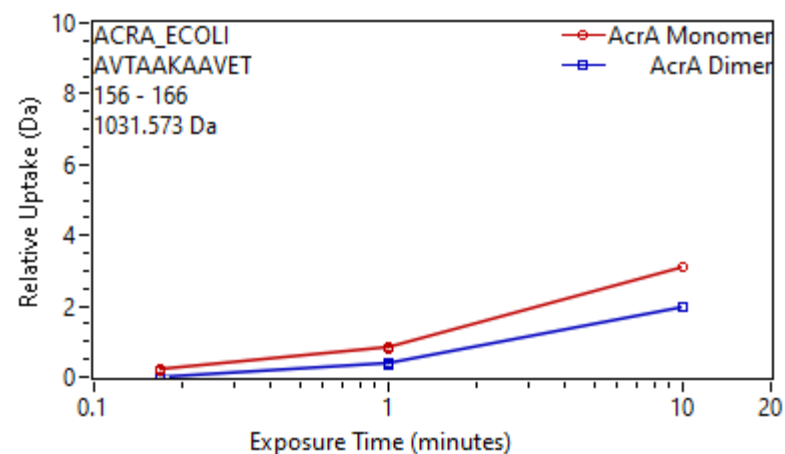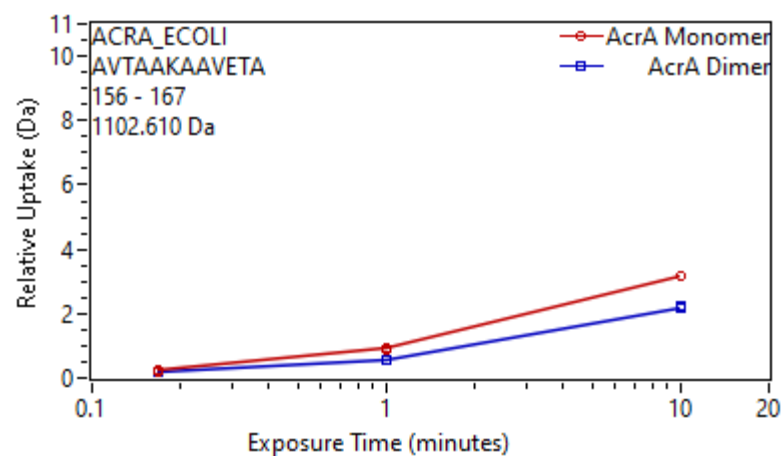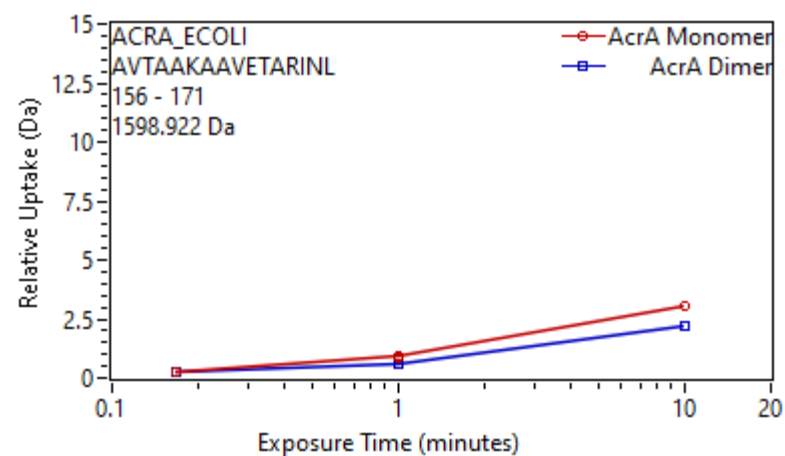

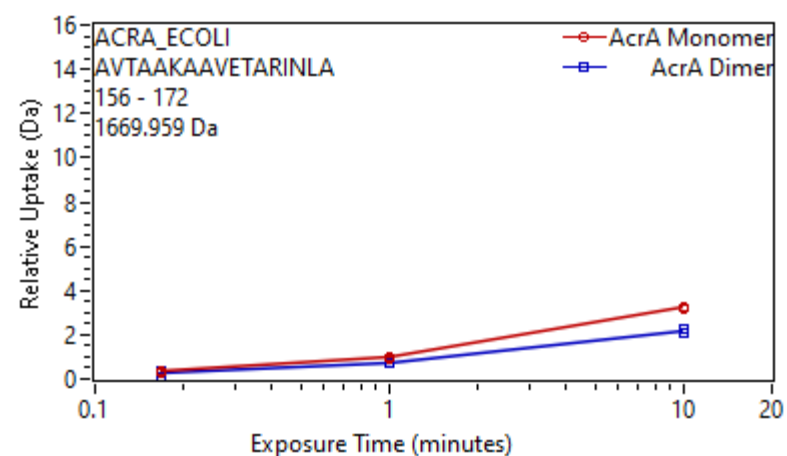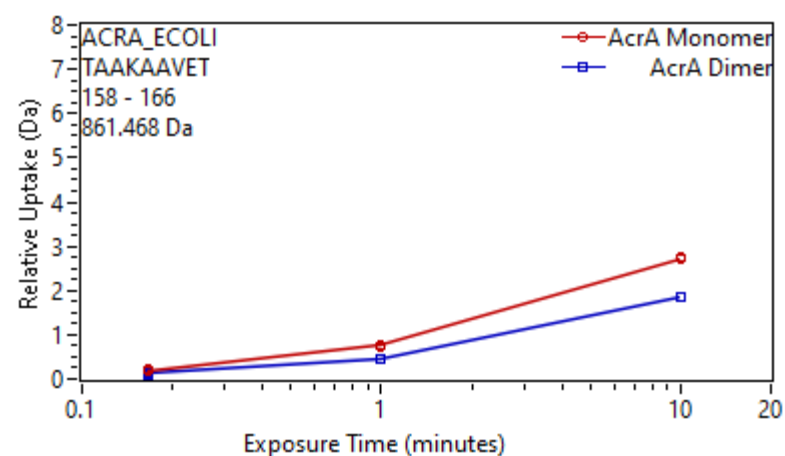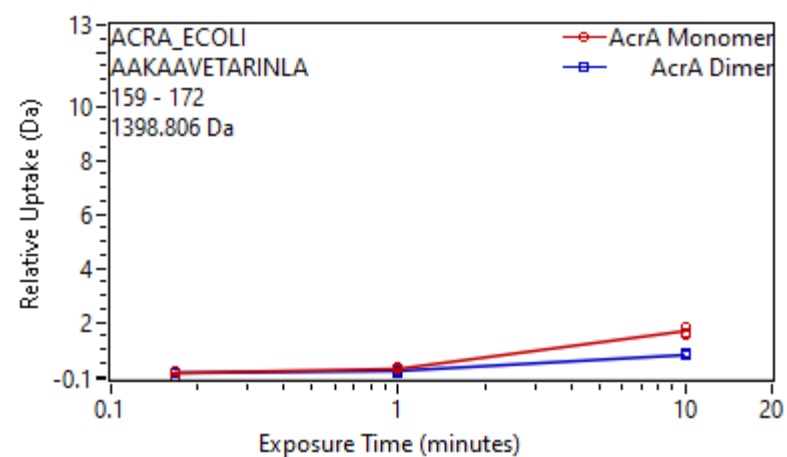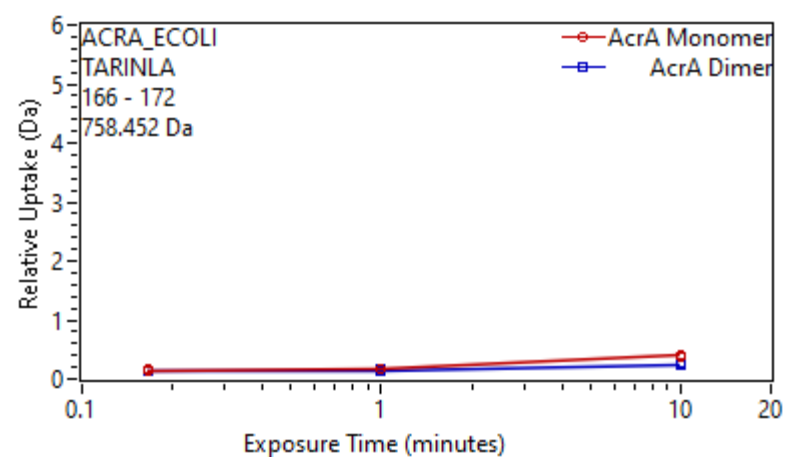

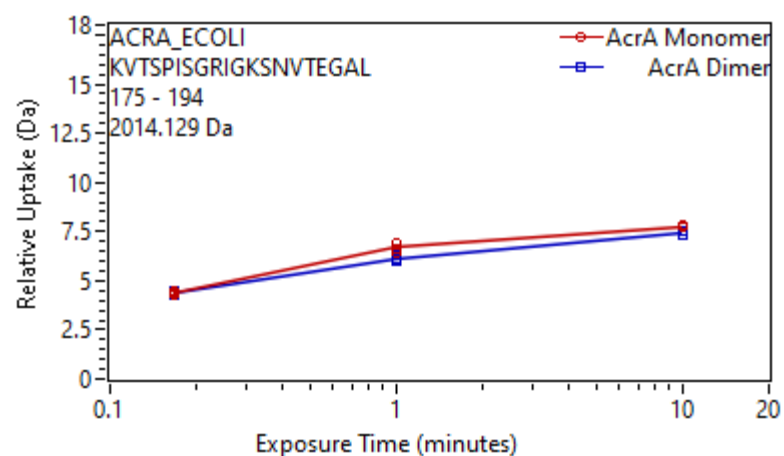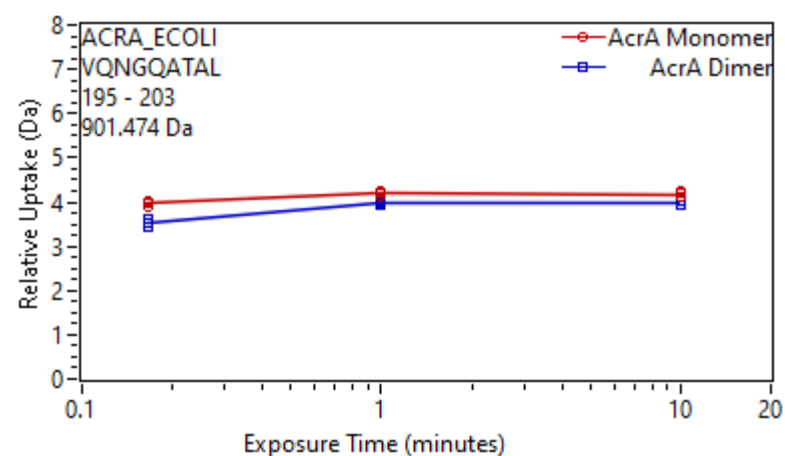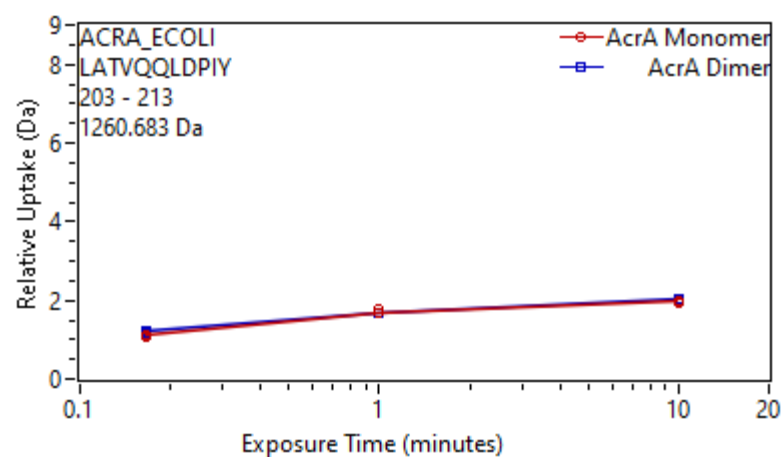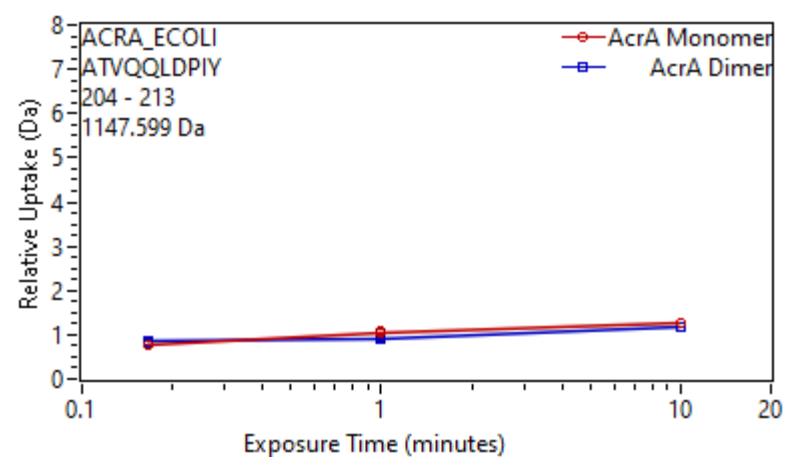

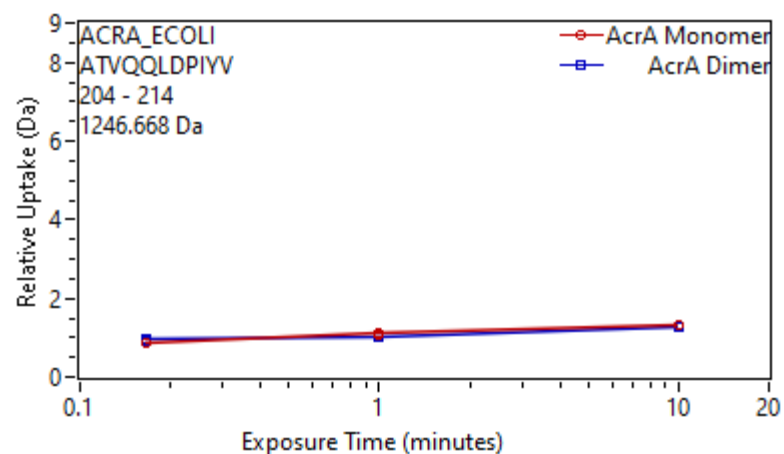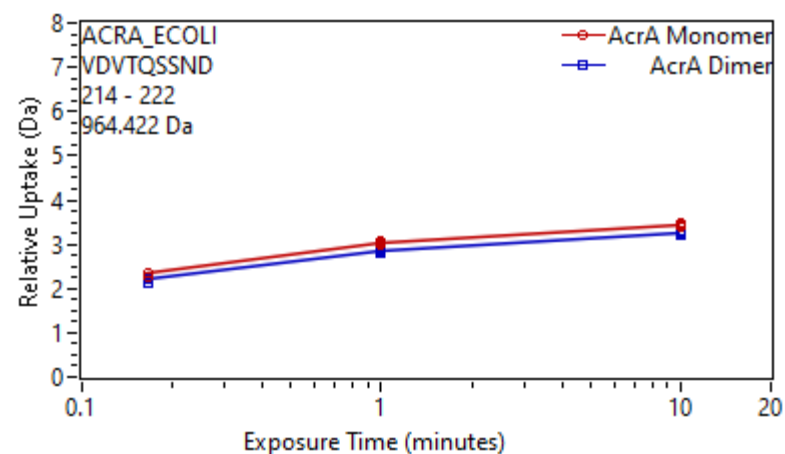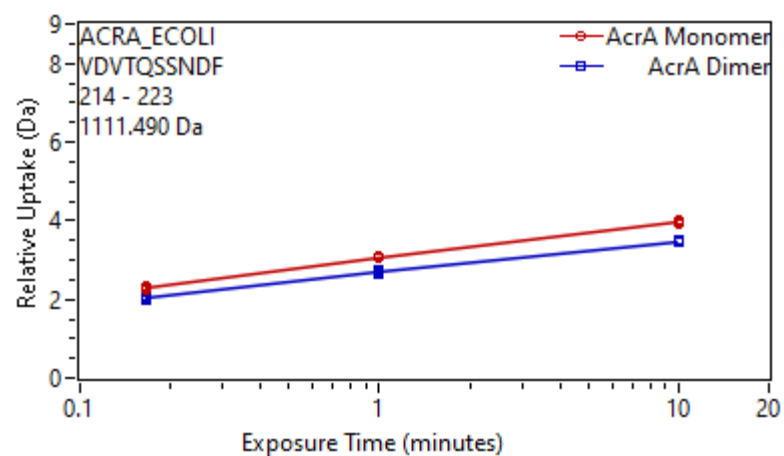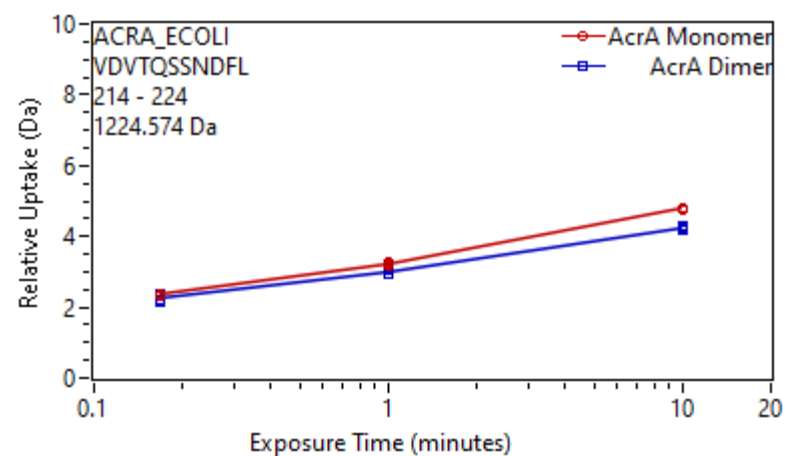

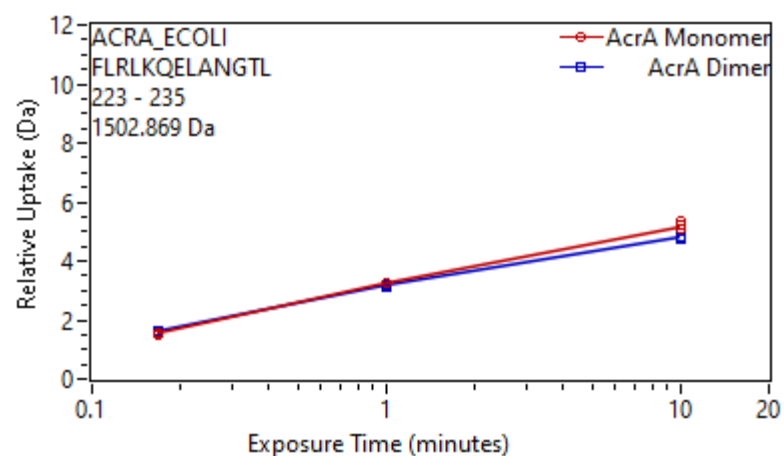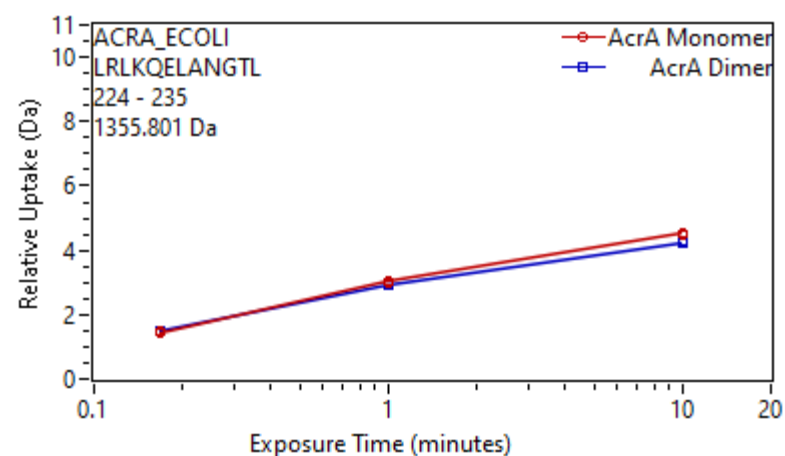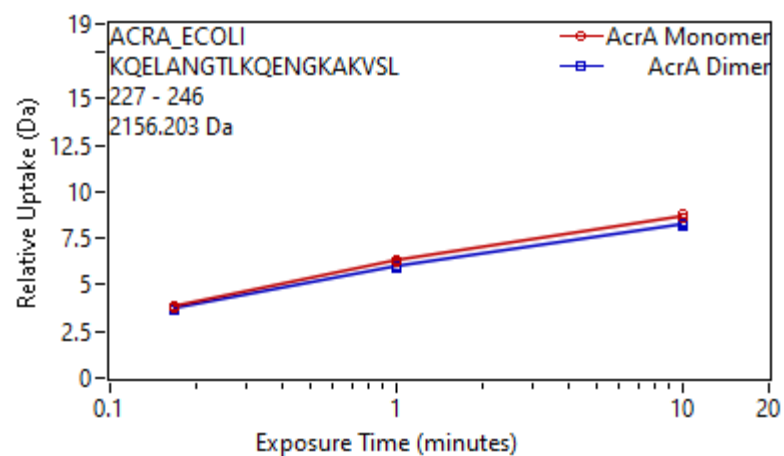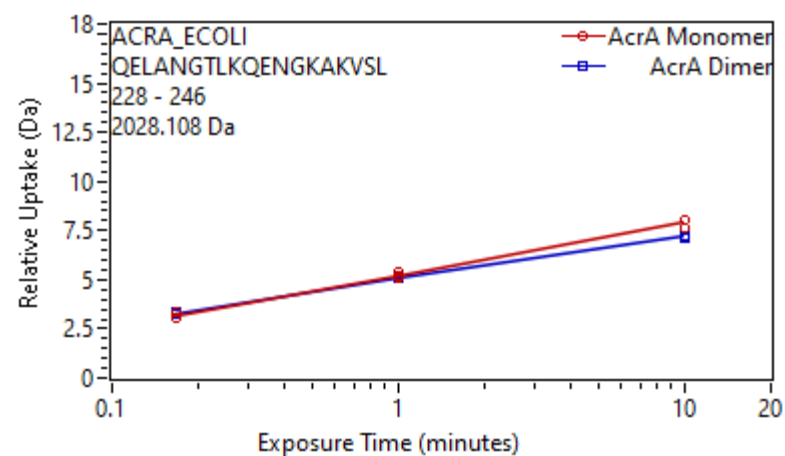

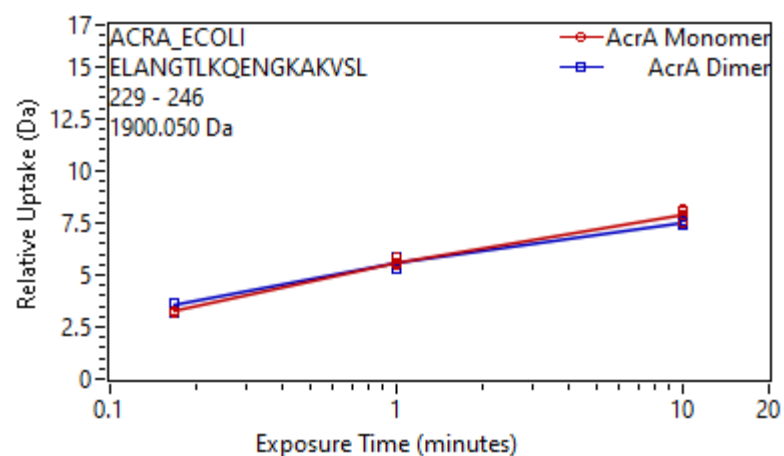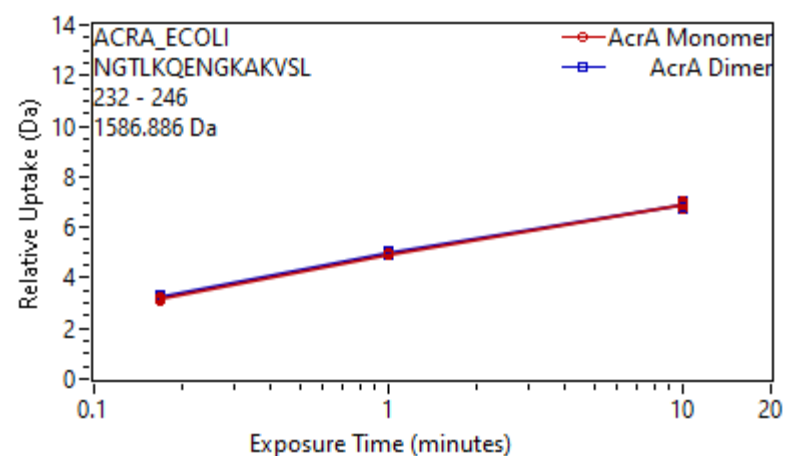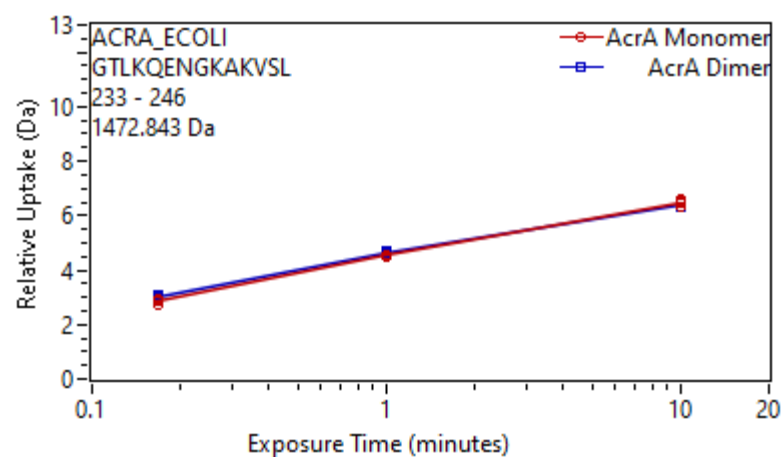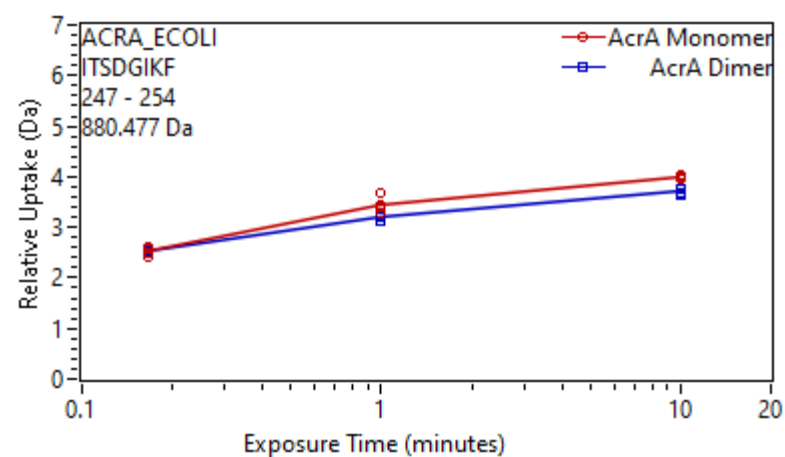

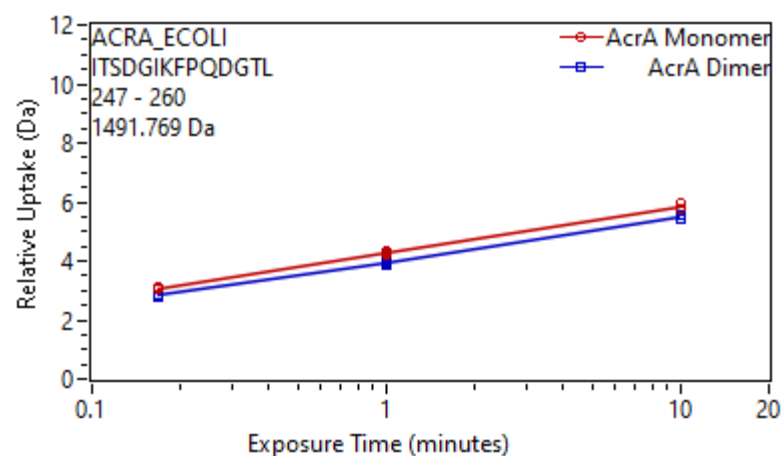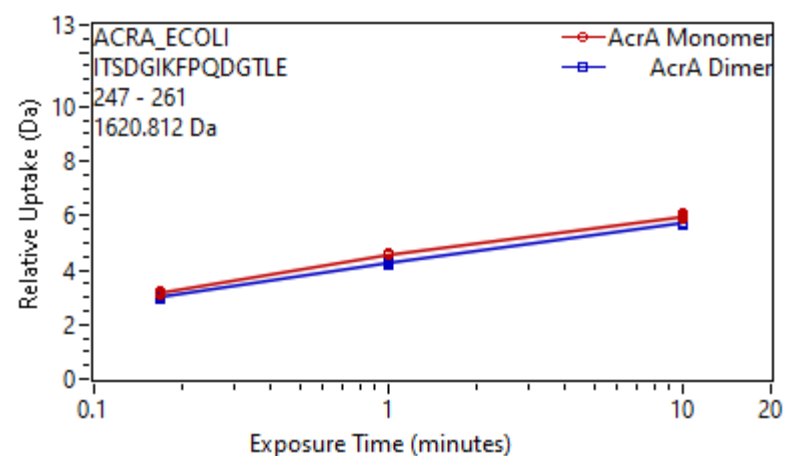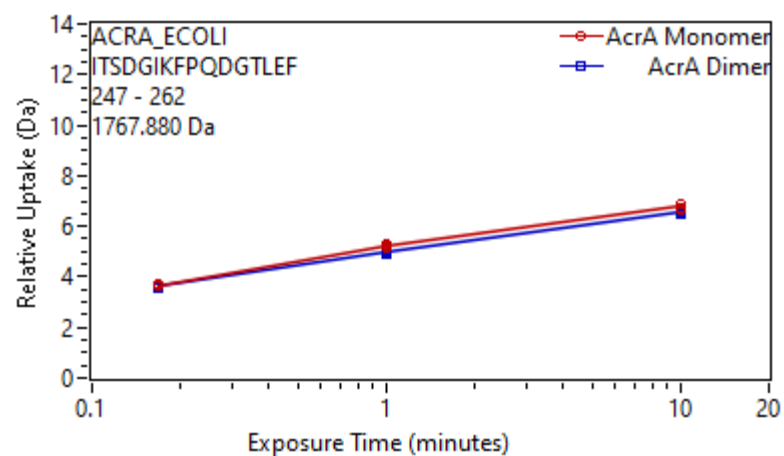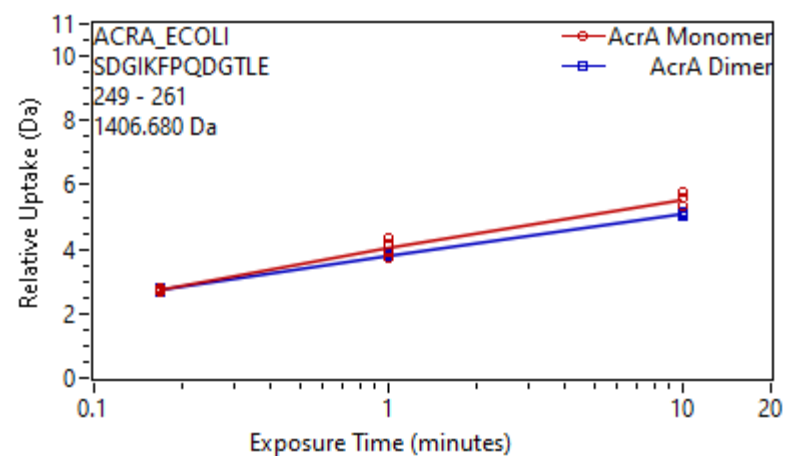

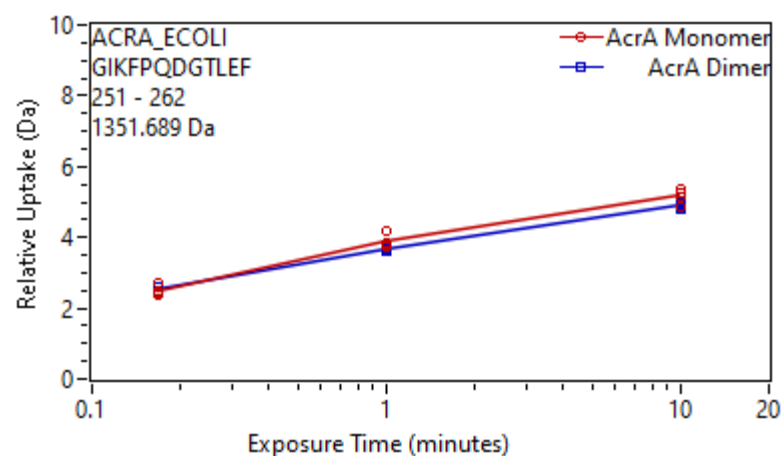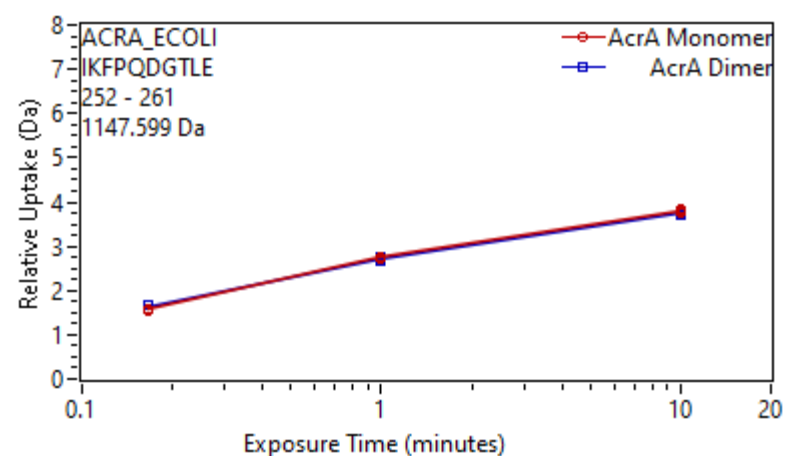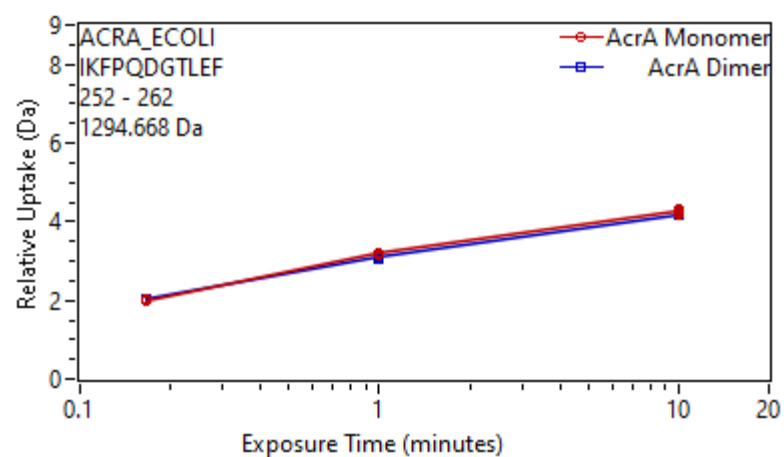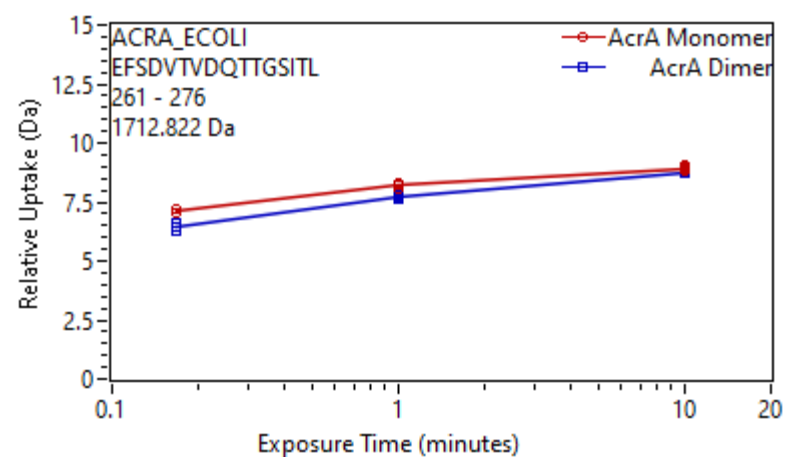

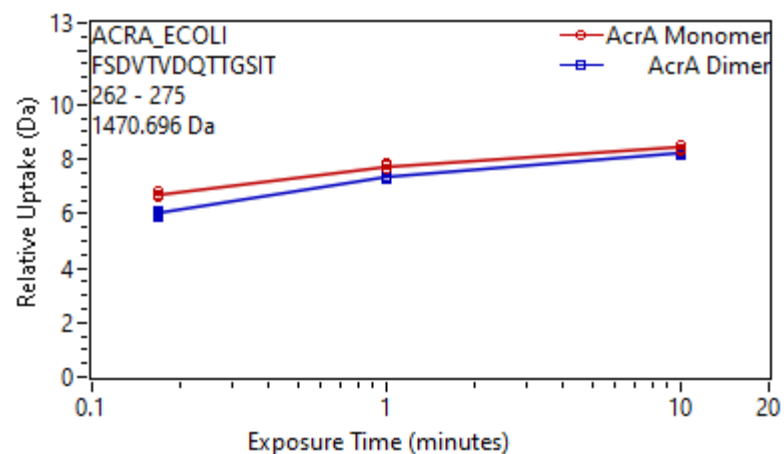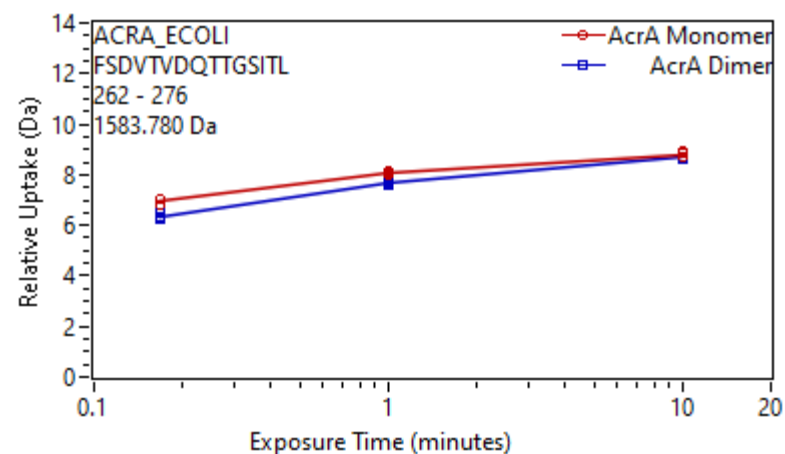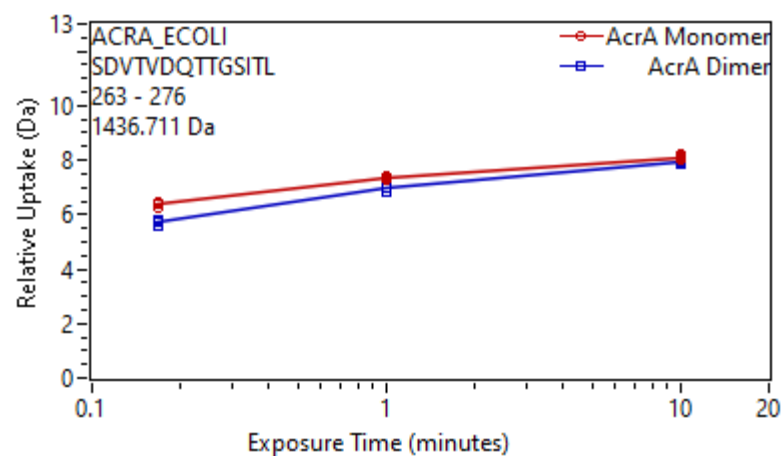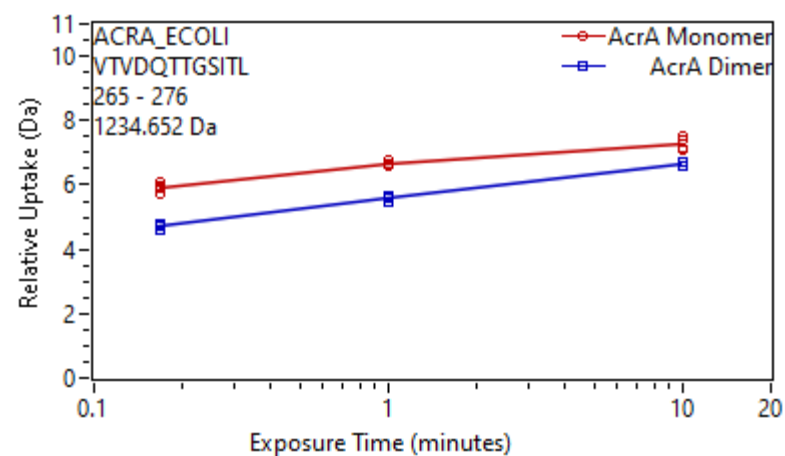

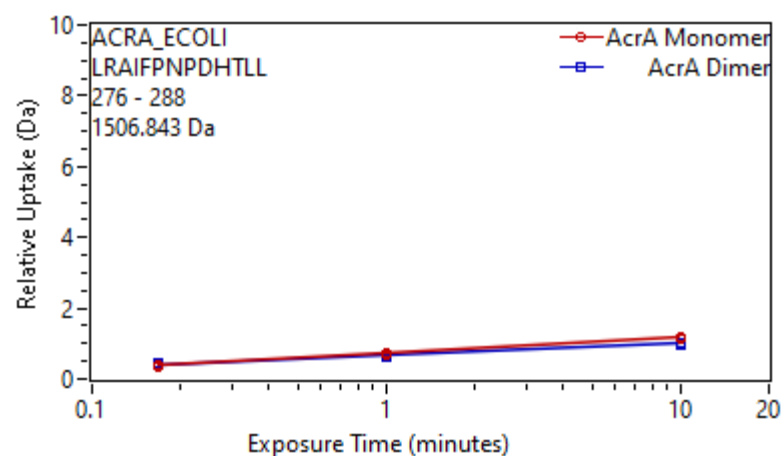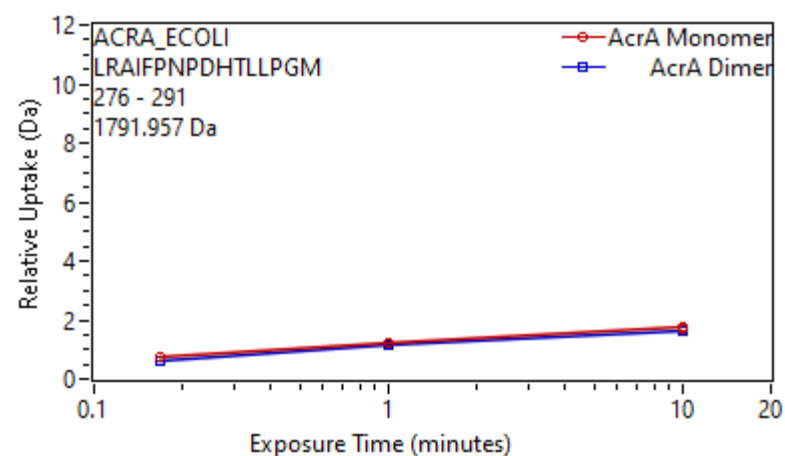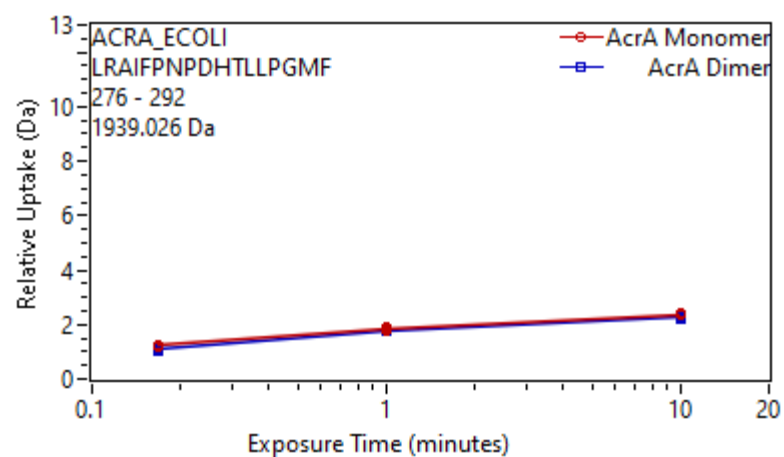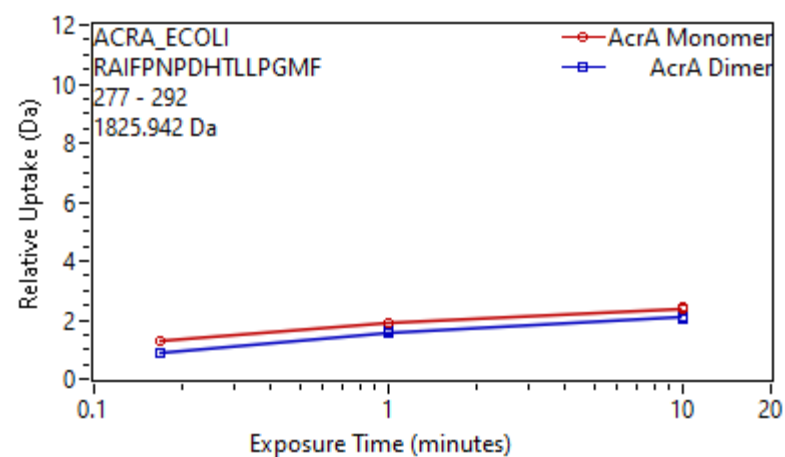

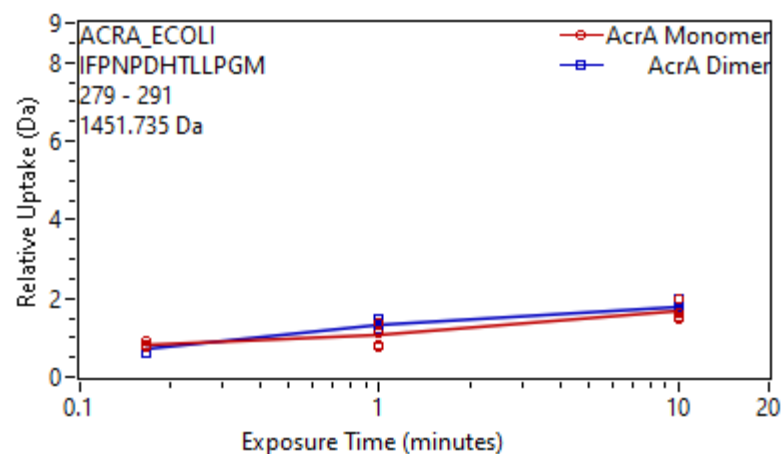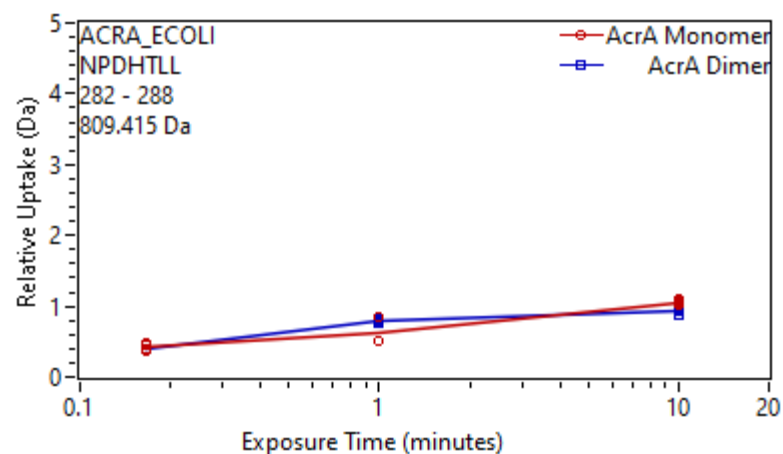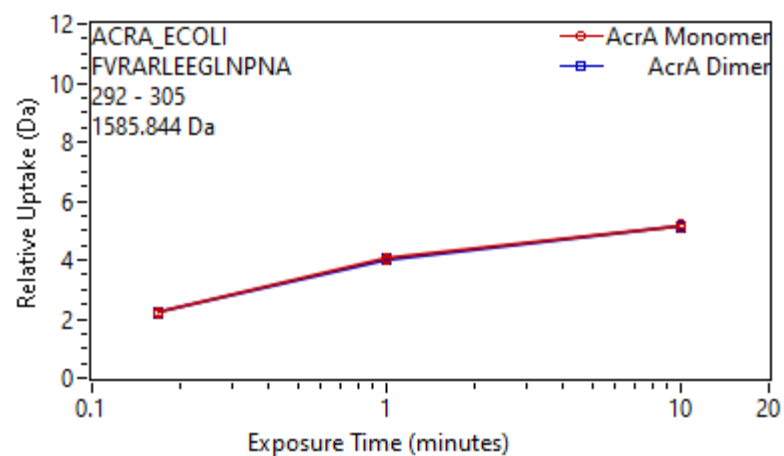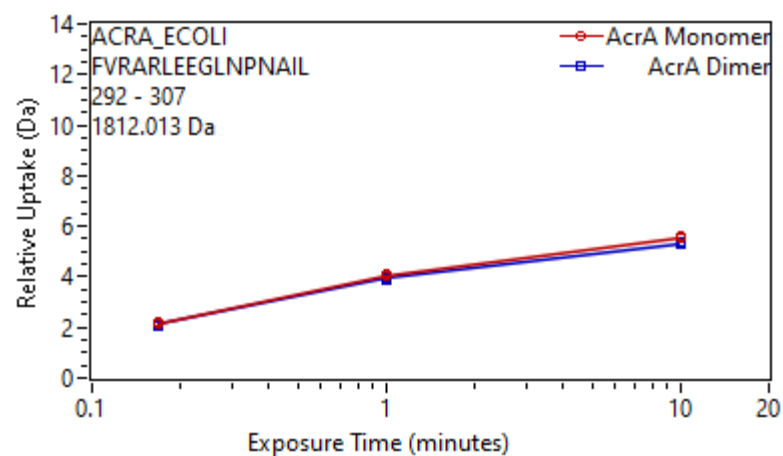

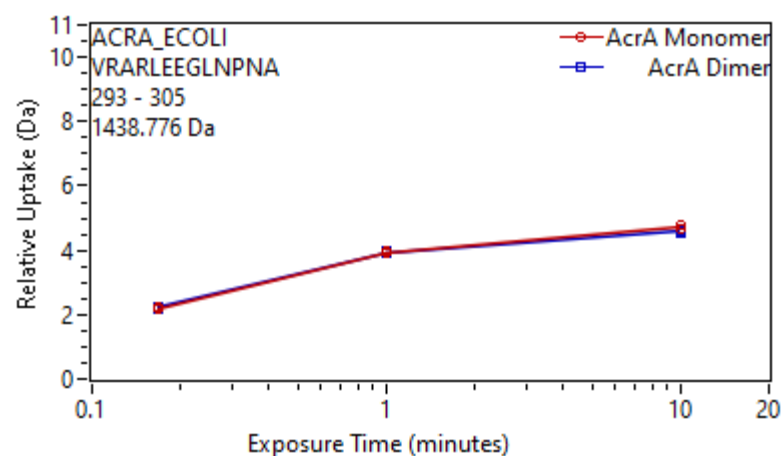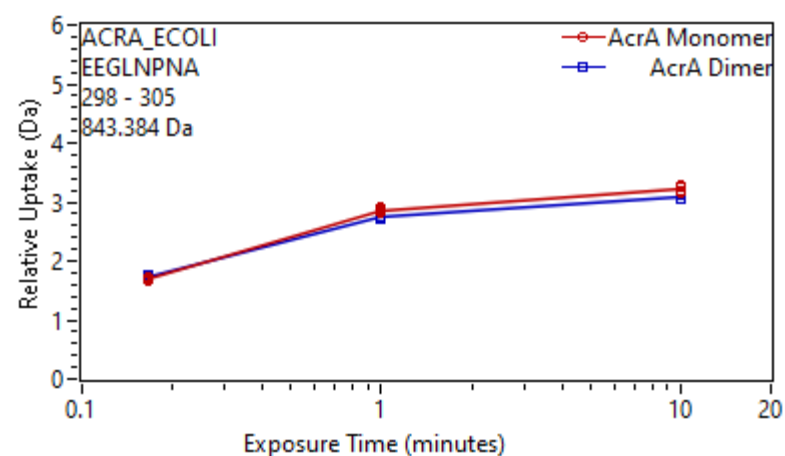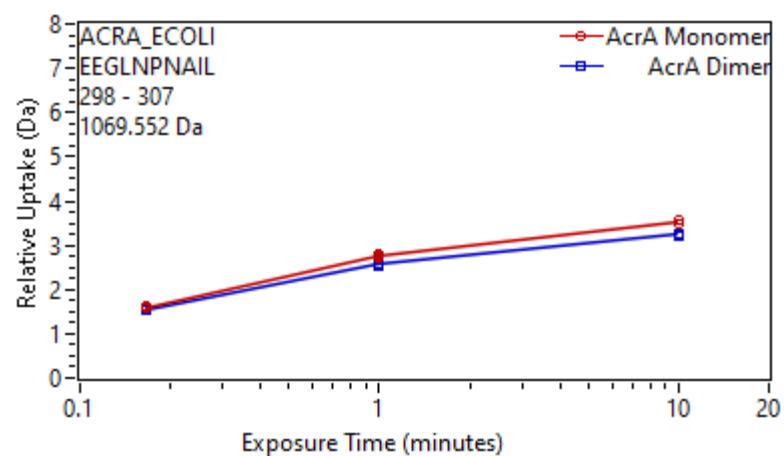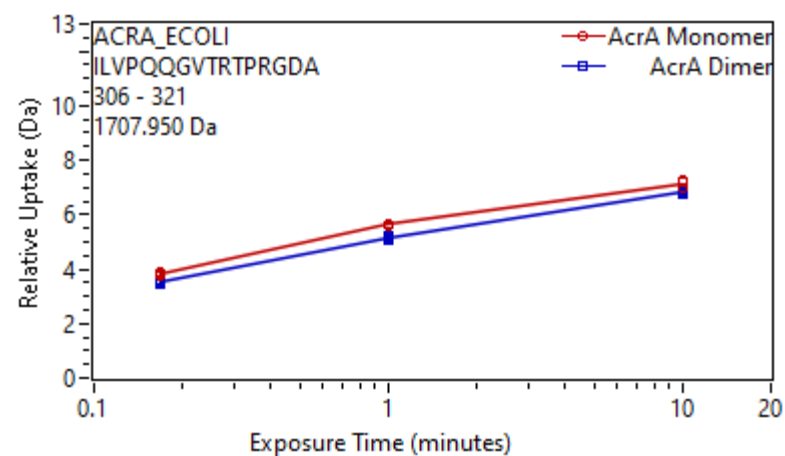

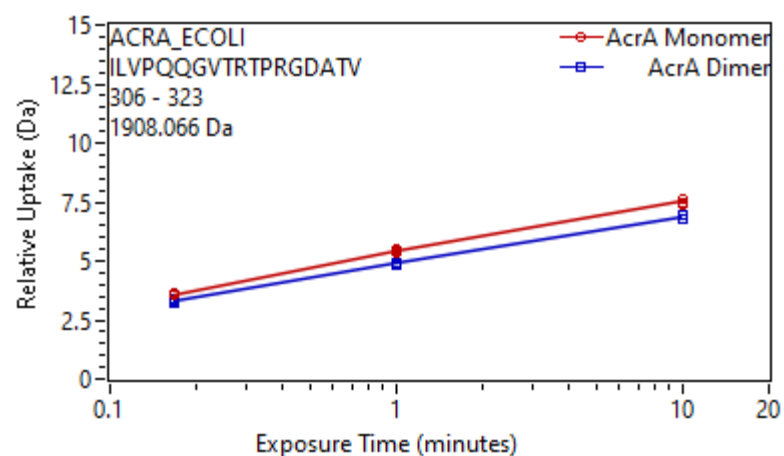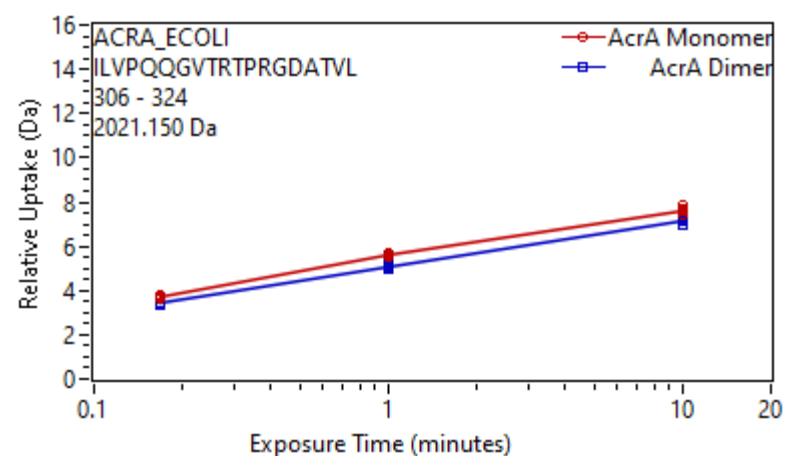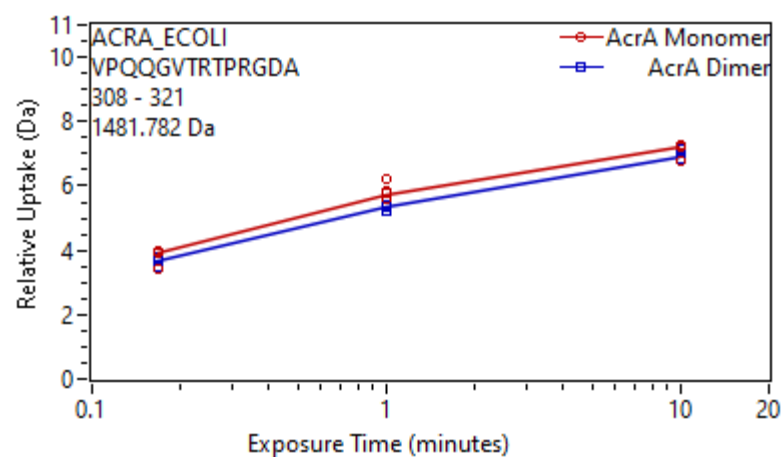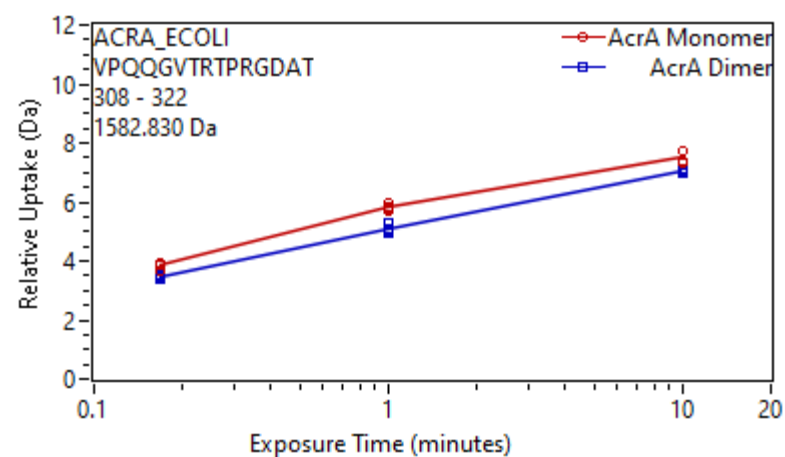

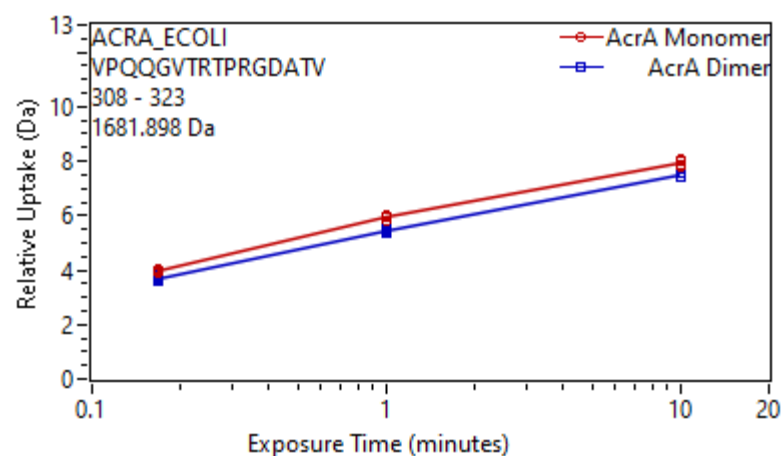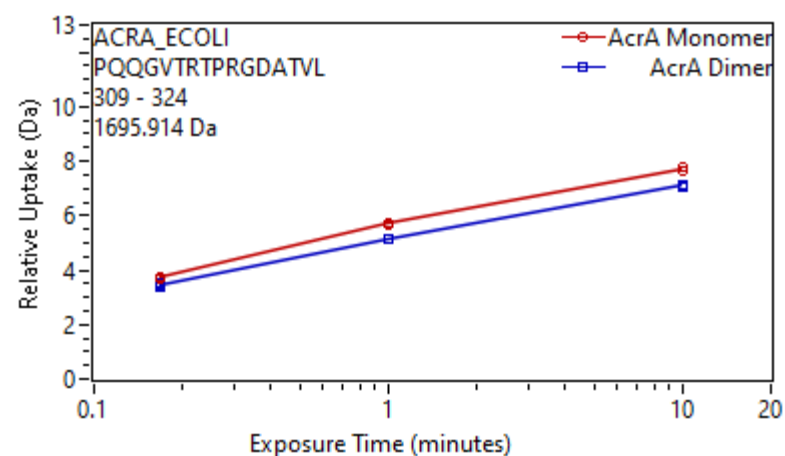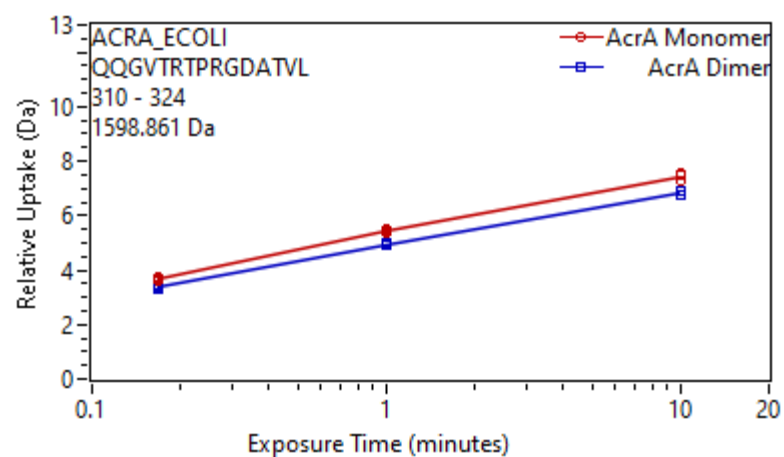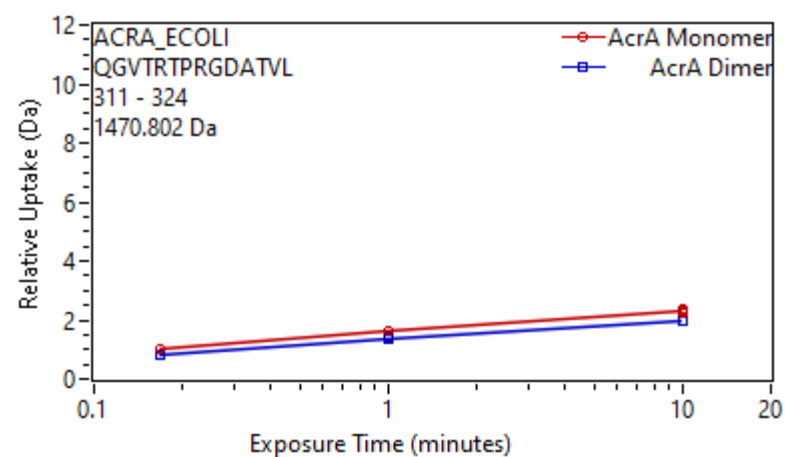

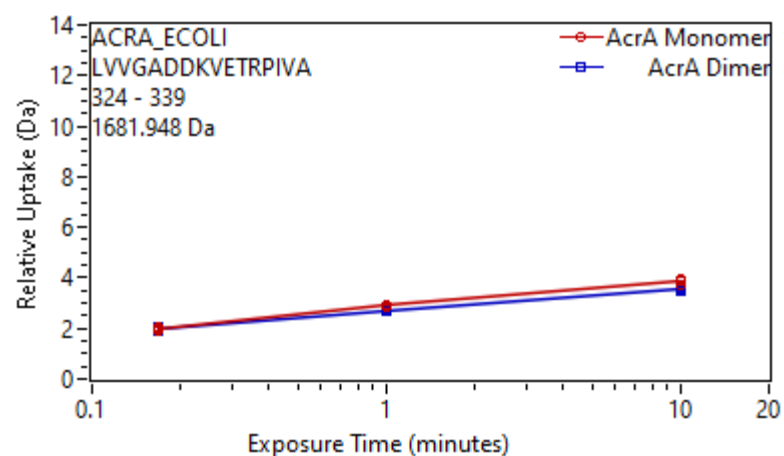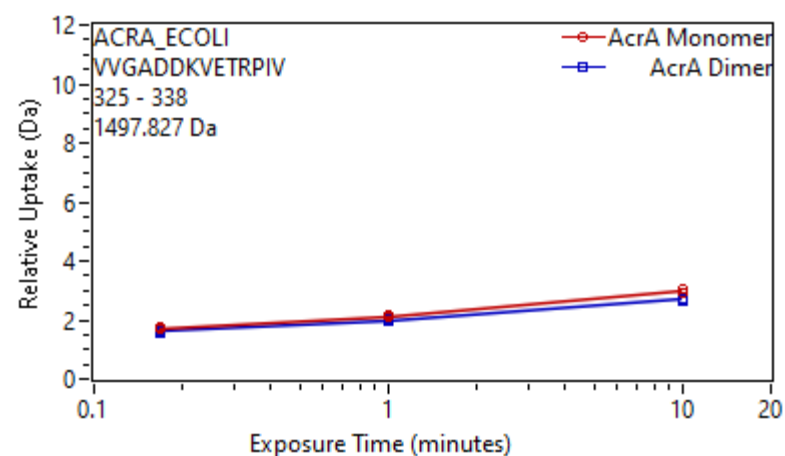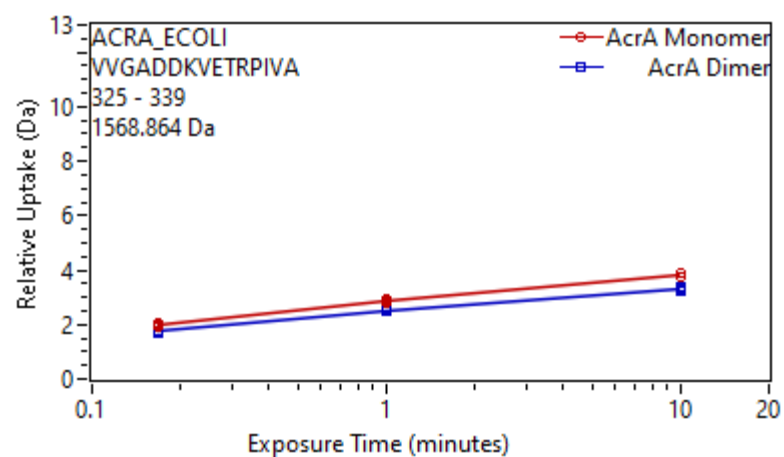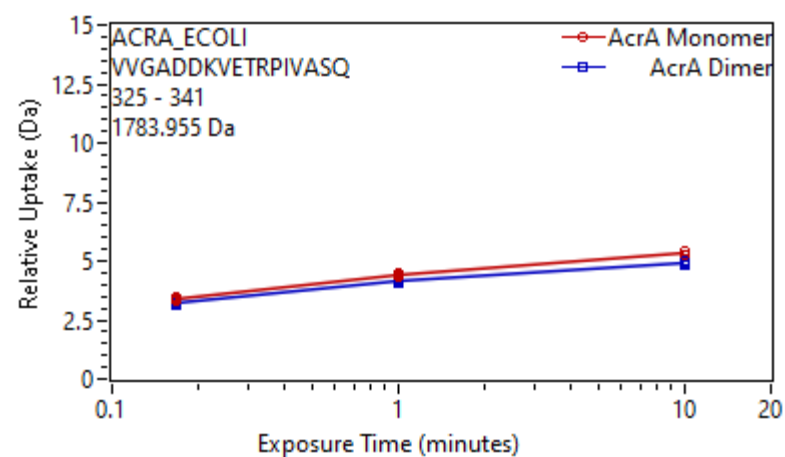

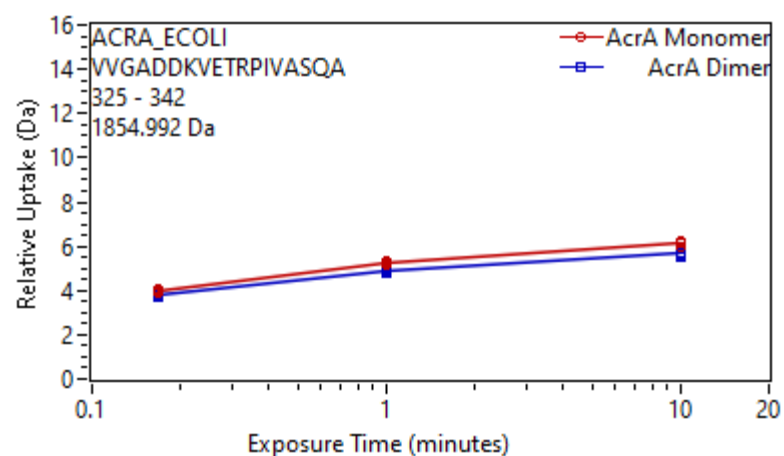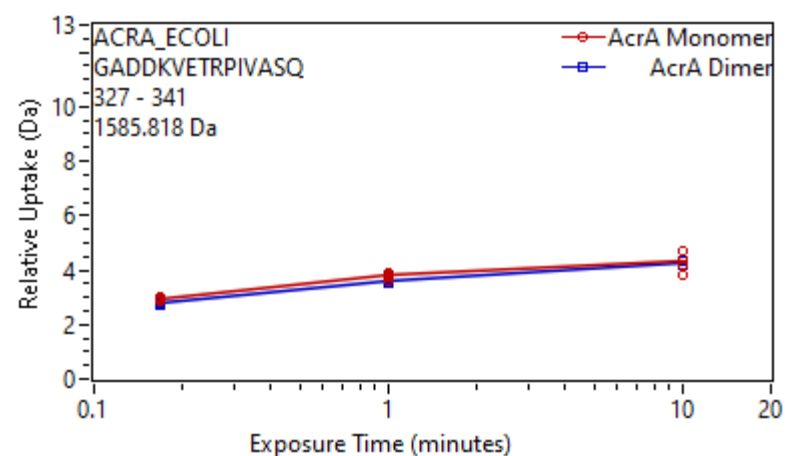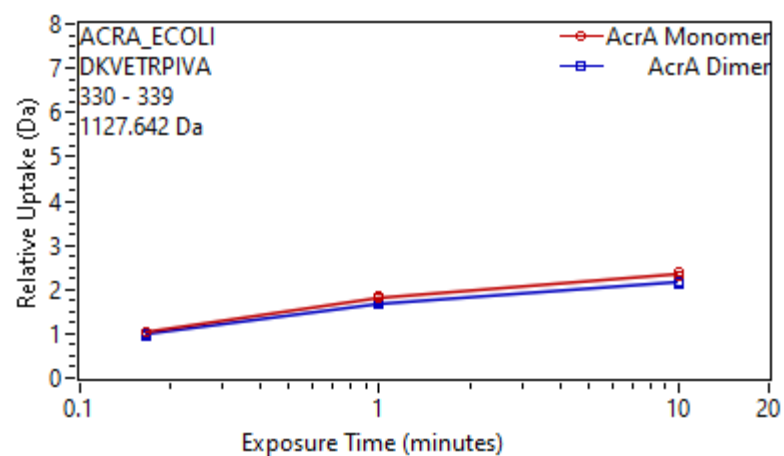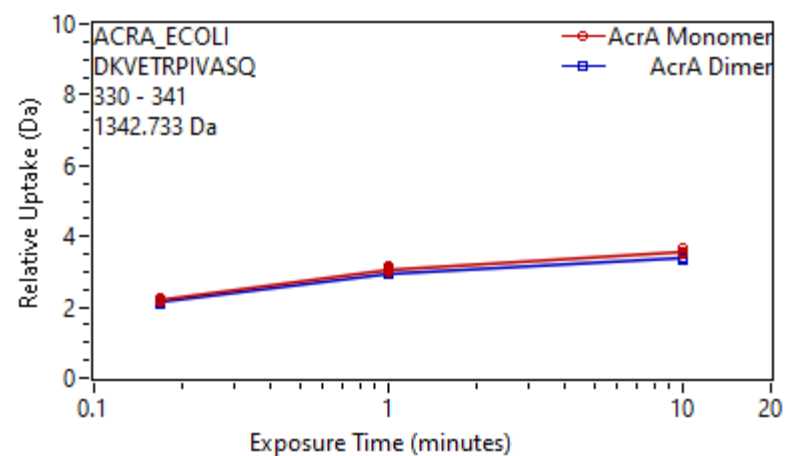

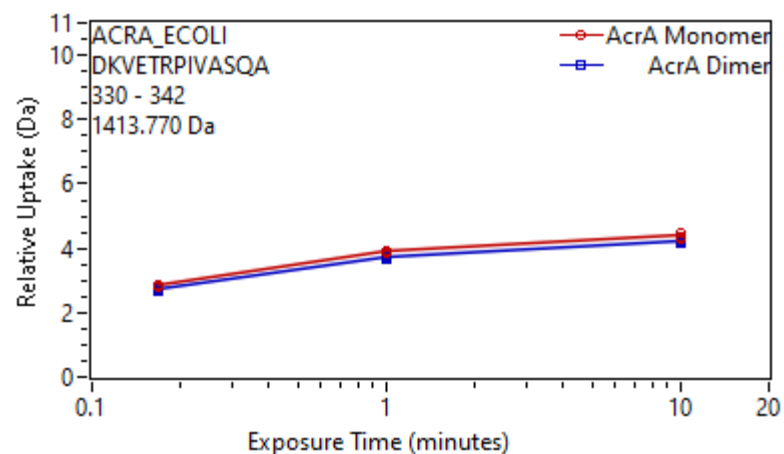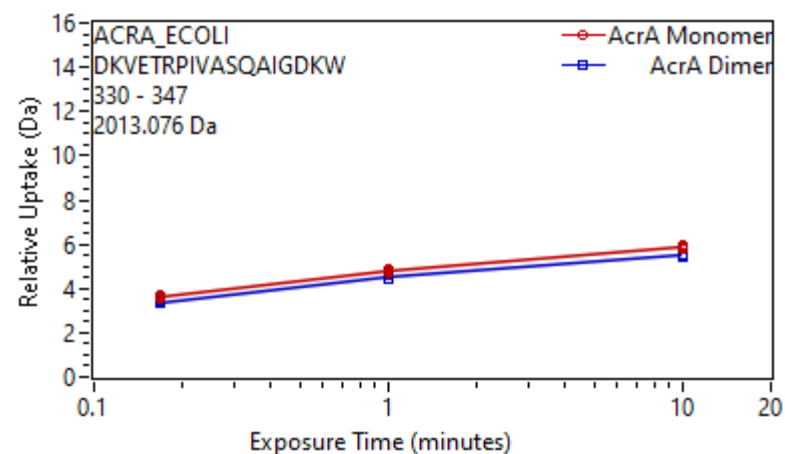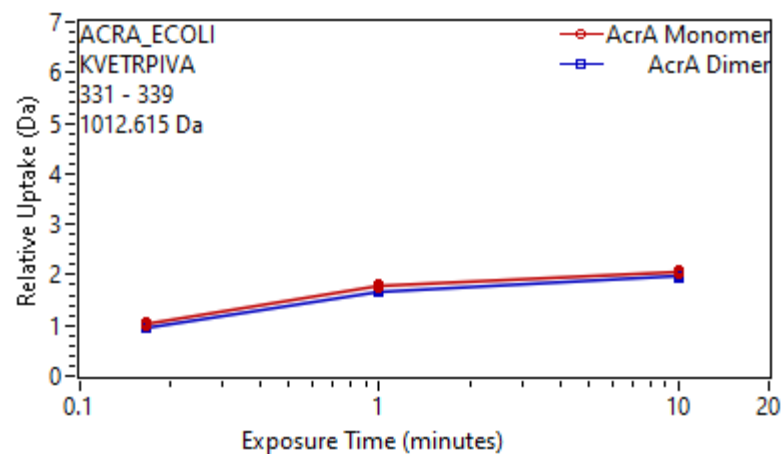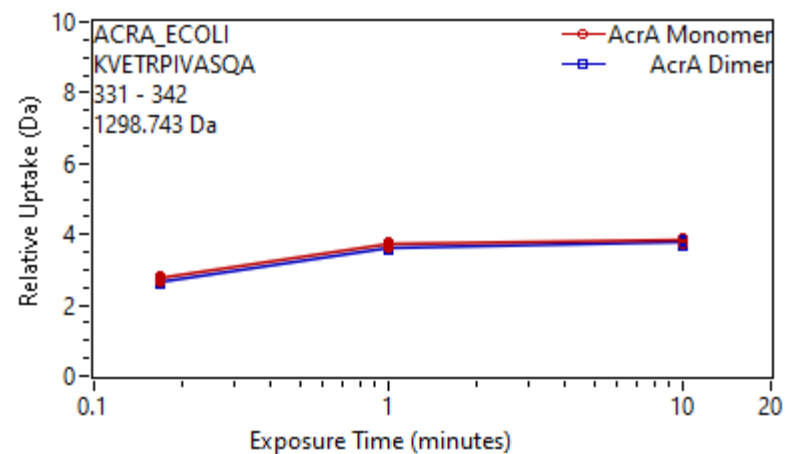

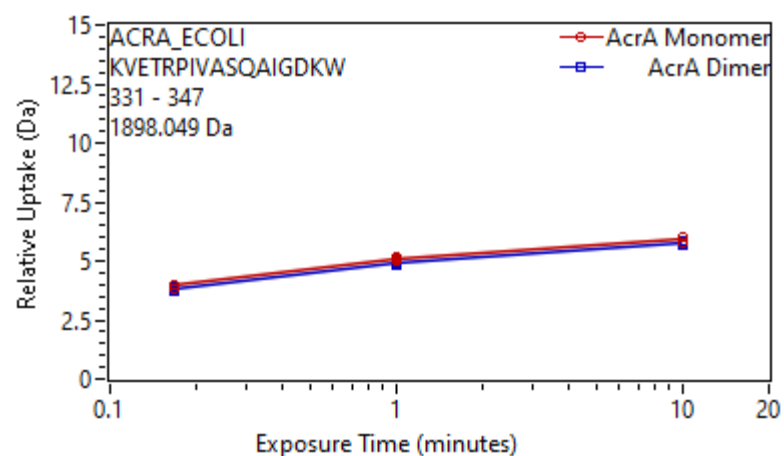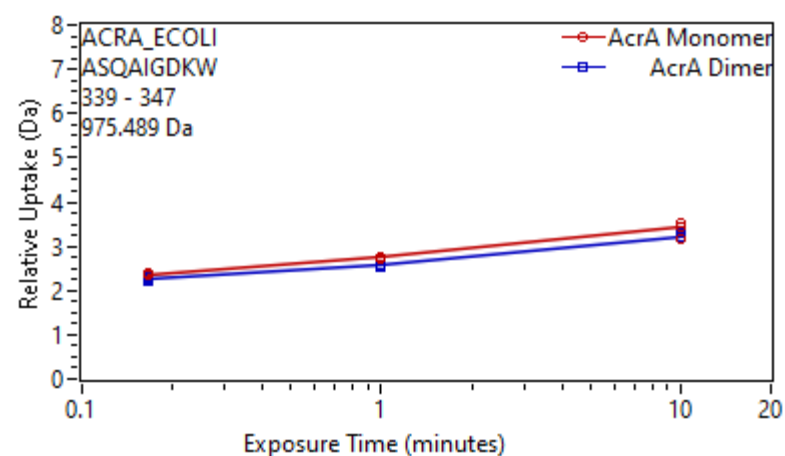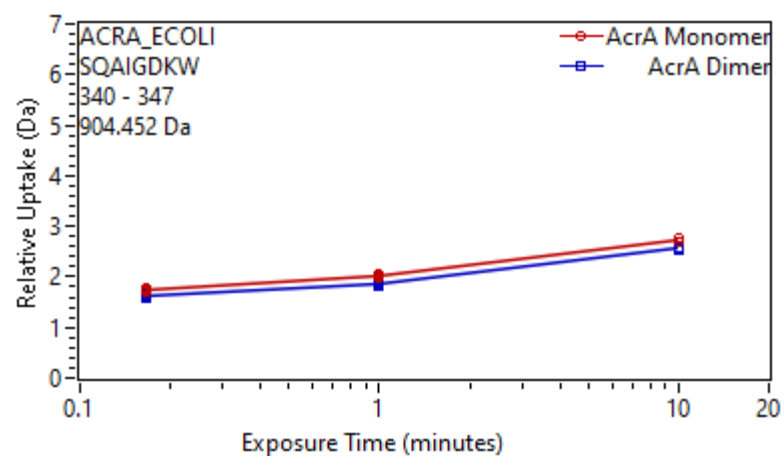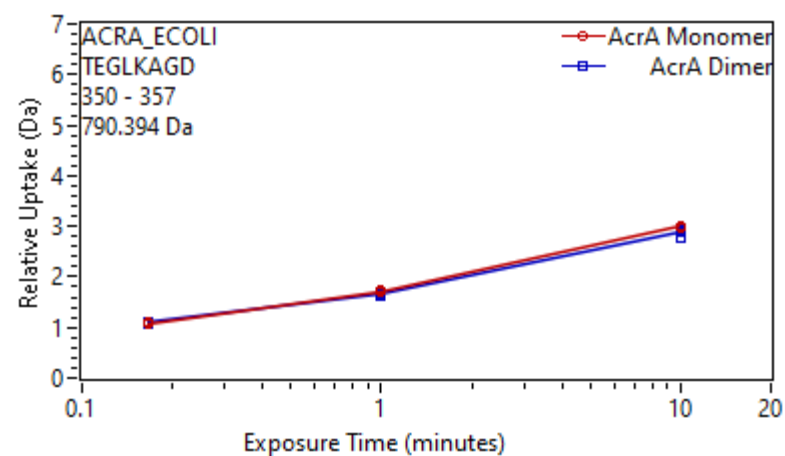

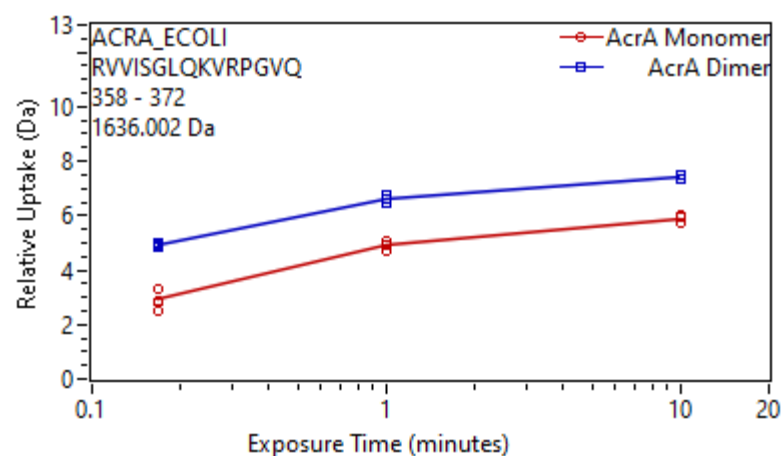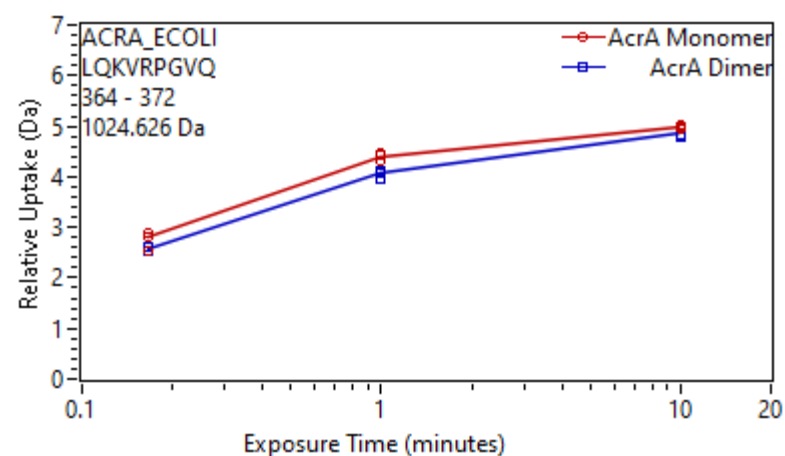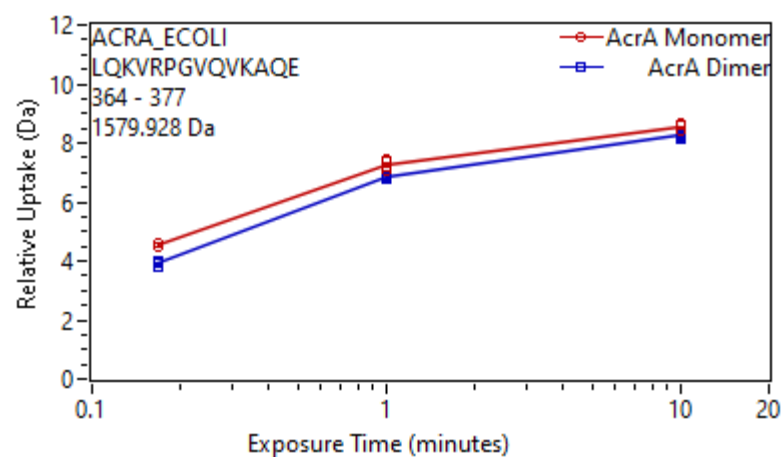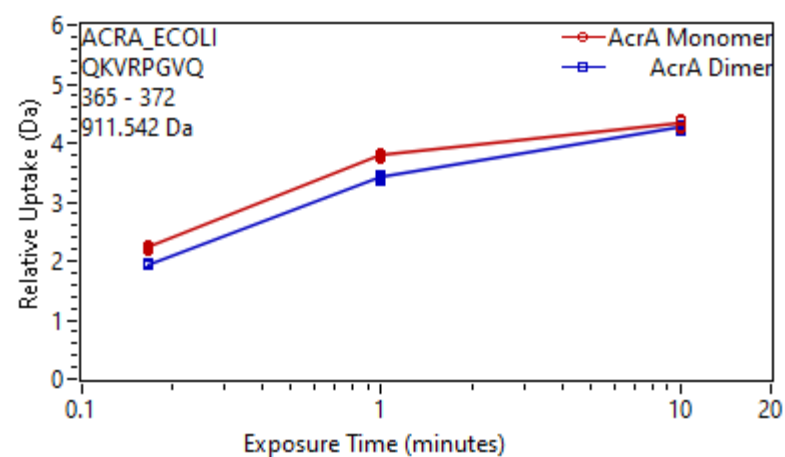

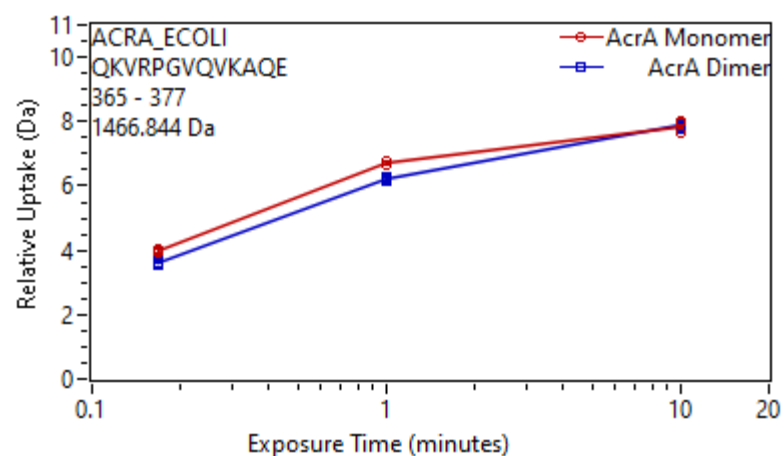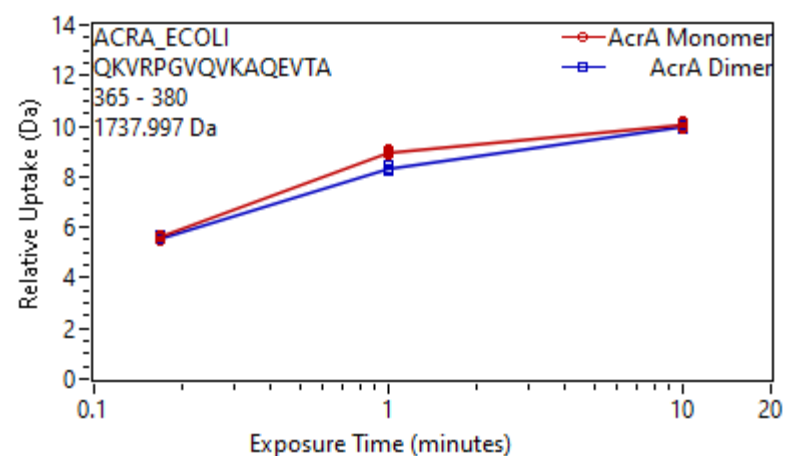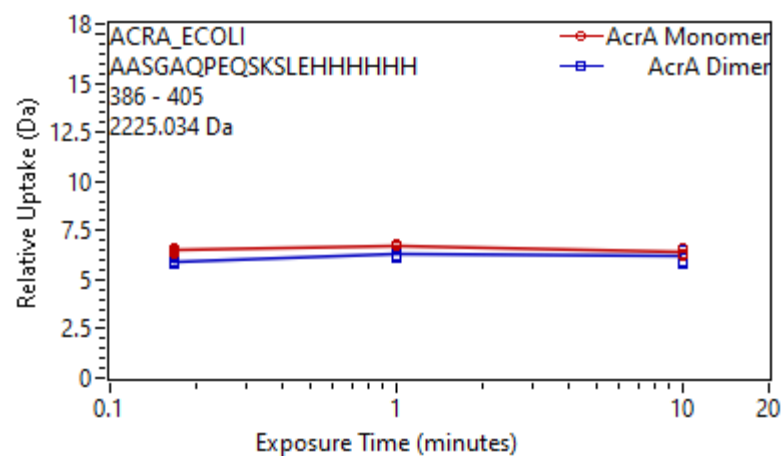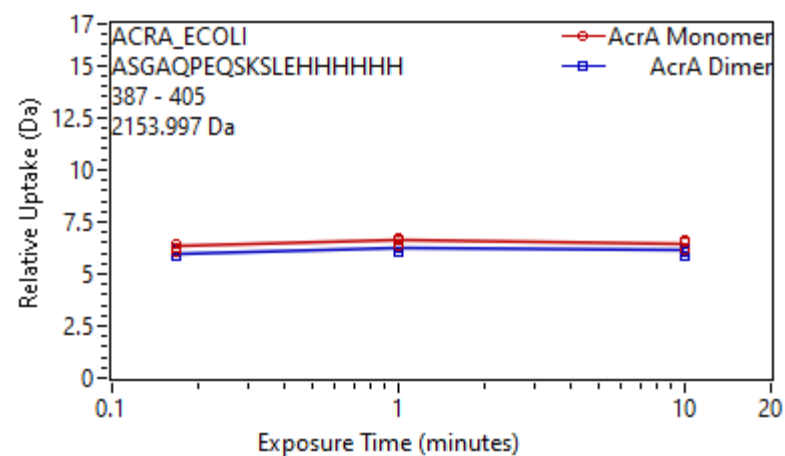

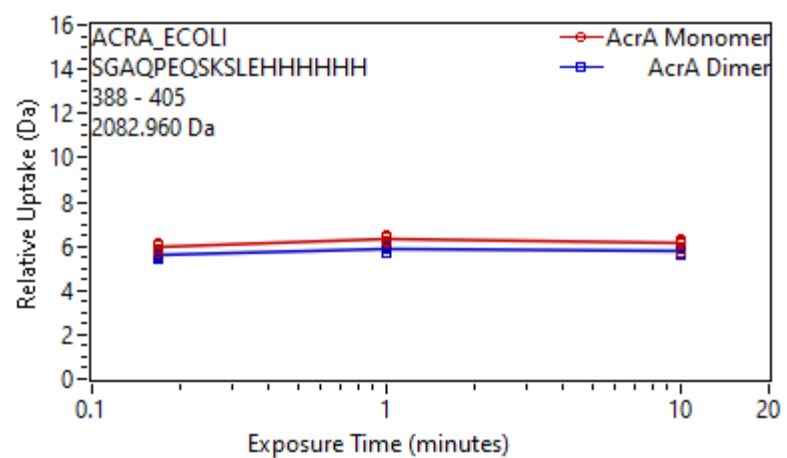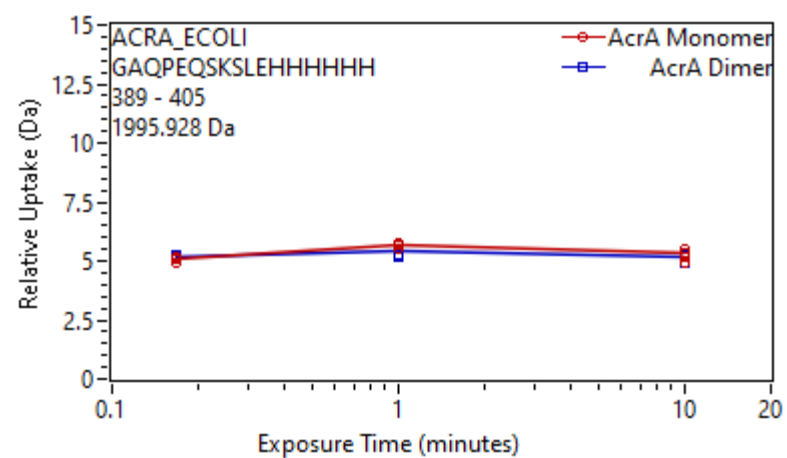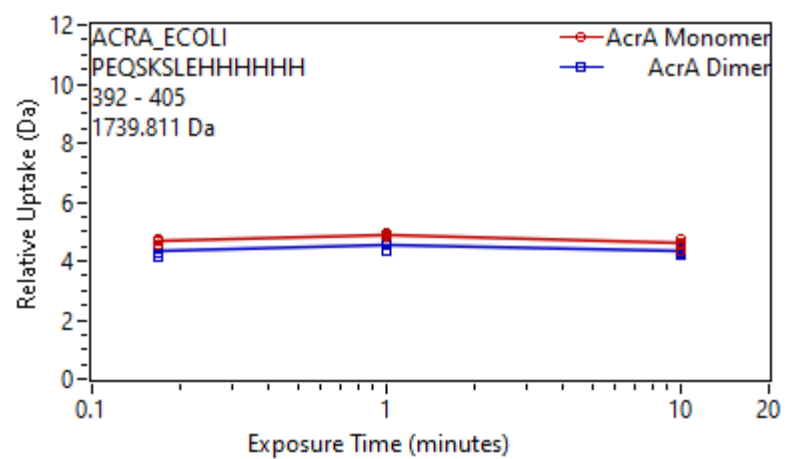

Supplement: Supplementary file 6 — Supplementary Data 3 [file 41467_2023_39615_MOESM6_ESM.zip › Supplementary Data 3/AcrAs_AcrApd_DMSO_UptakePlots.pdf]

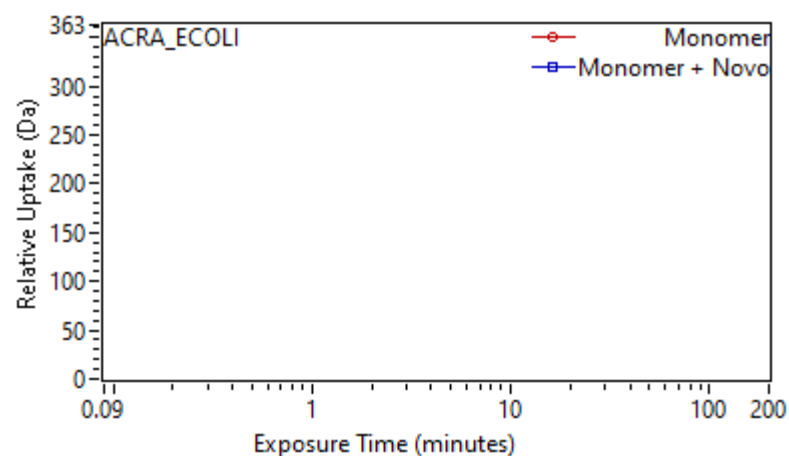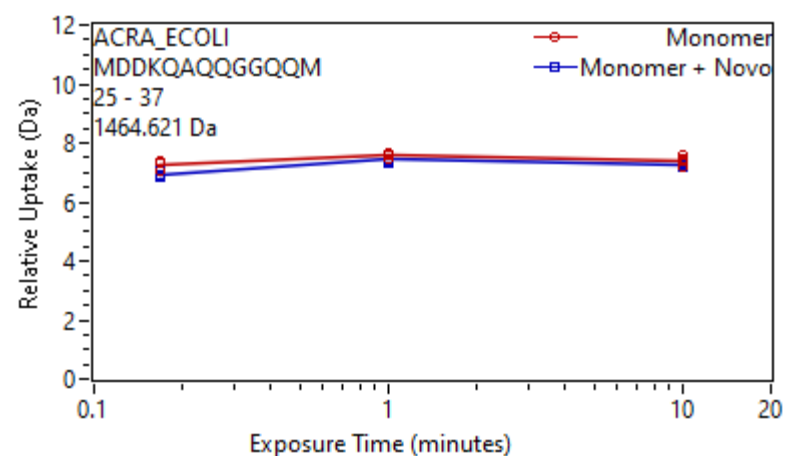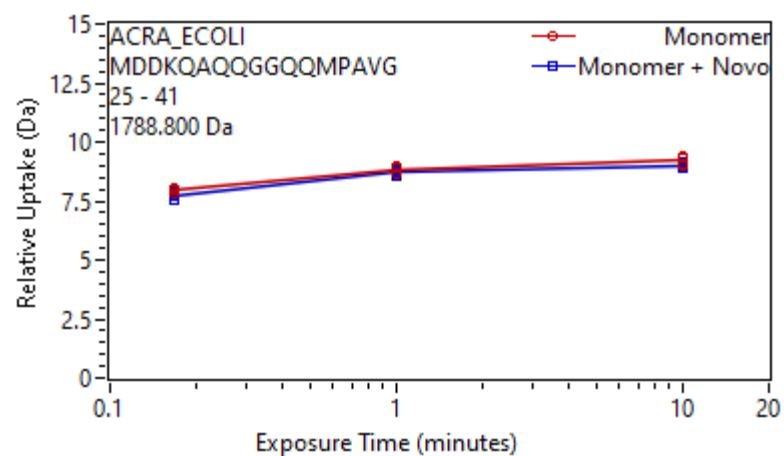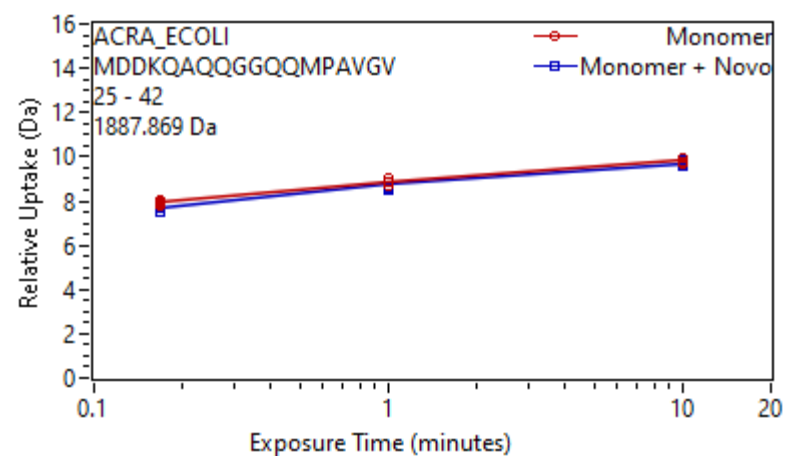

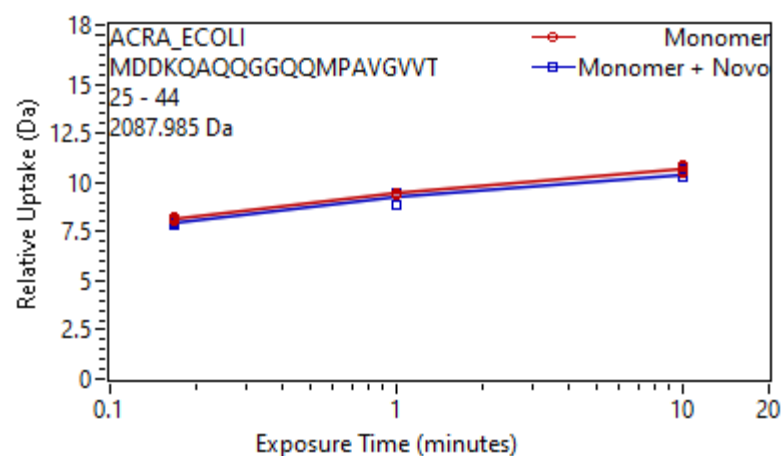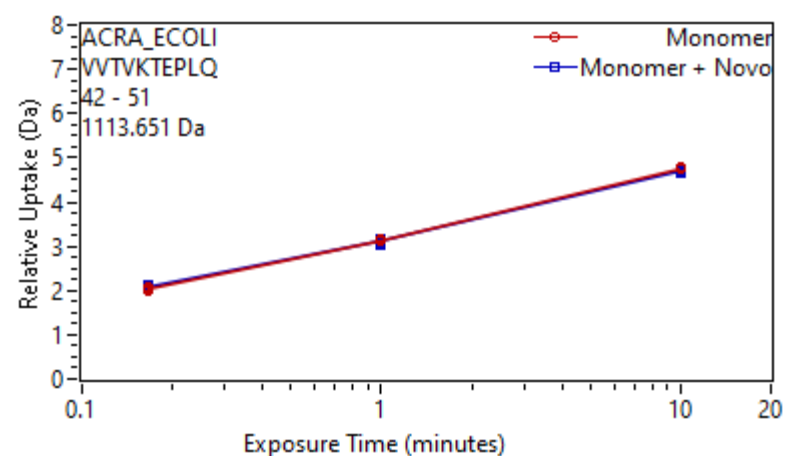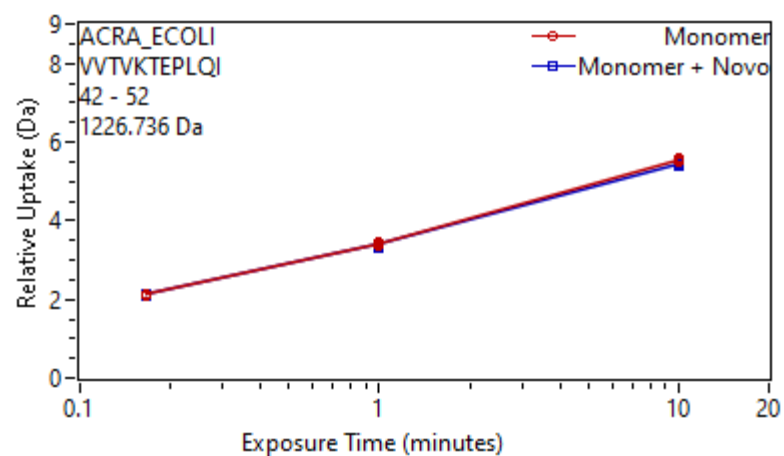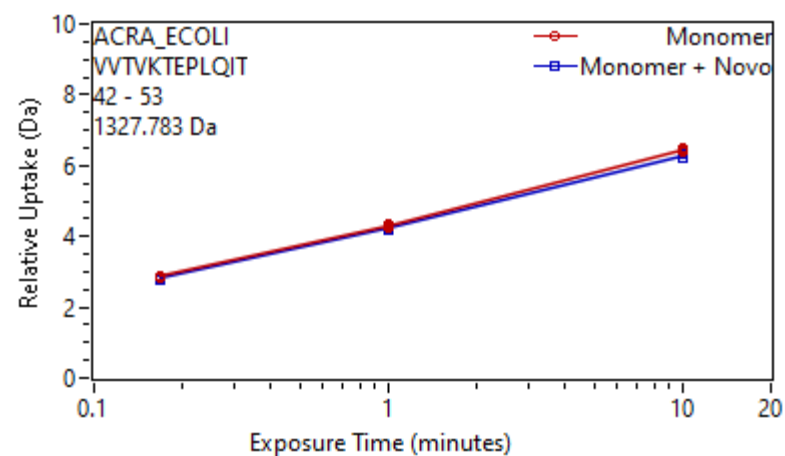

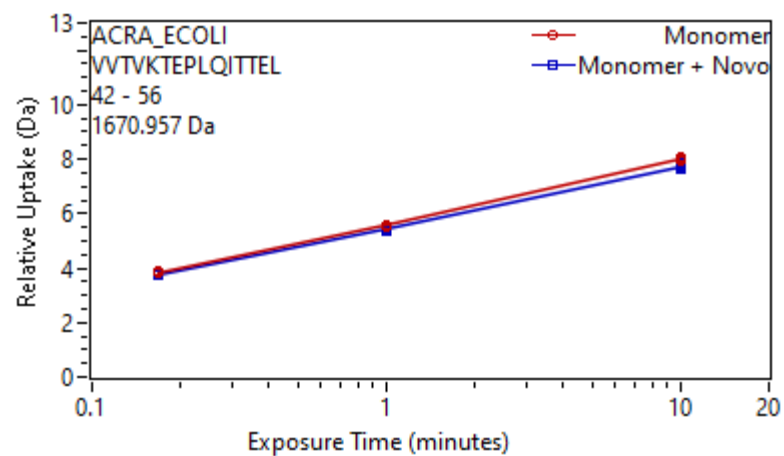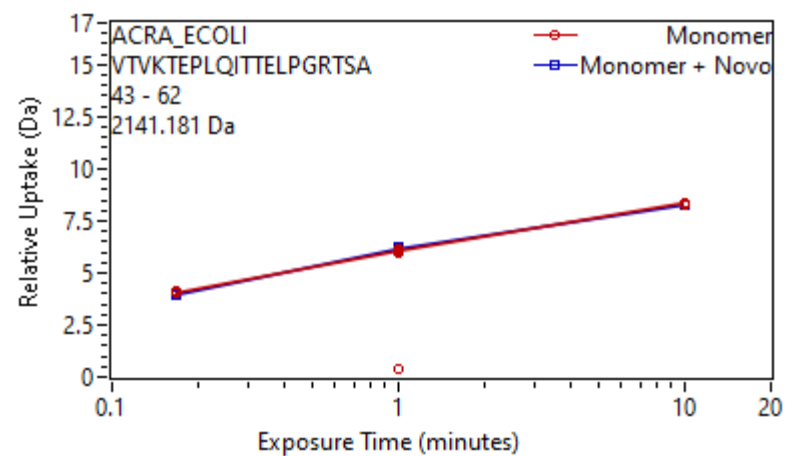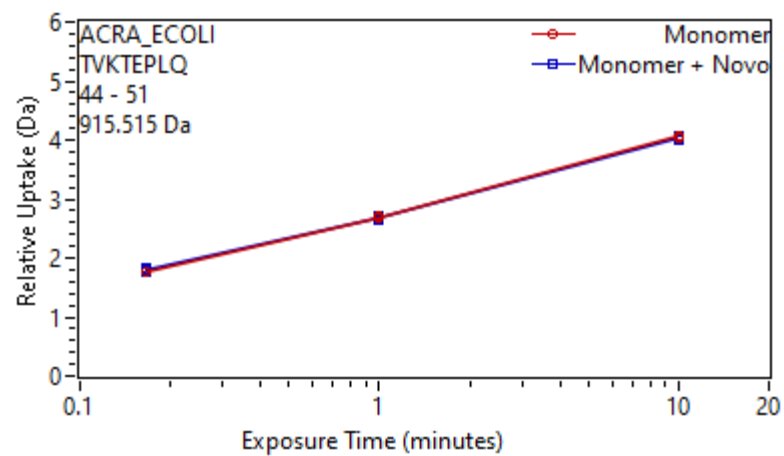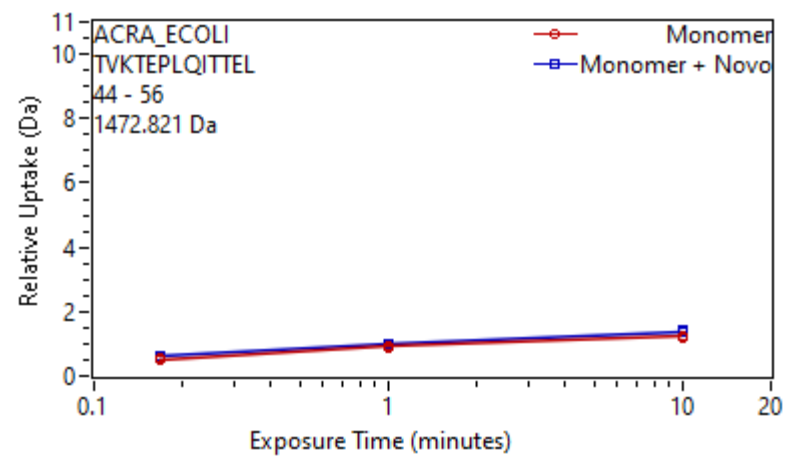

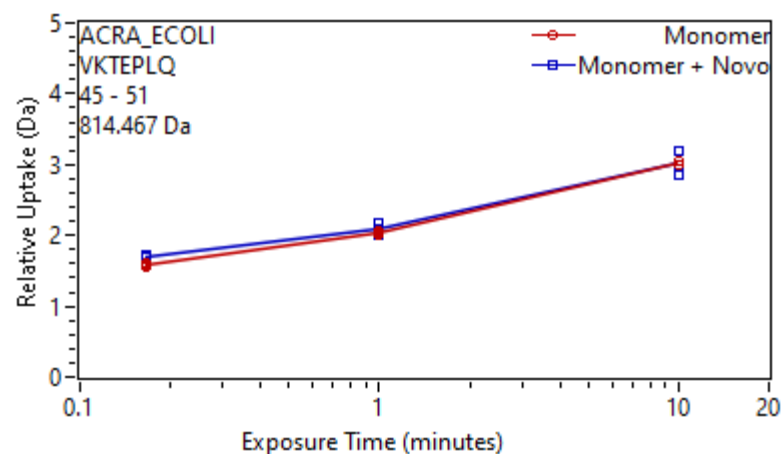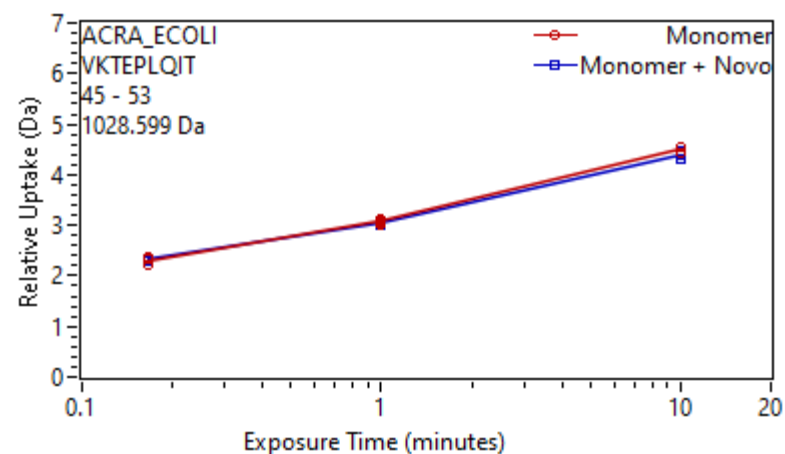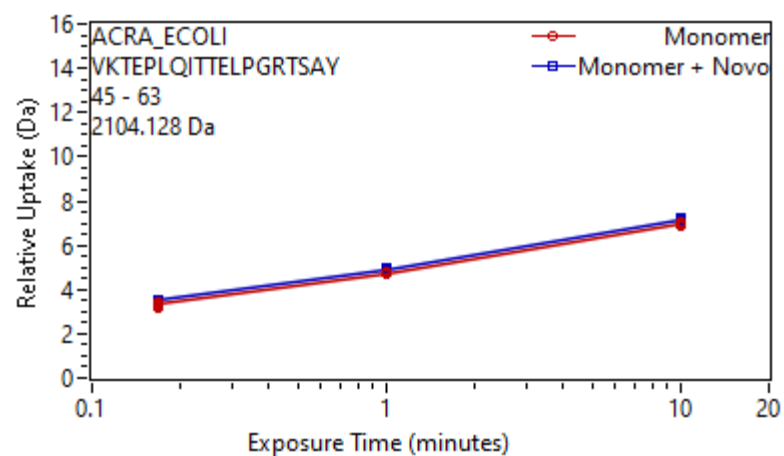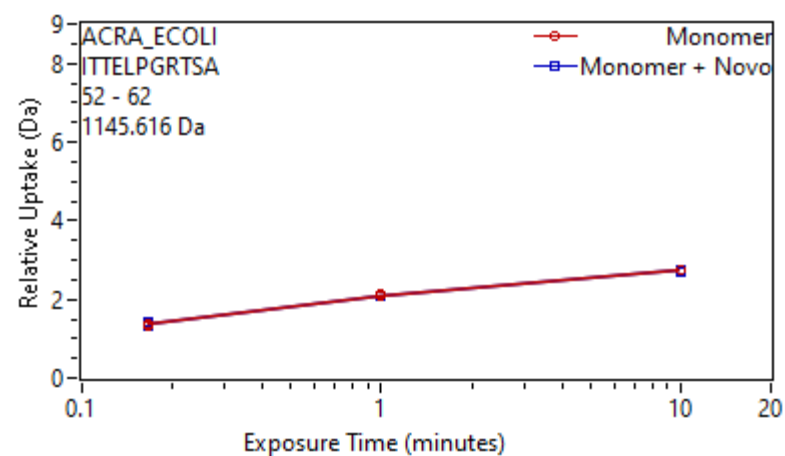

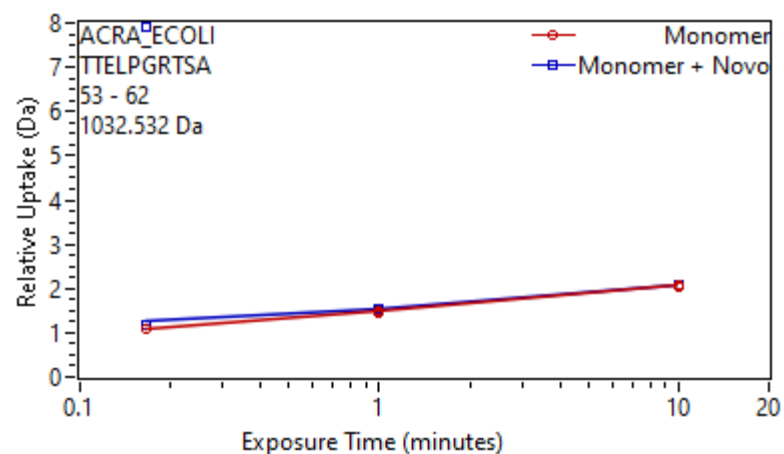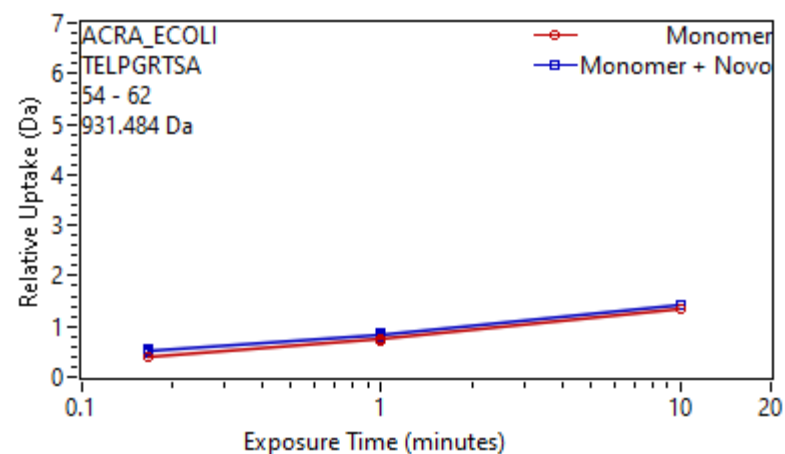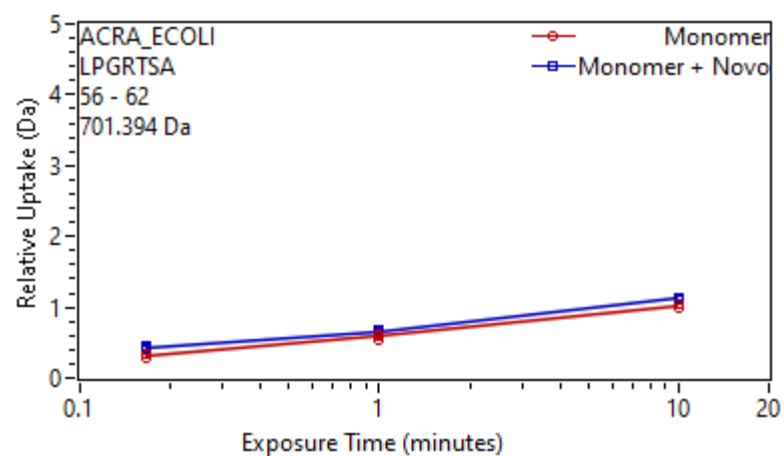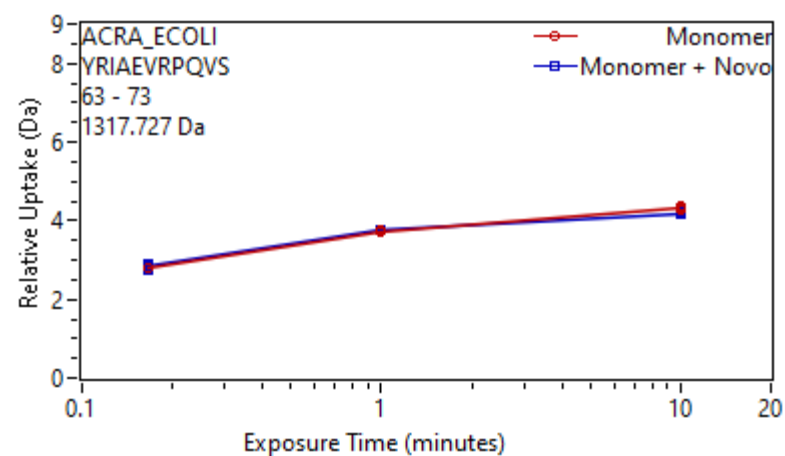

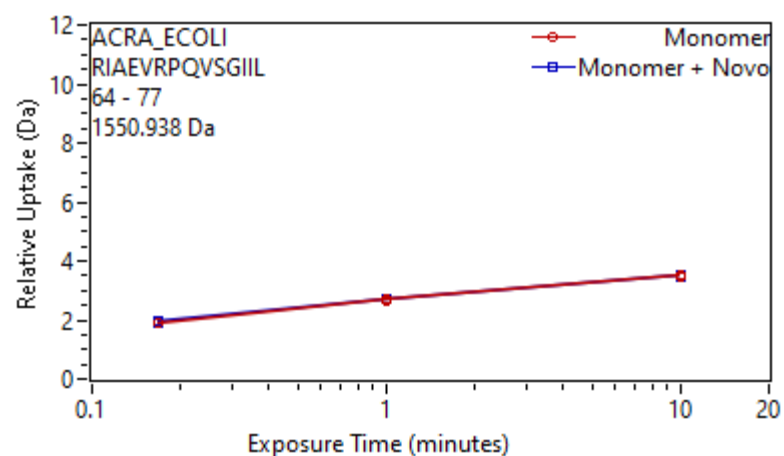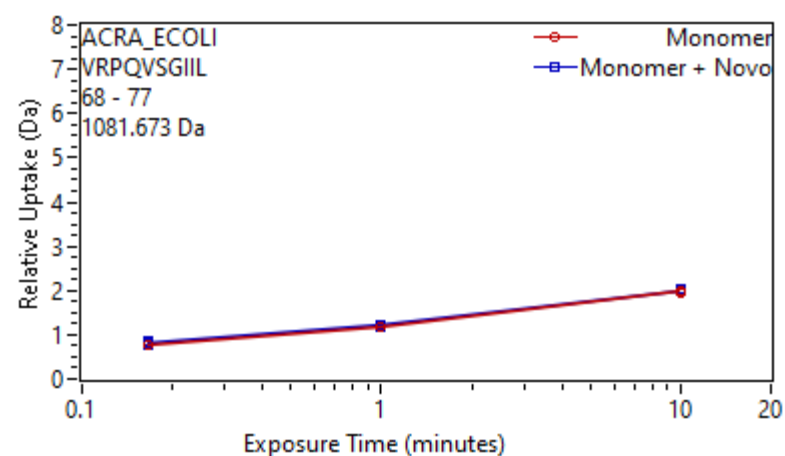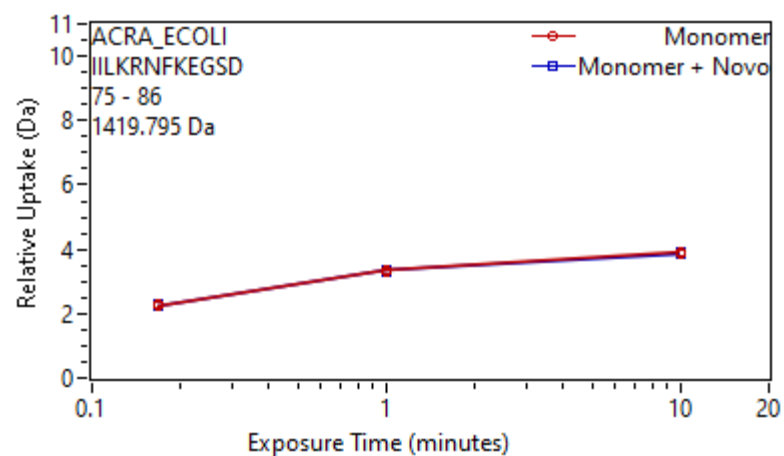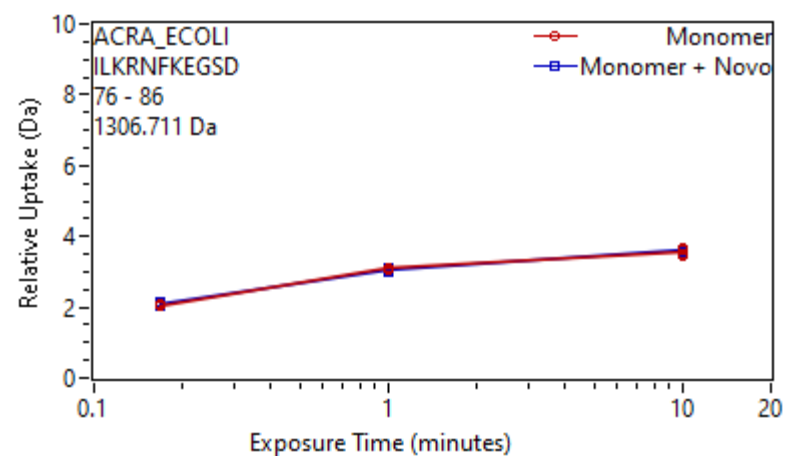

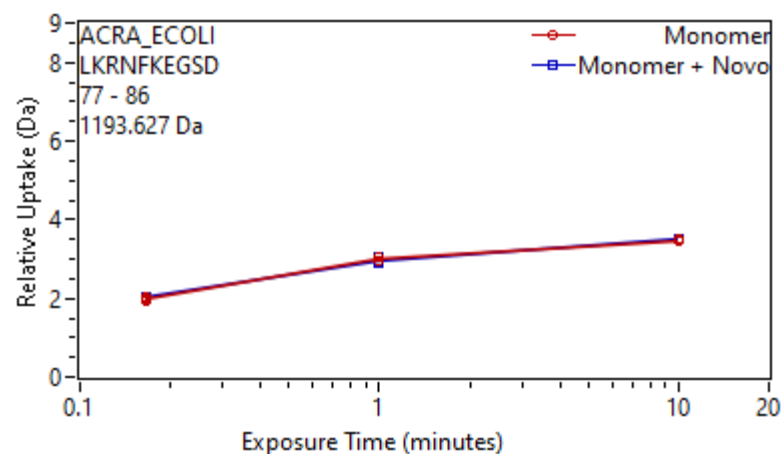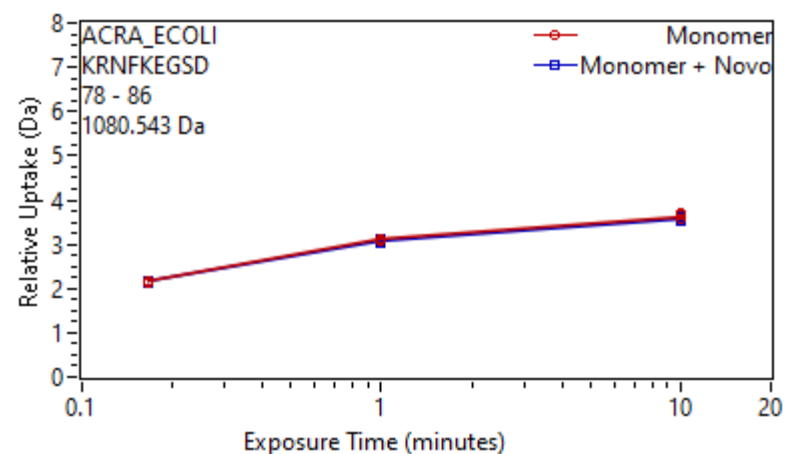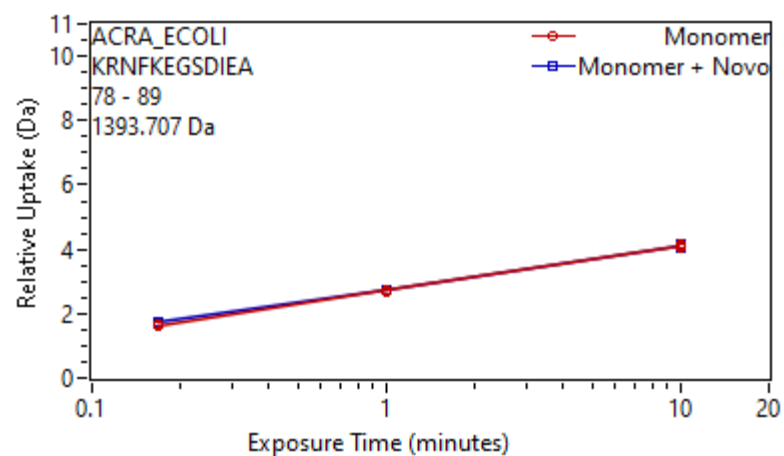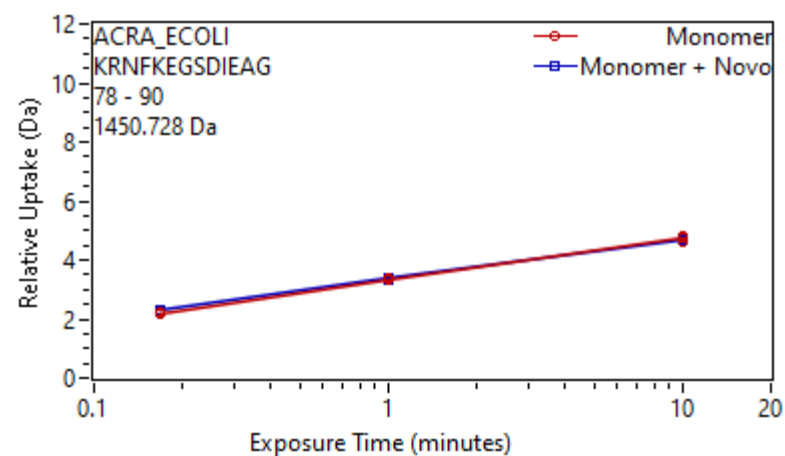

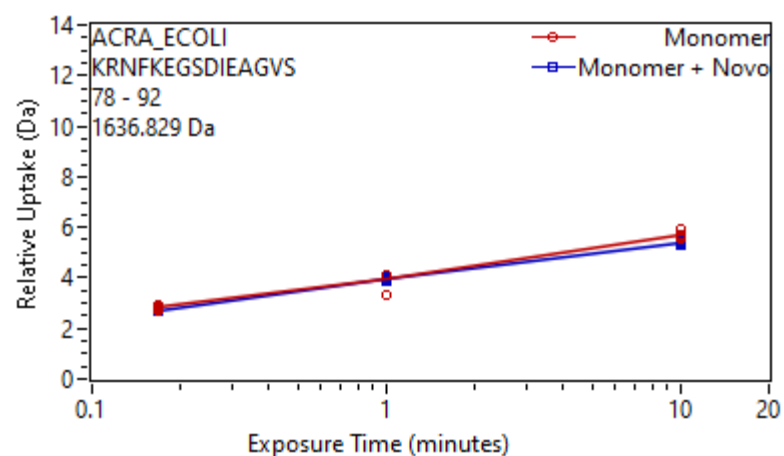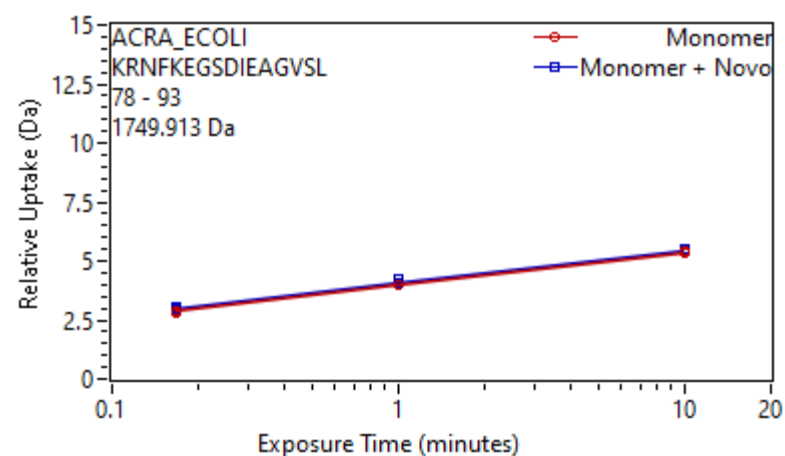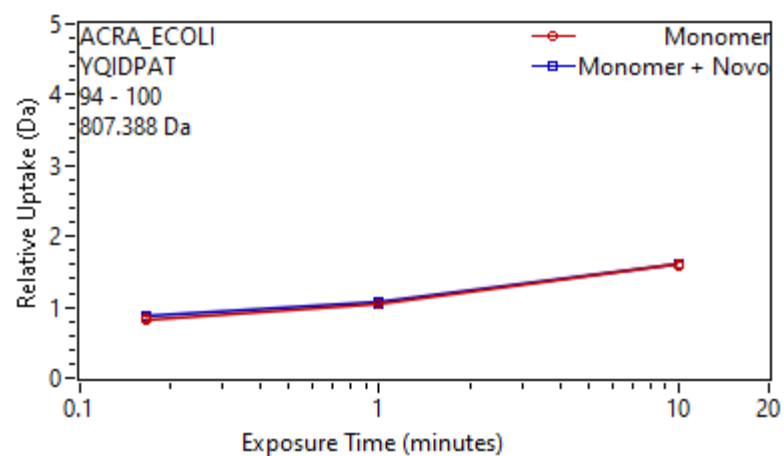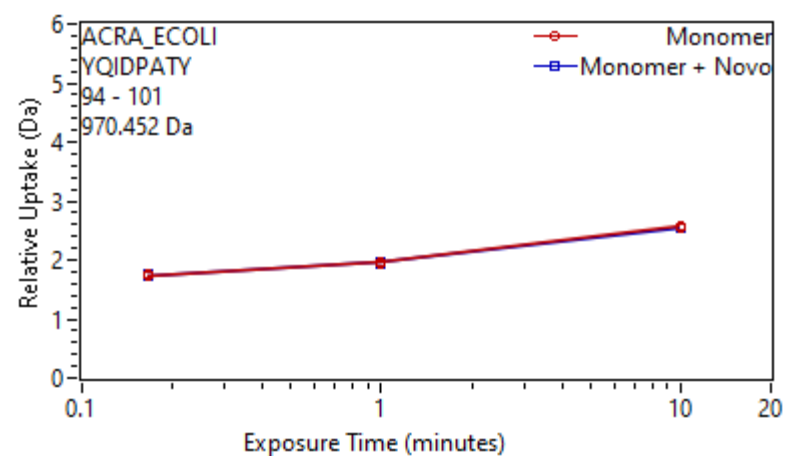

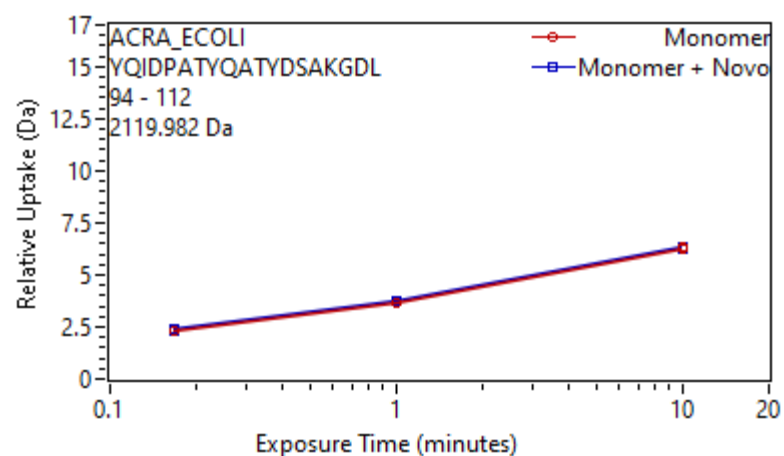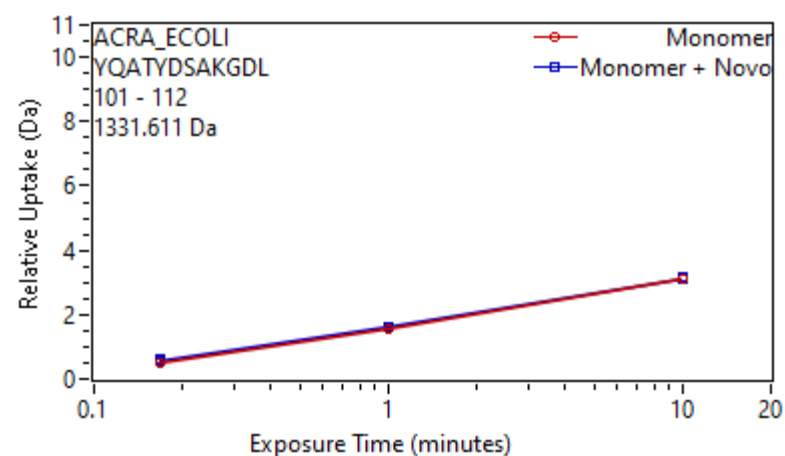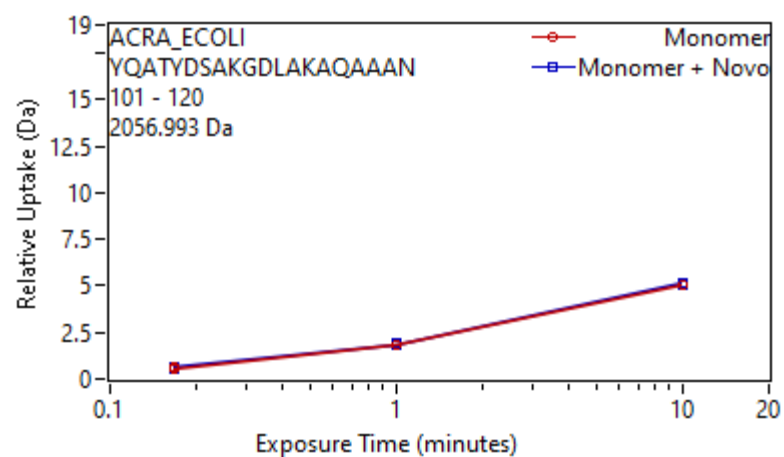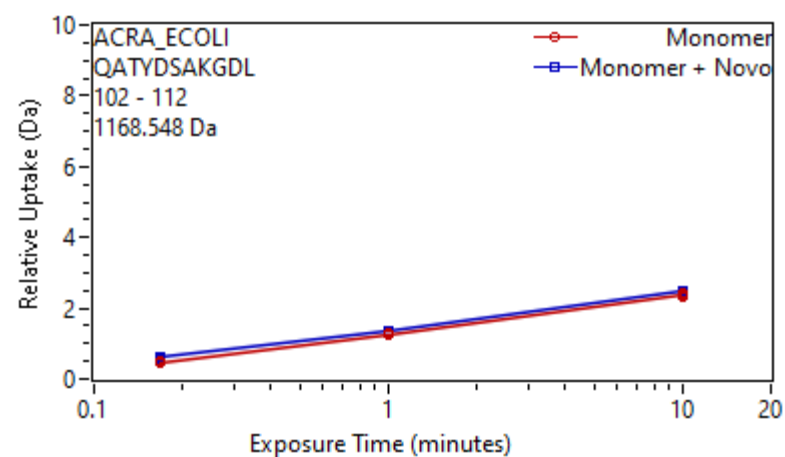

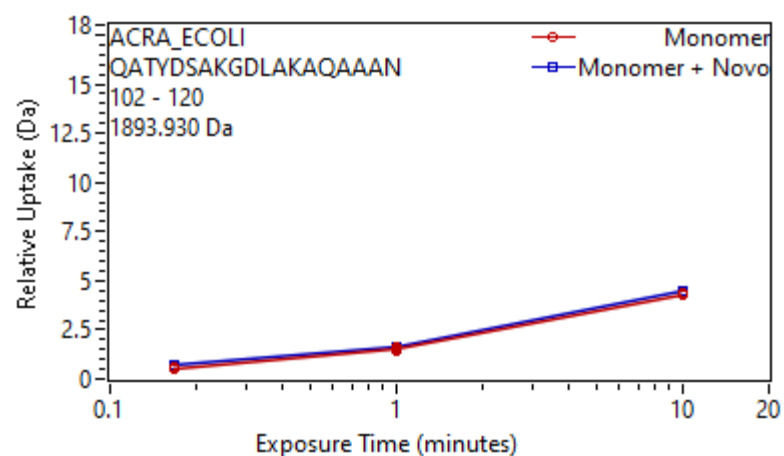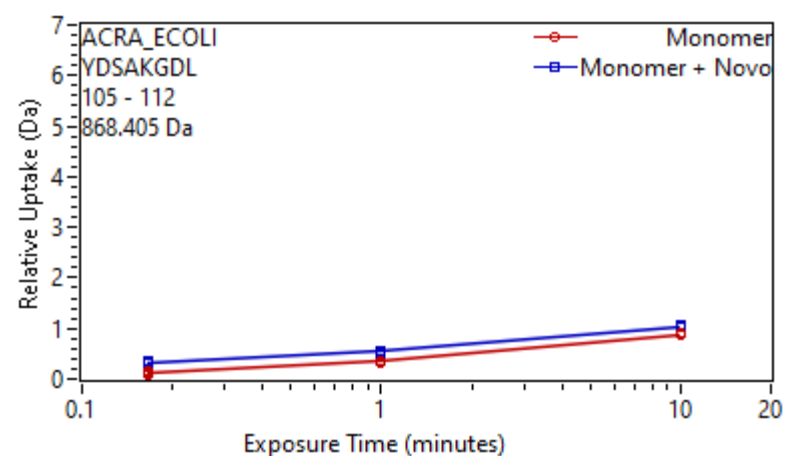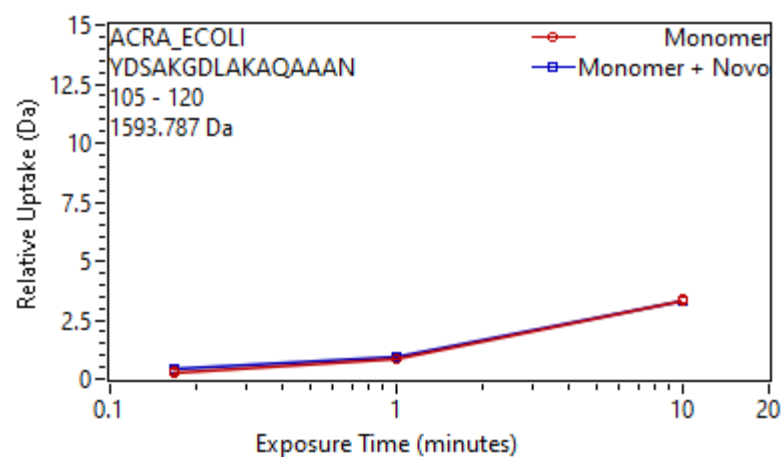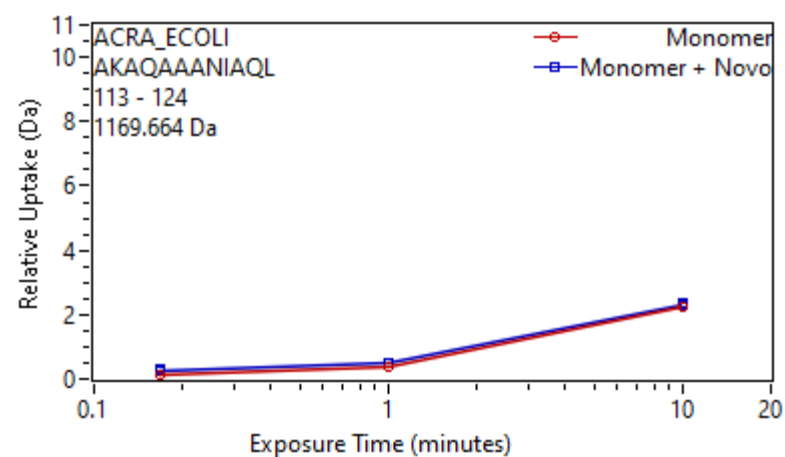

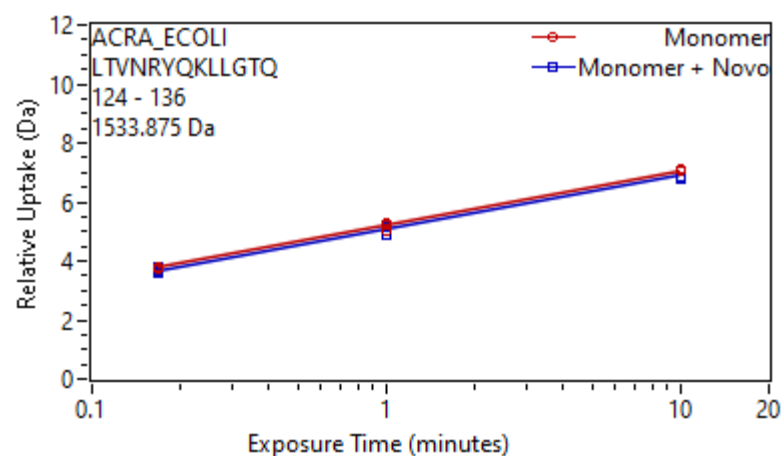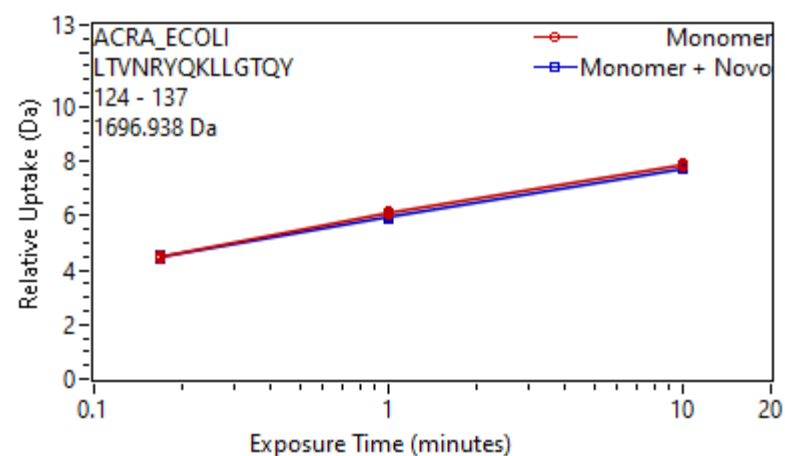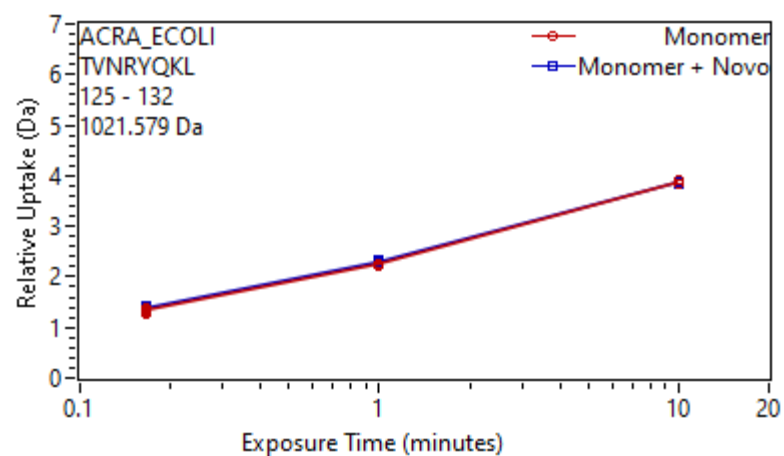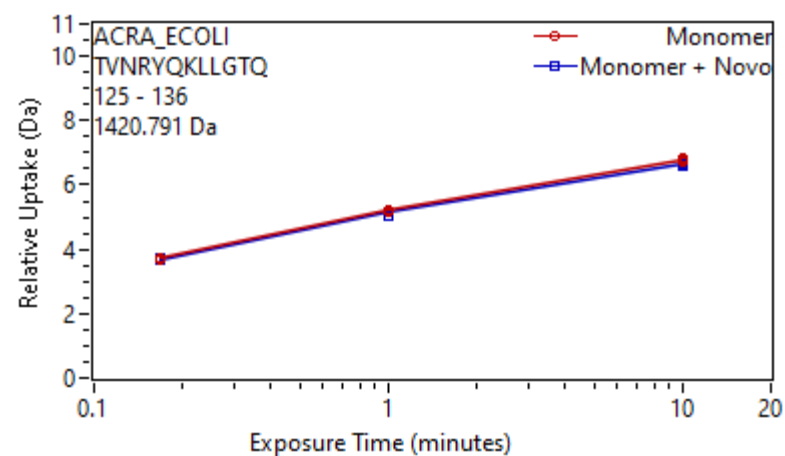

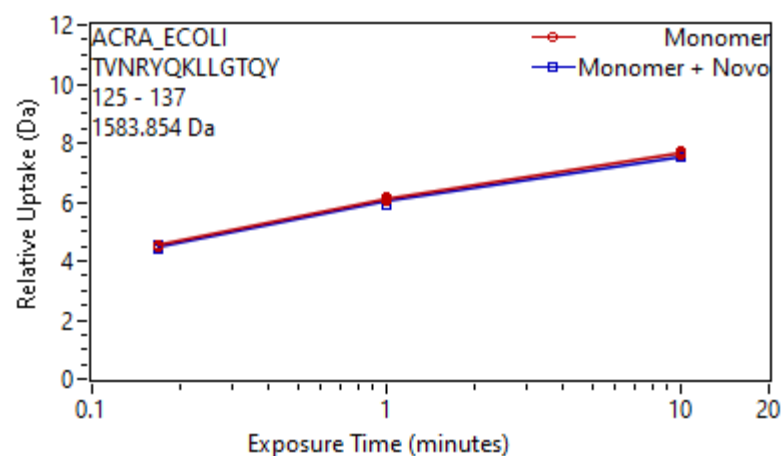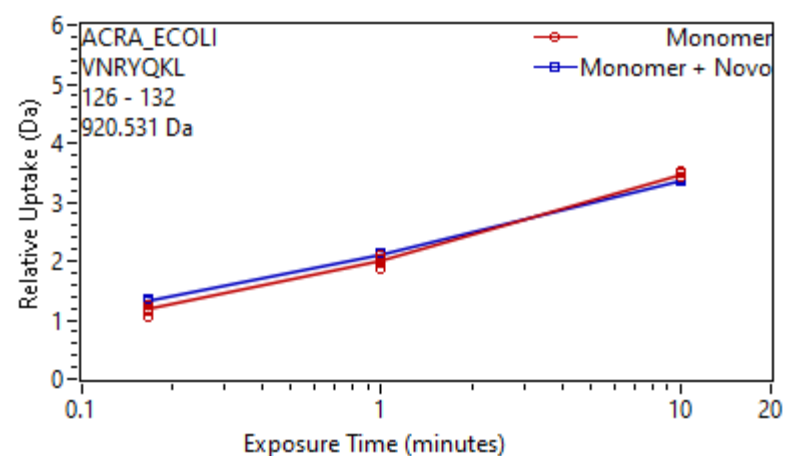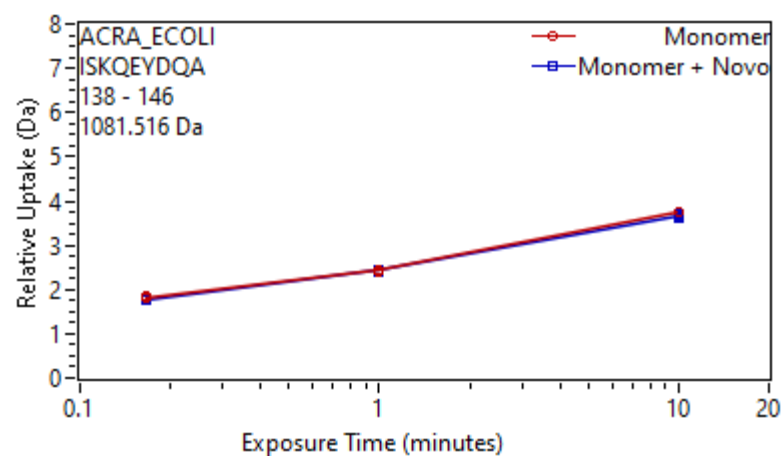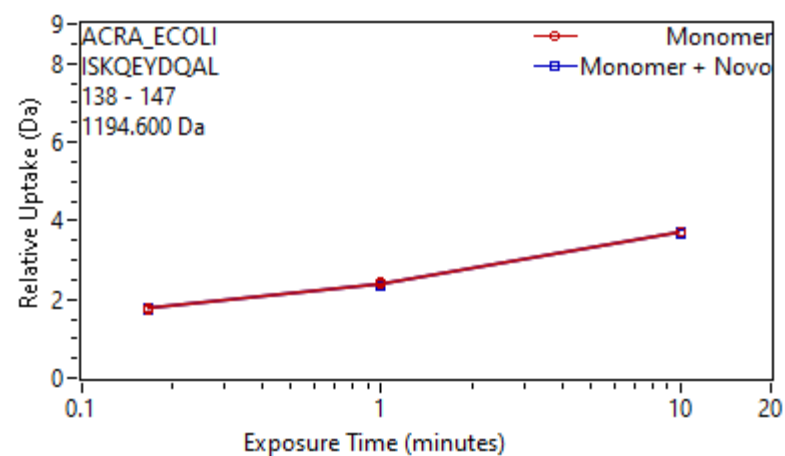

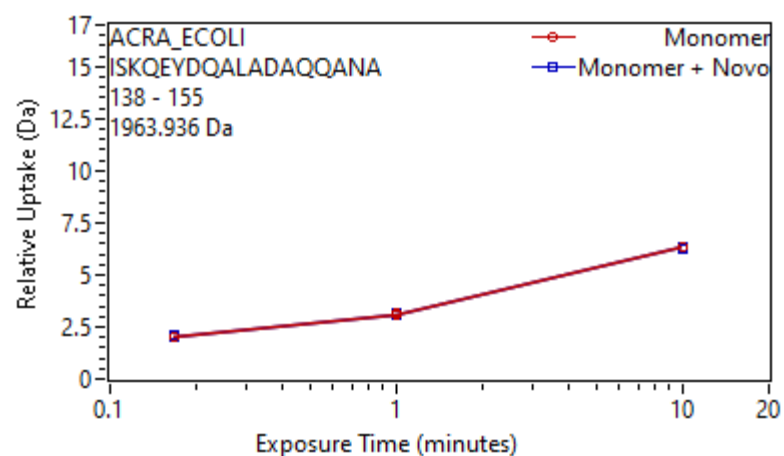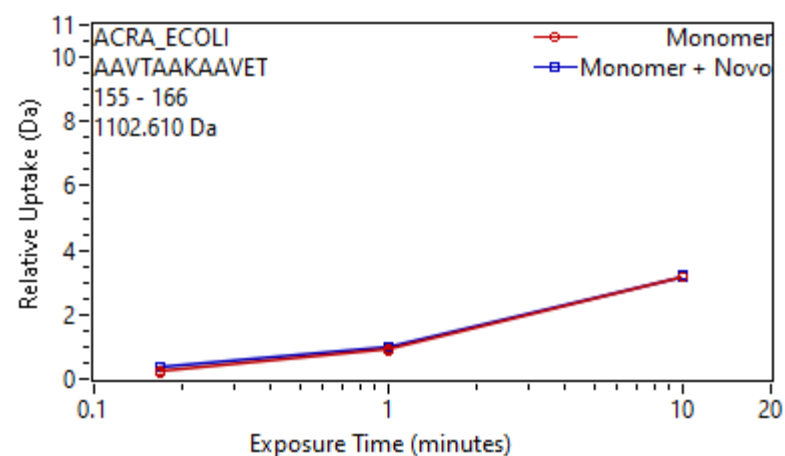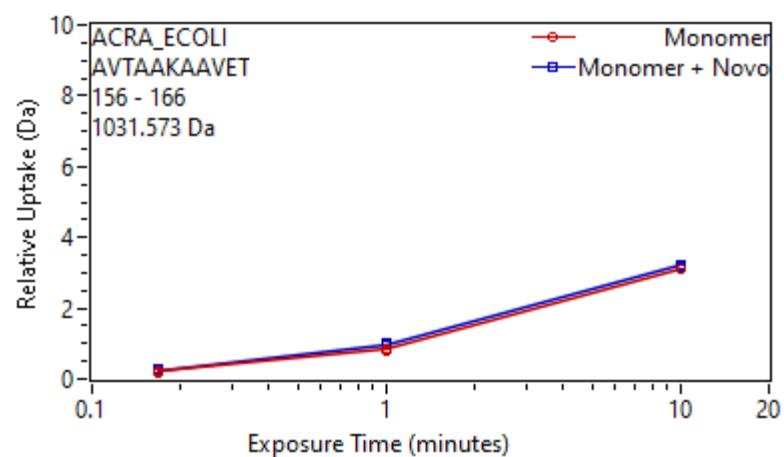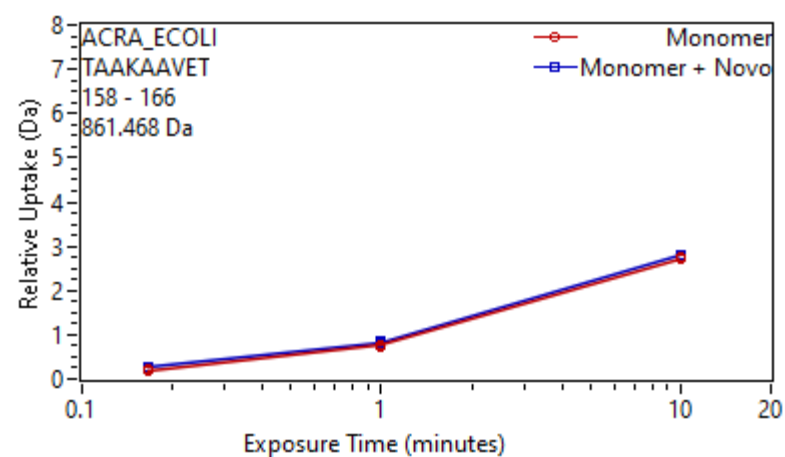

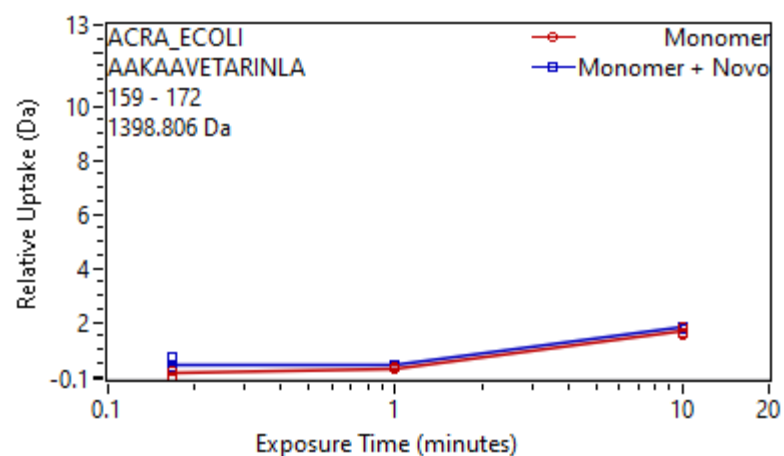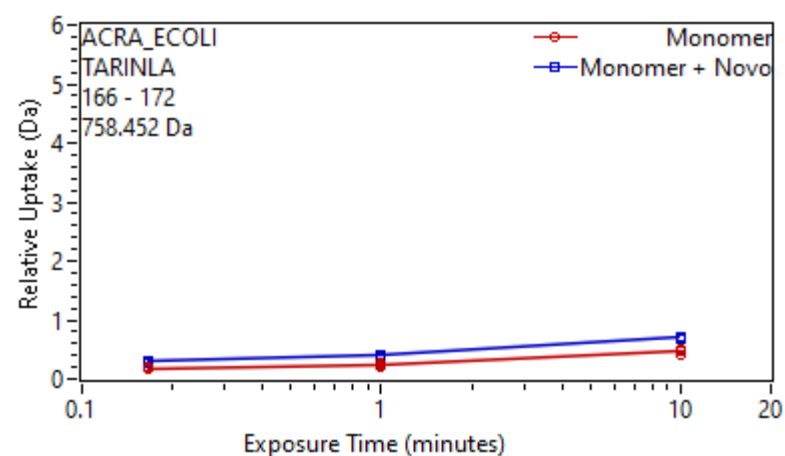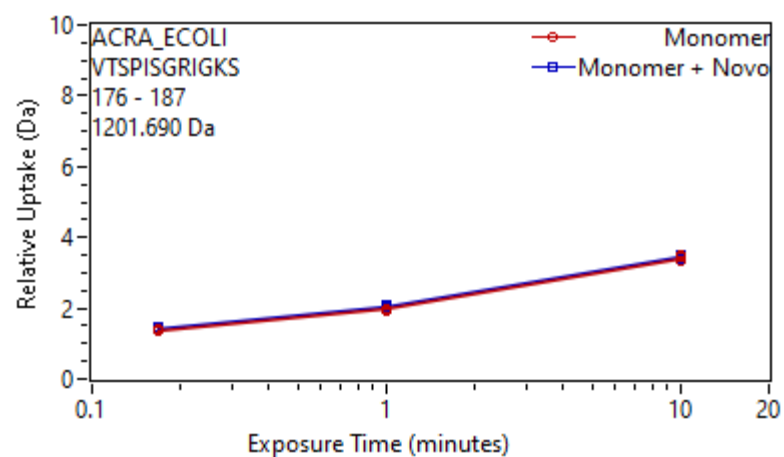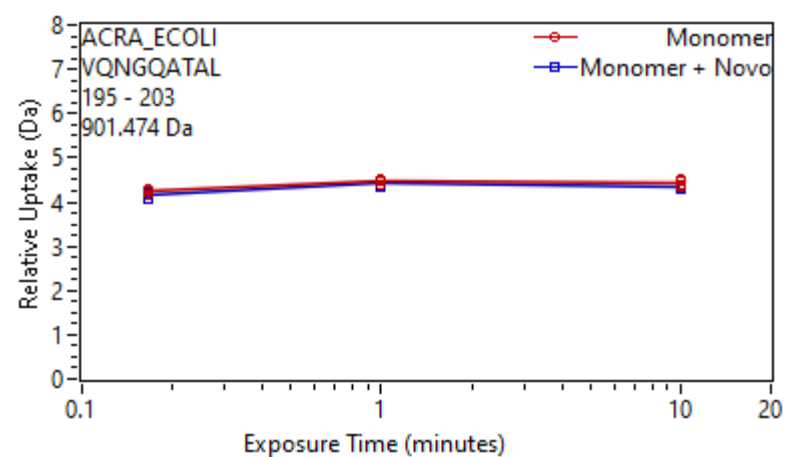

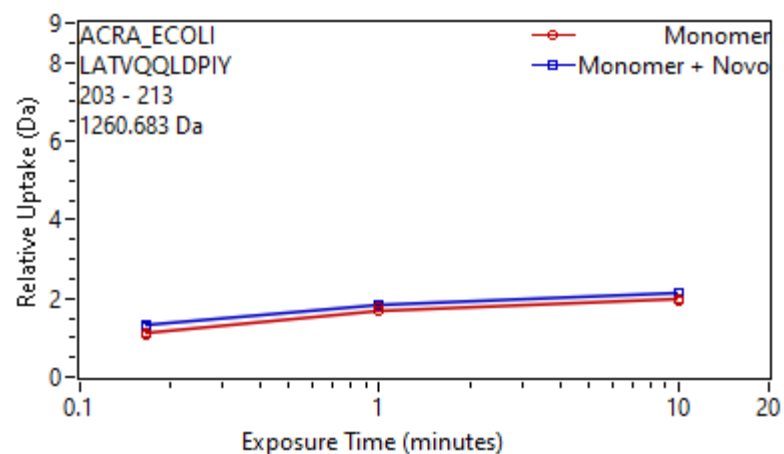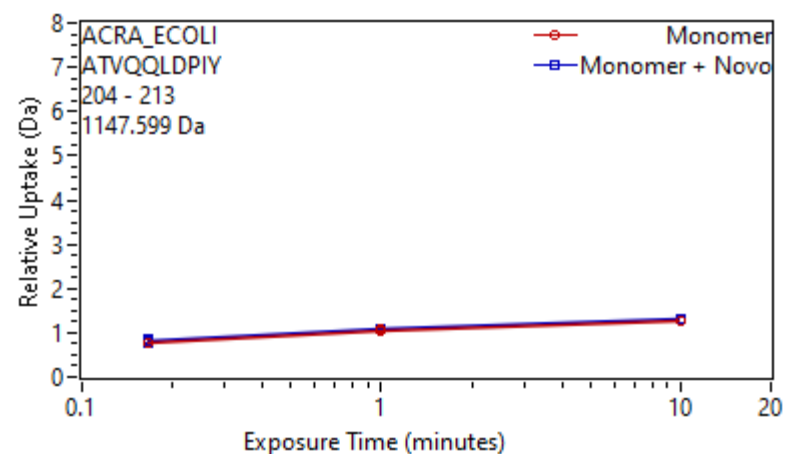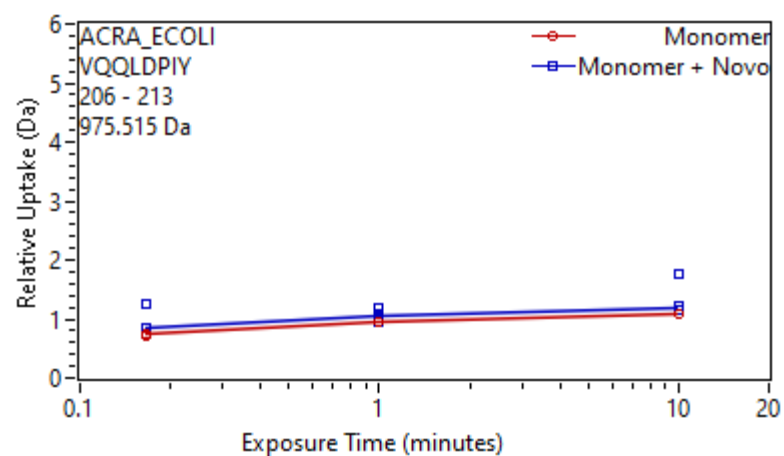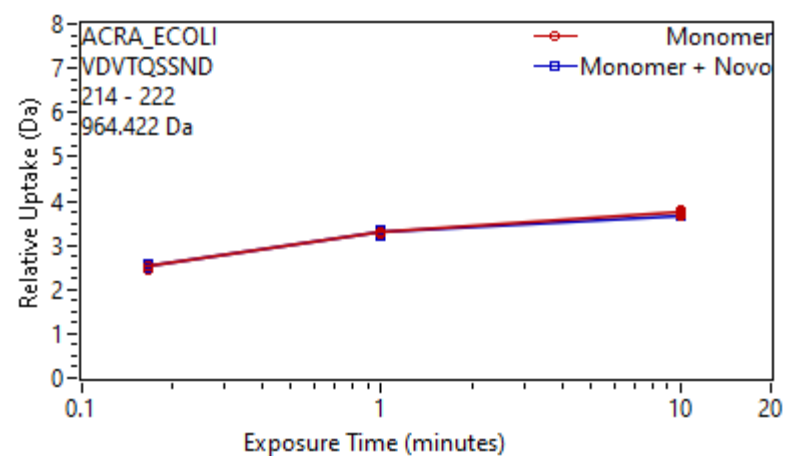

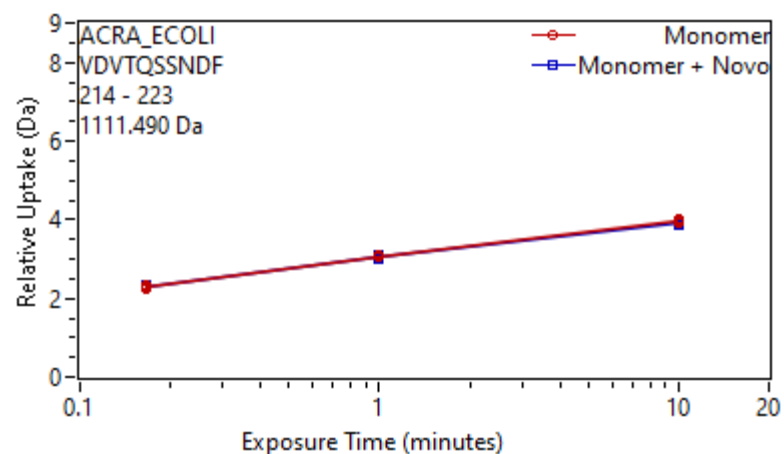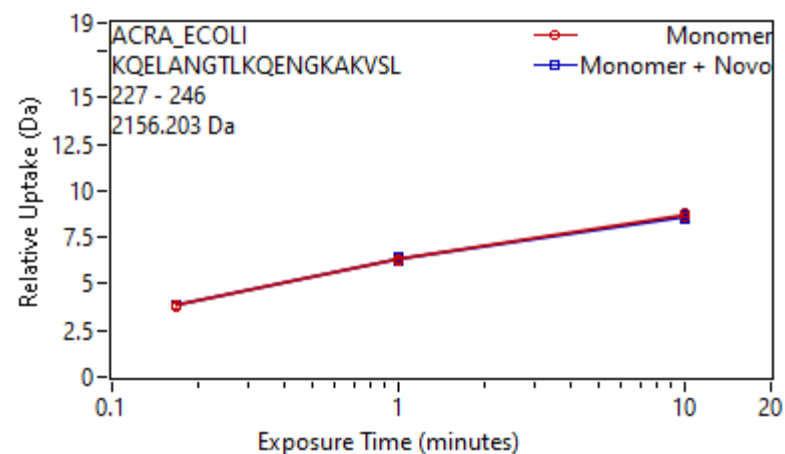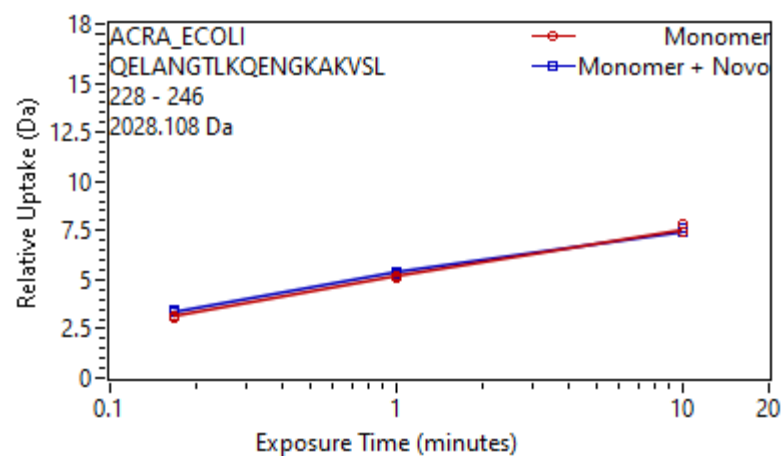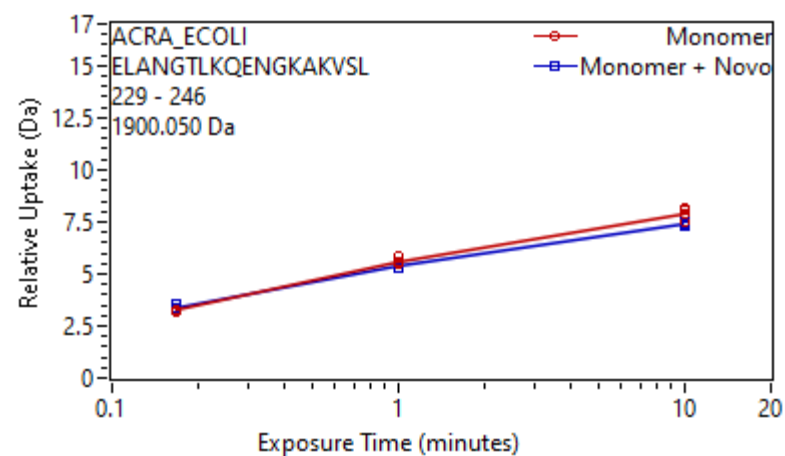

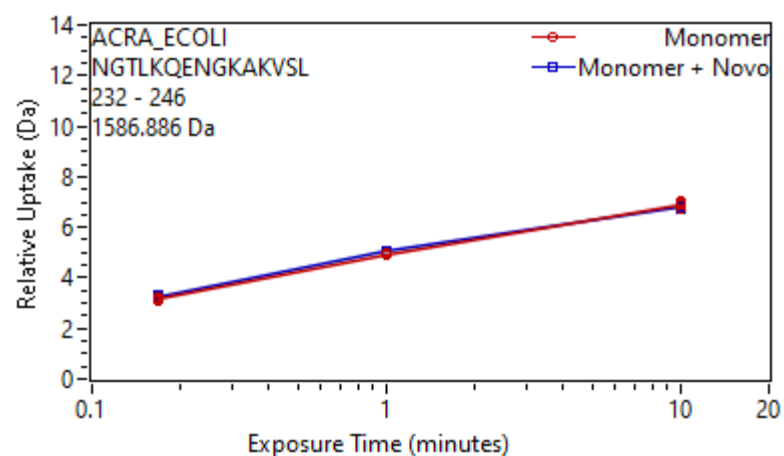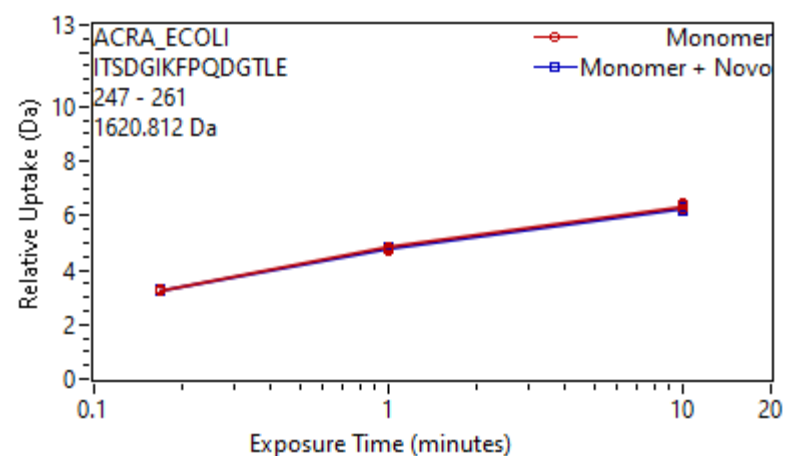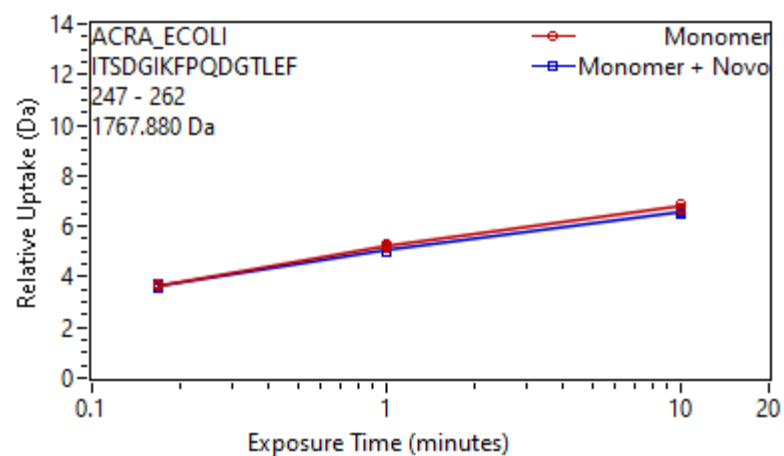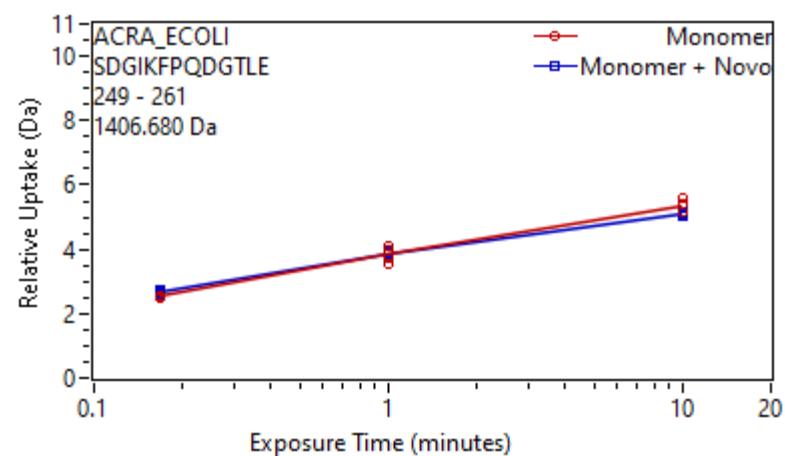

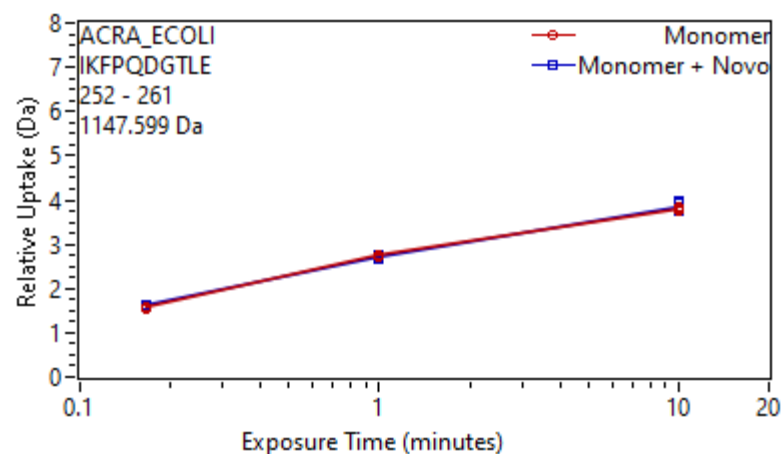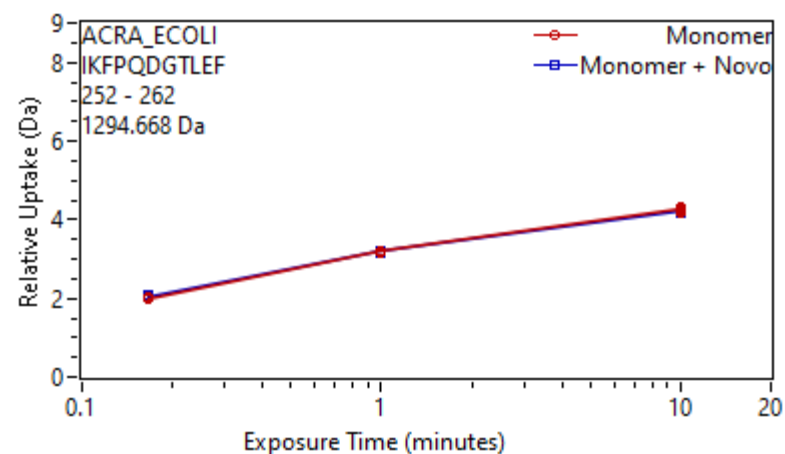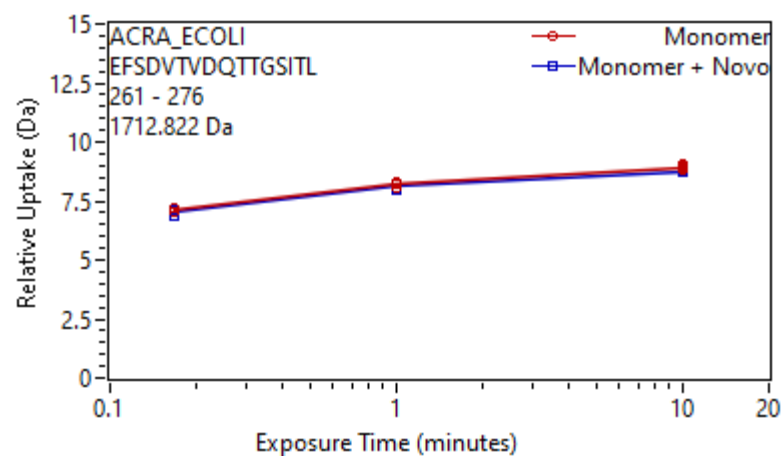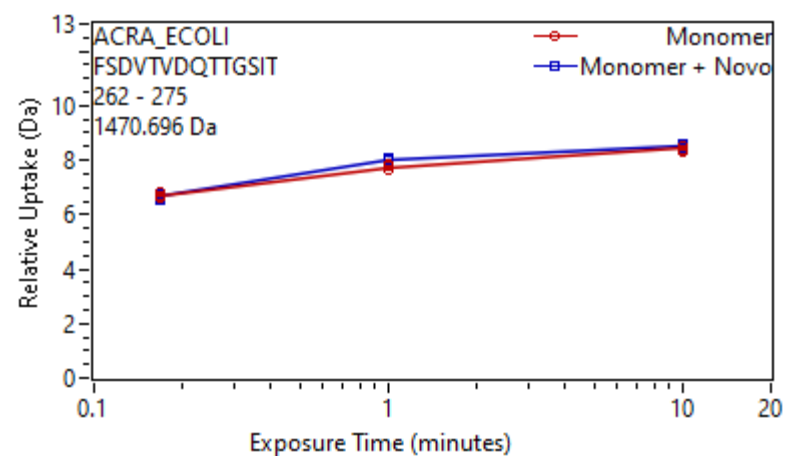

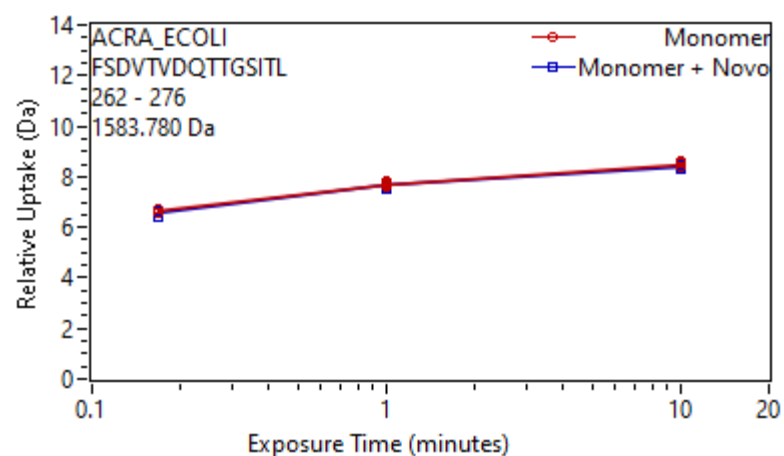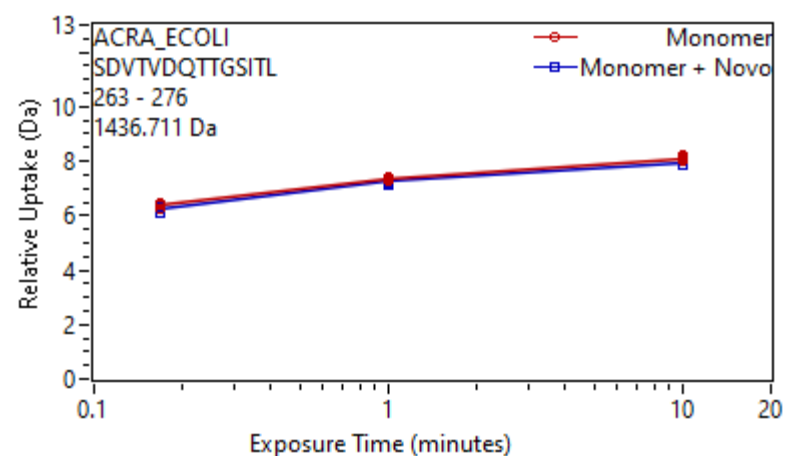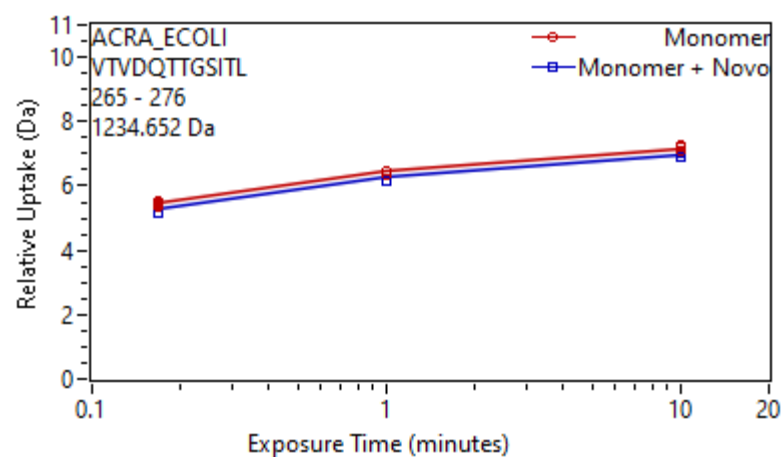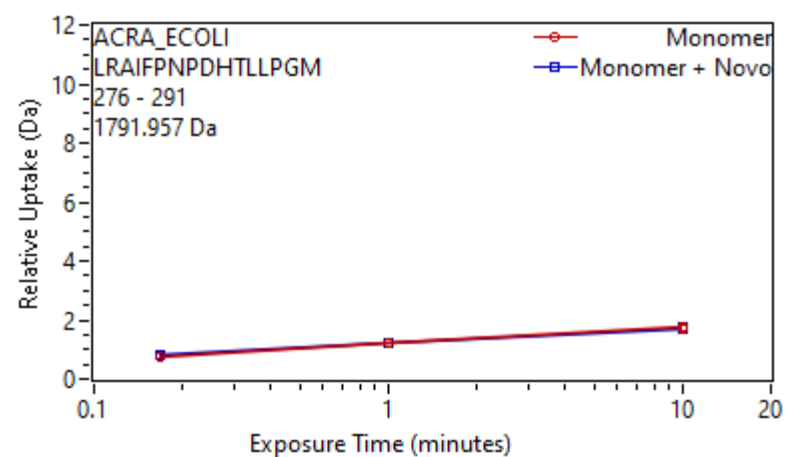

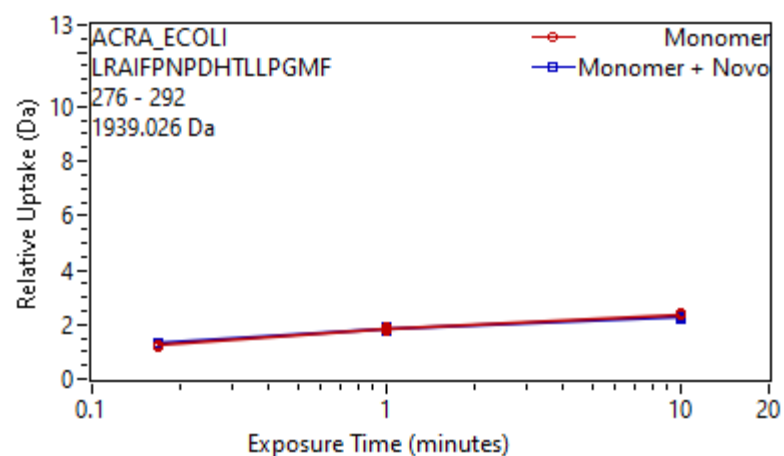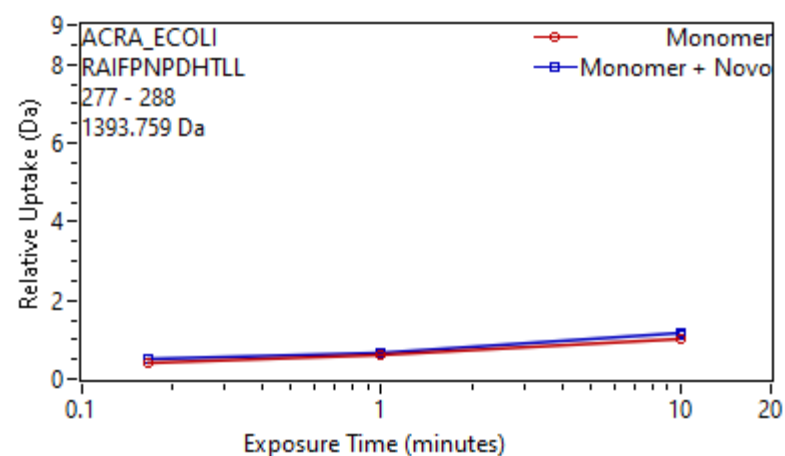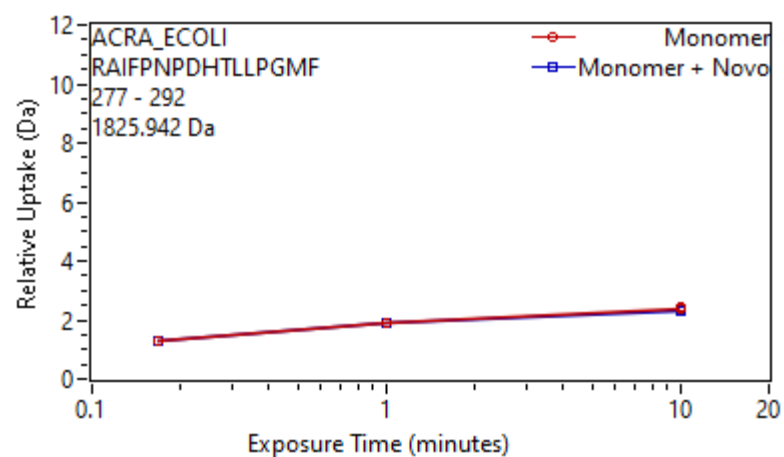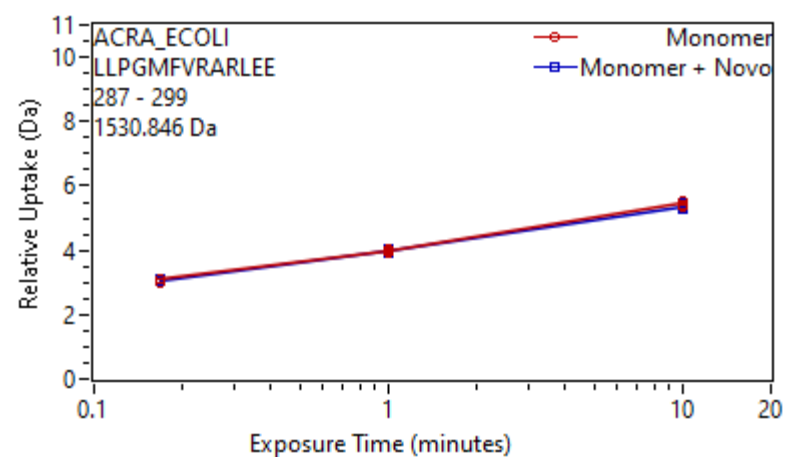

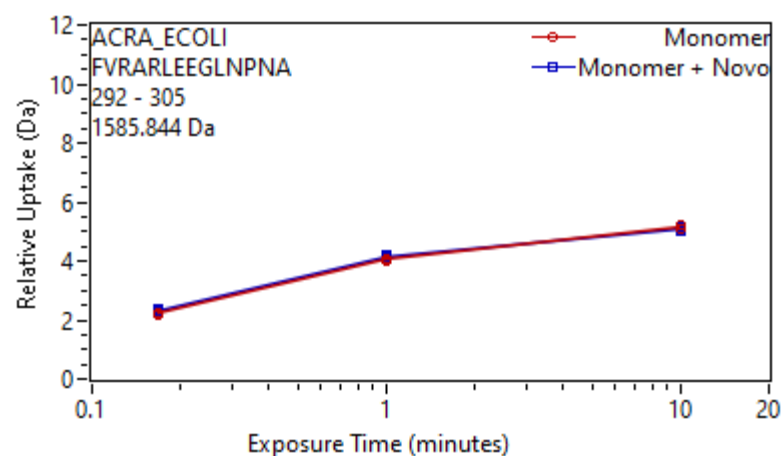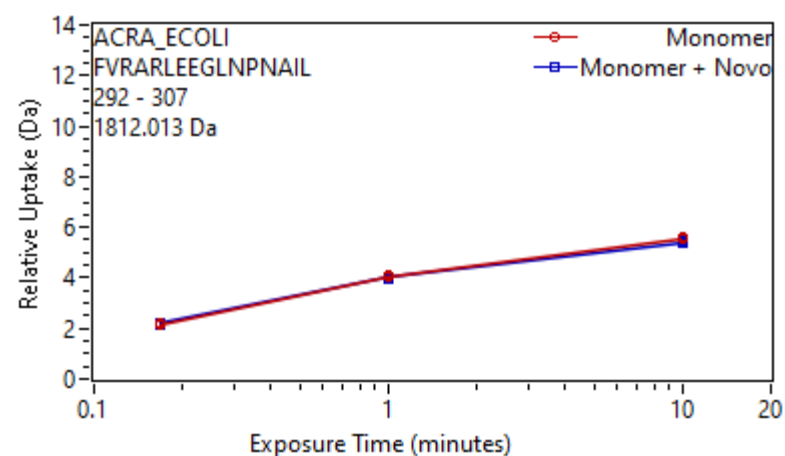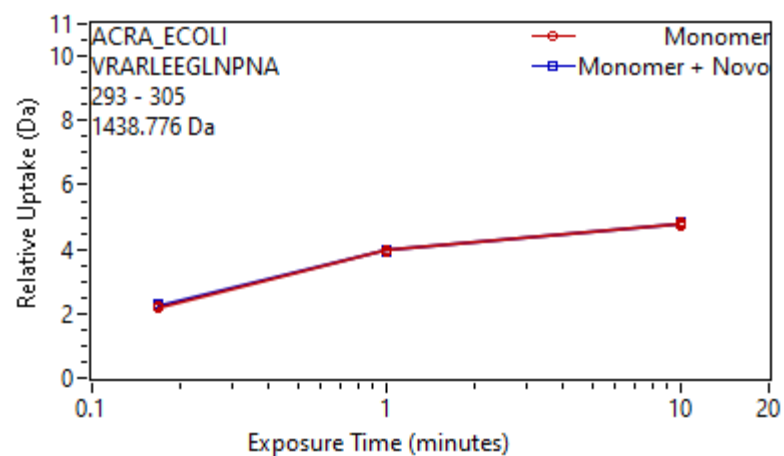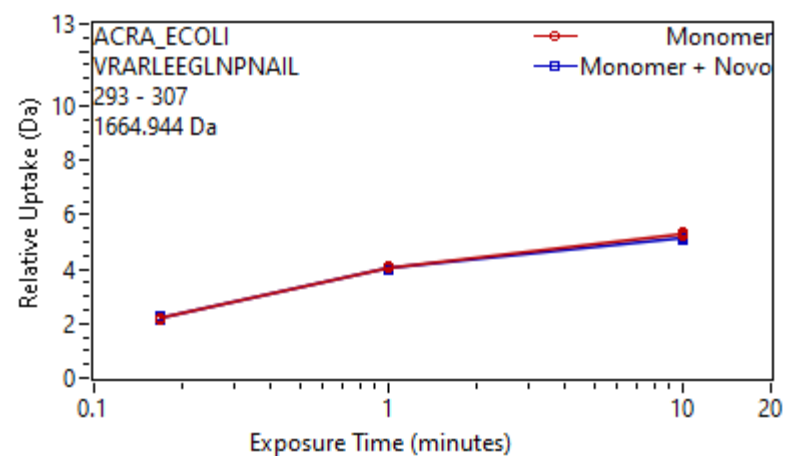

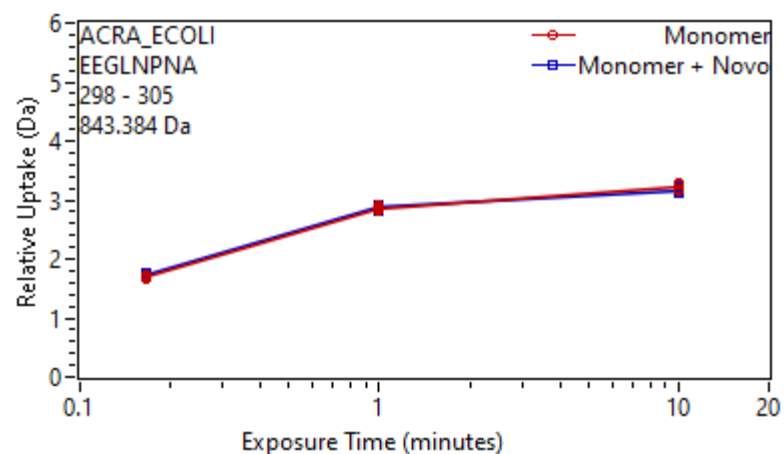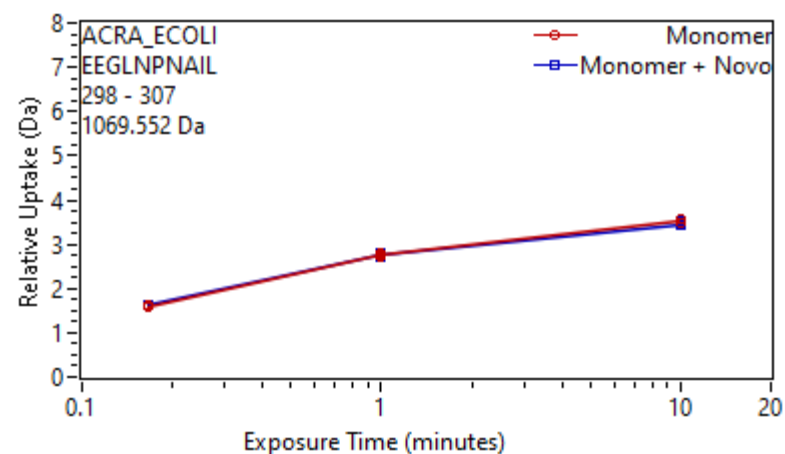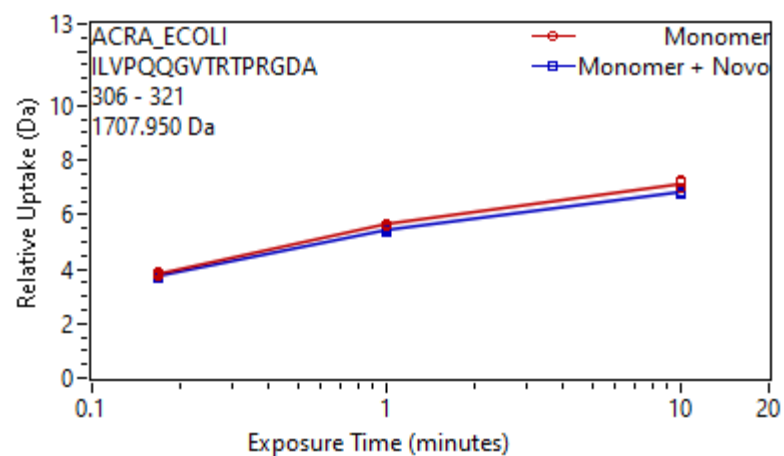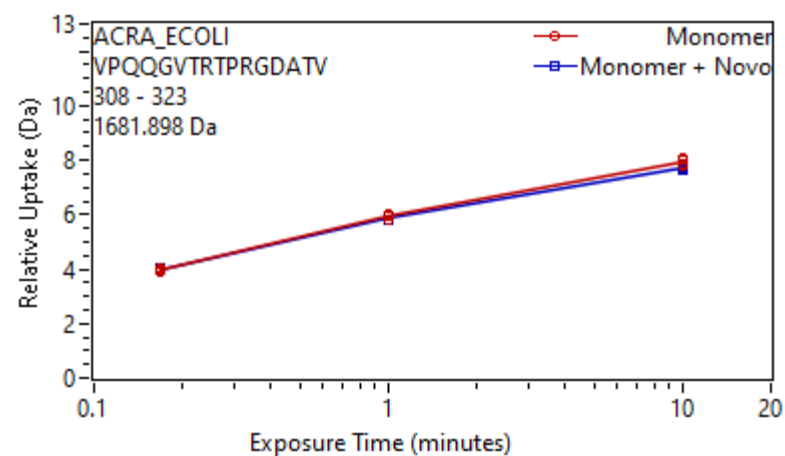

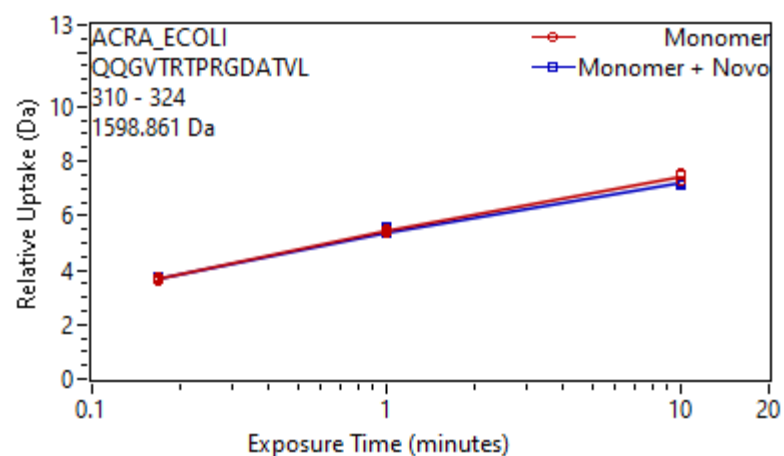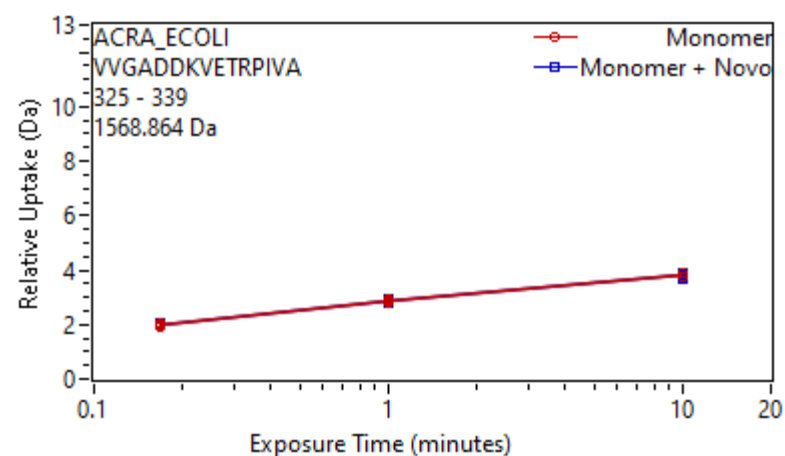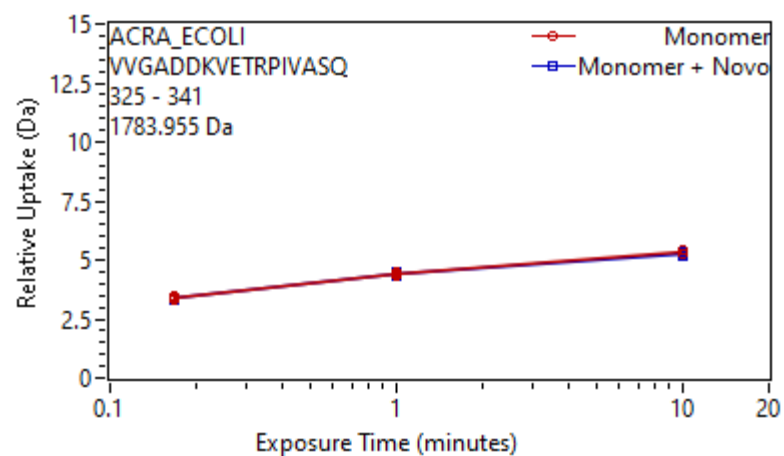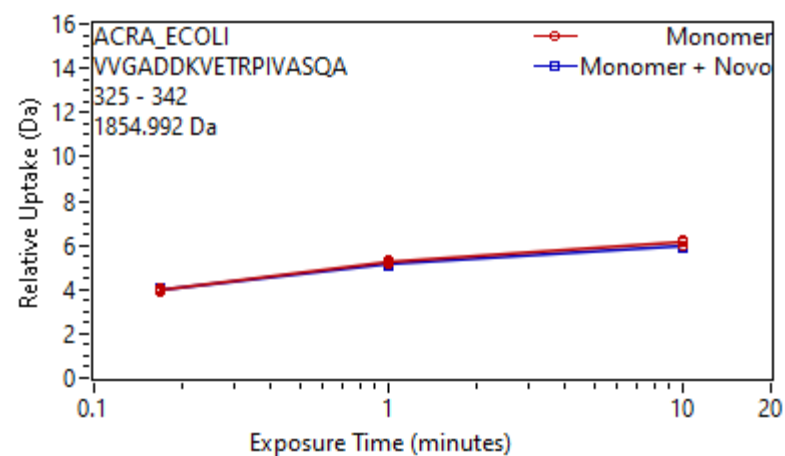

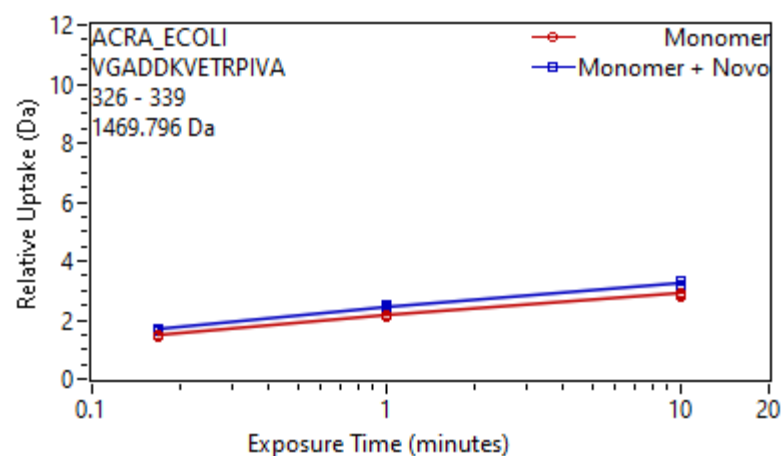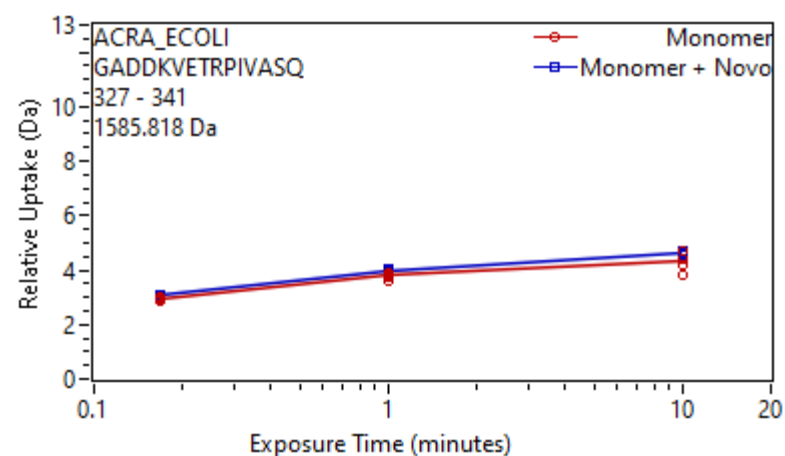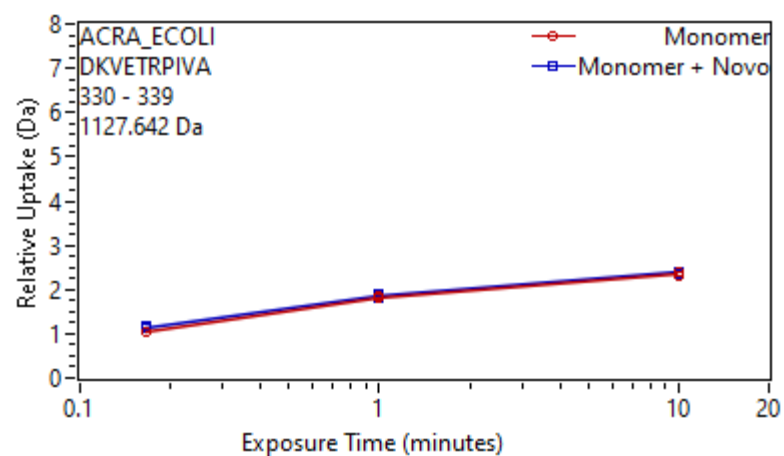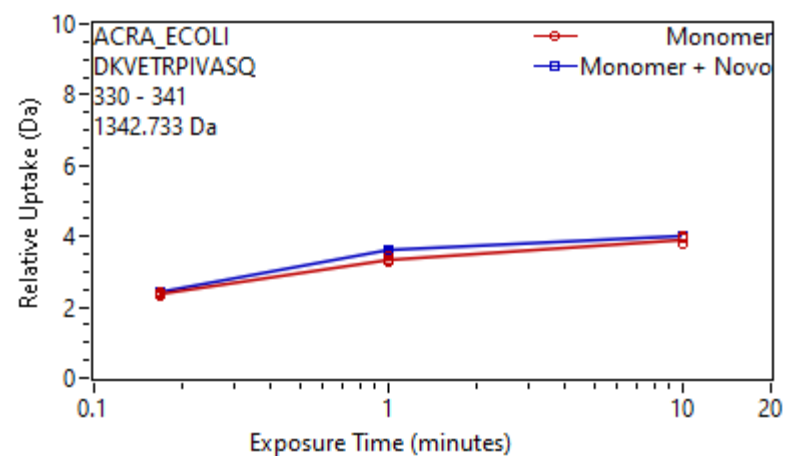

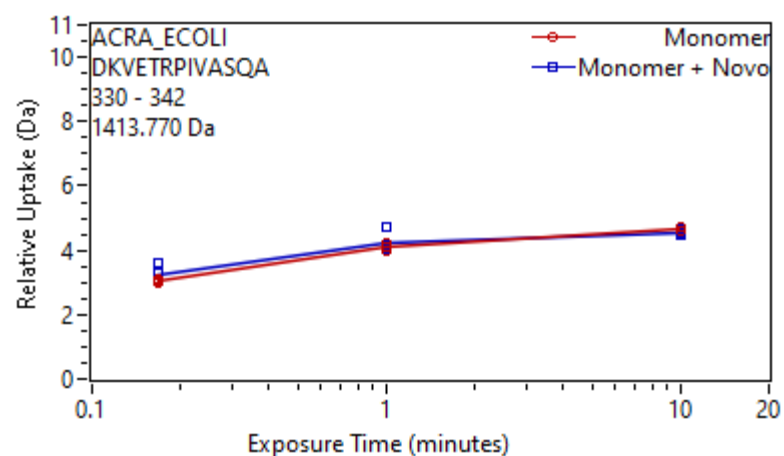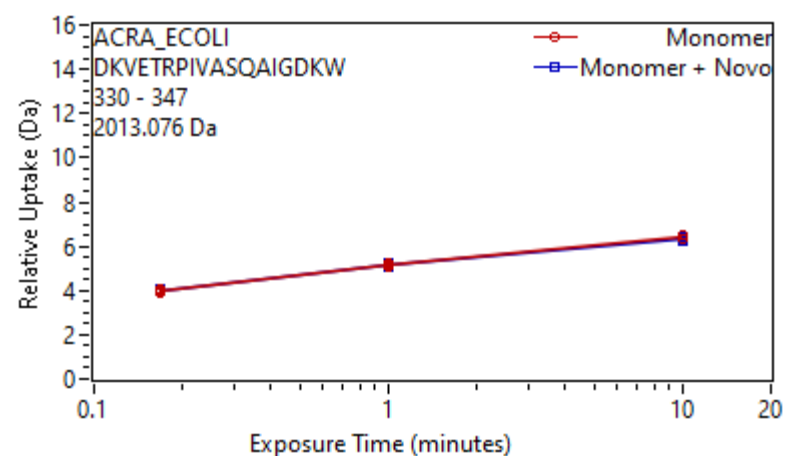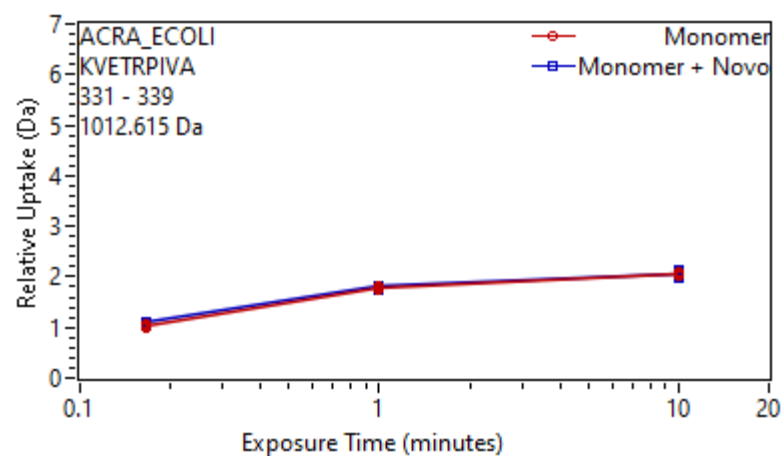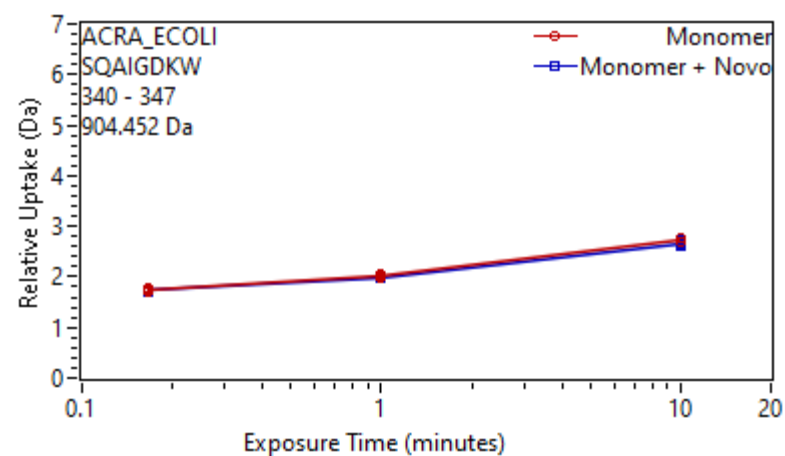

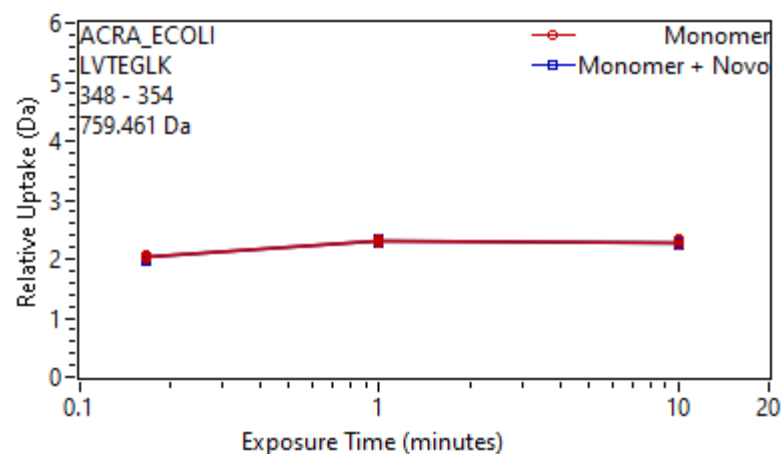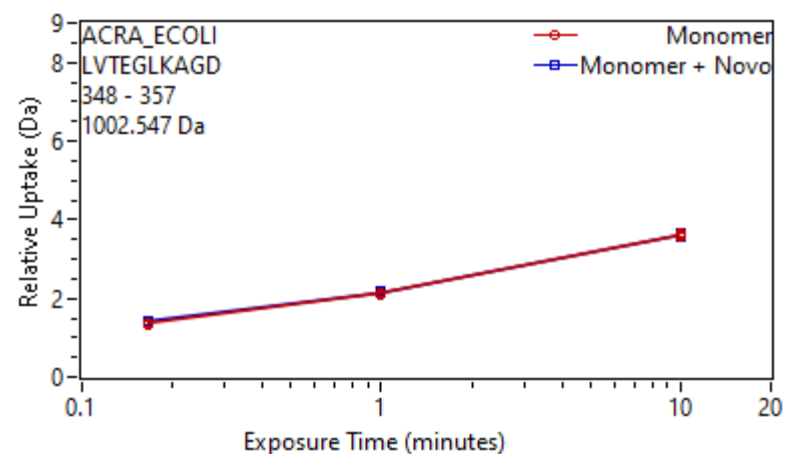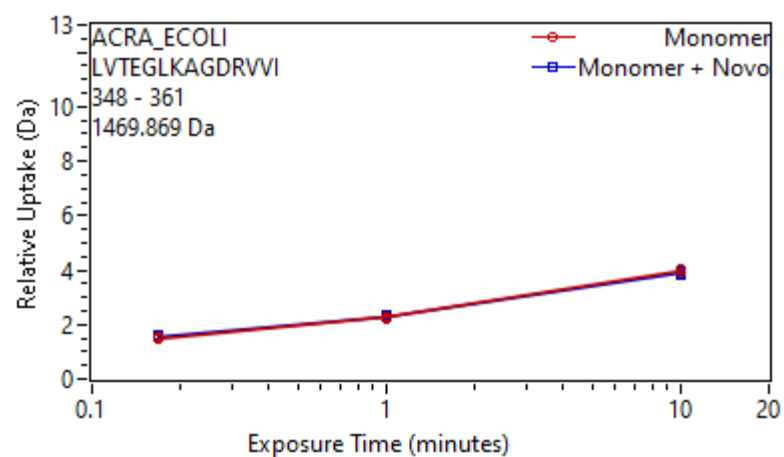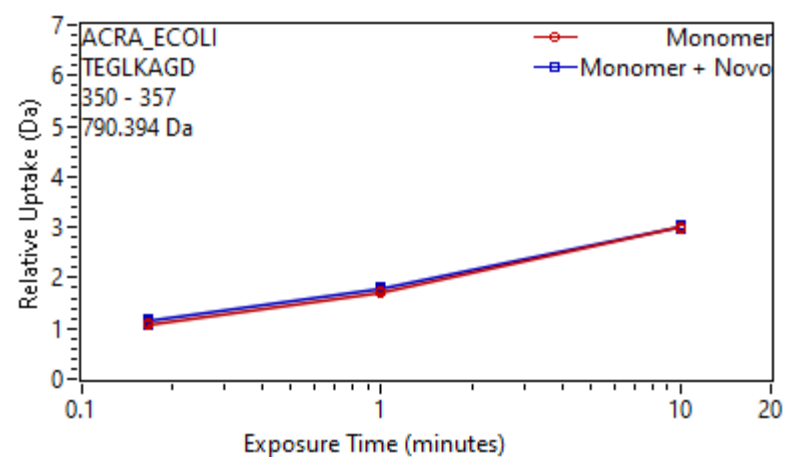

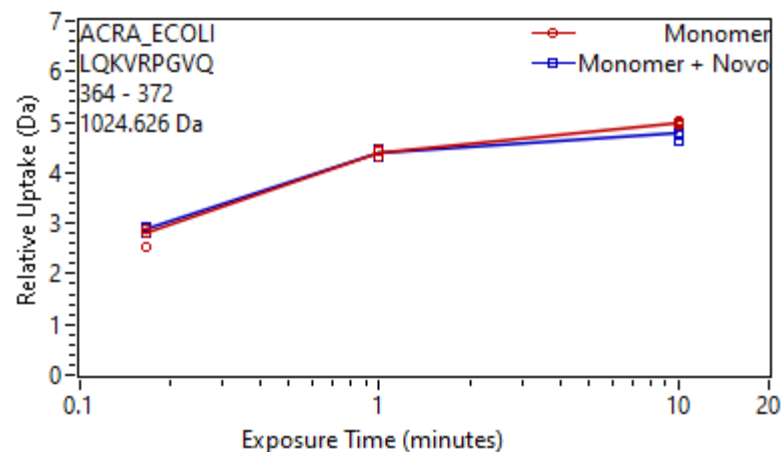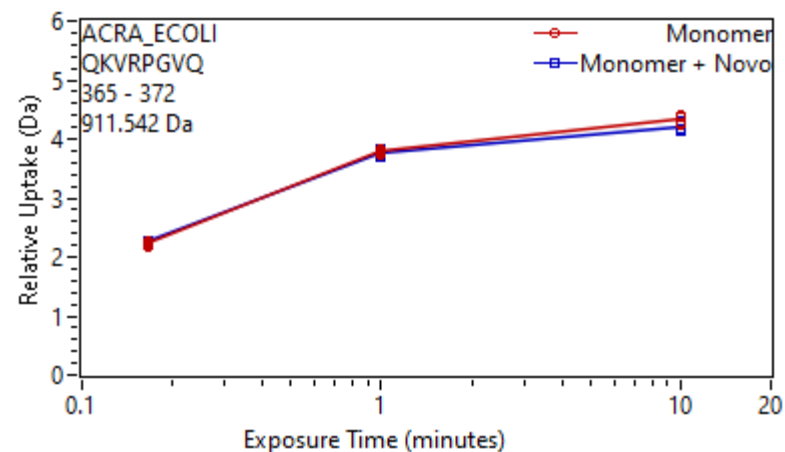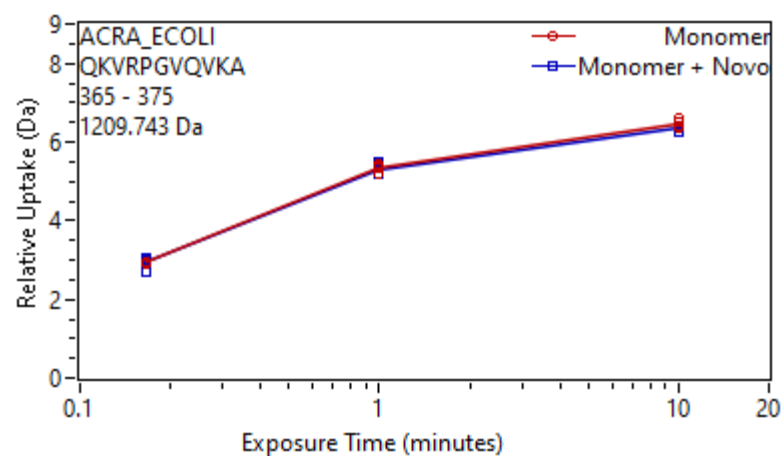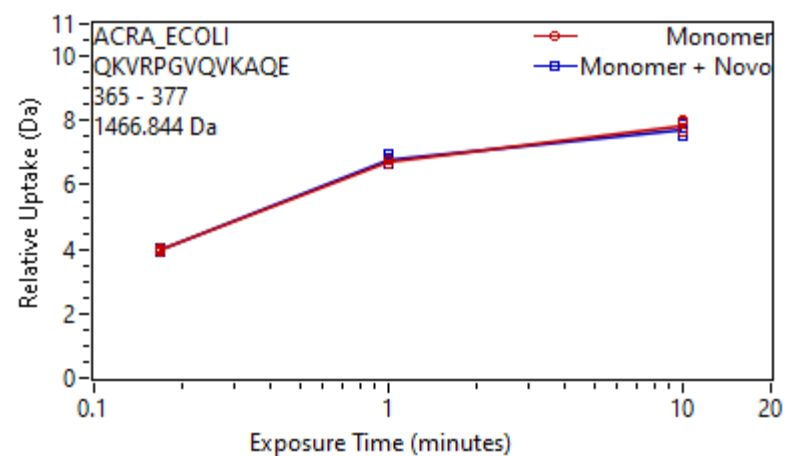

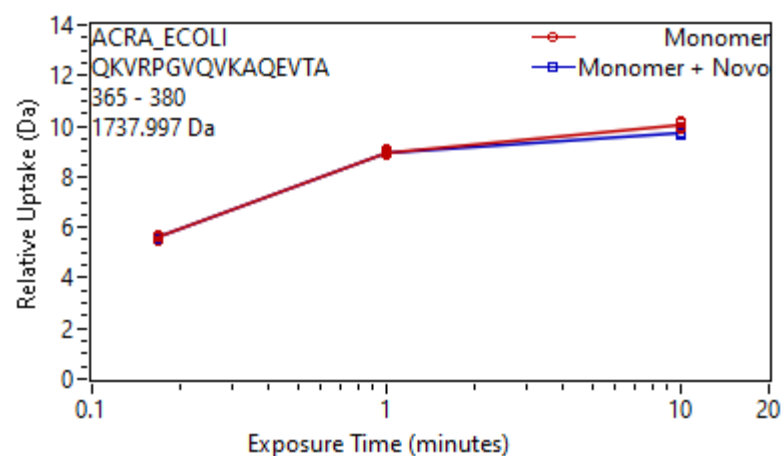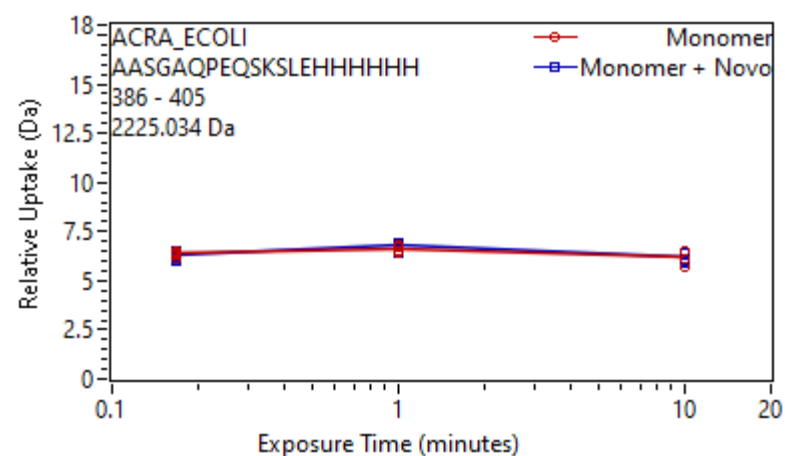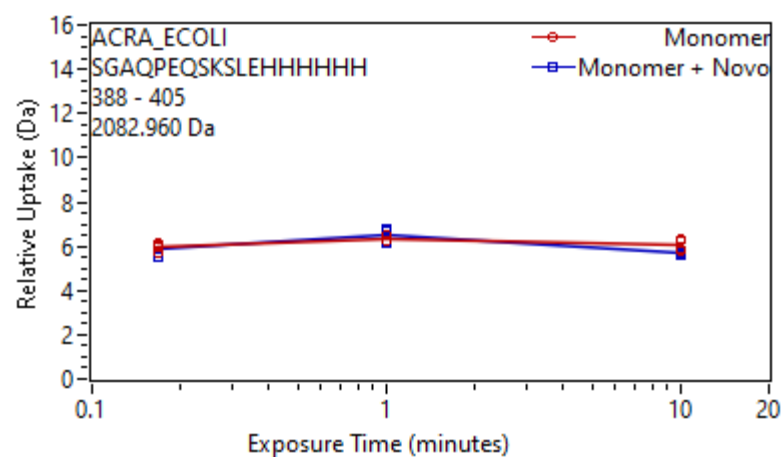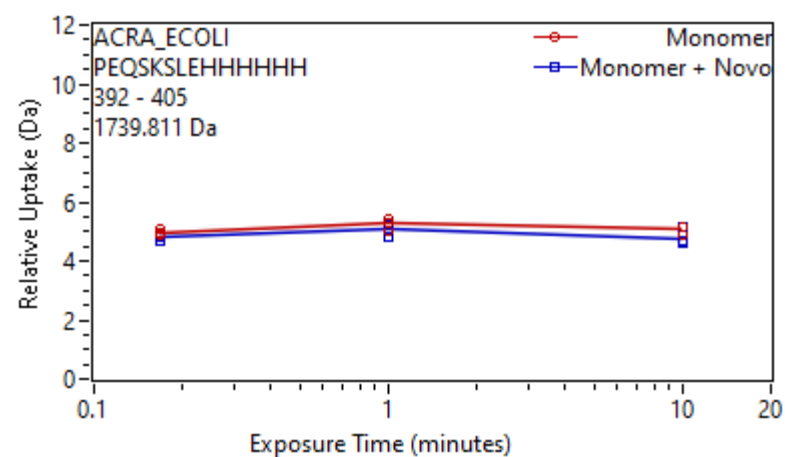

Supplement: Supplementary file 6 — Supplementary Data 3 [file 41467_2023_39615_MOESM6_ESM.zip › Supplementary Data 3/AcrAs_novobiocin_uptakeplots.pdf]

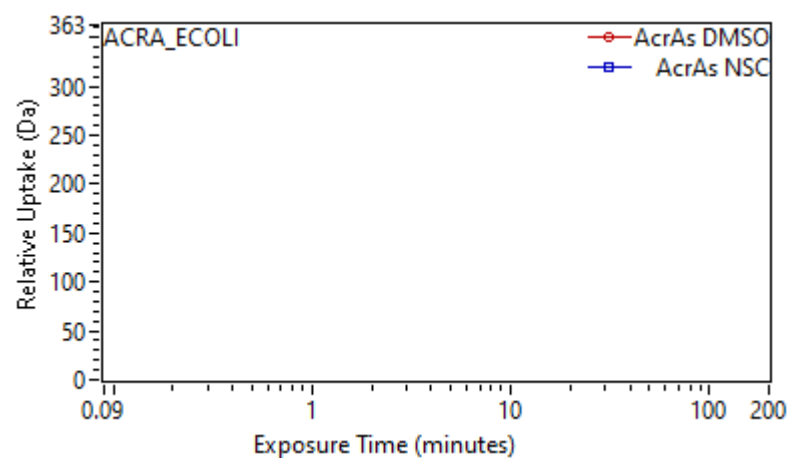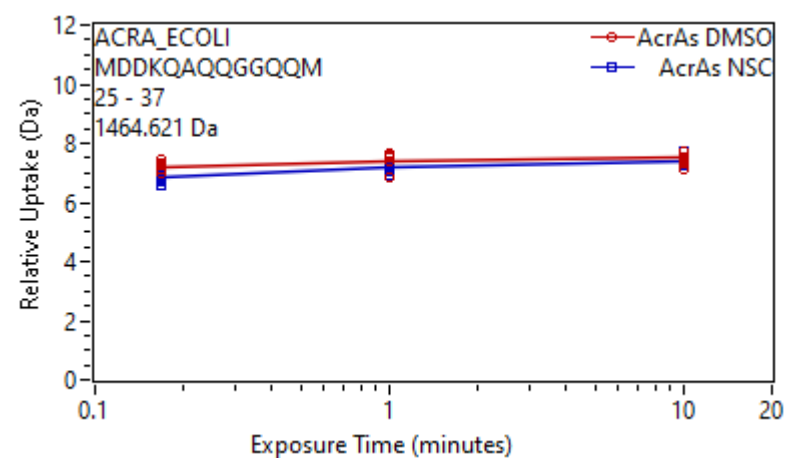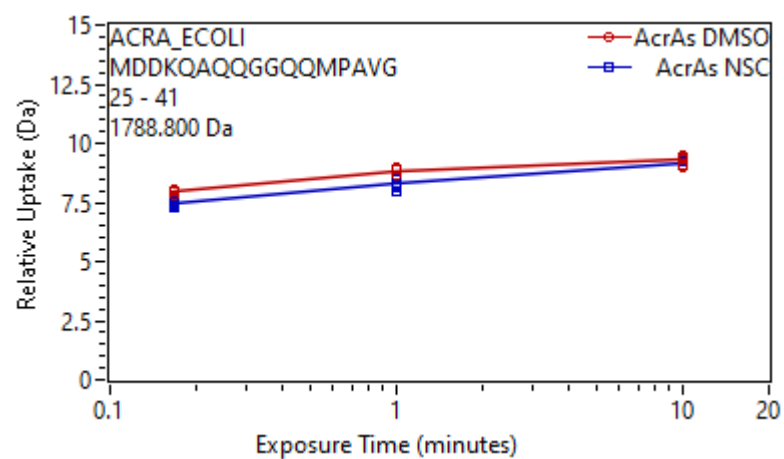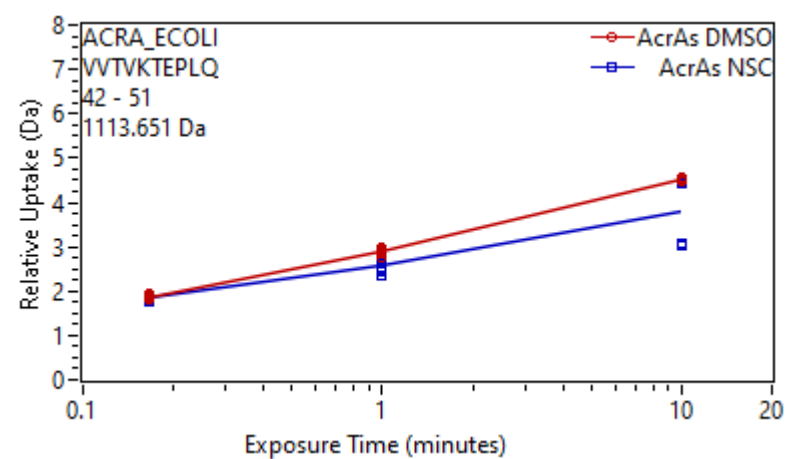

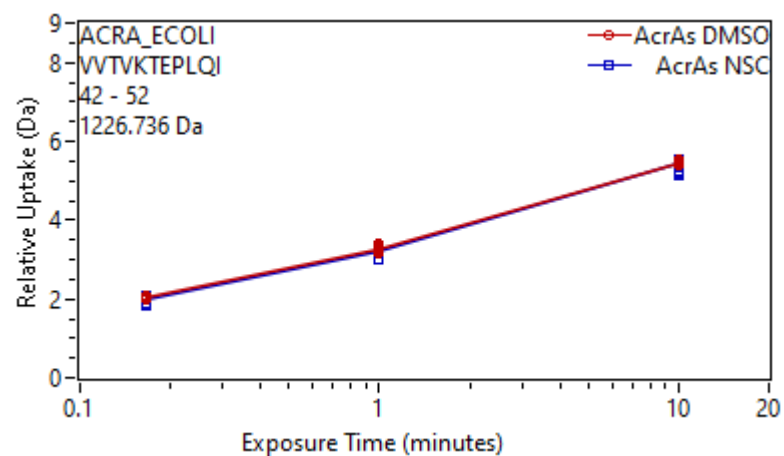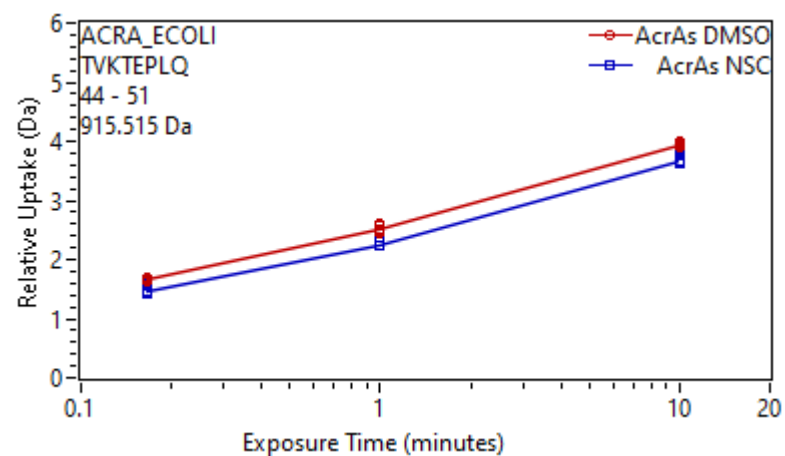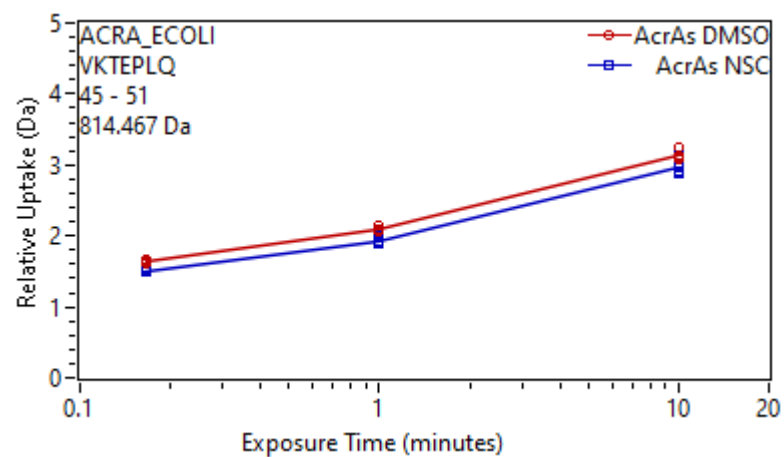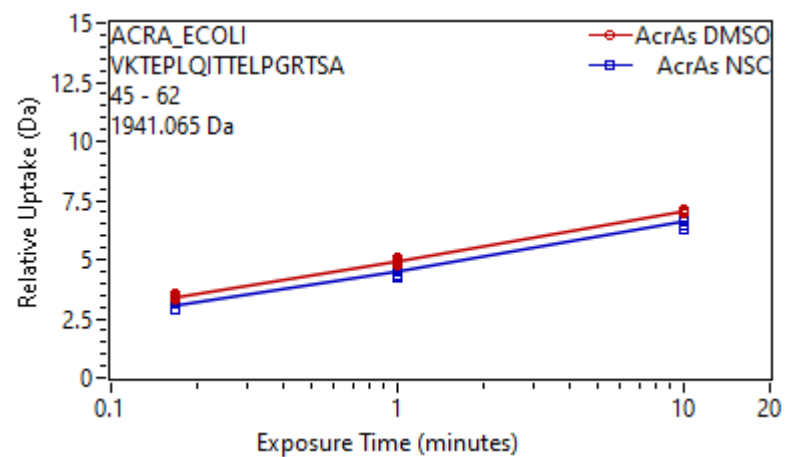

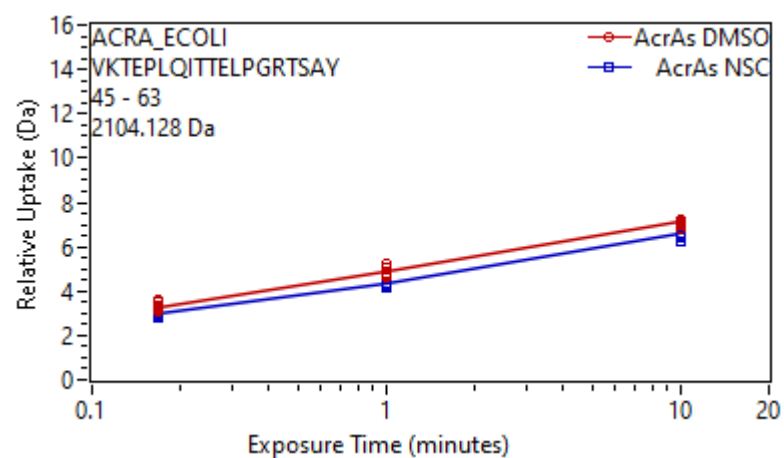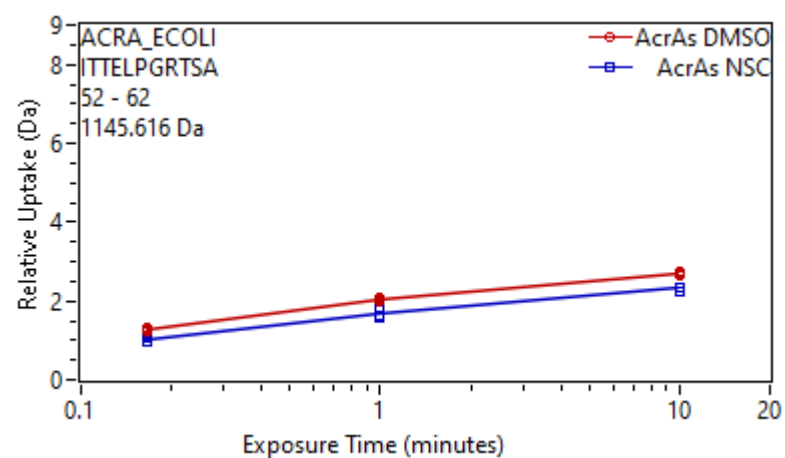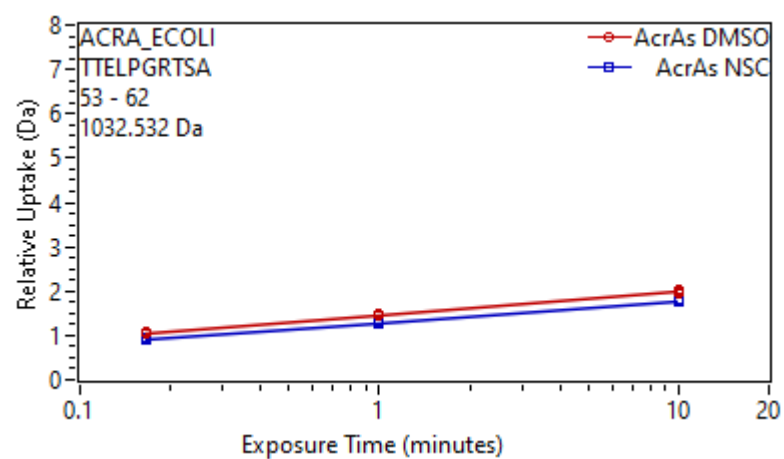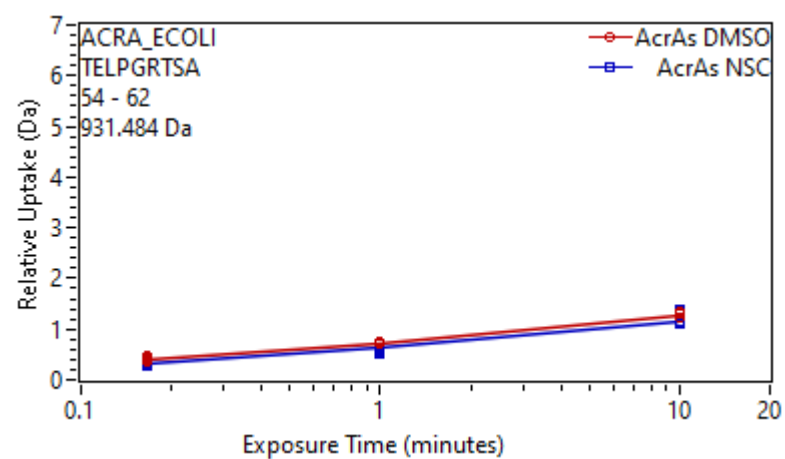

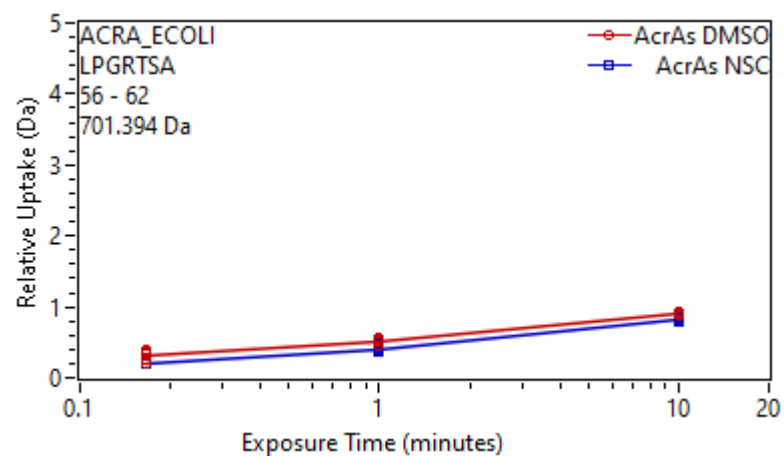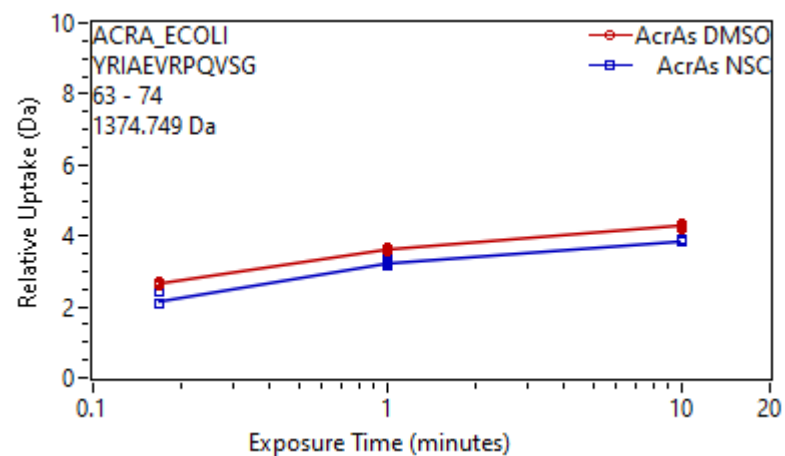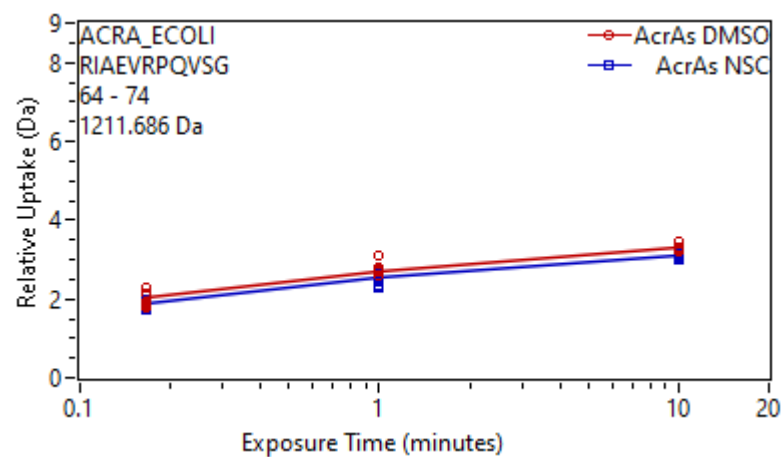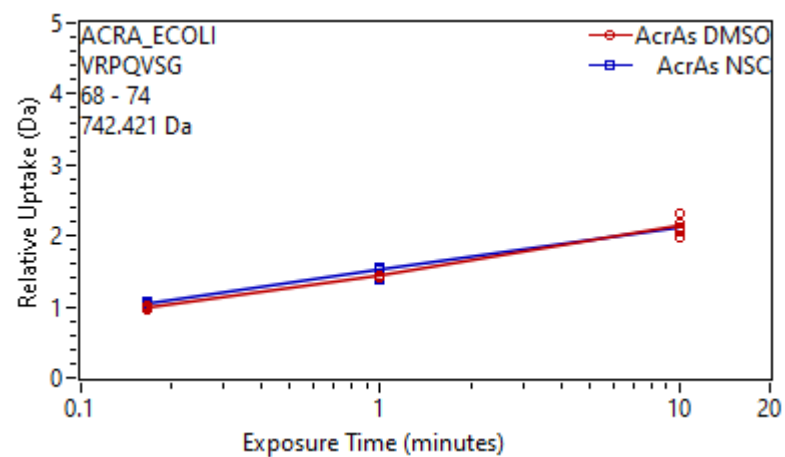

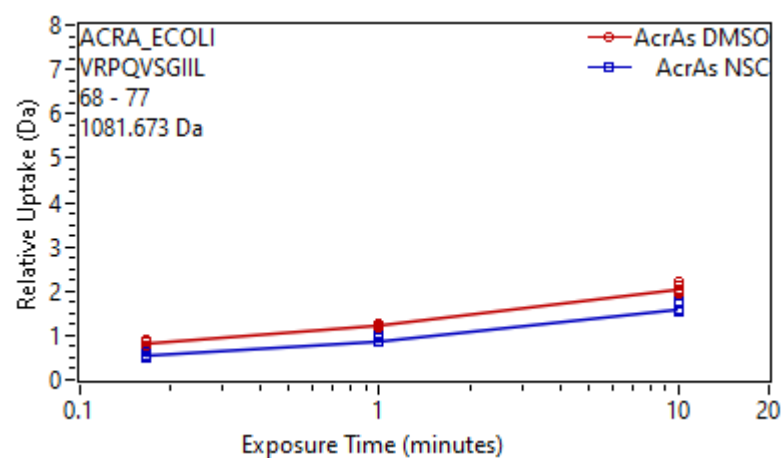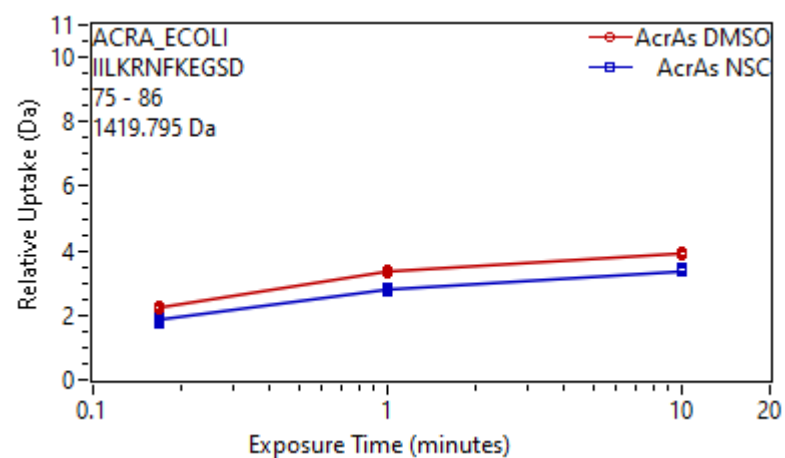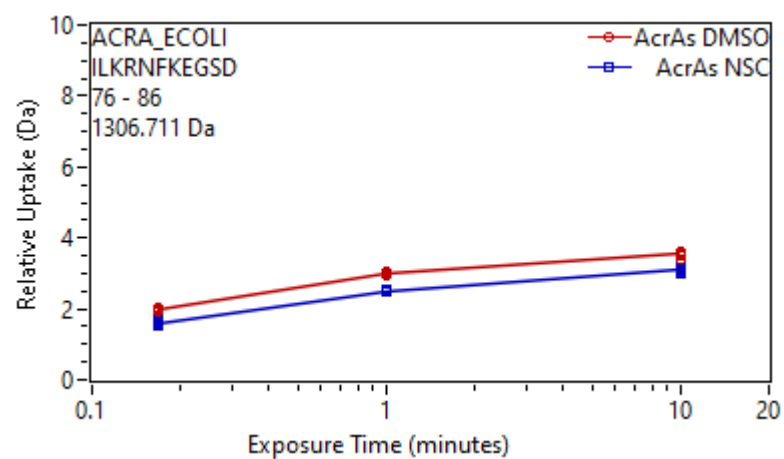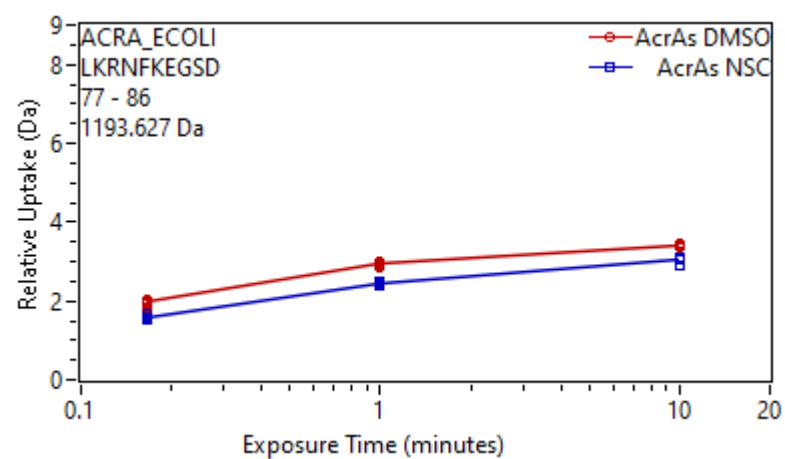

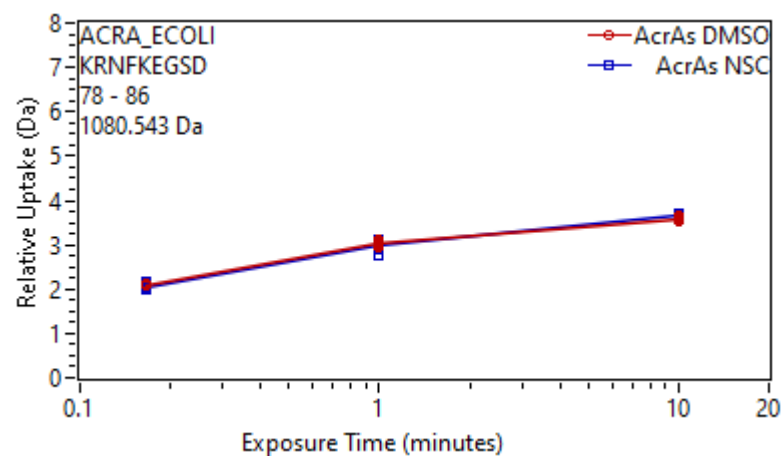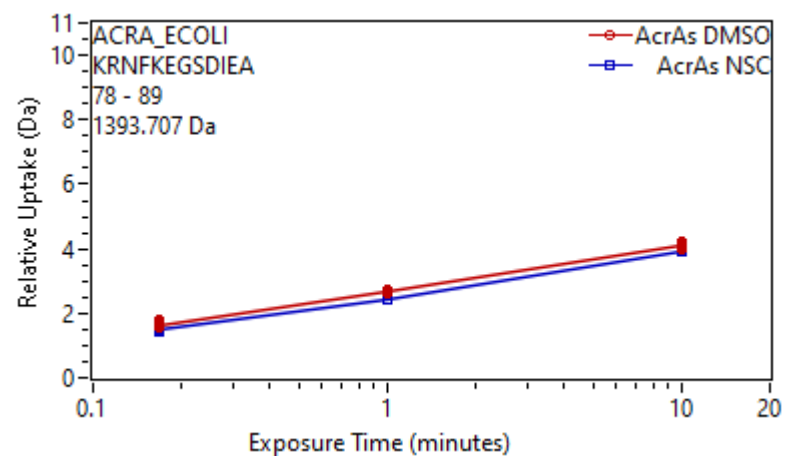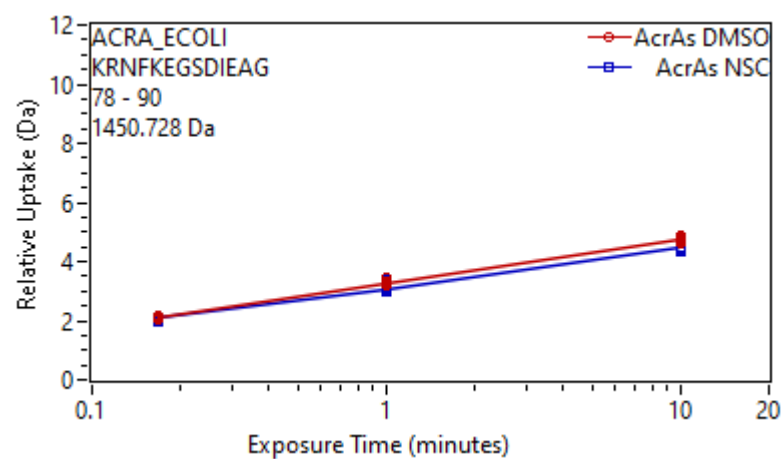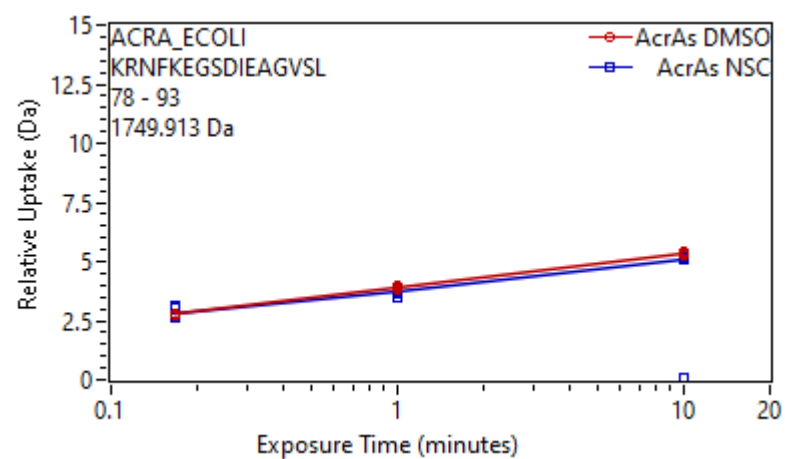

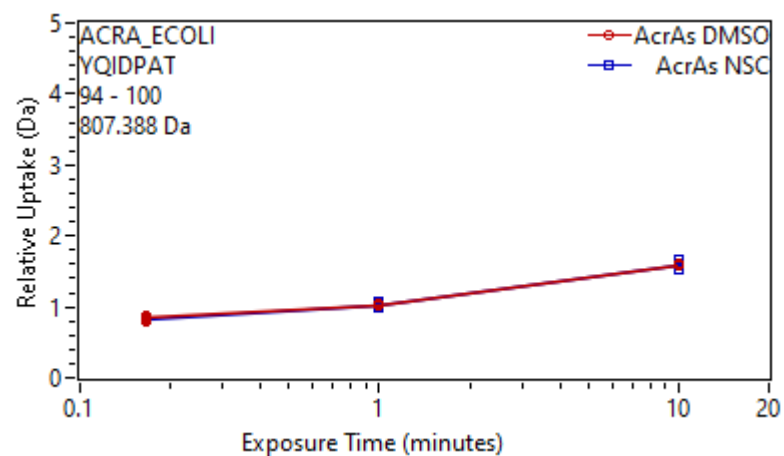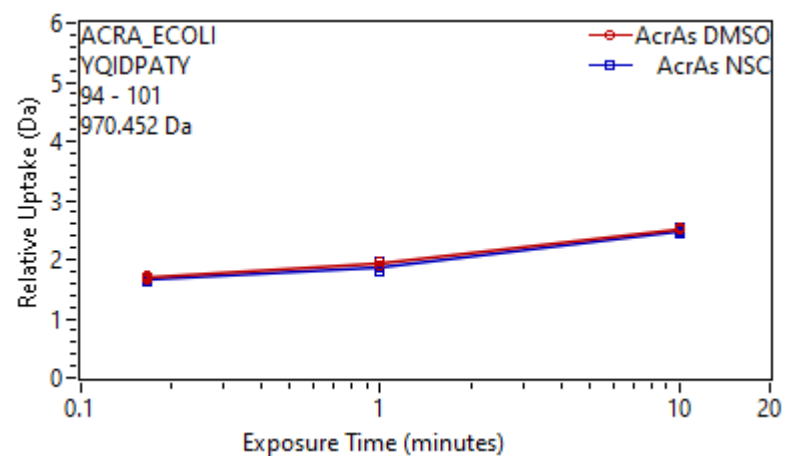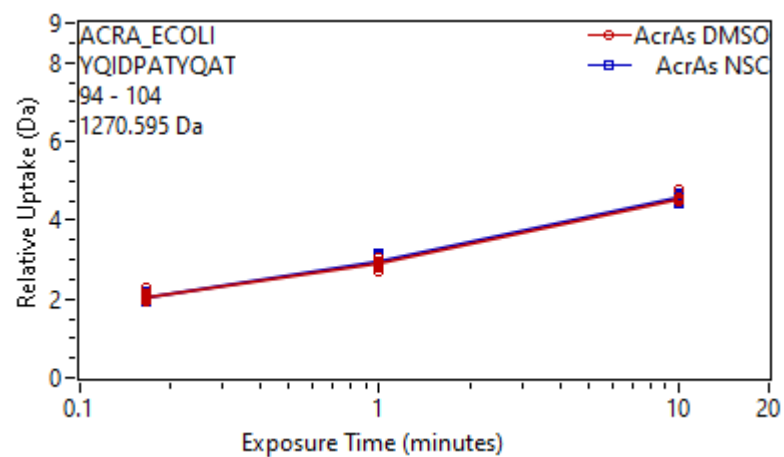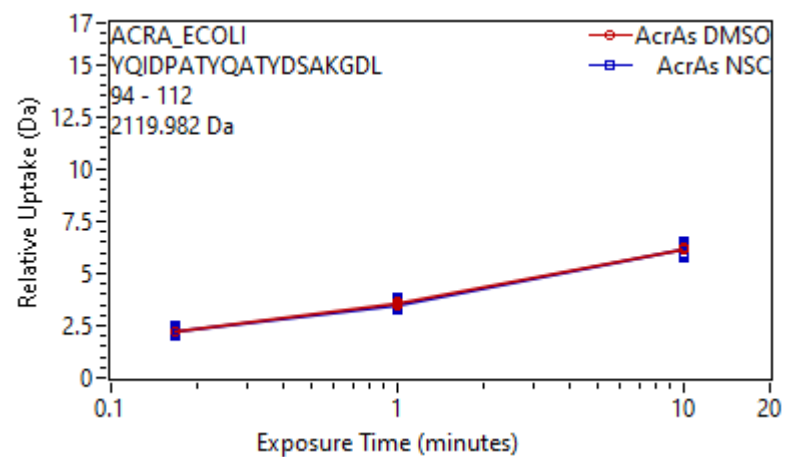

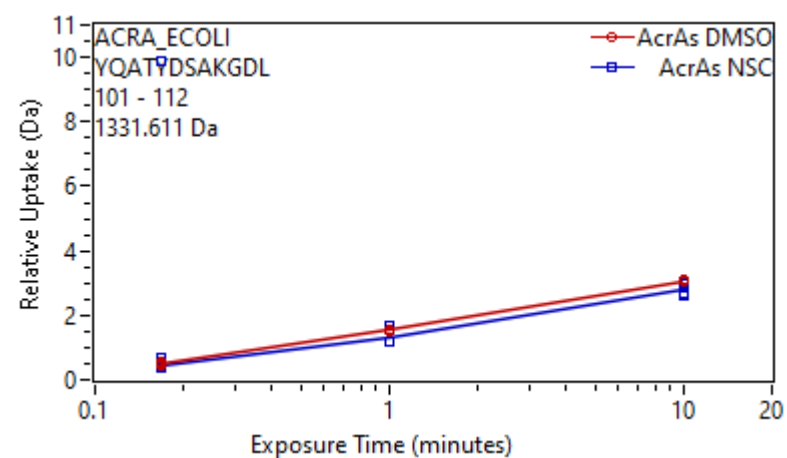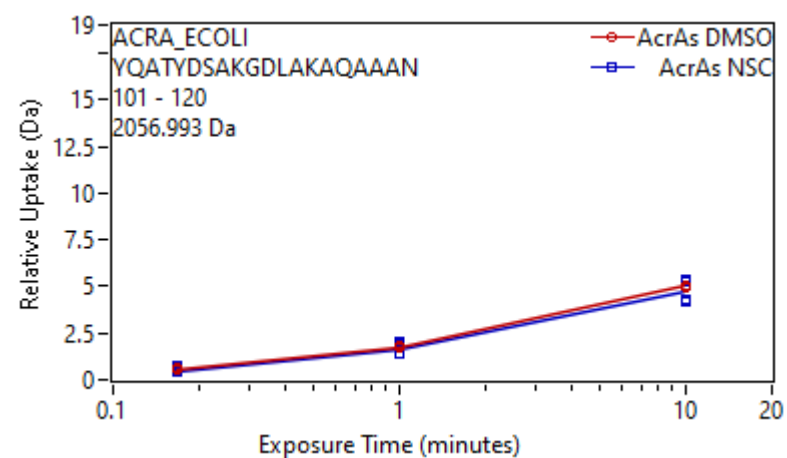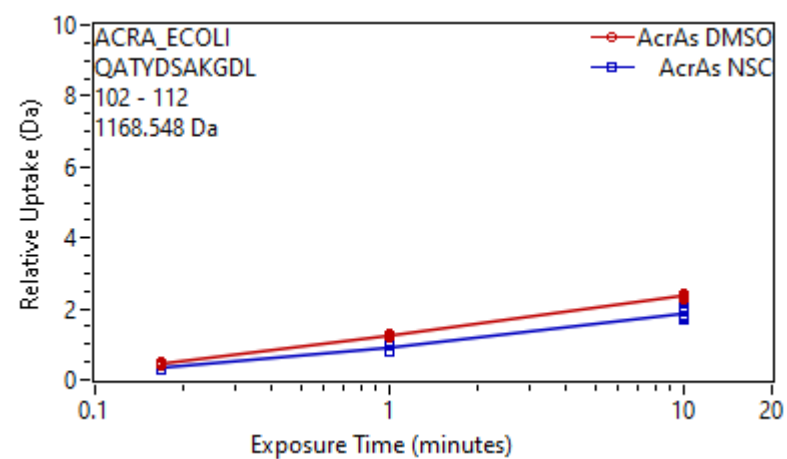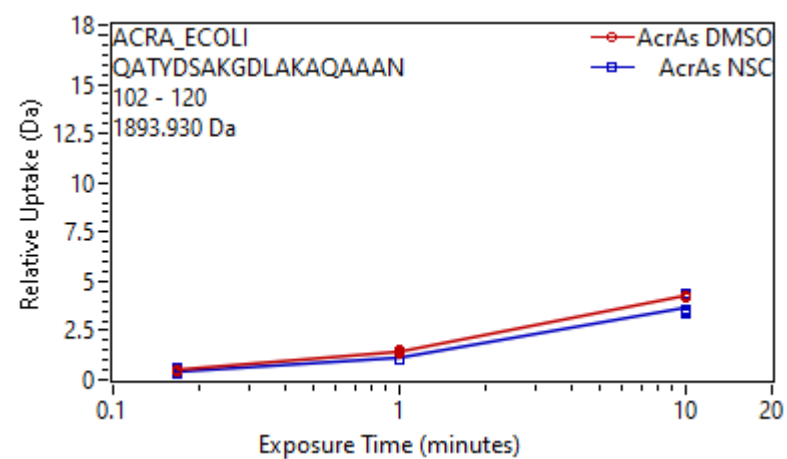

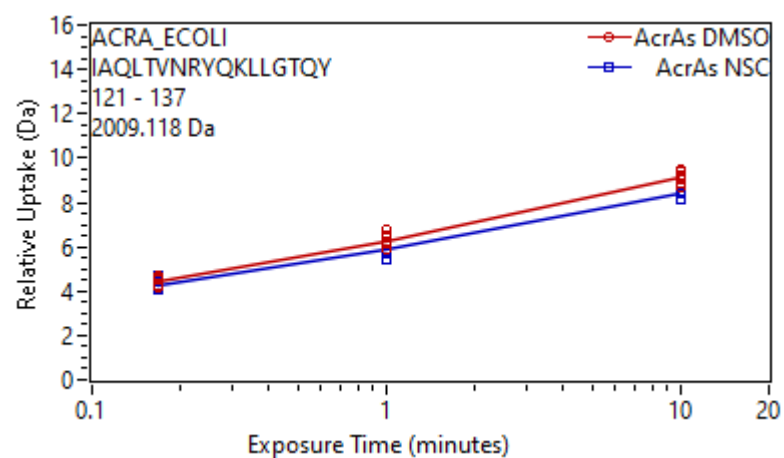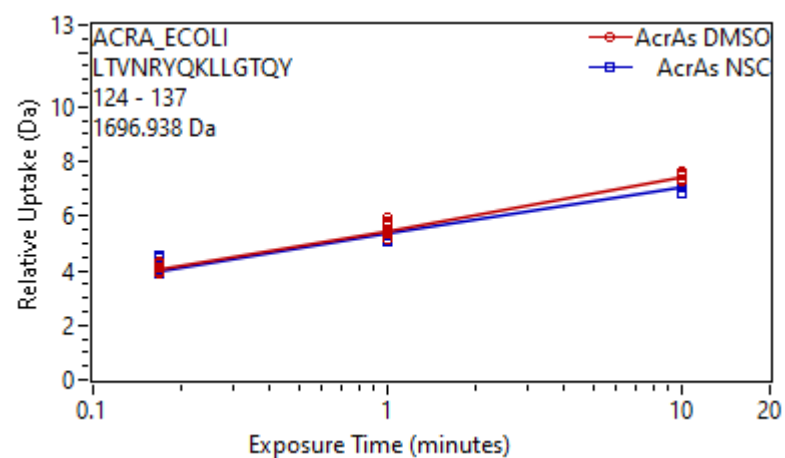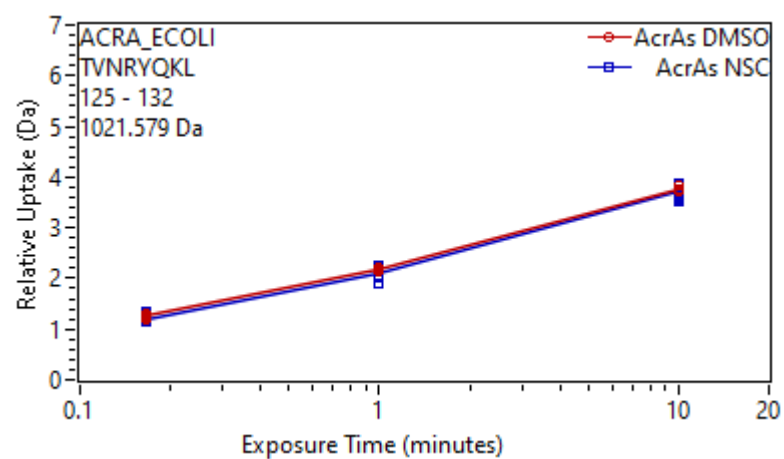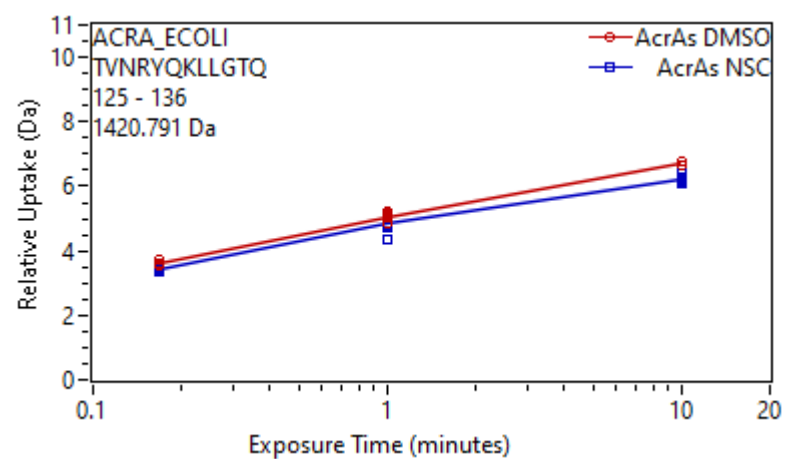

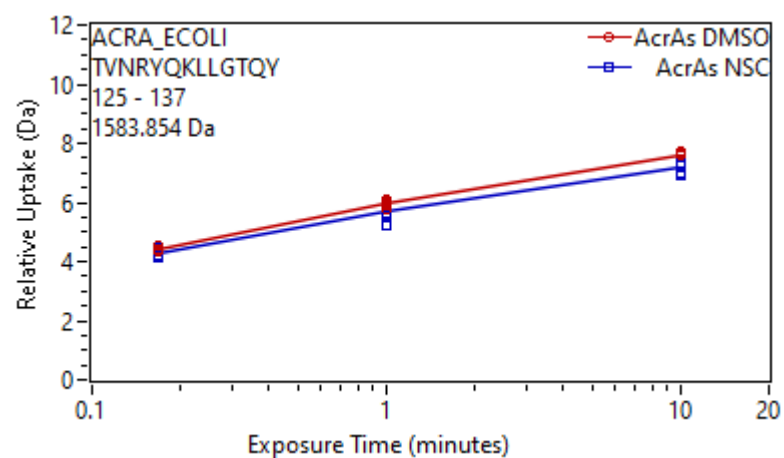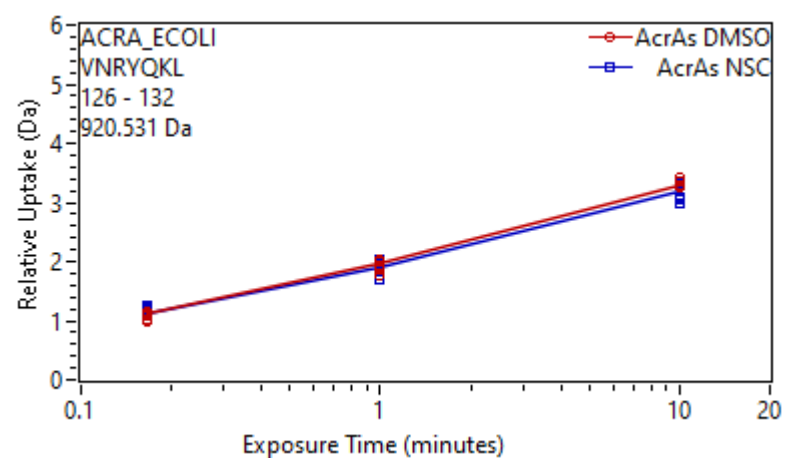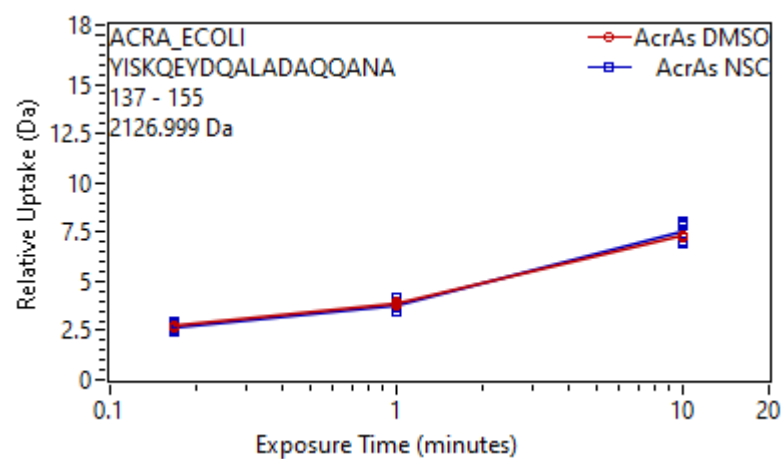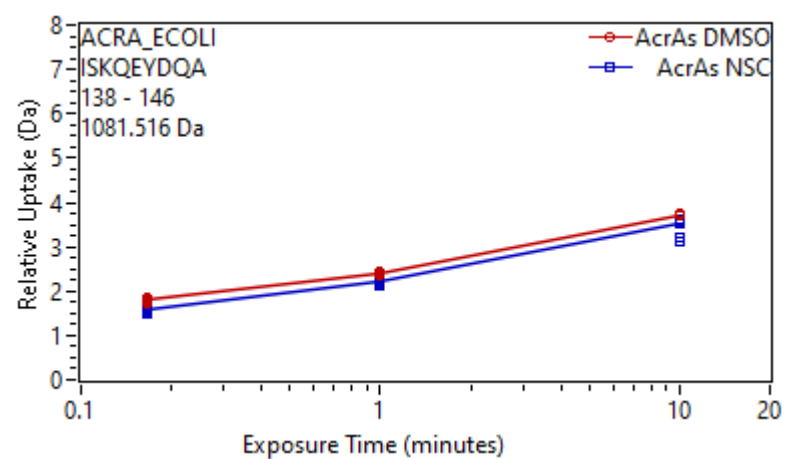

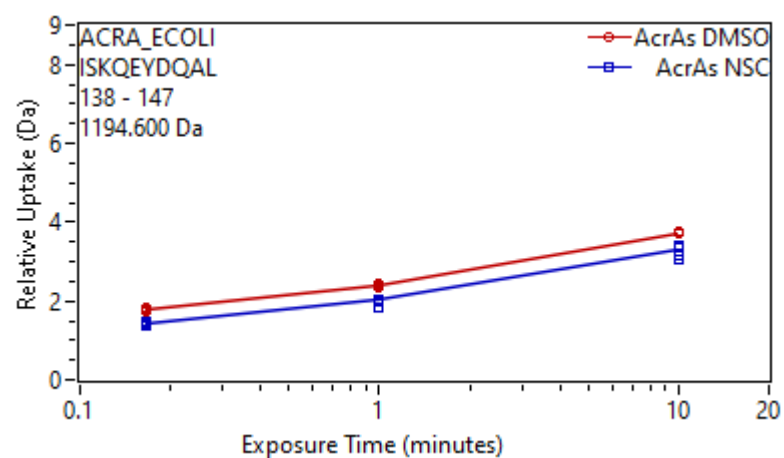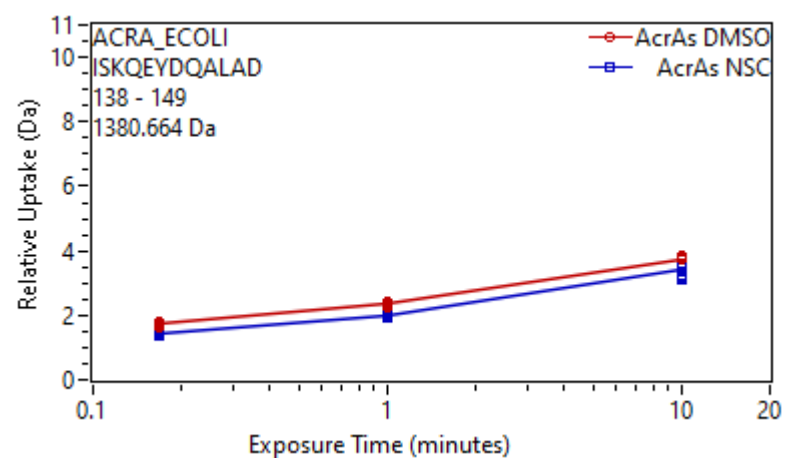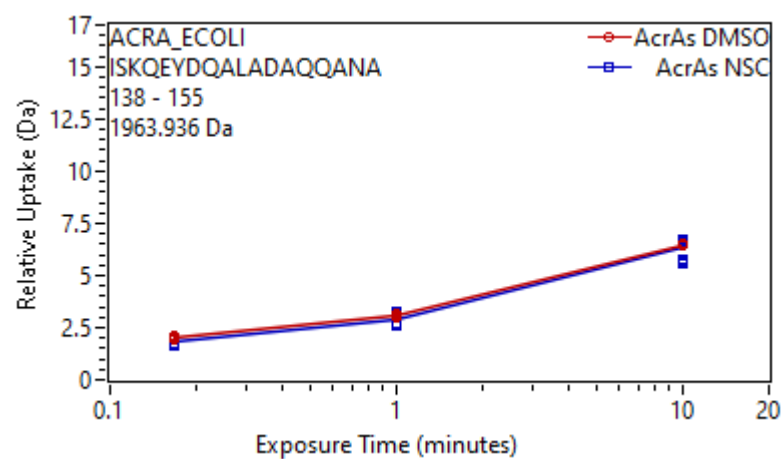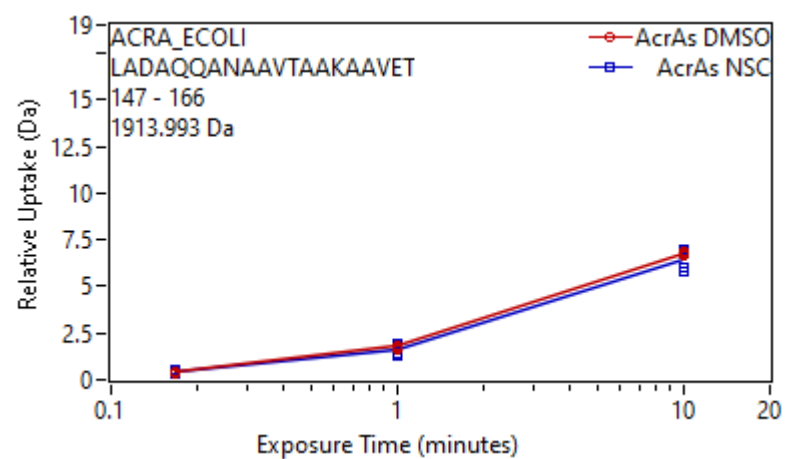

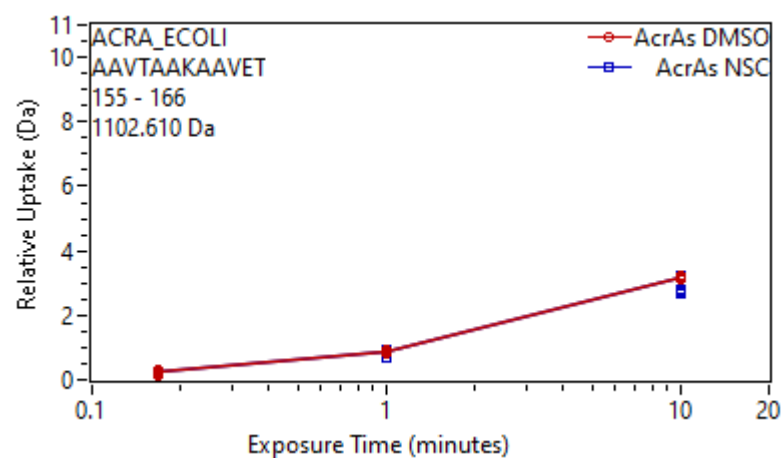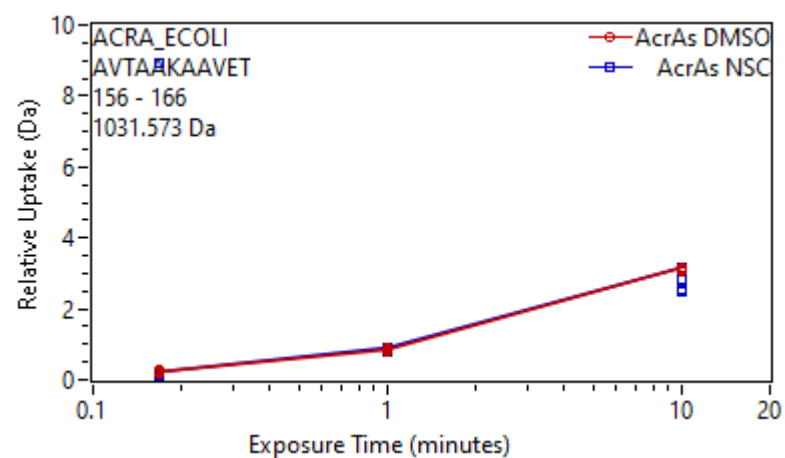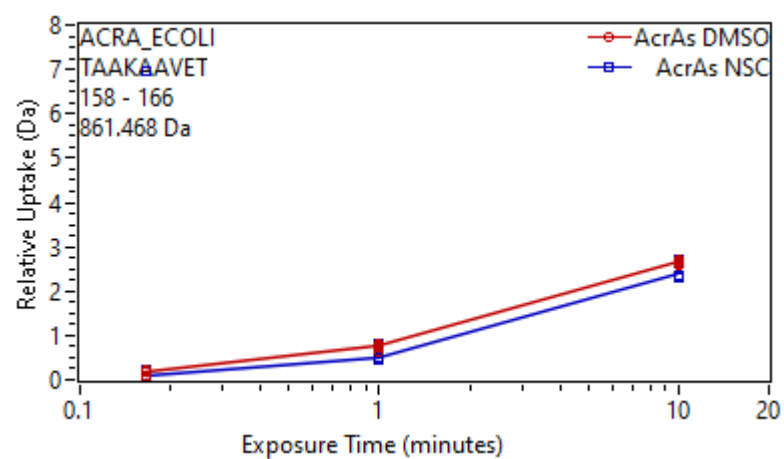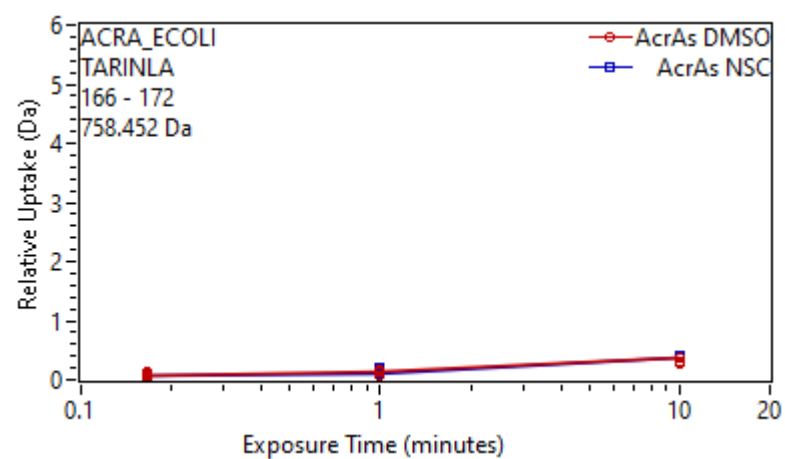

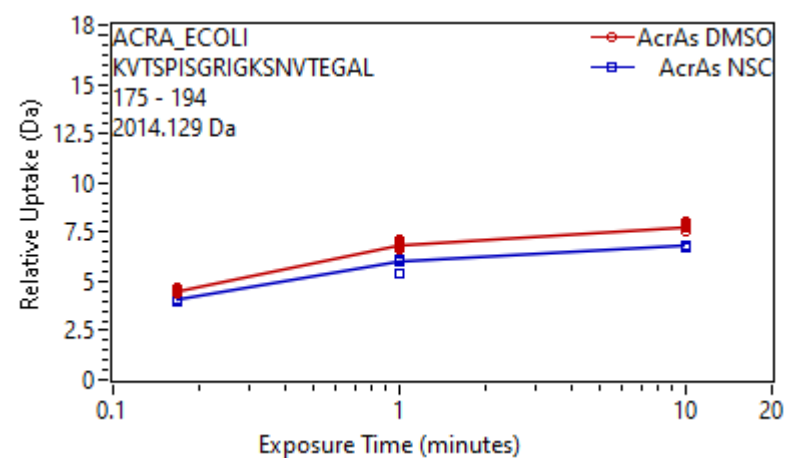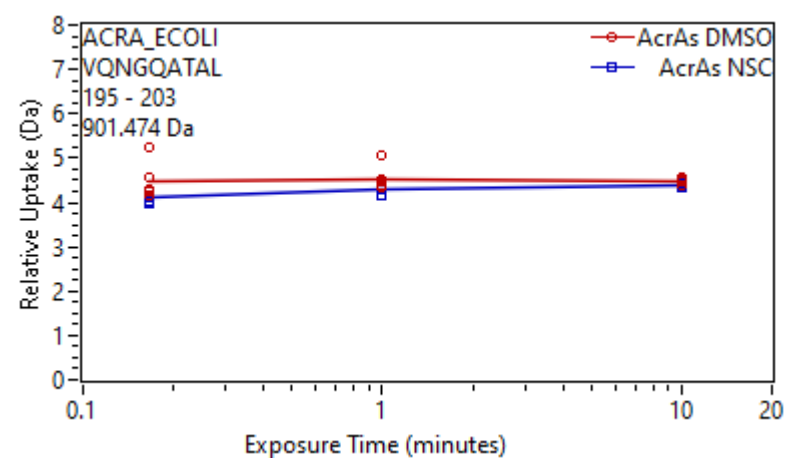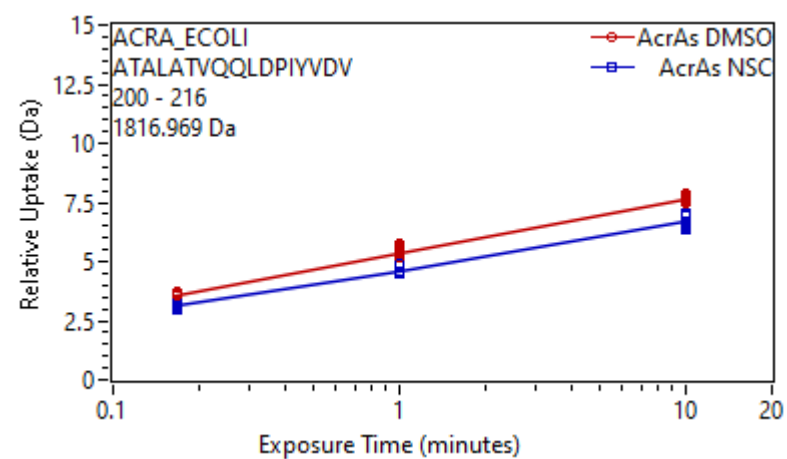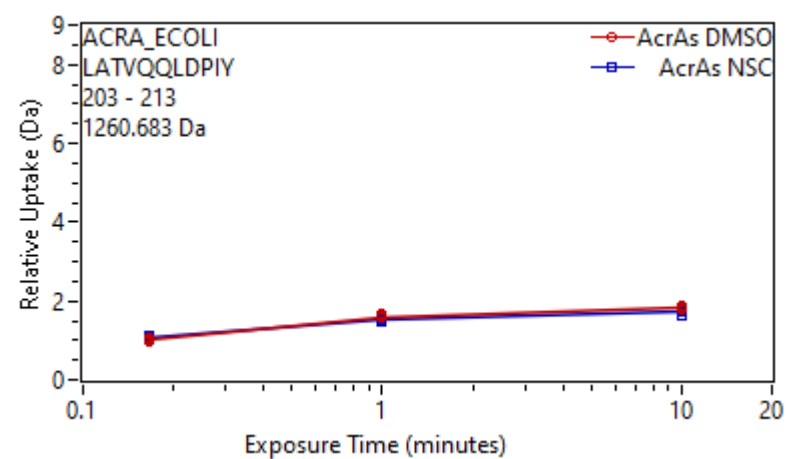

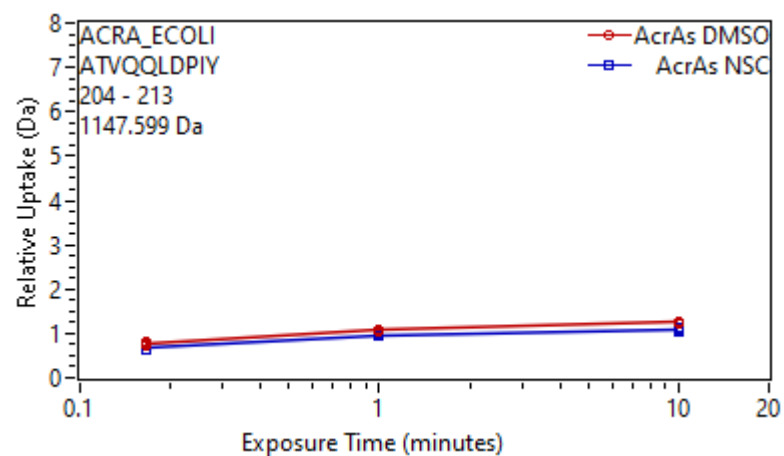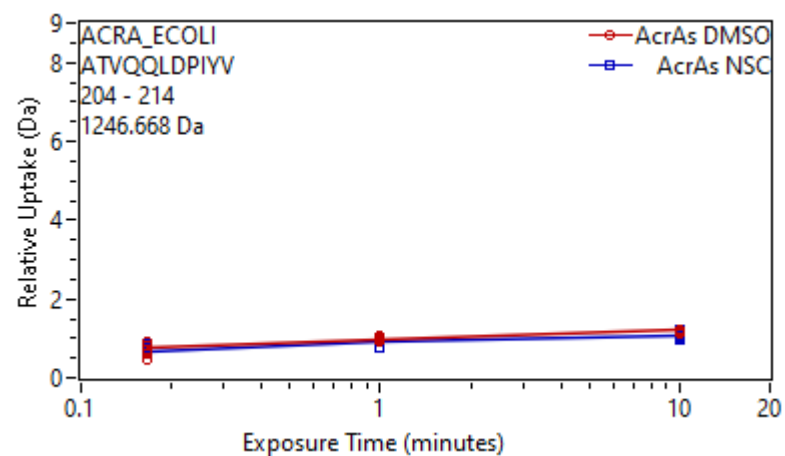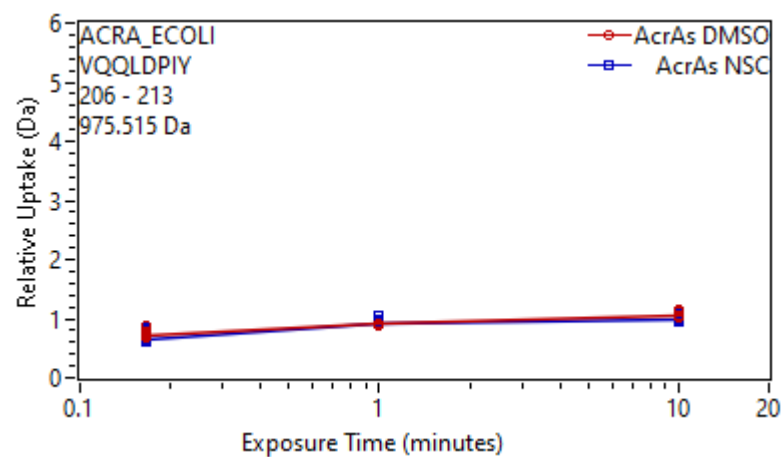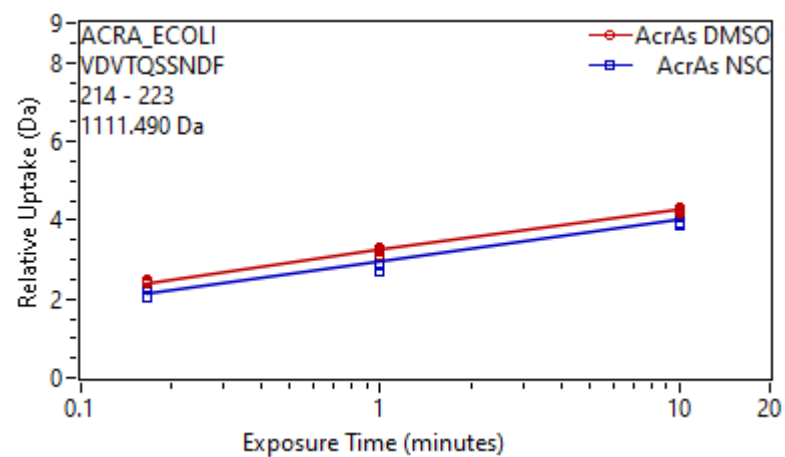

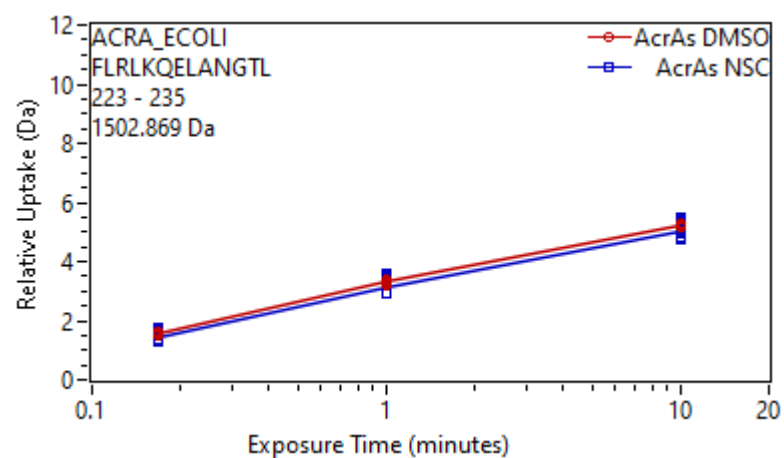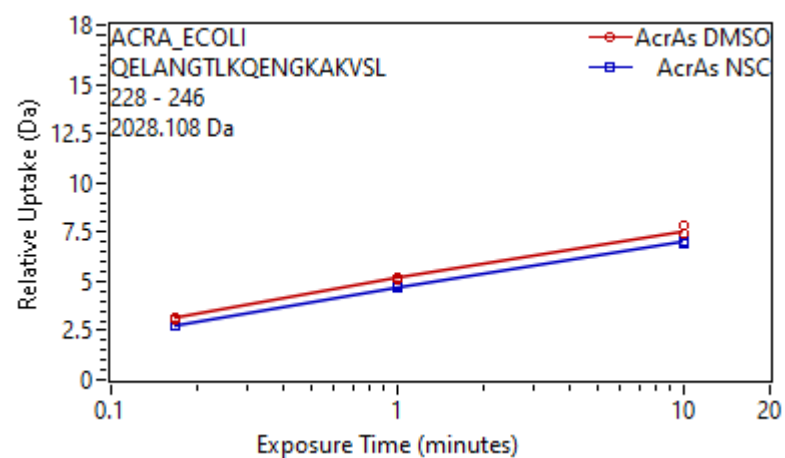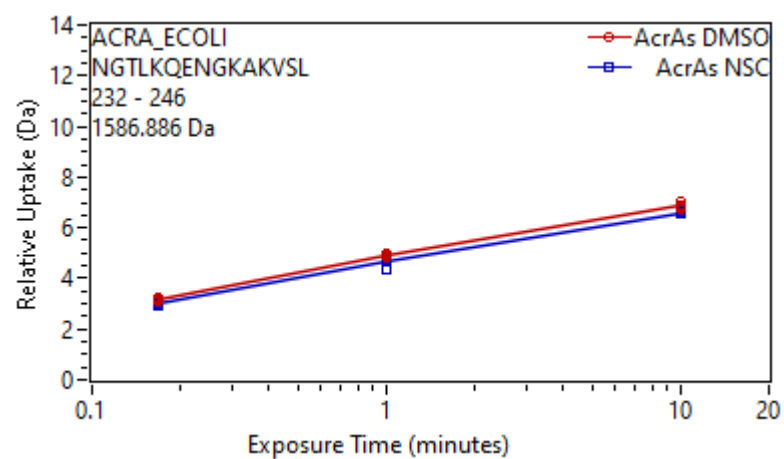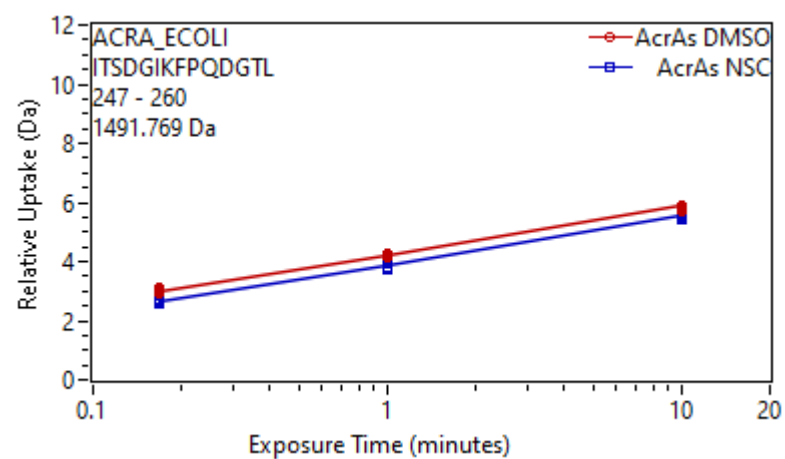

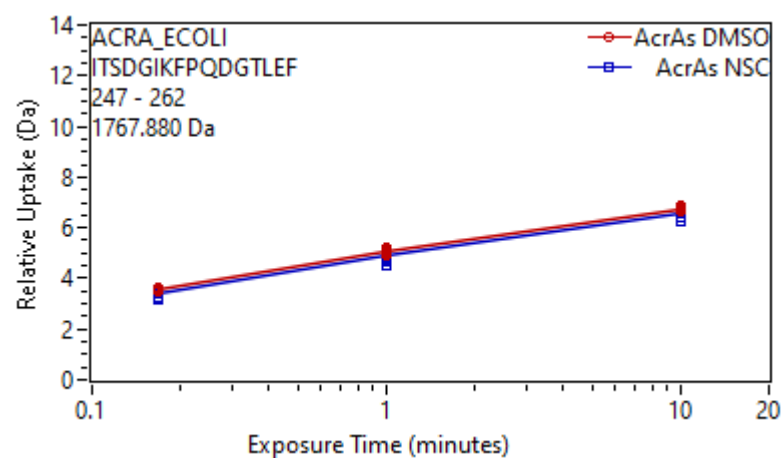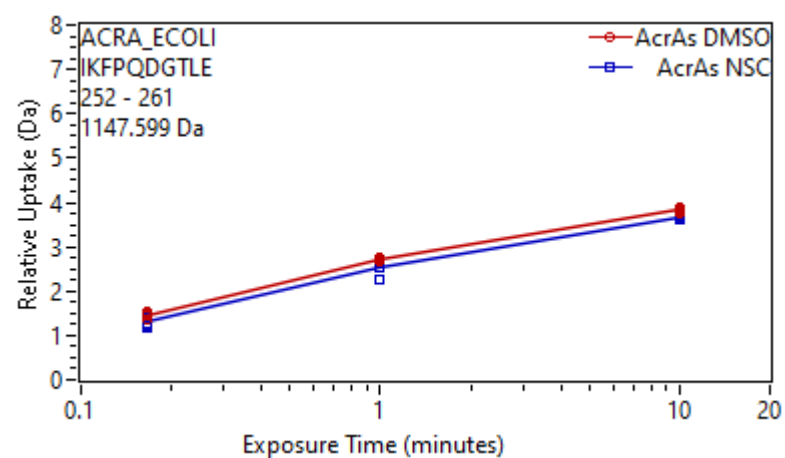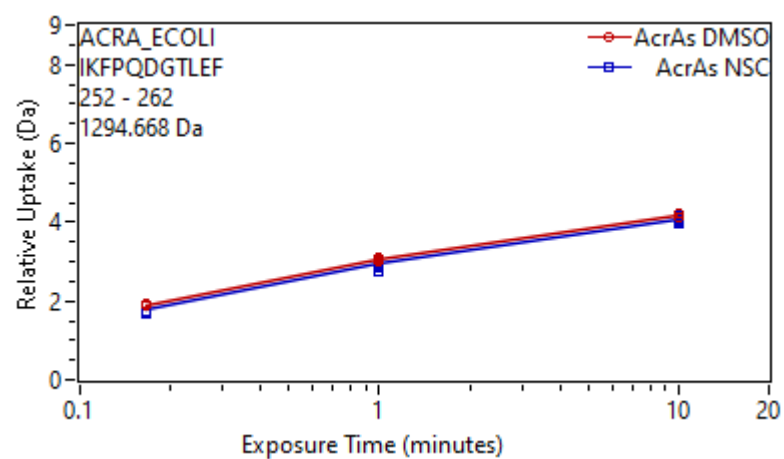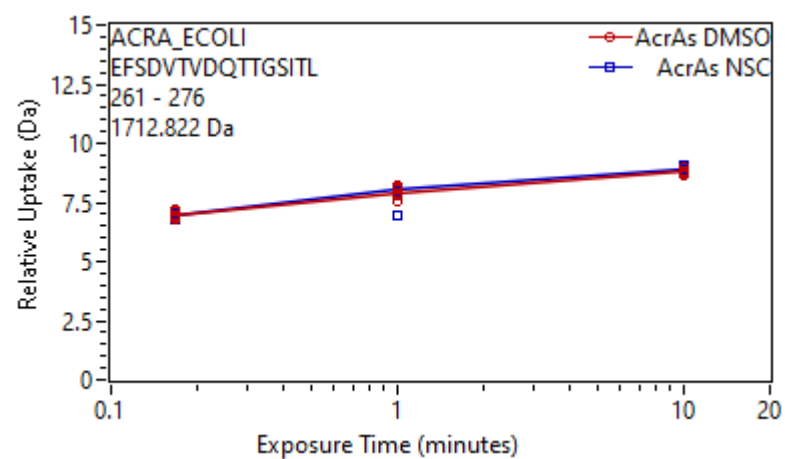

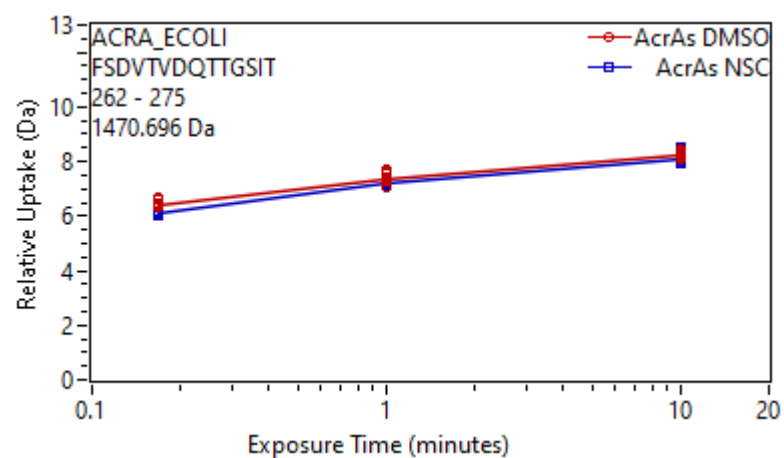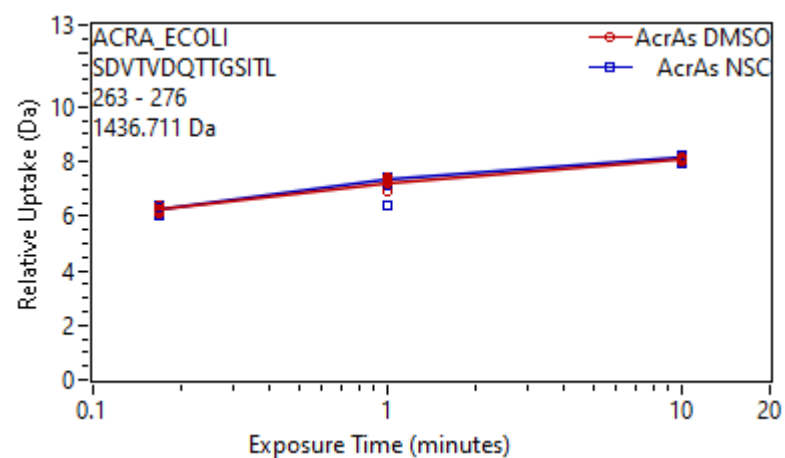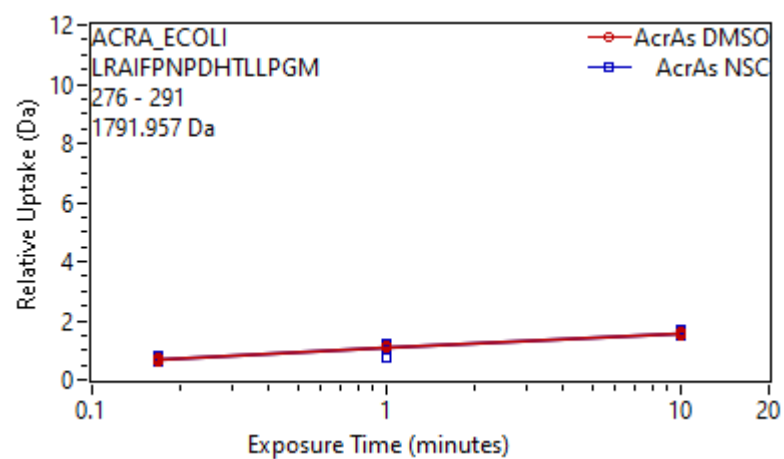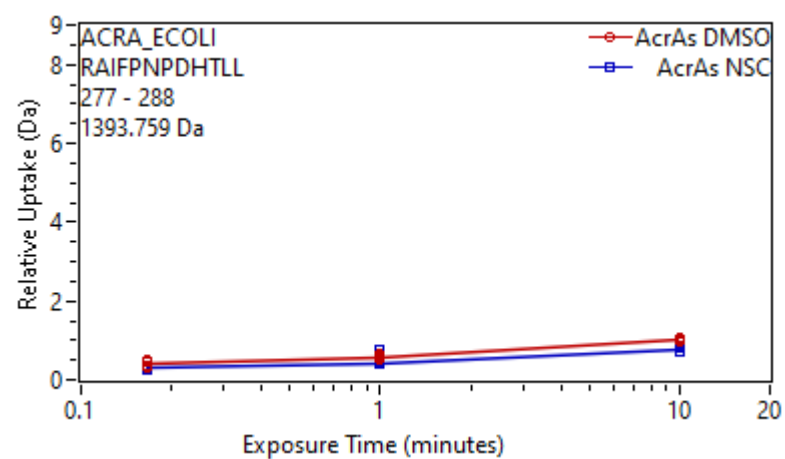

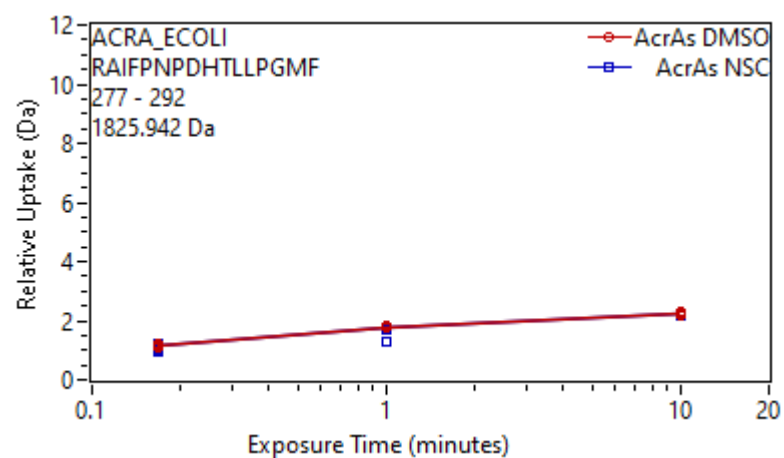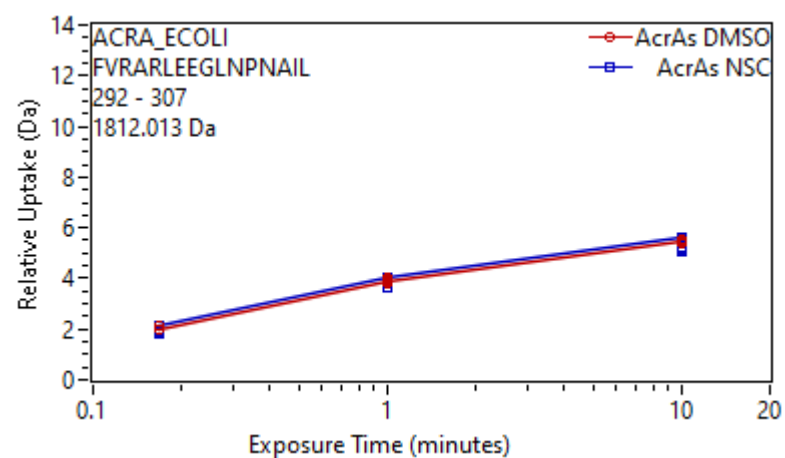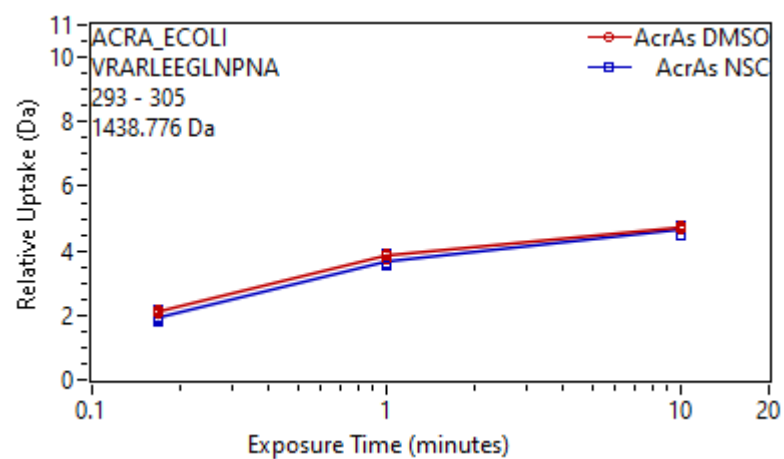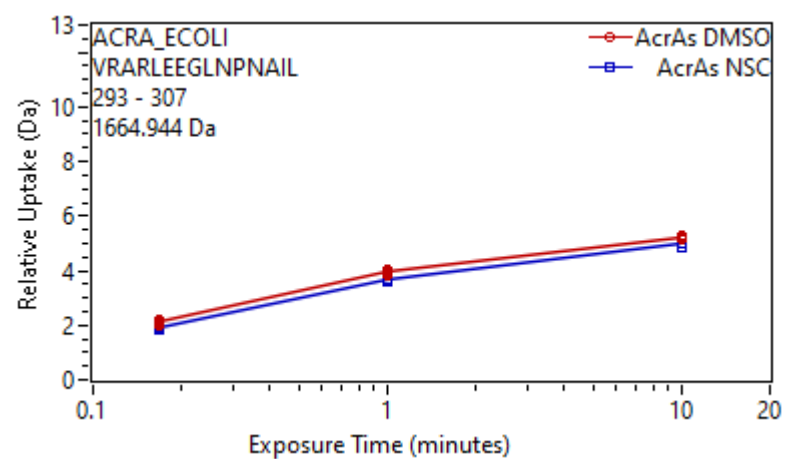

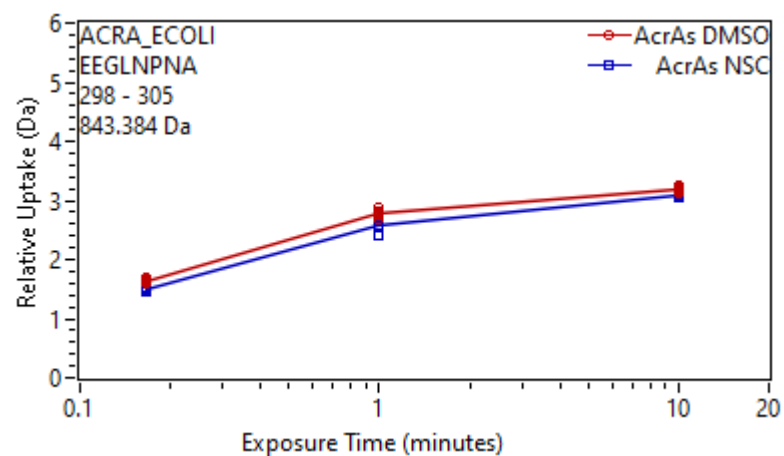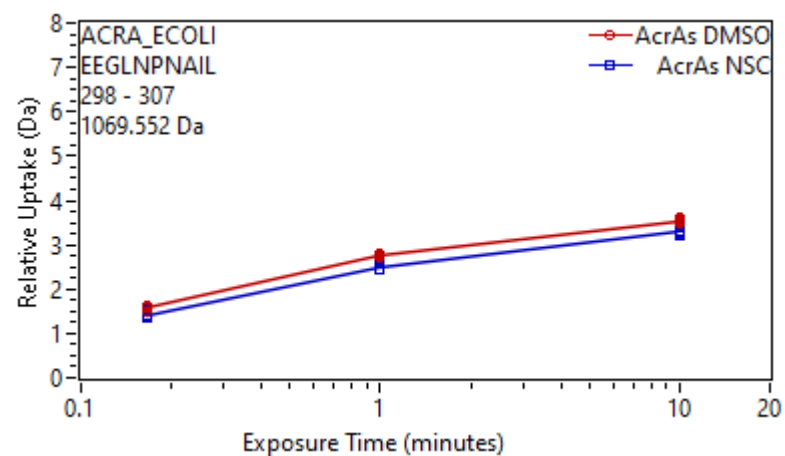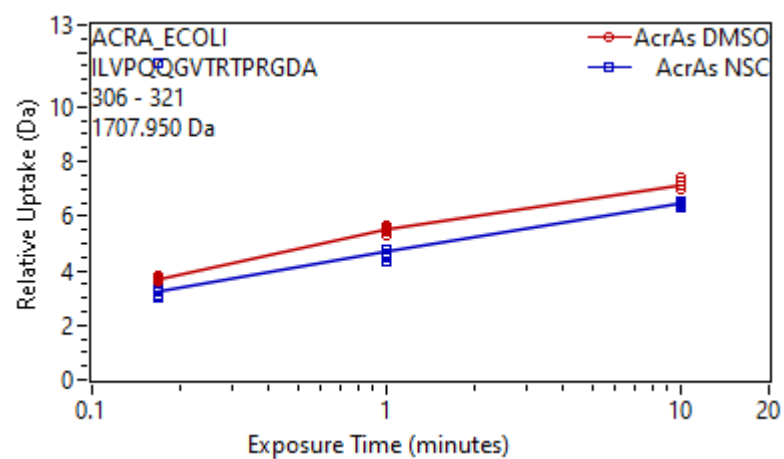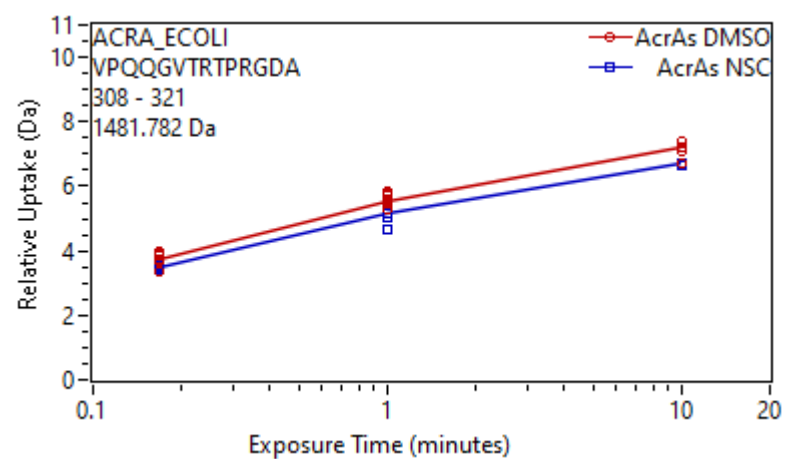

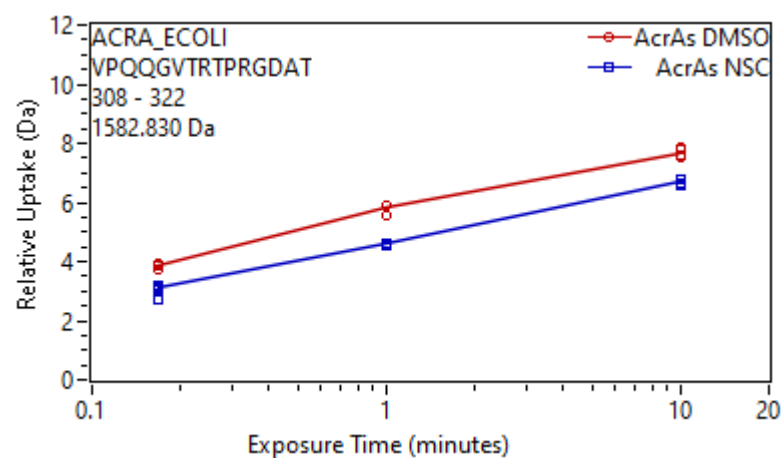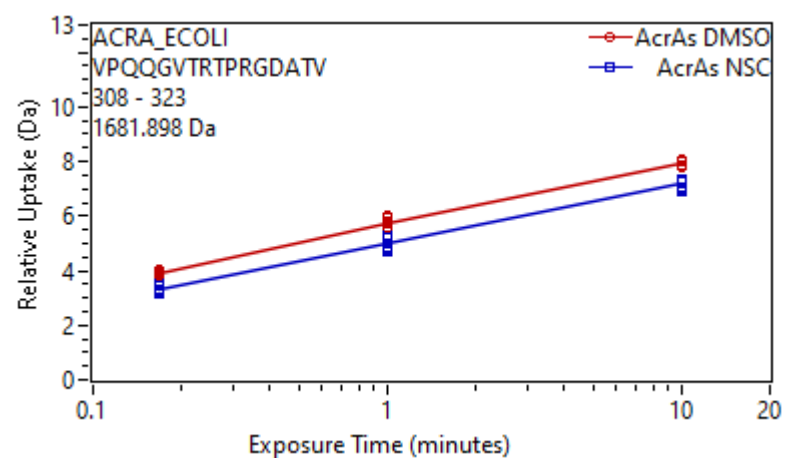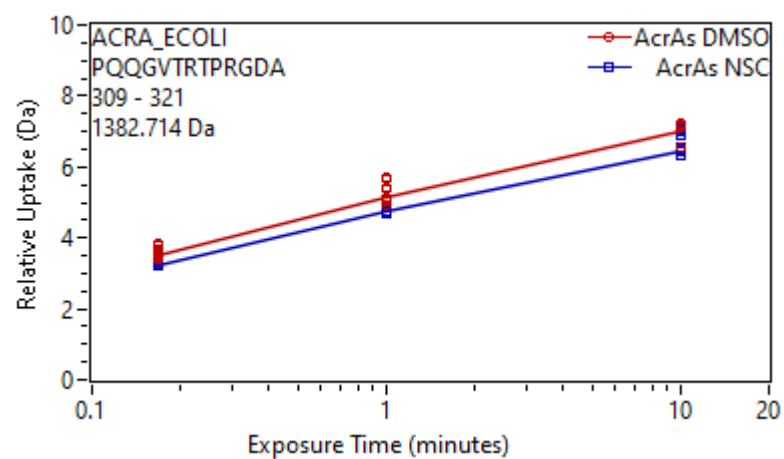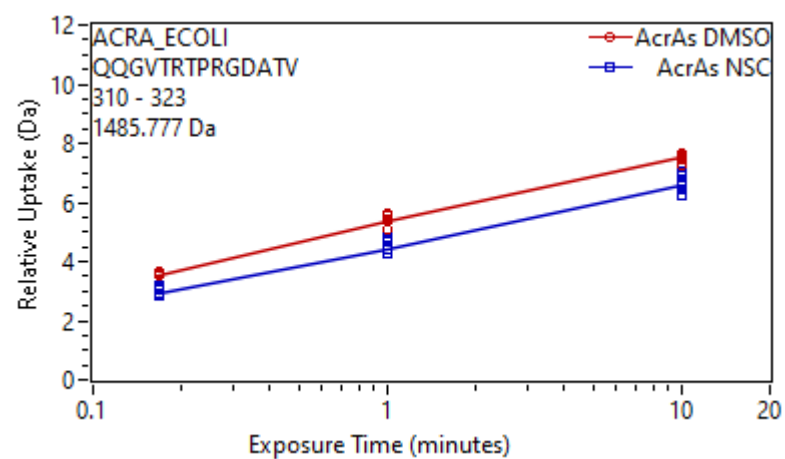

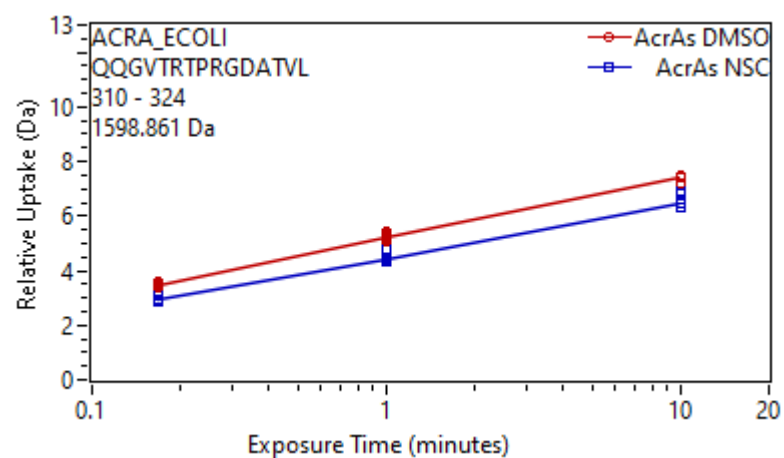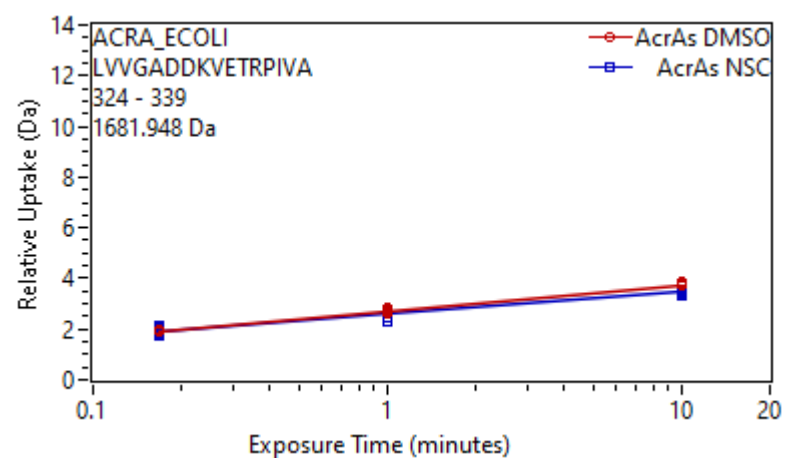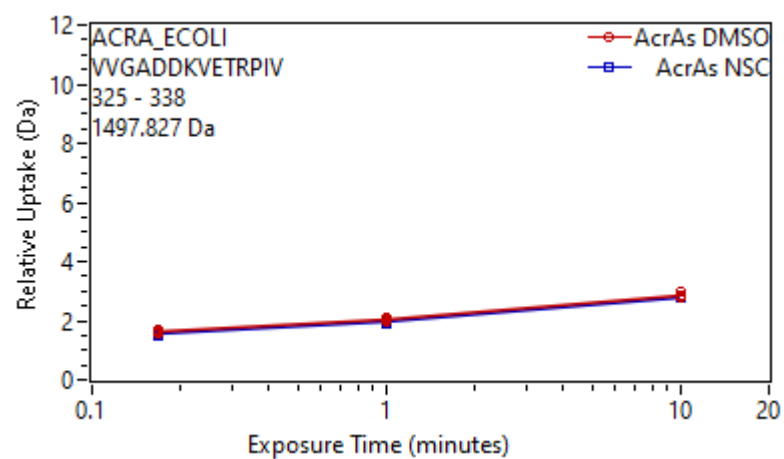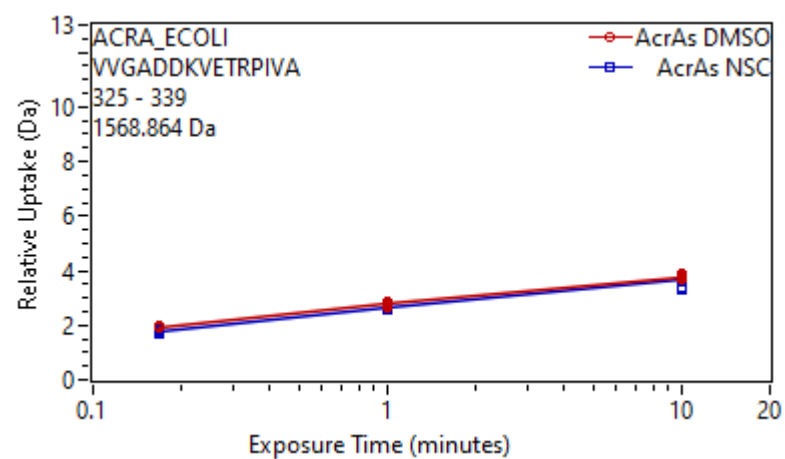

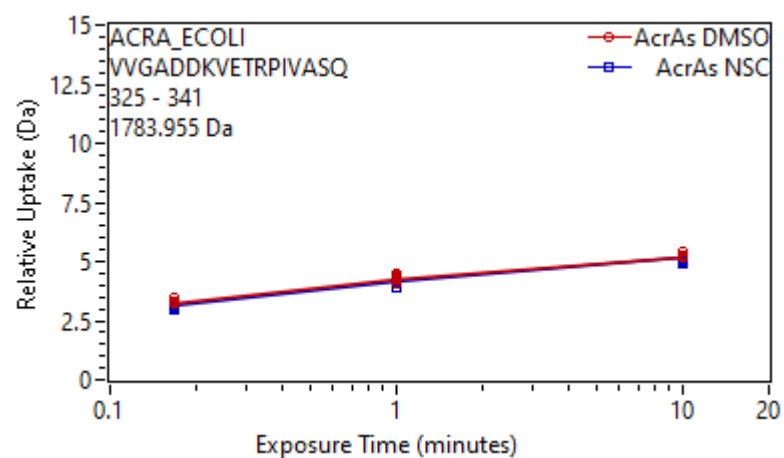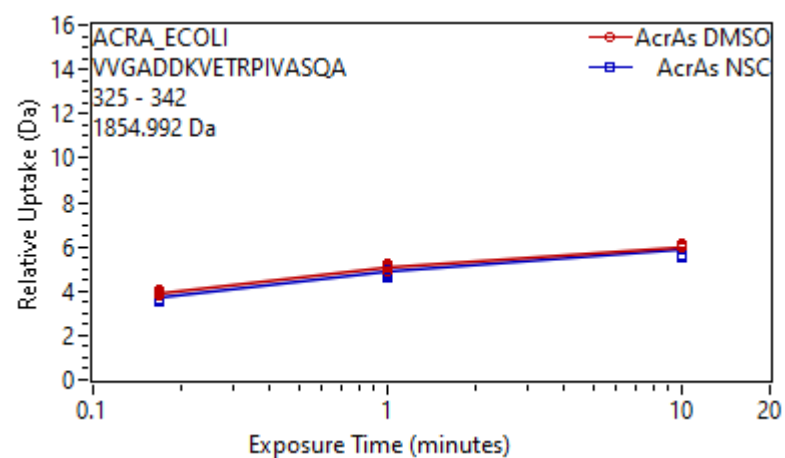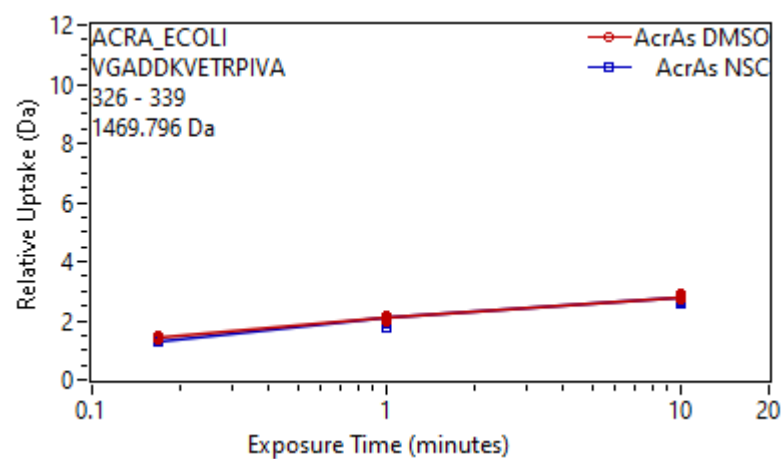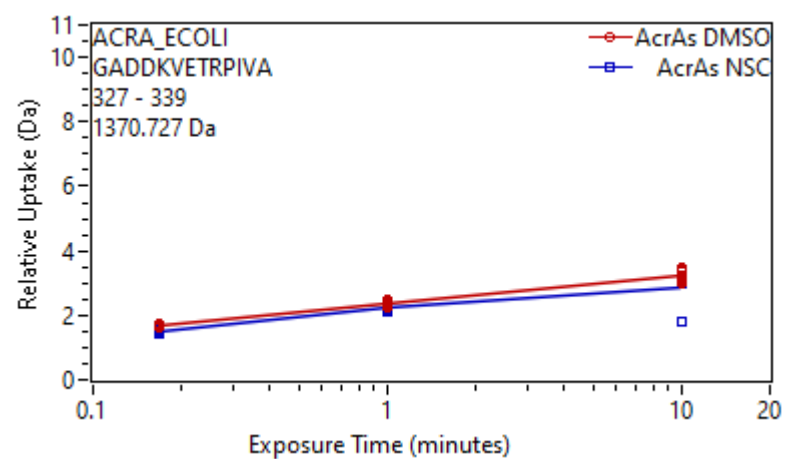

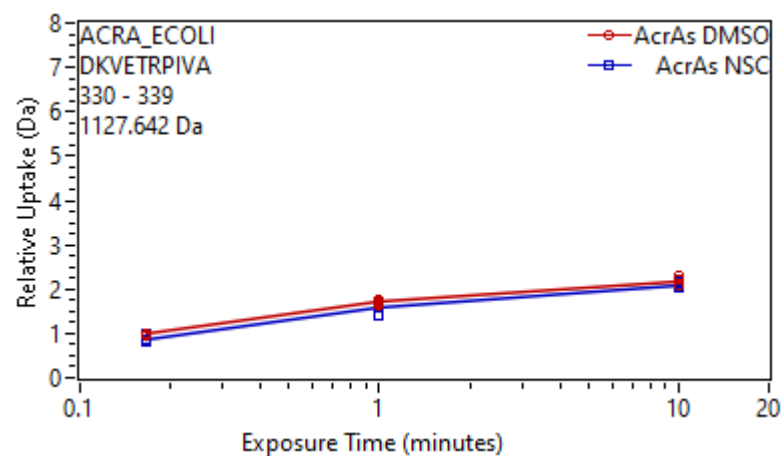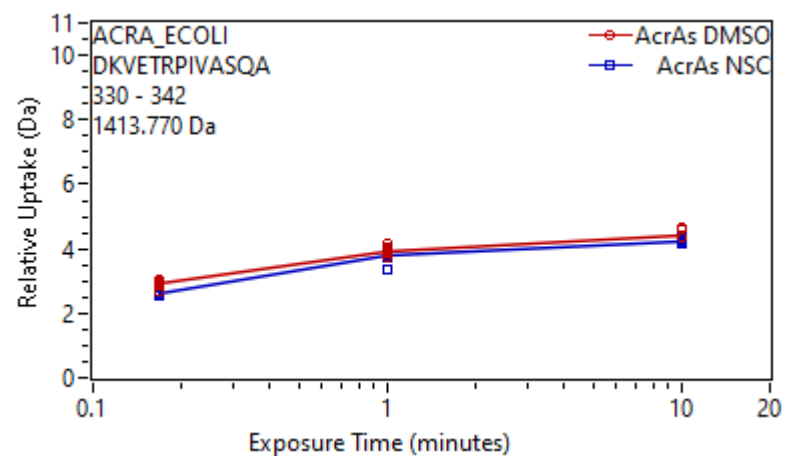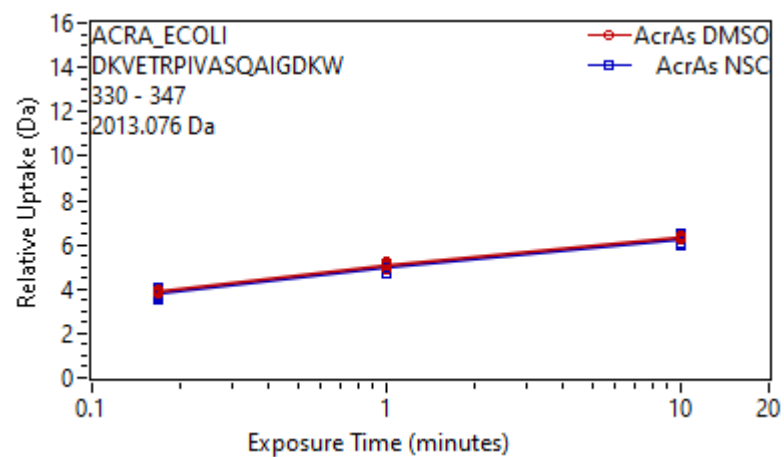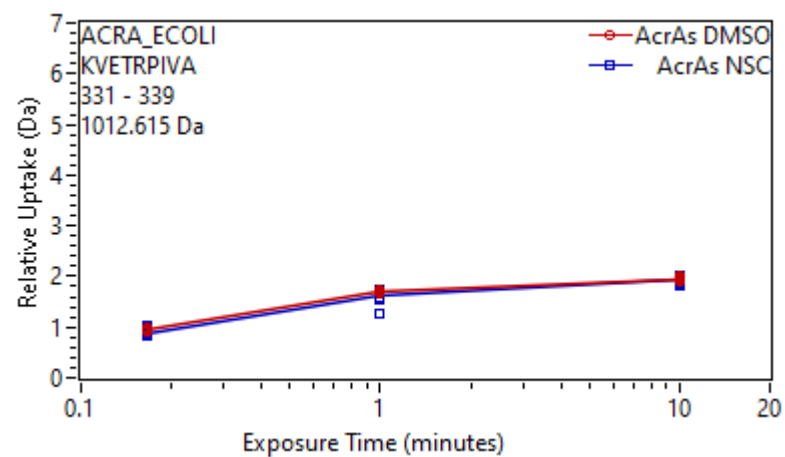

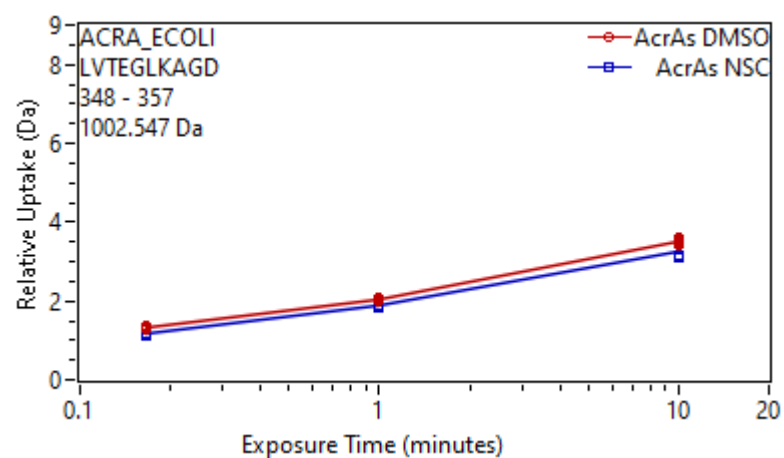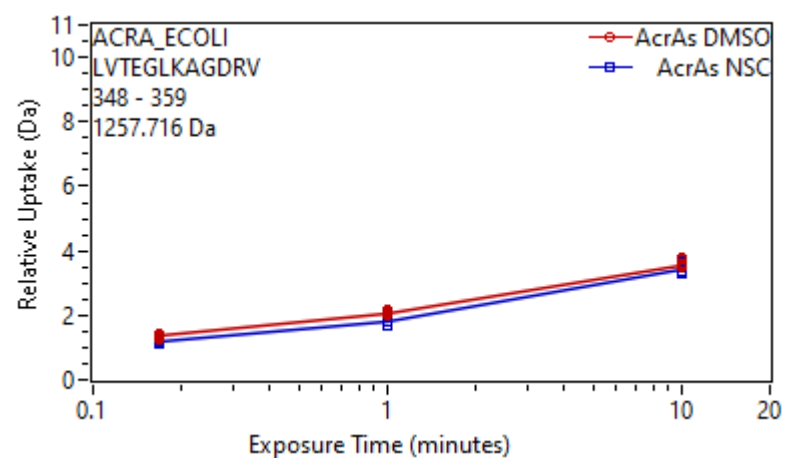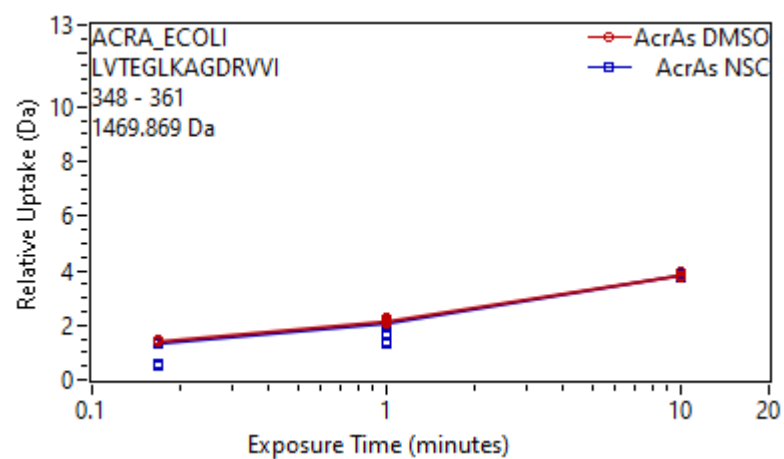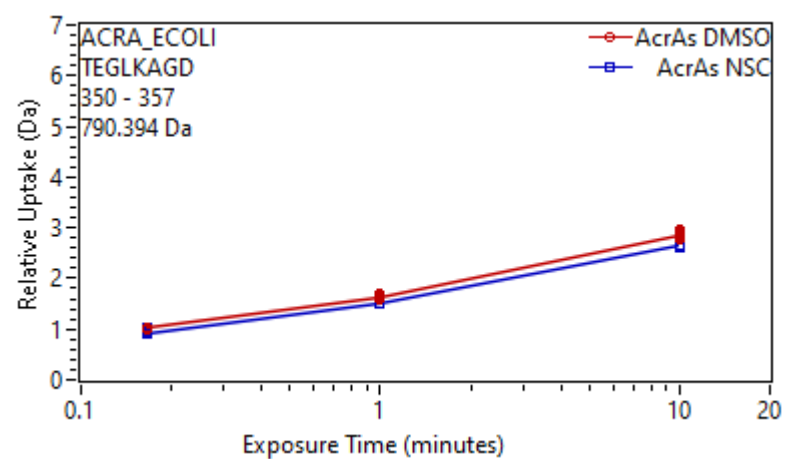

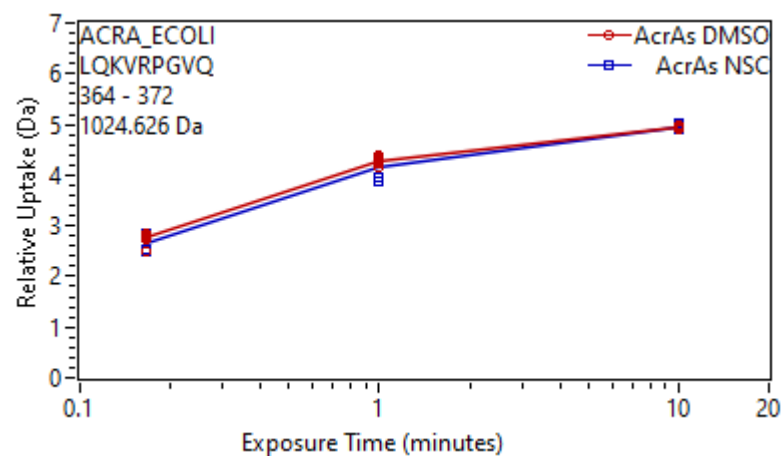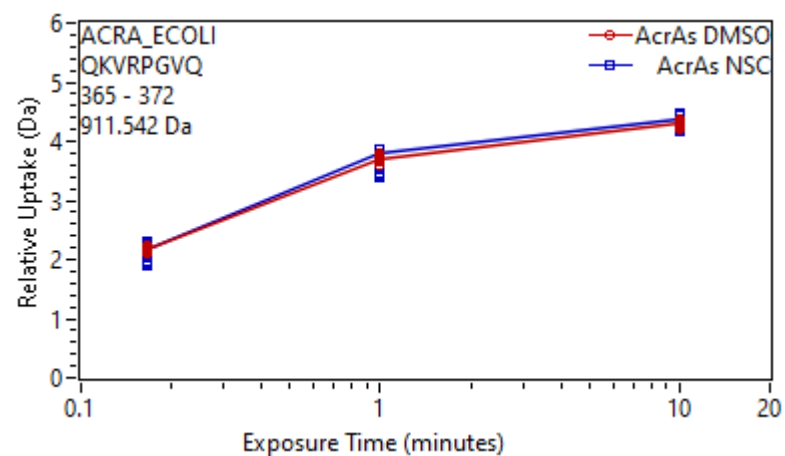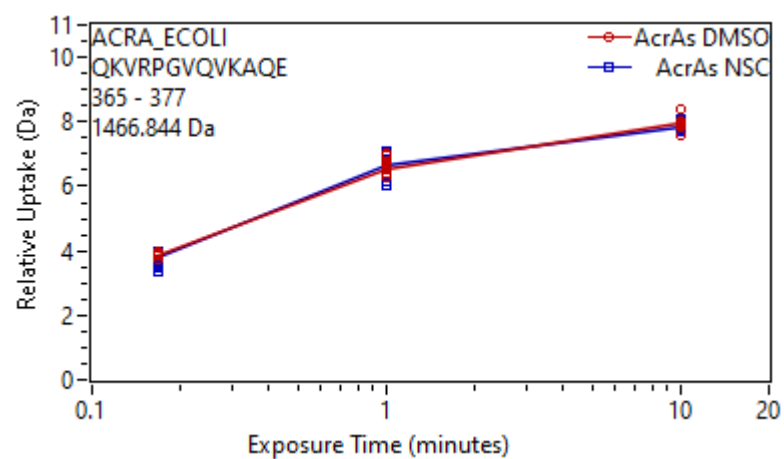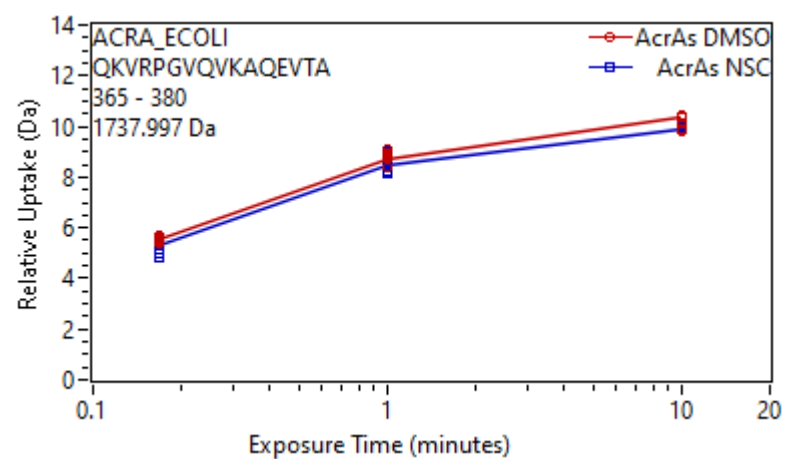

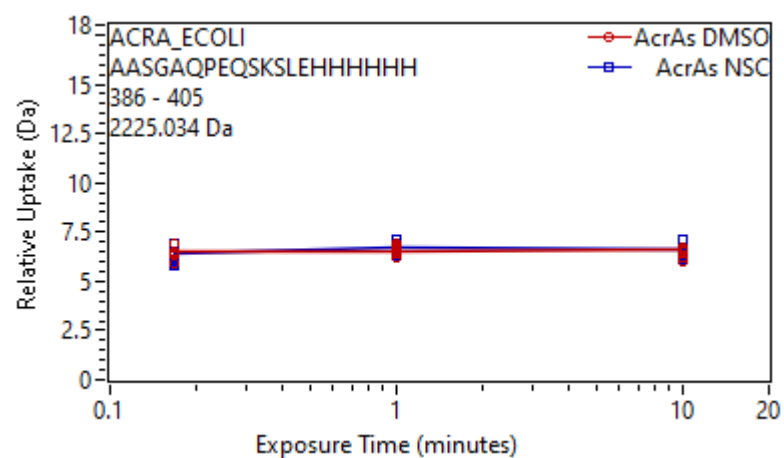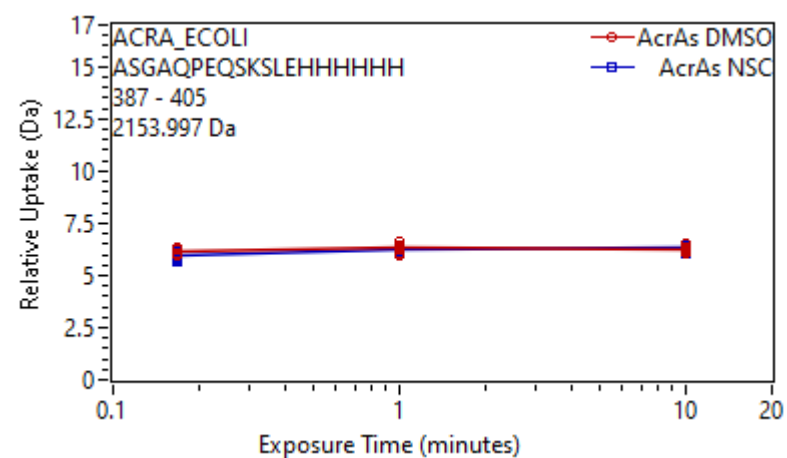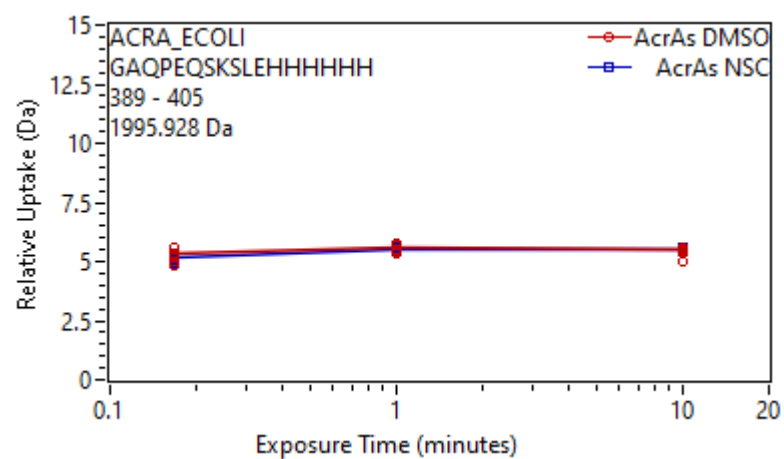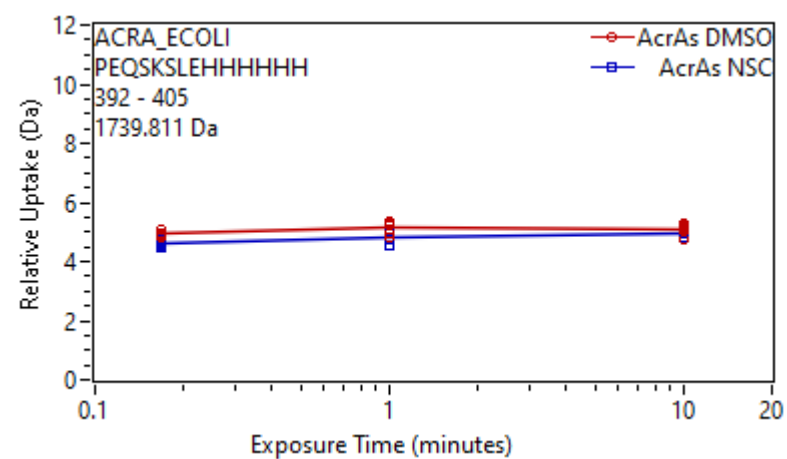

Supplement: Supplementary file 6 — Supplementary Data 3 [file 41467_2023_39615_MOESM6_ESM.zip › Supplementary Data 3/AcrAs_NSC60339_UptakePlots.pdf]

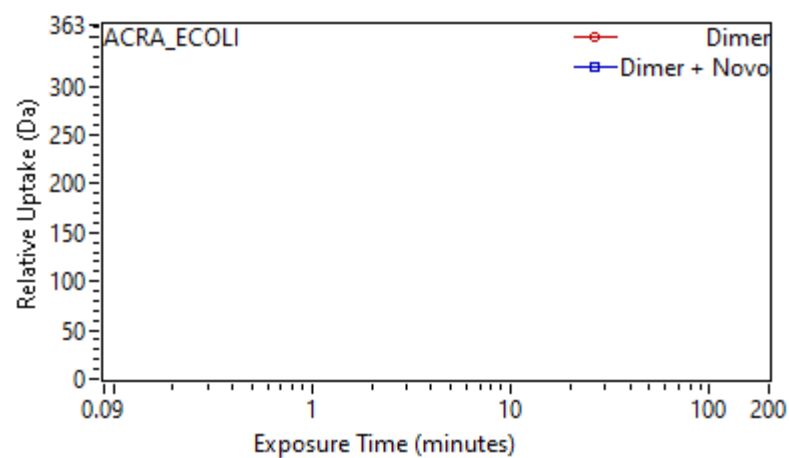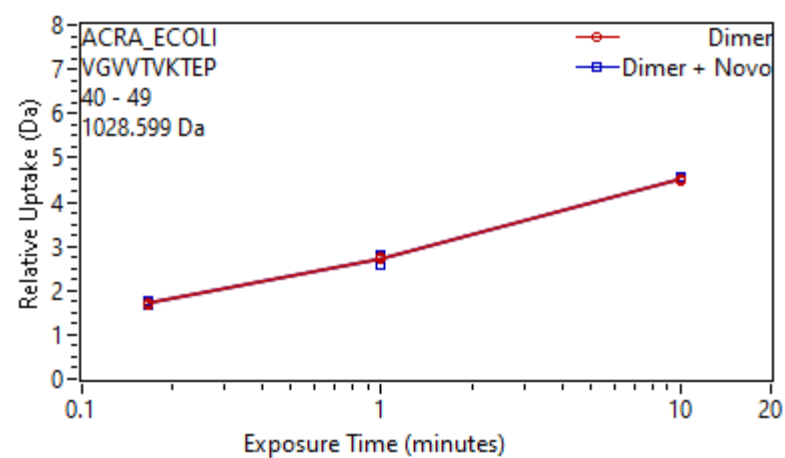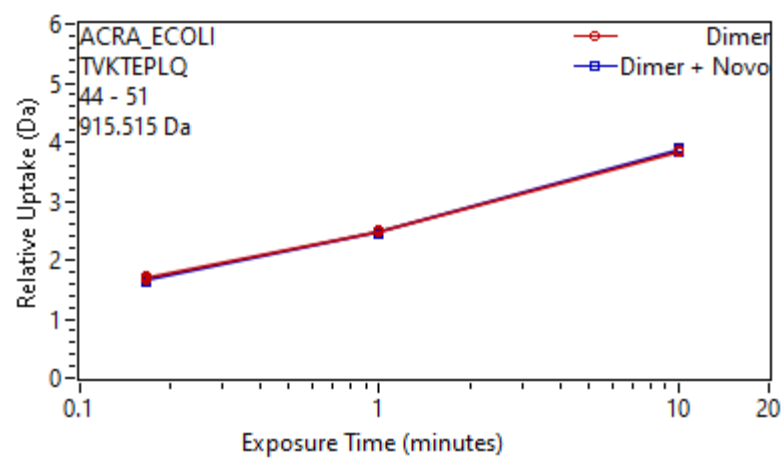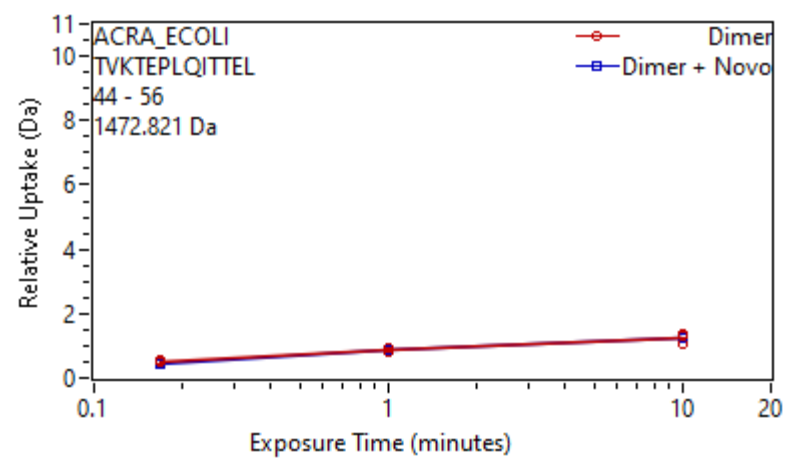

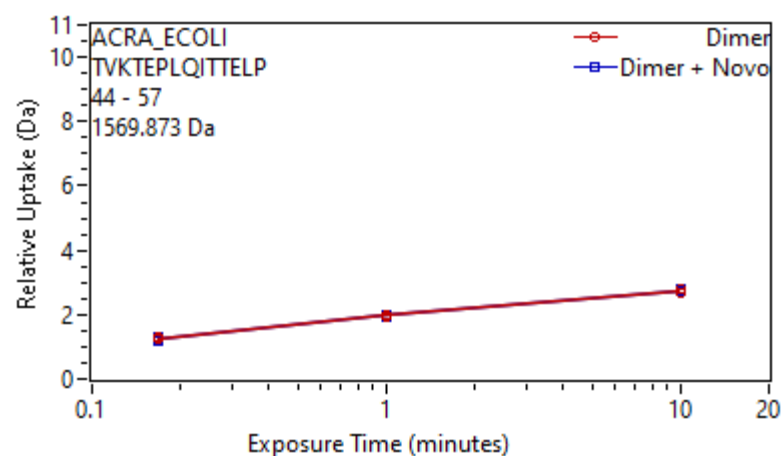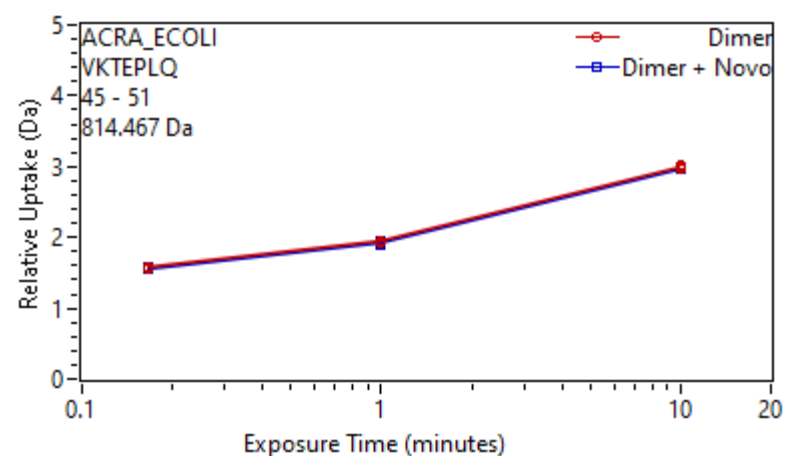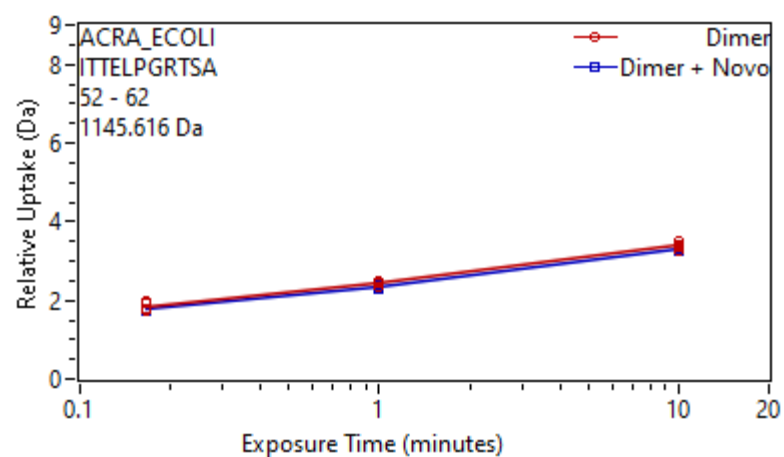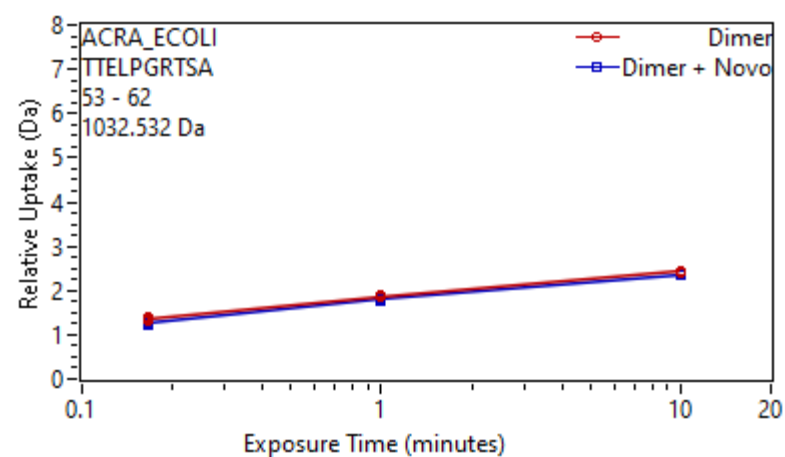

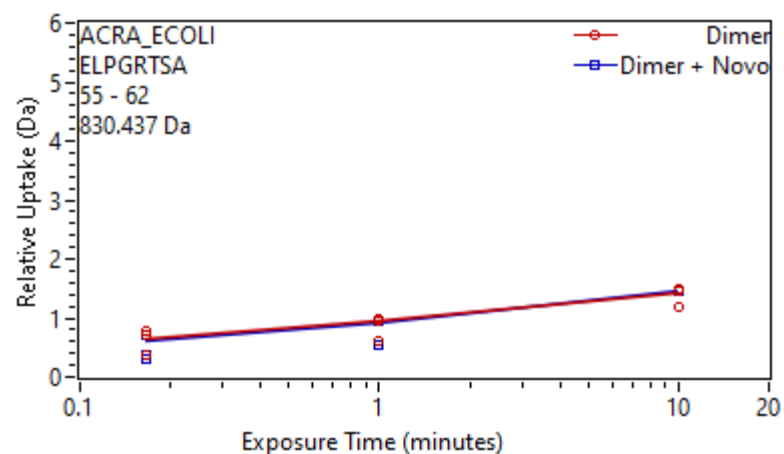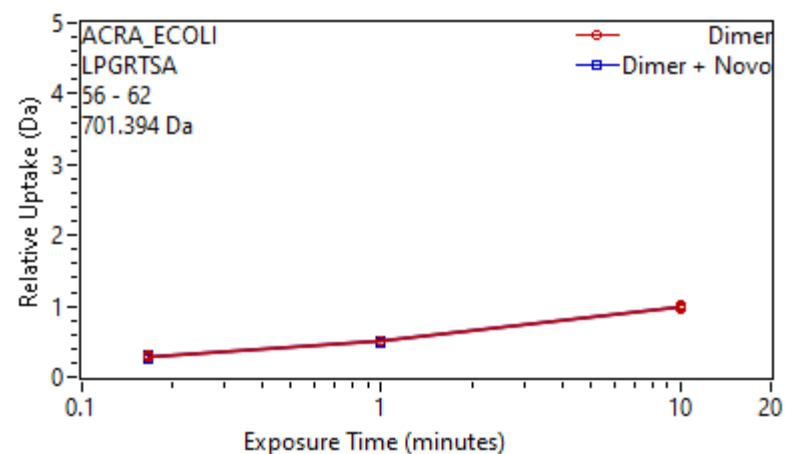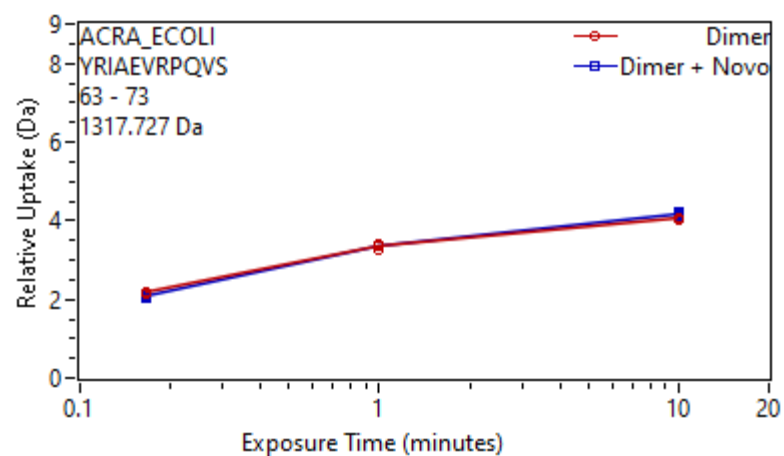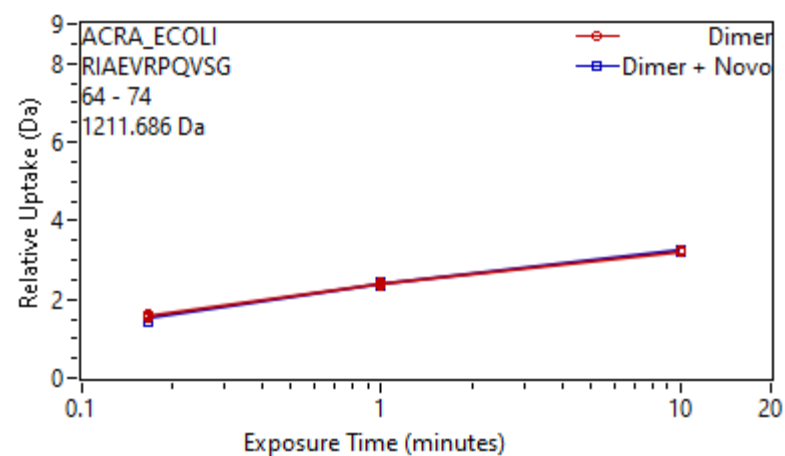

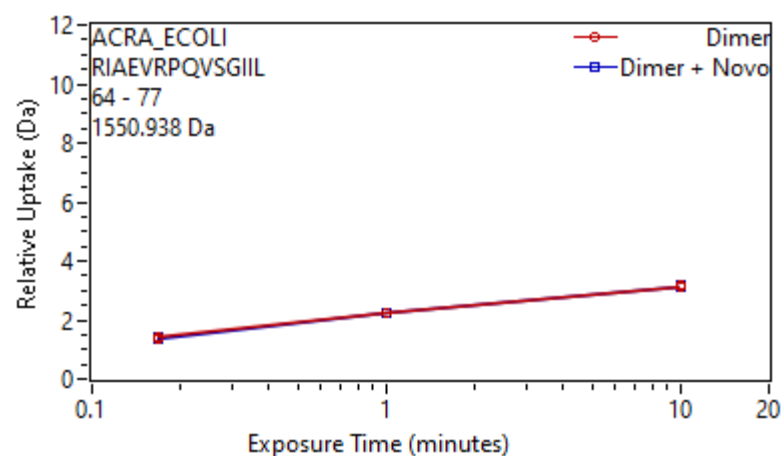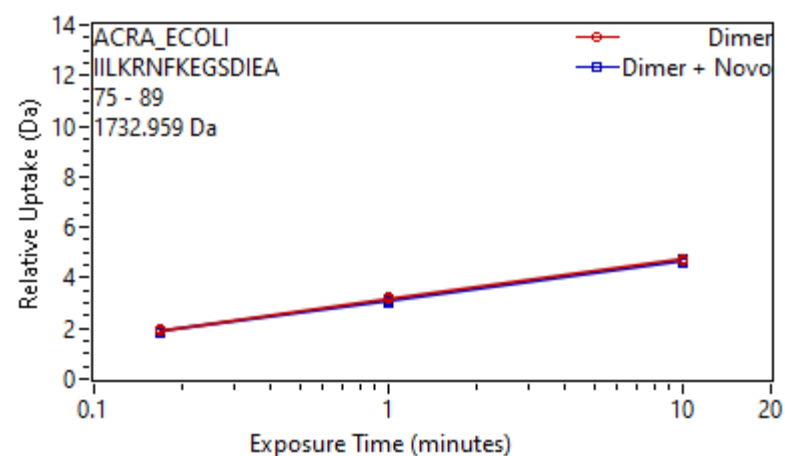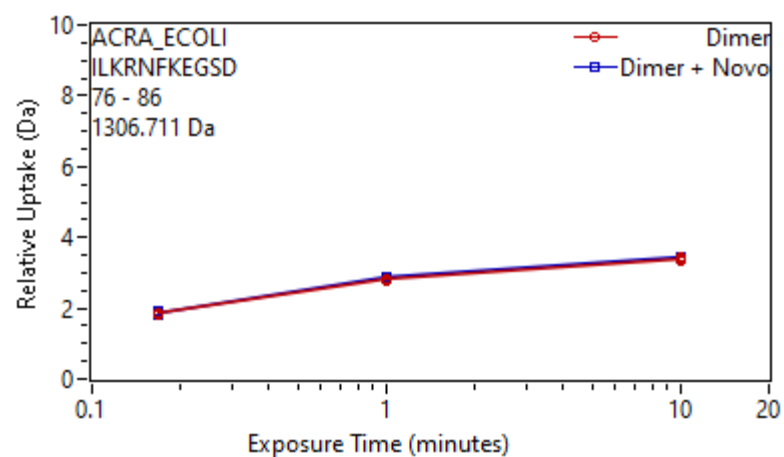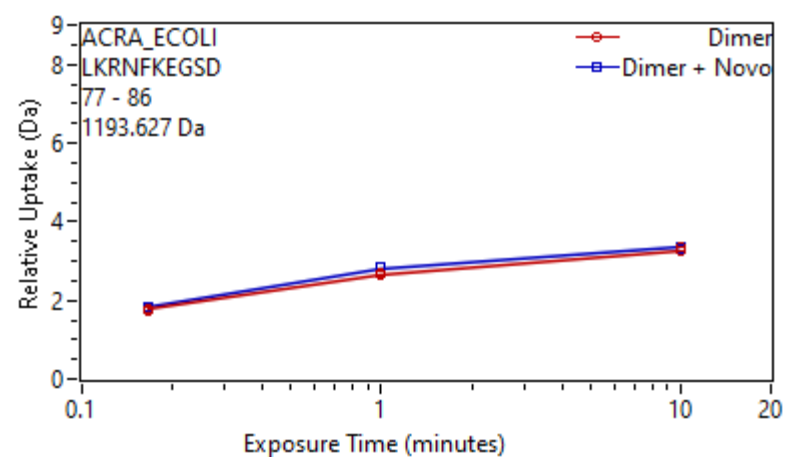

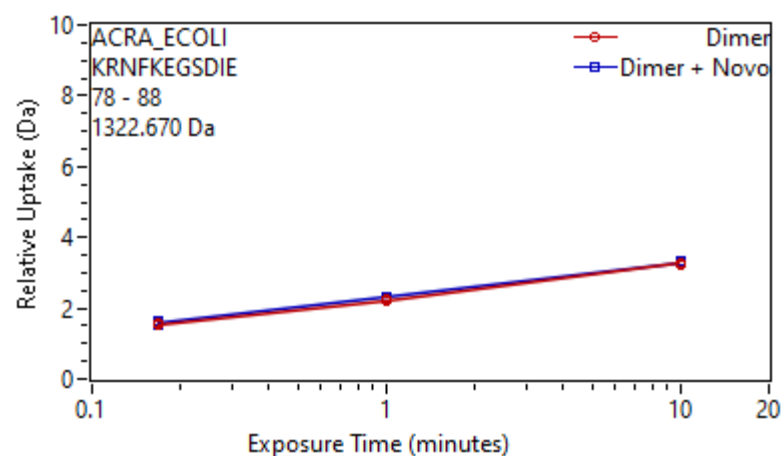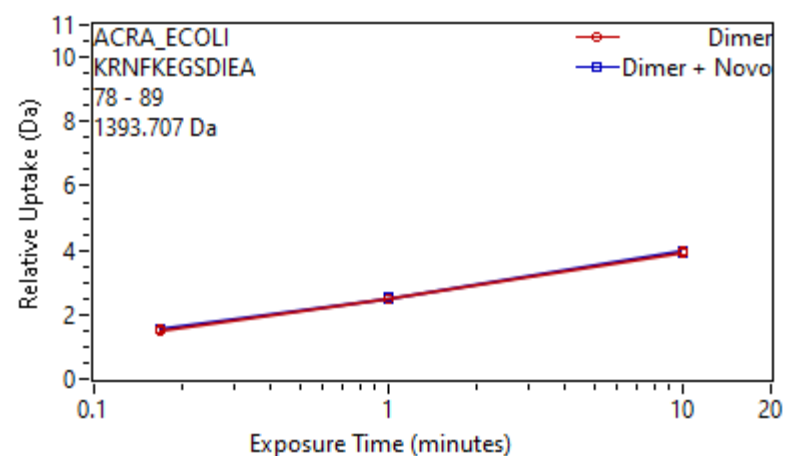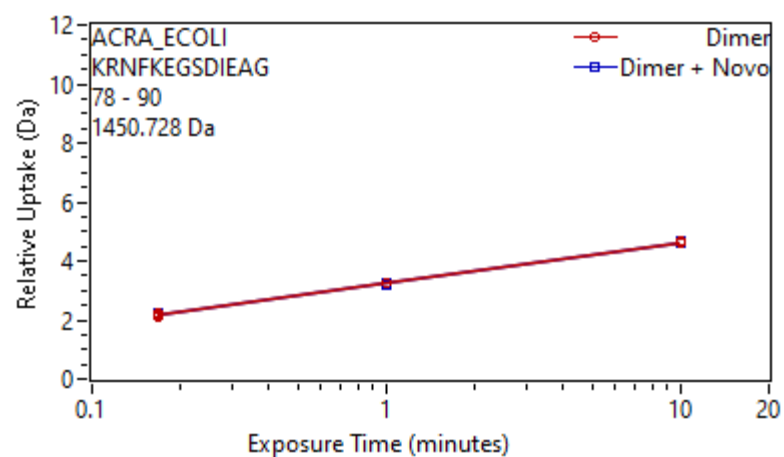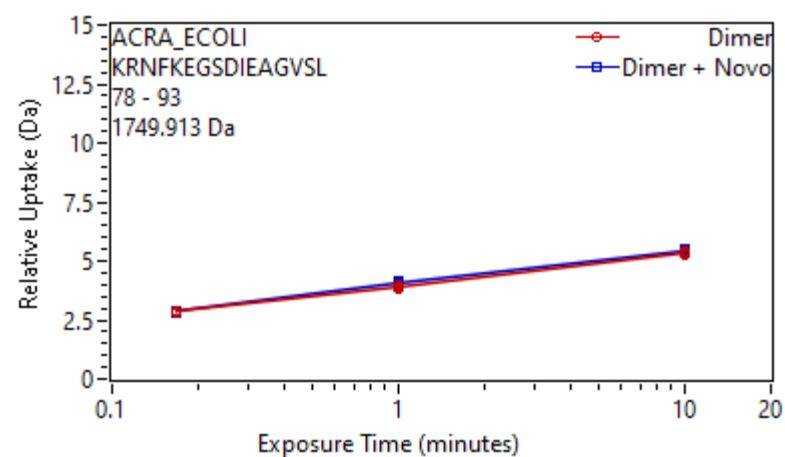

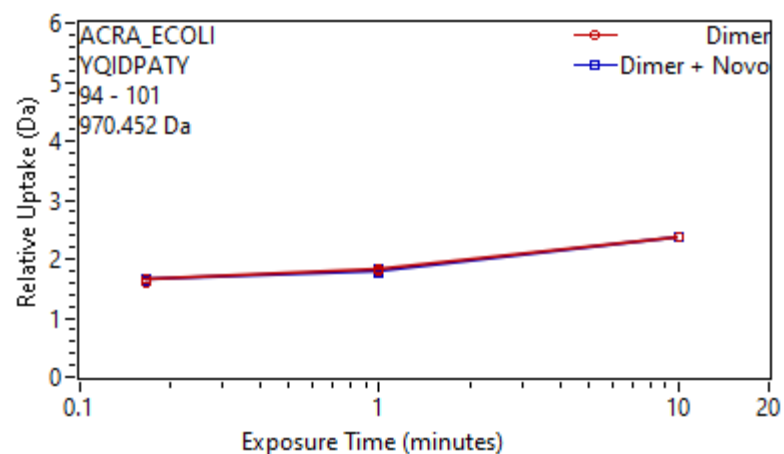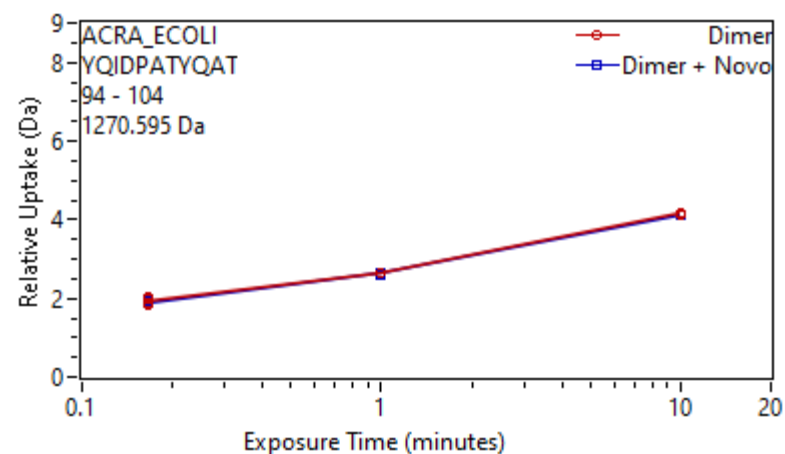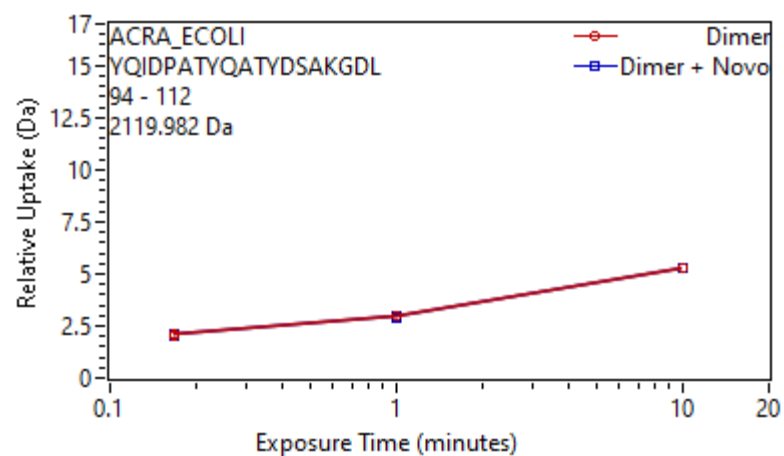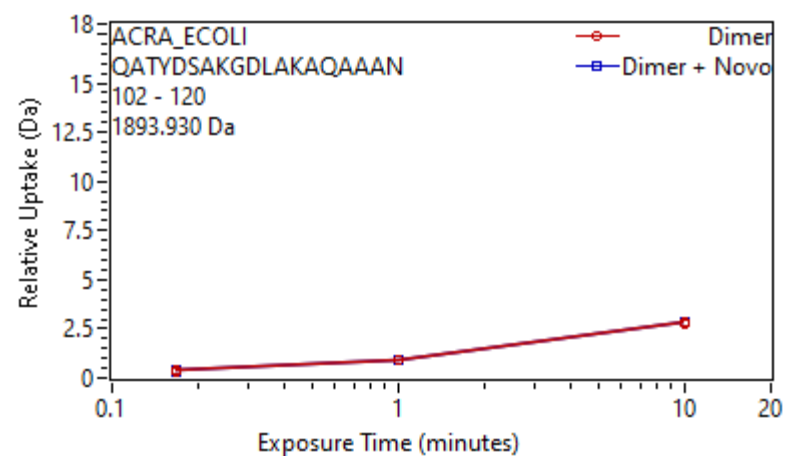

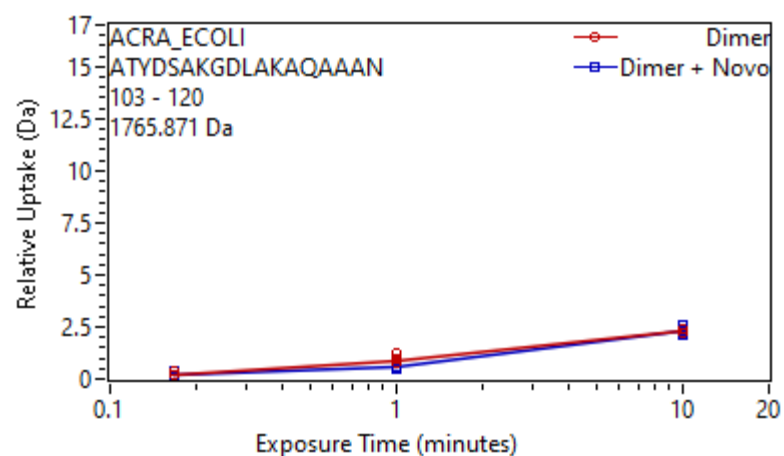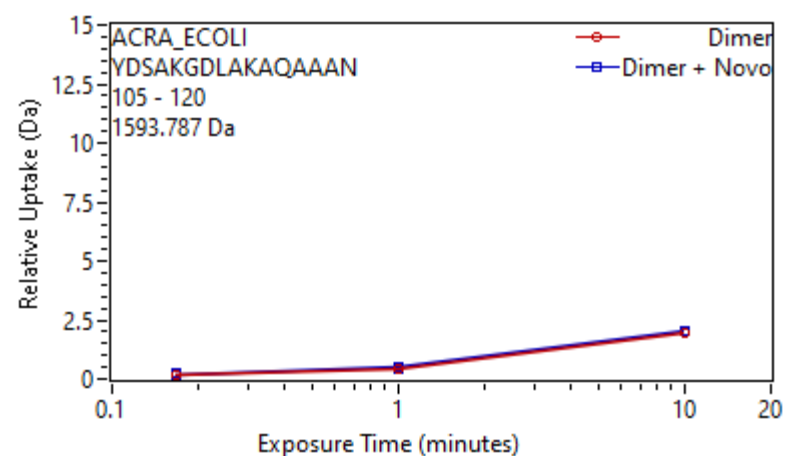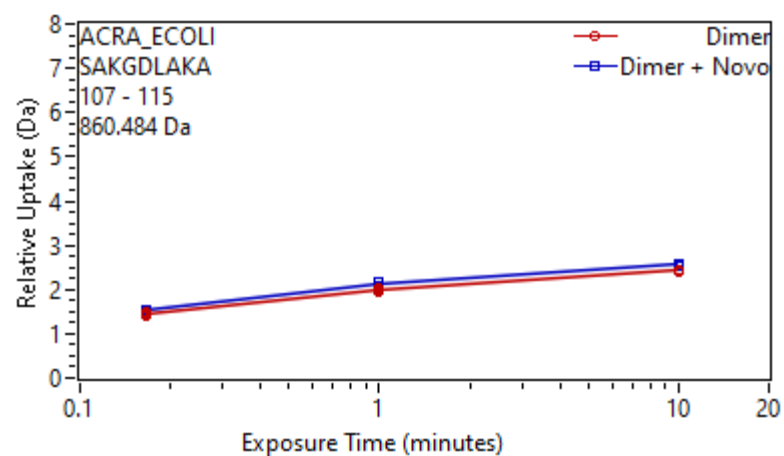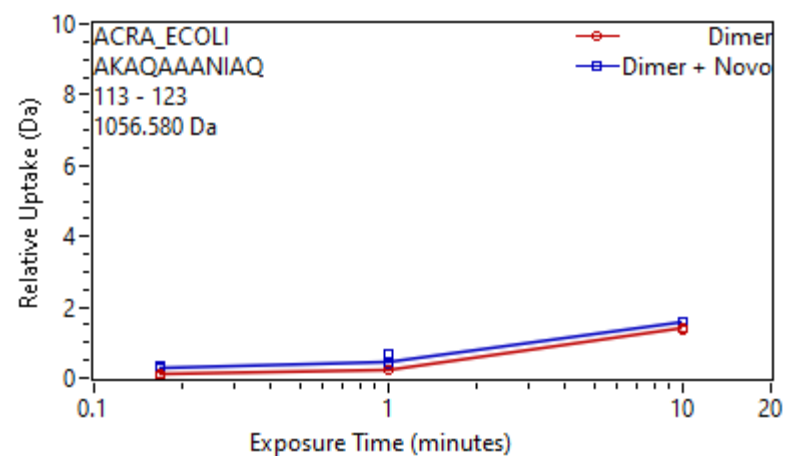

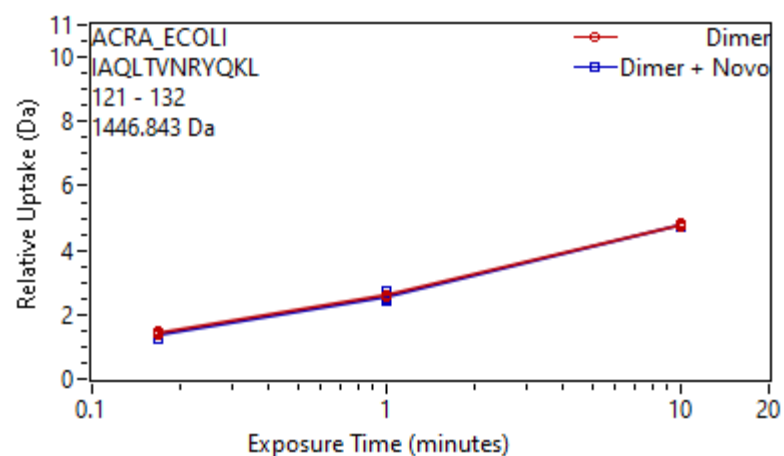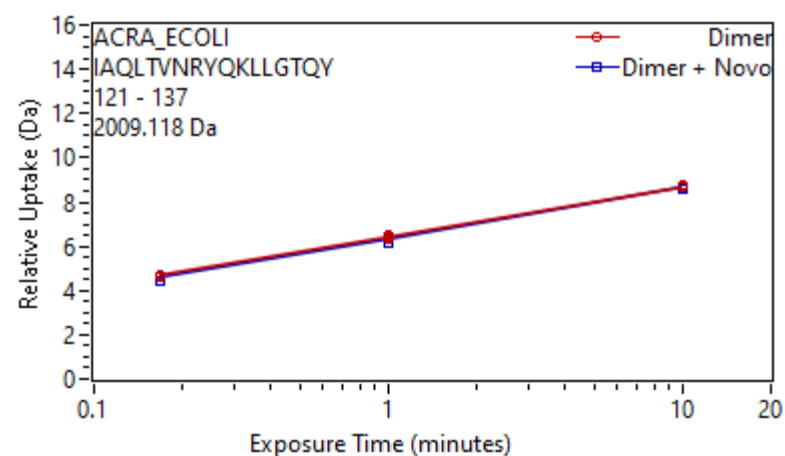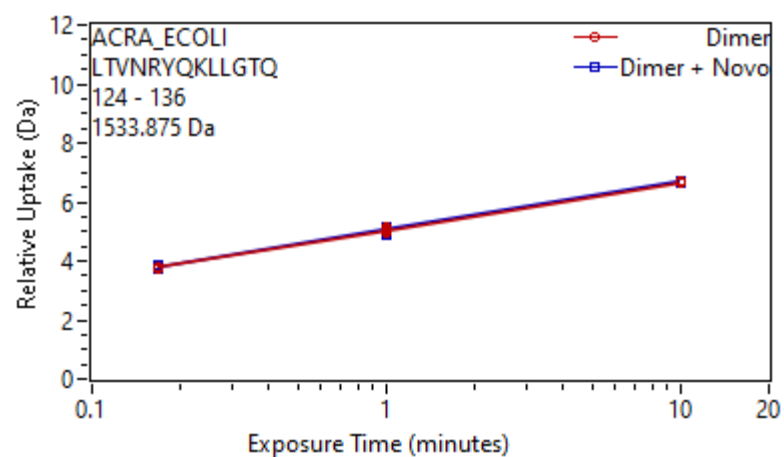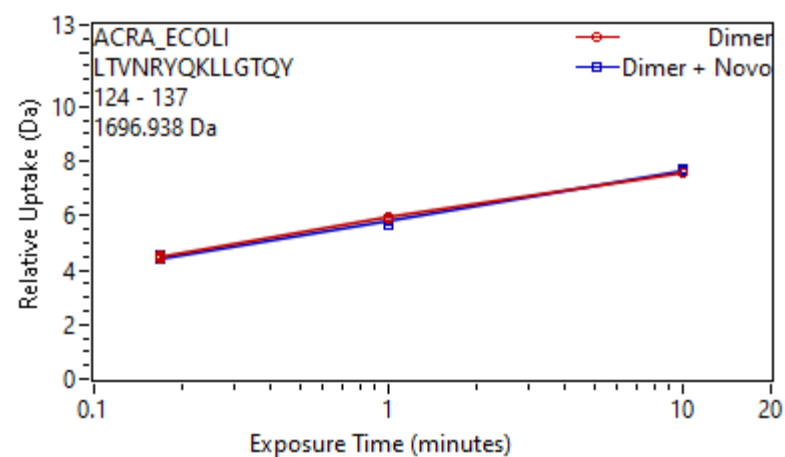

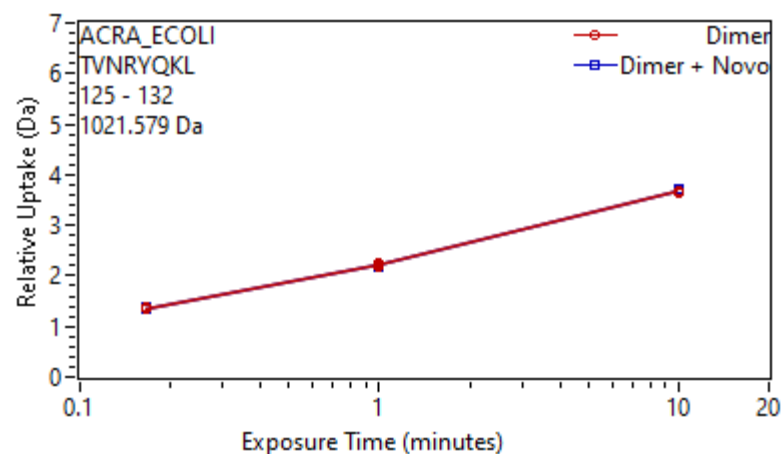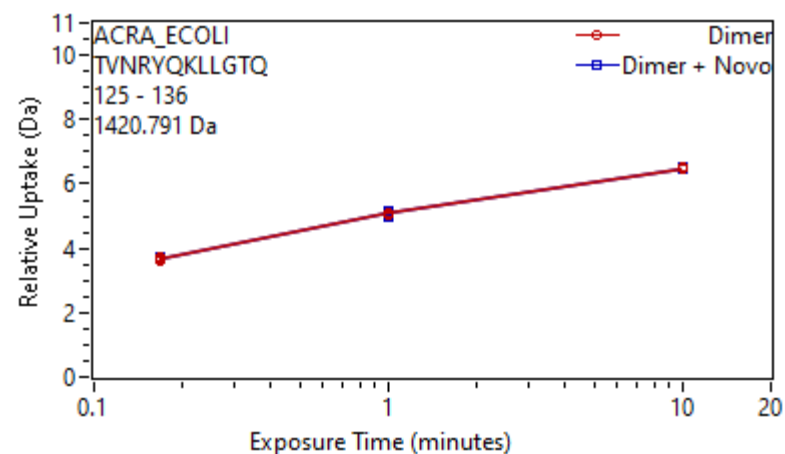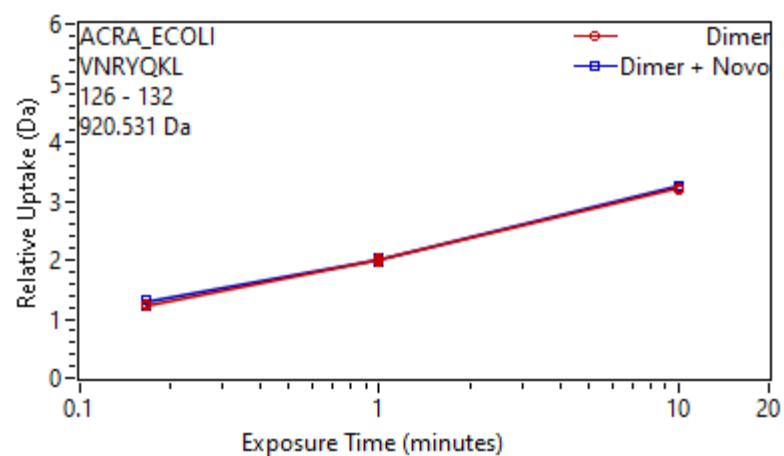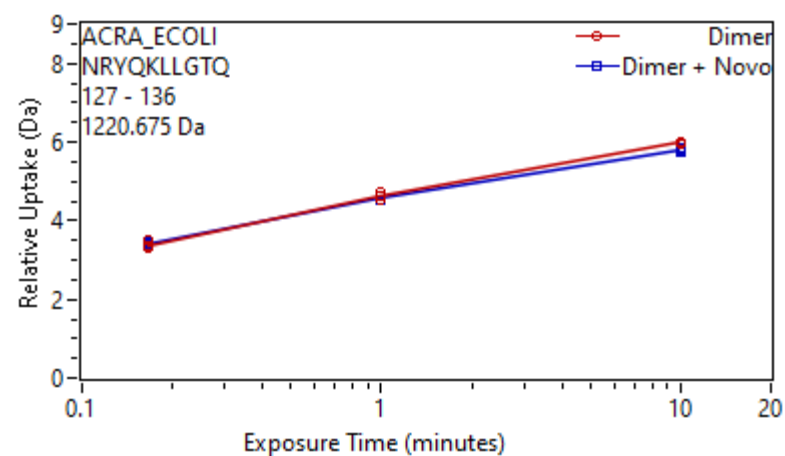

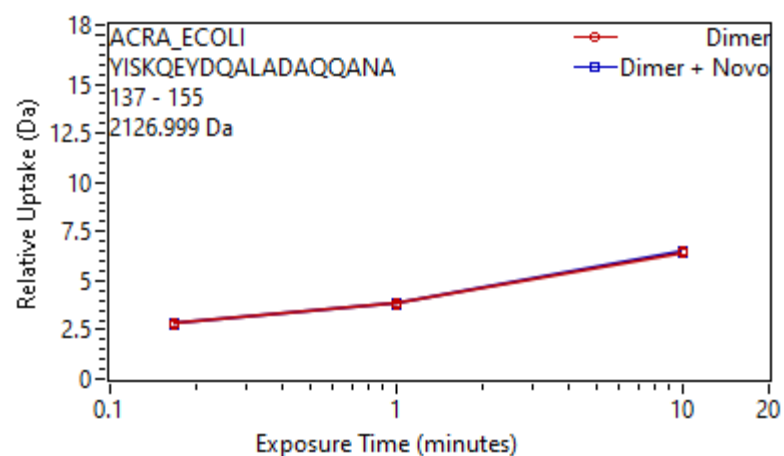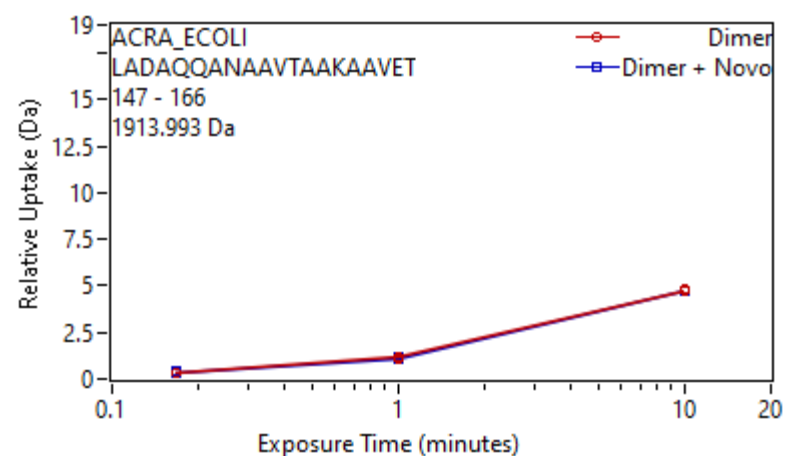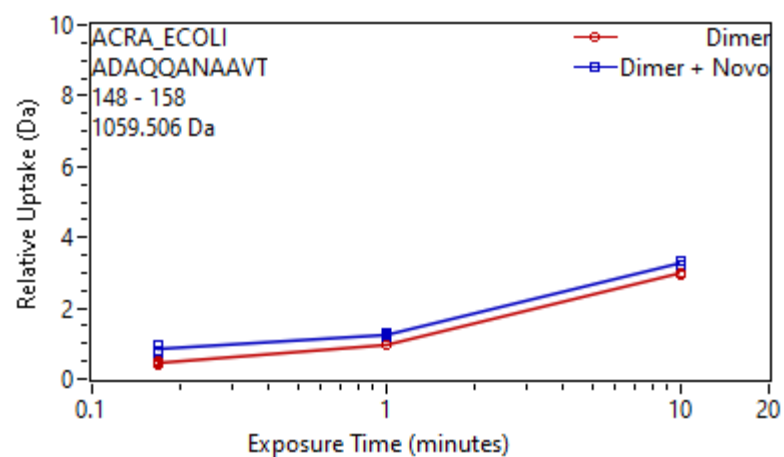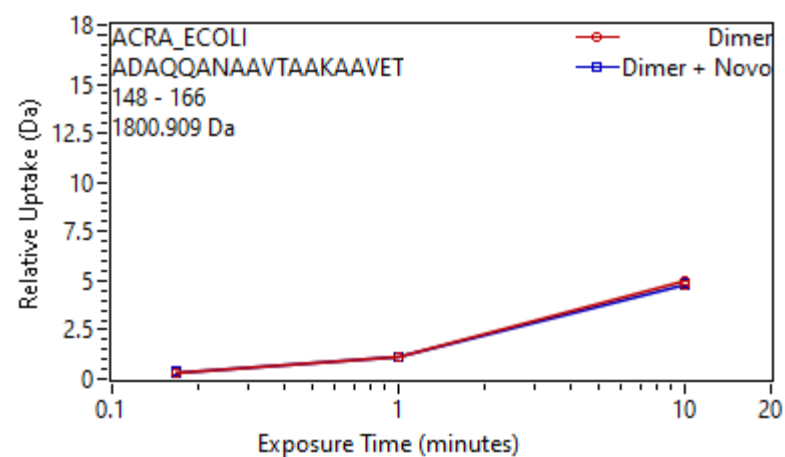

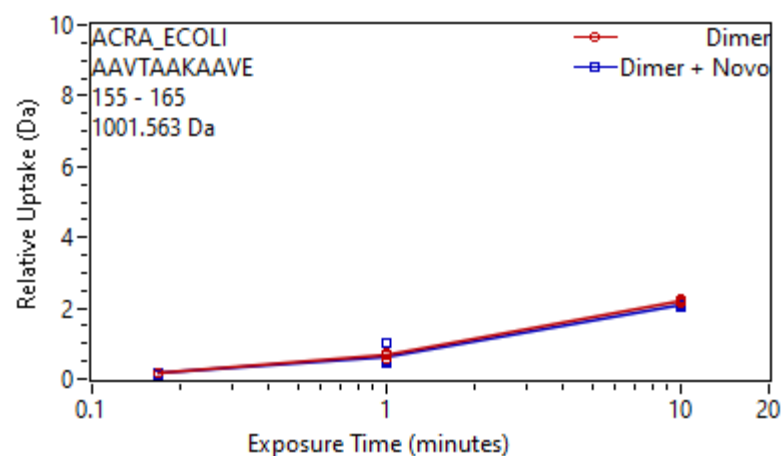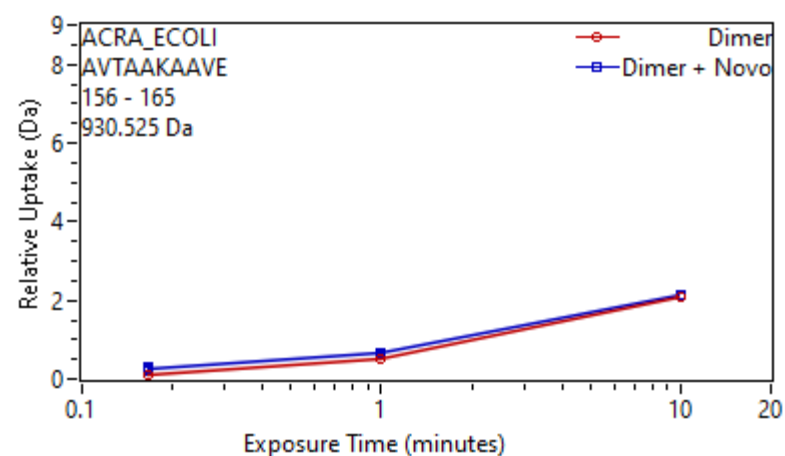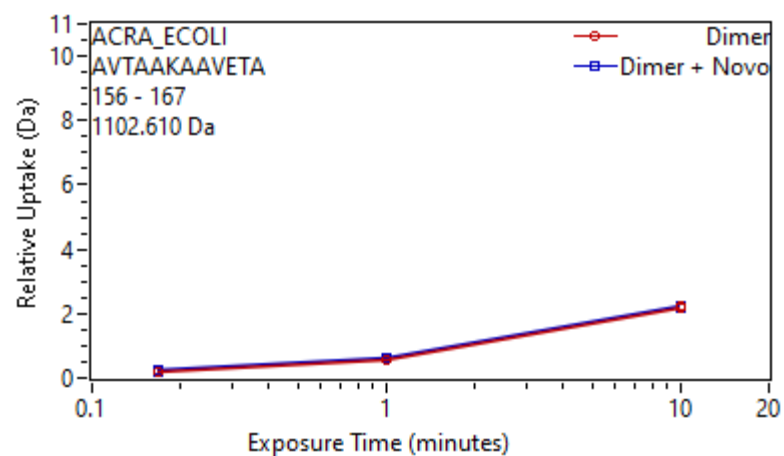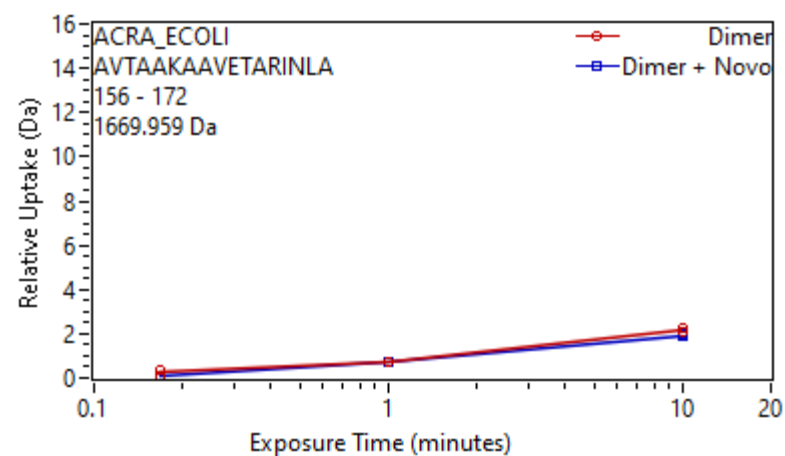

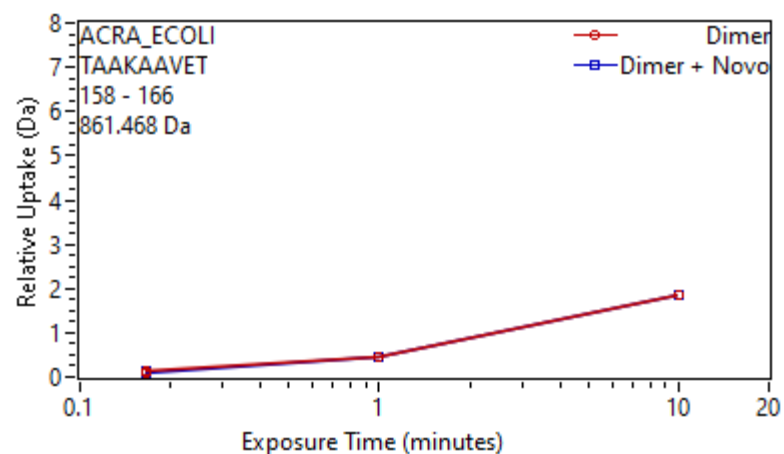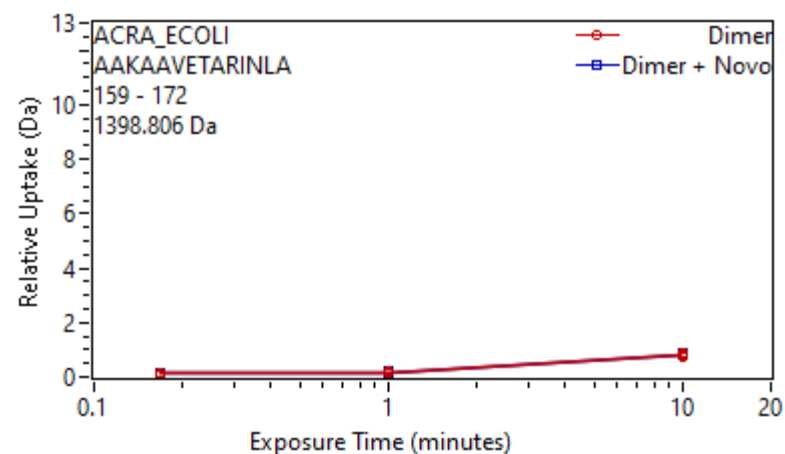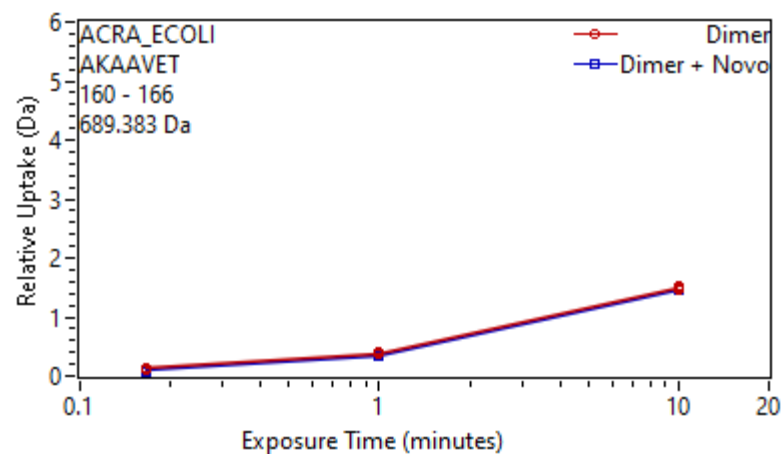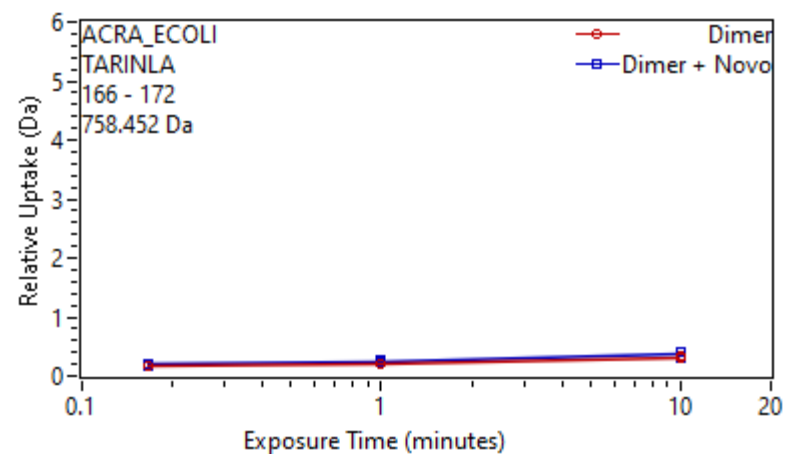

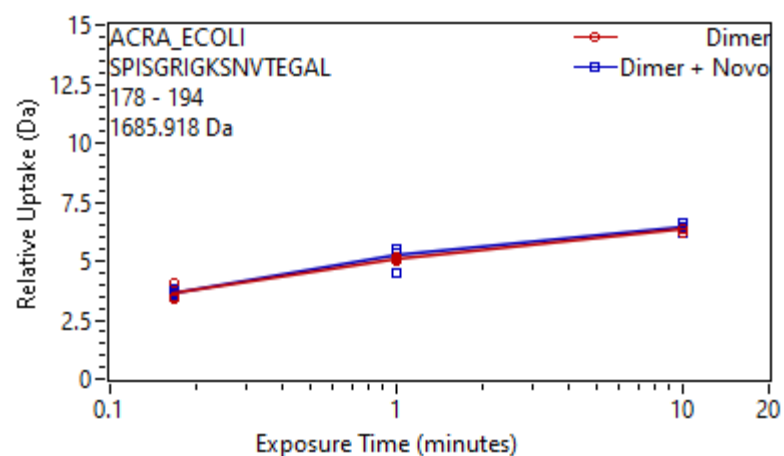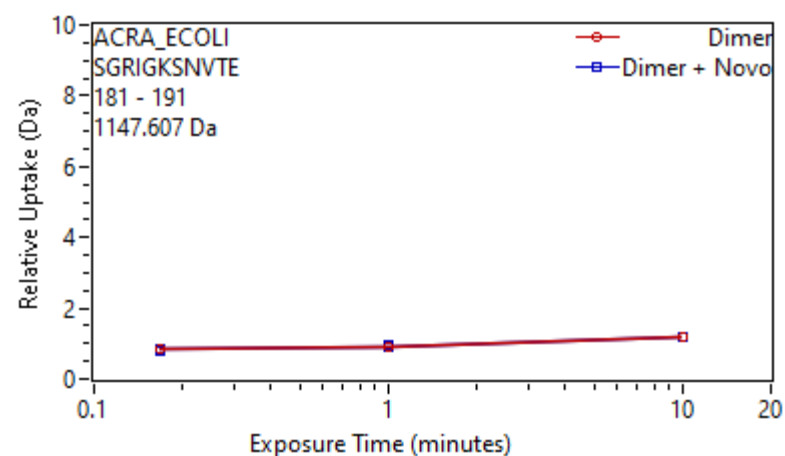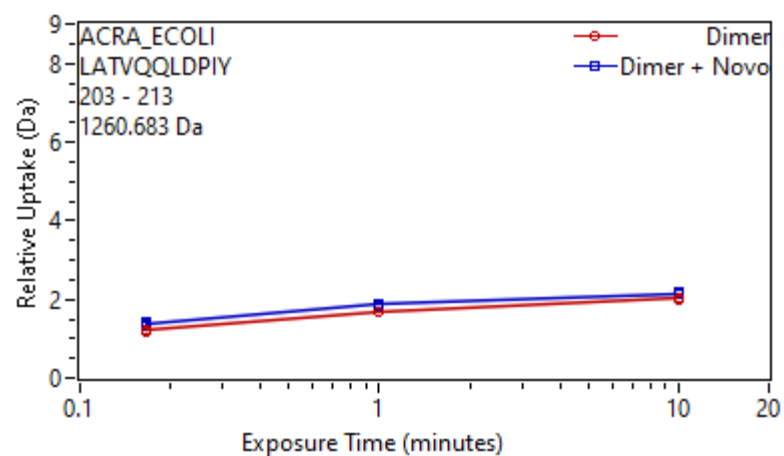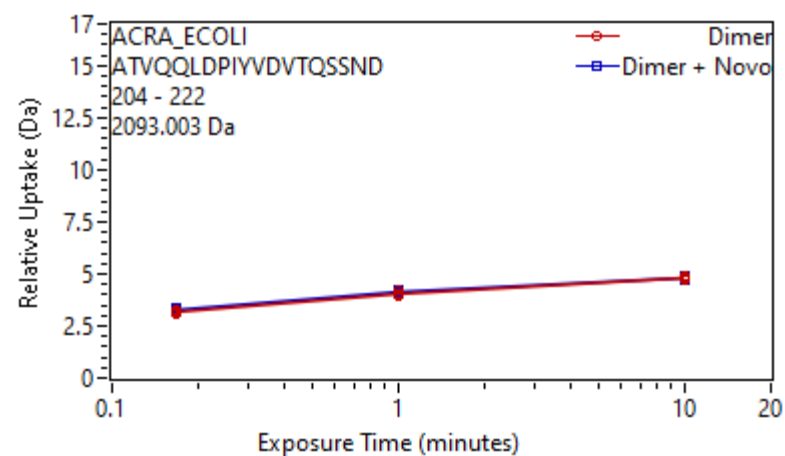

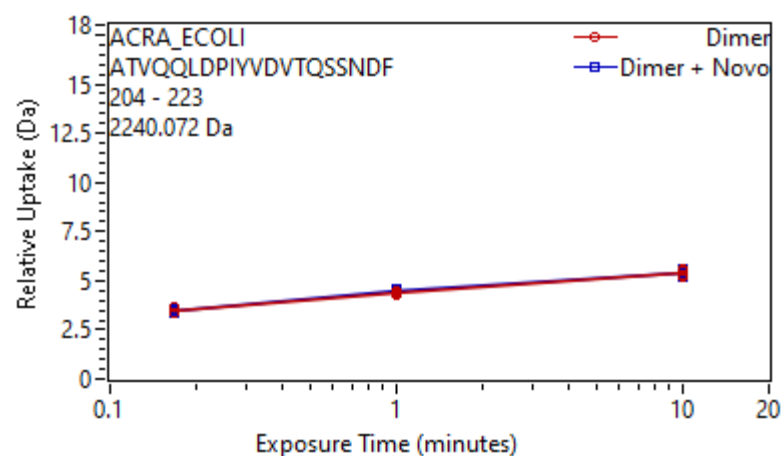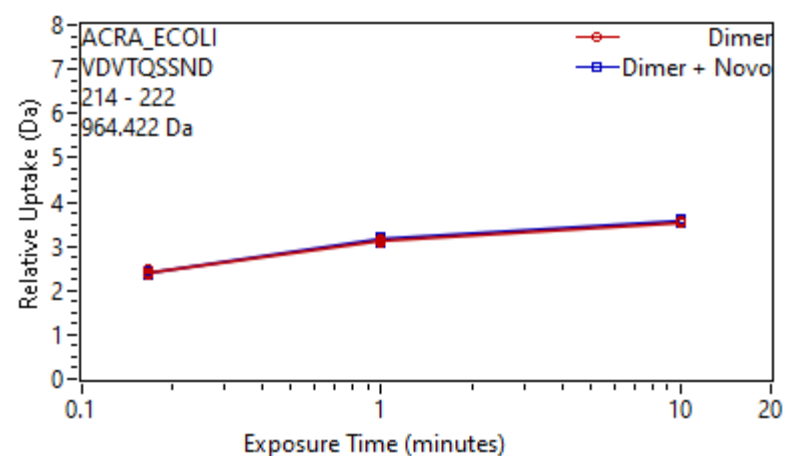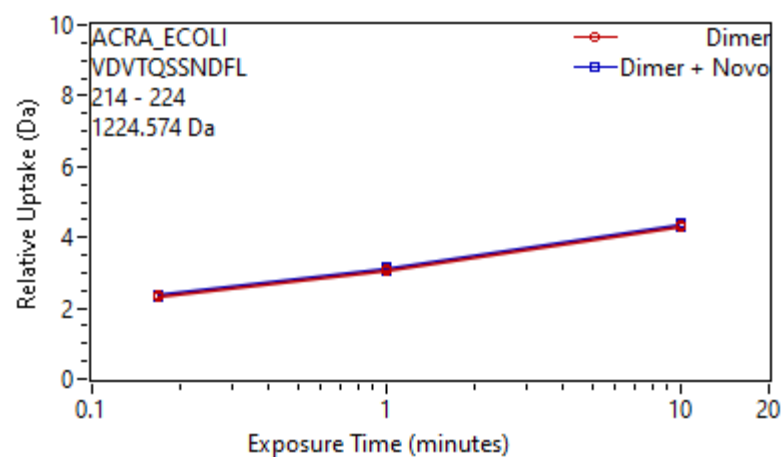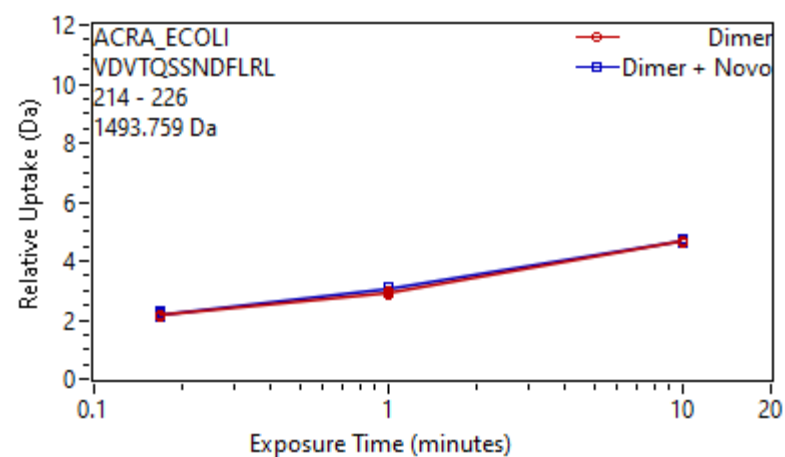

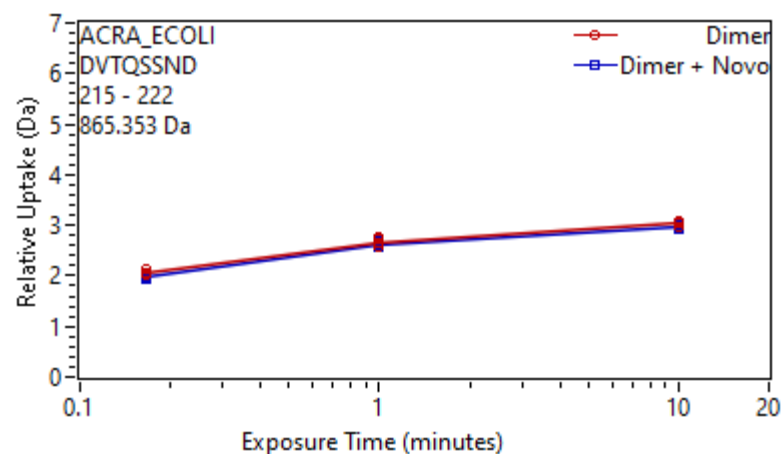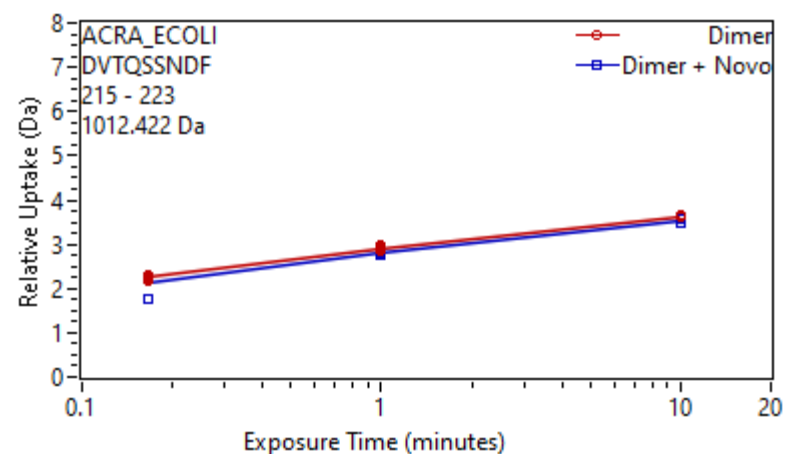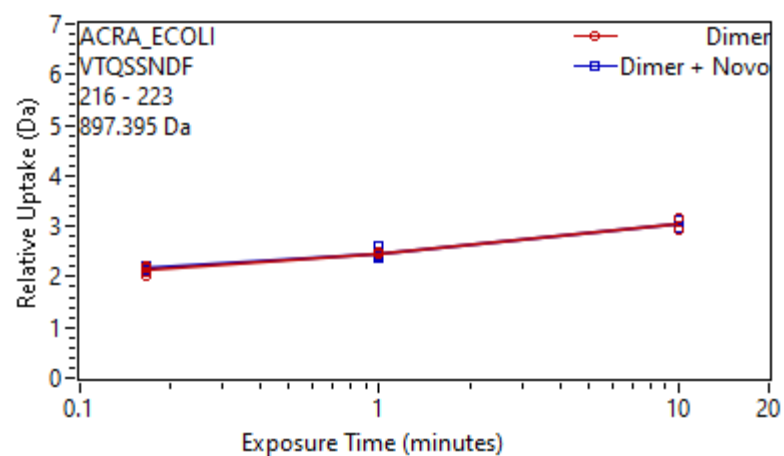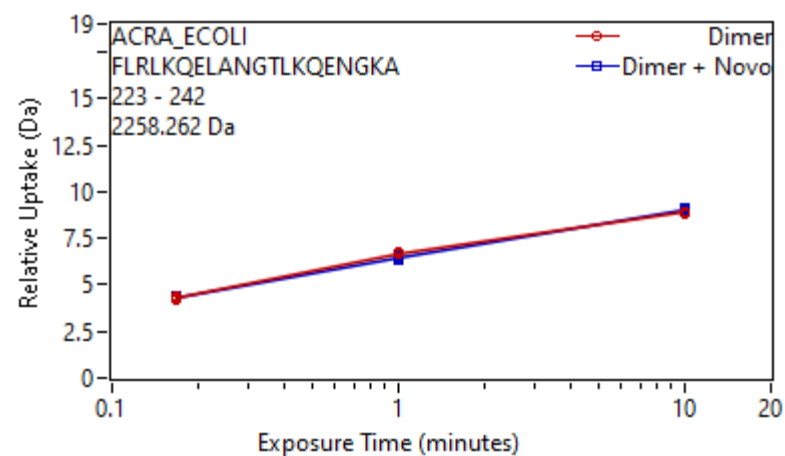

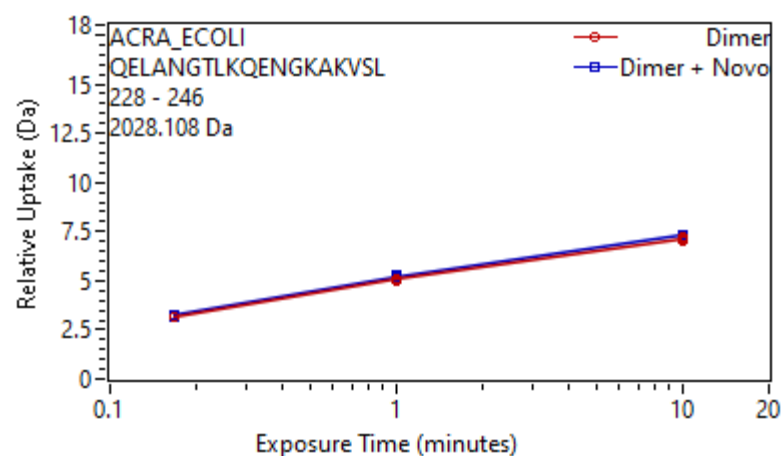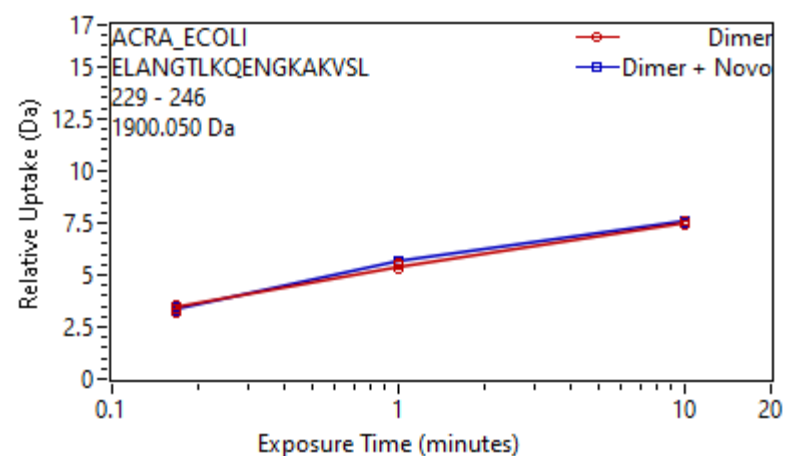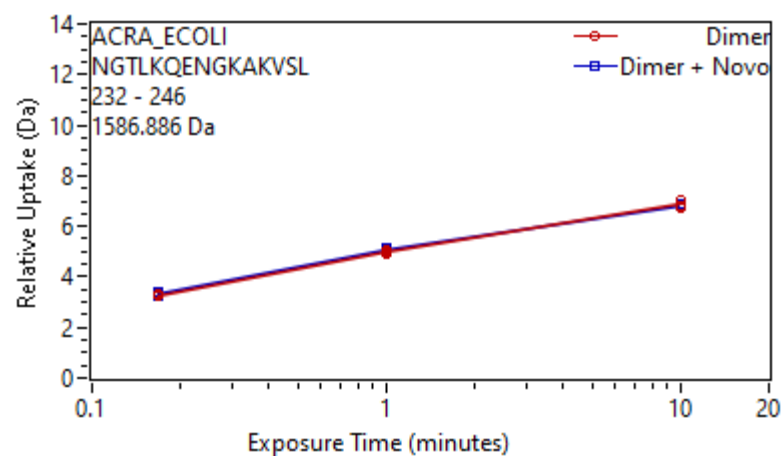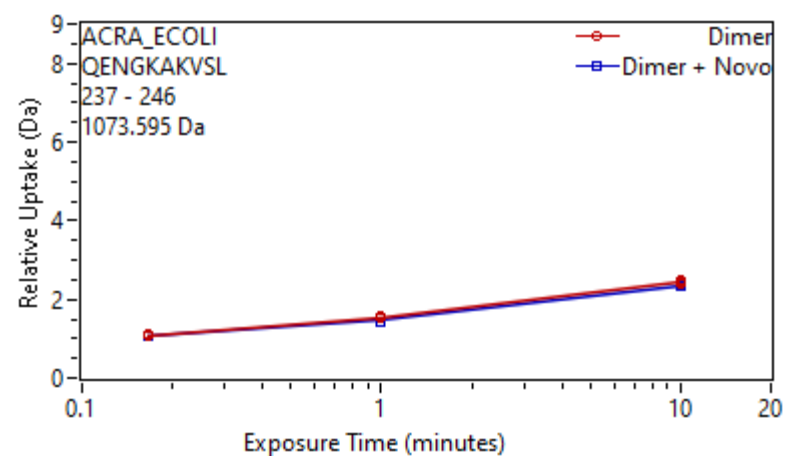

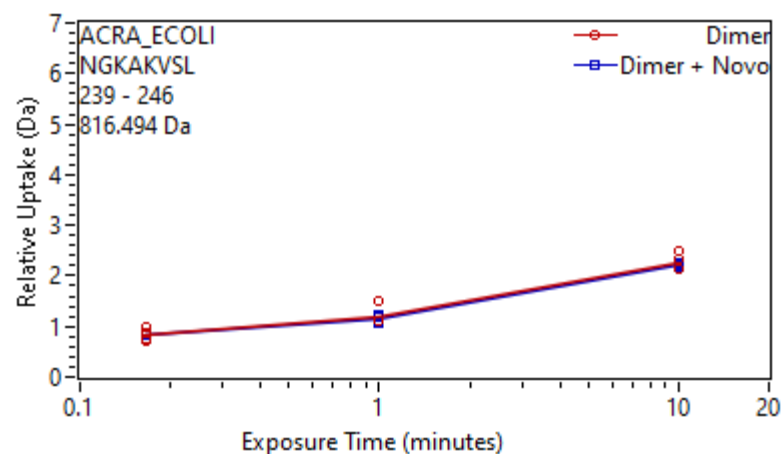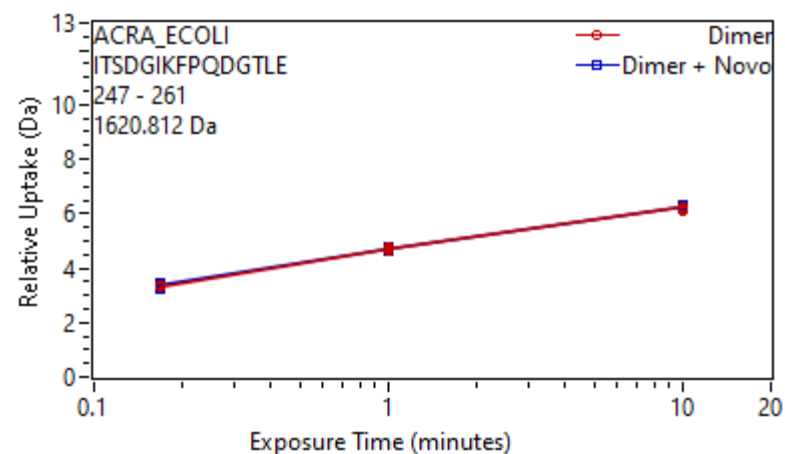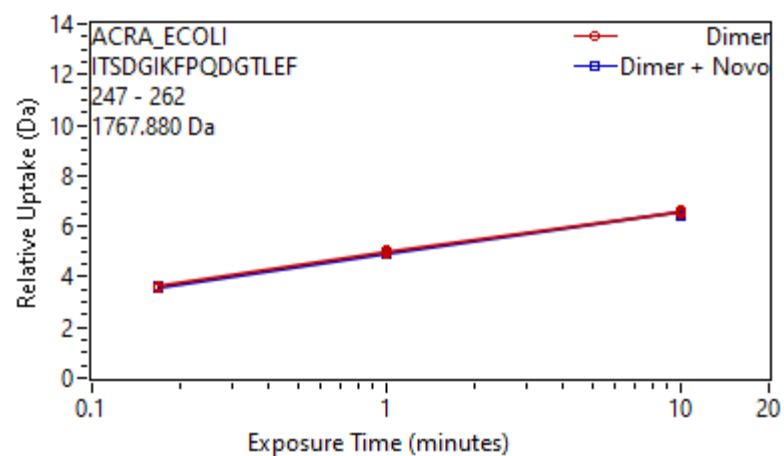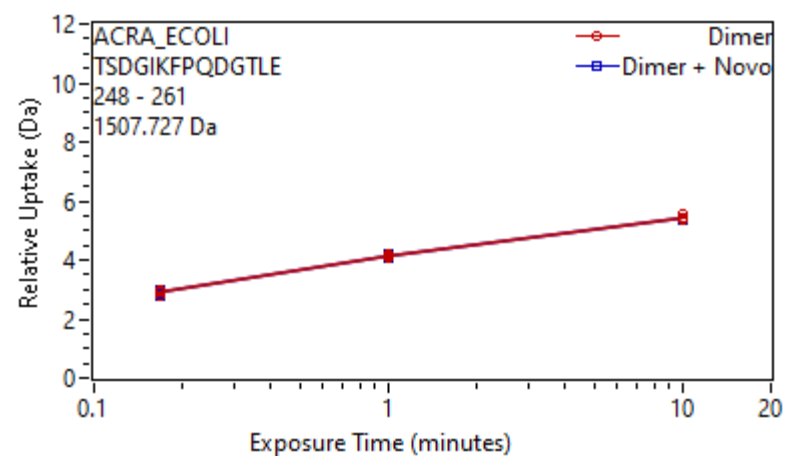

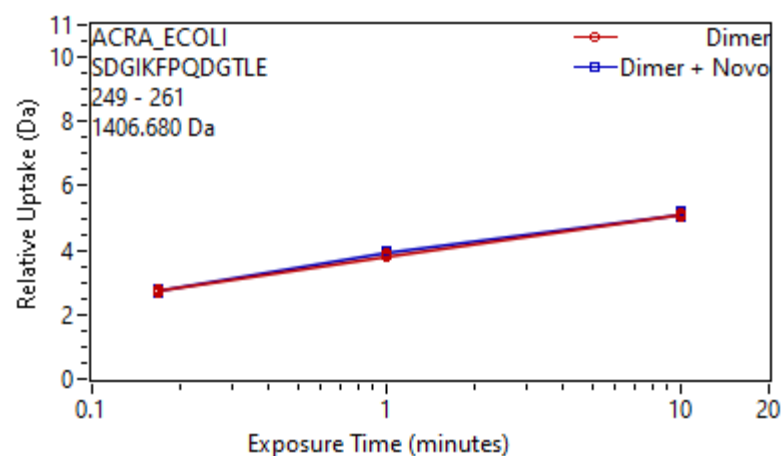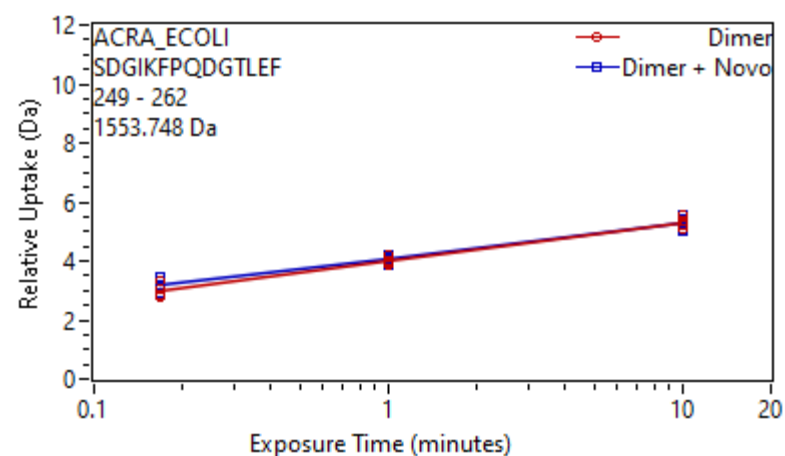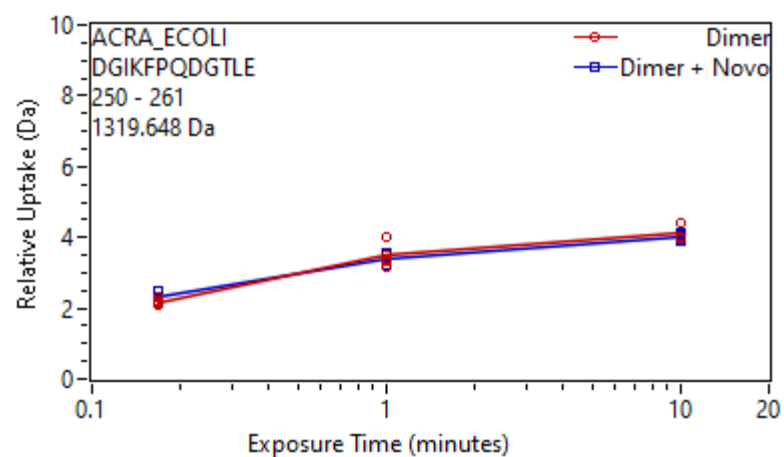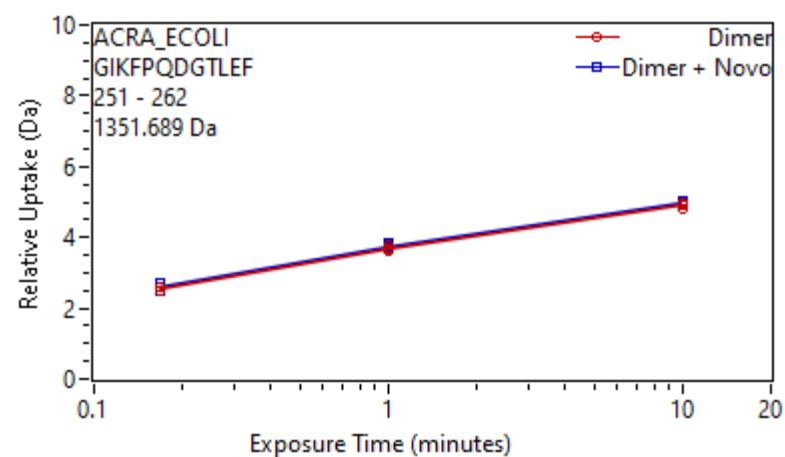

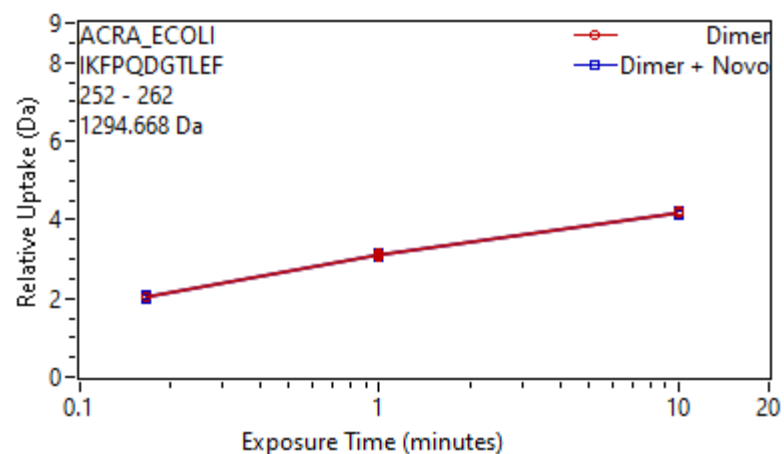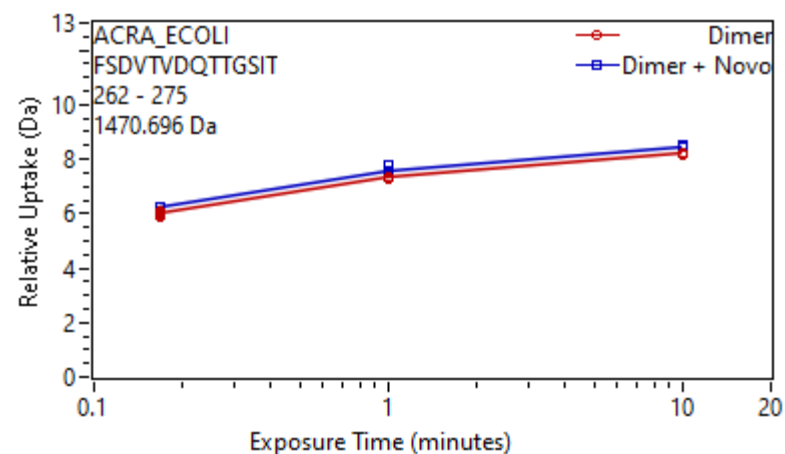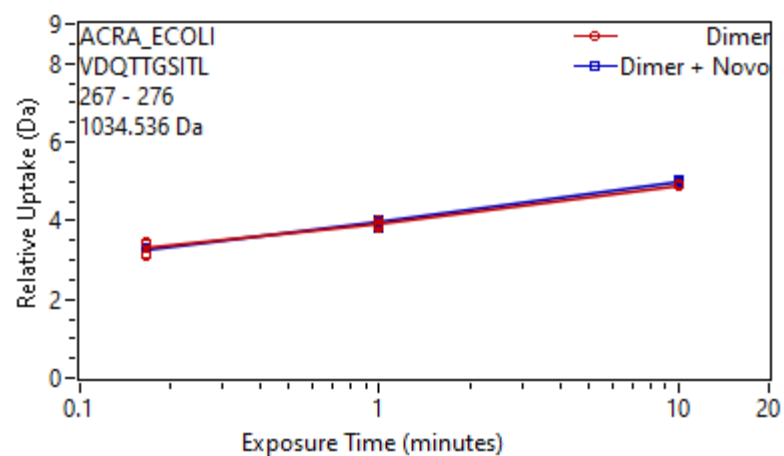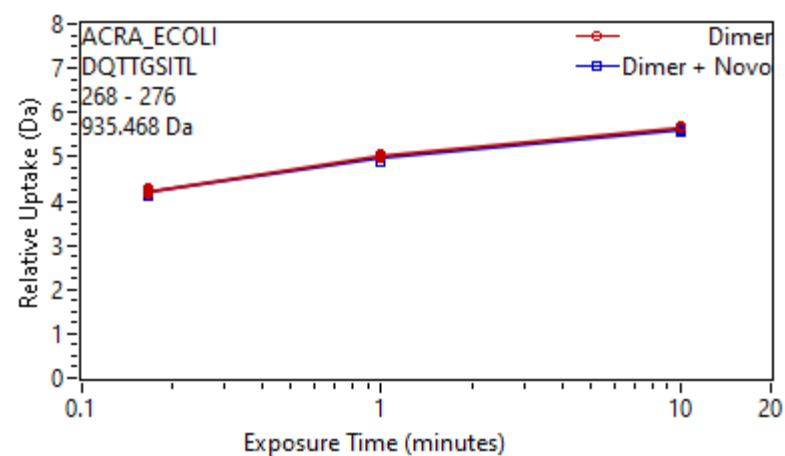

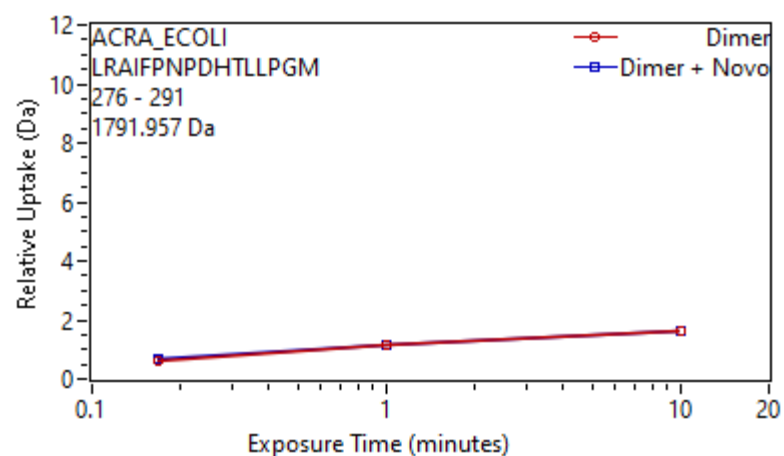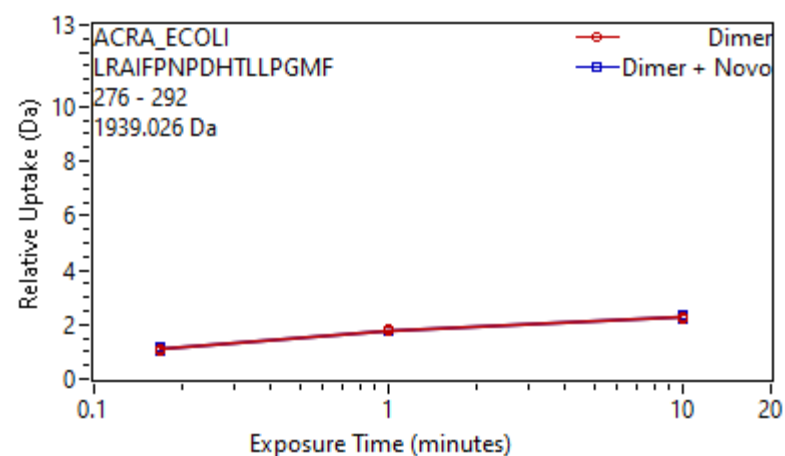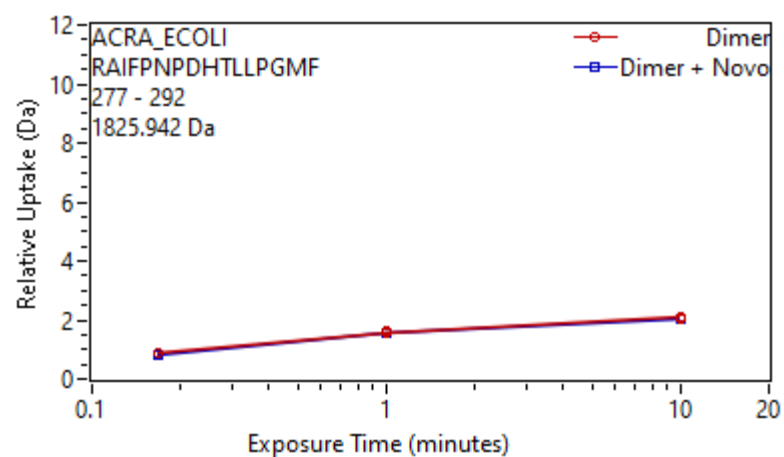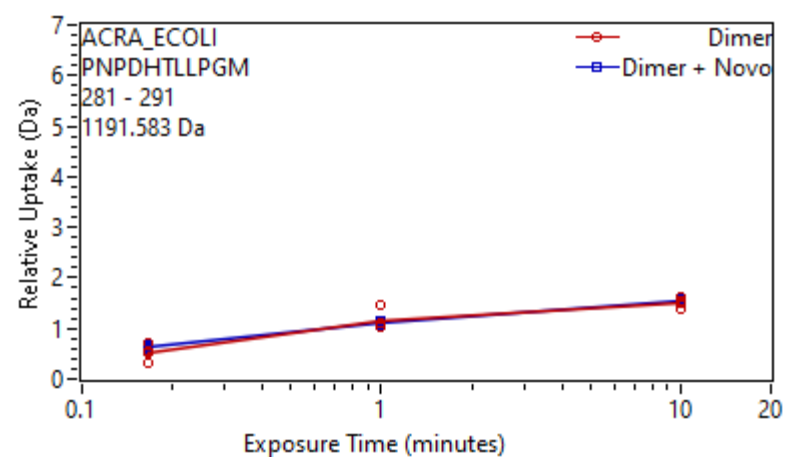

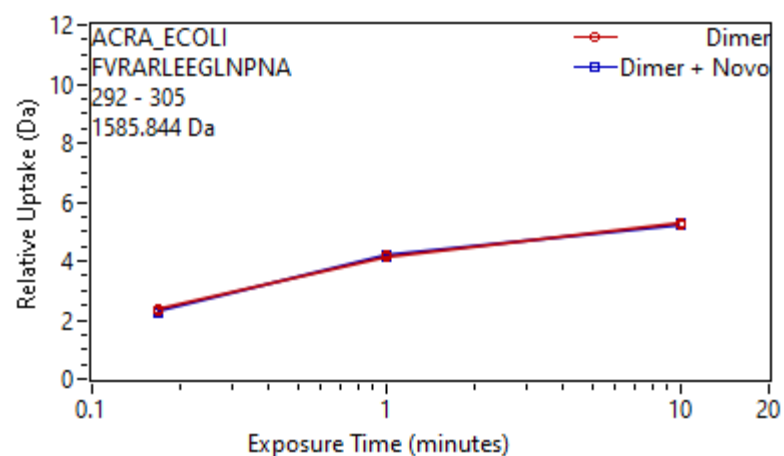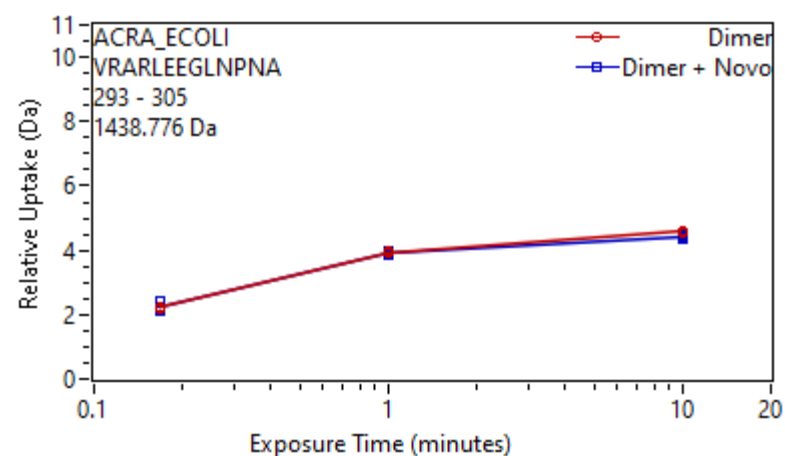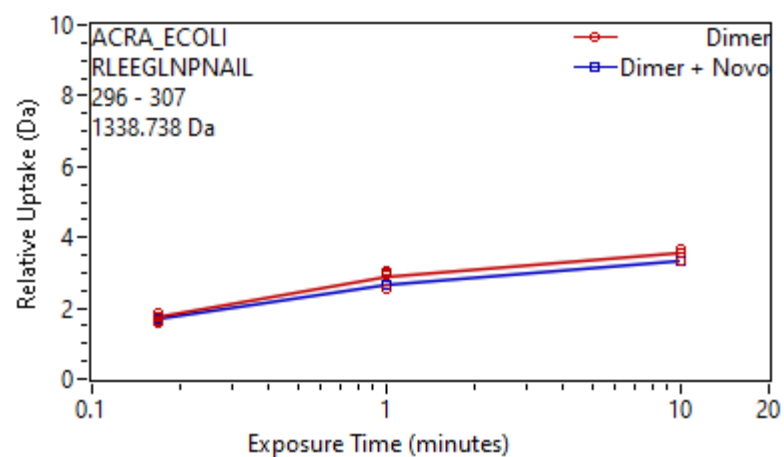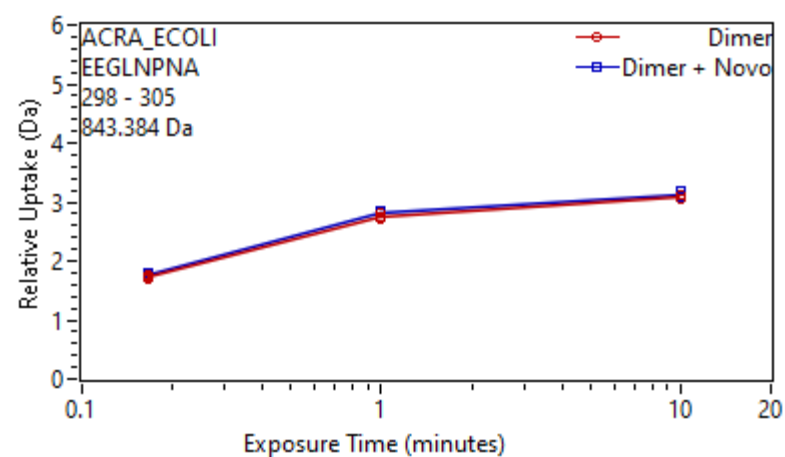

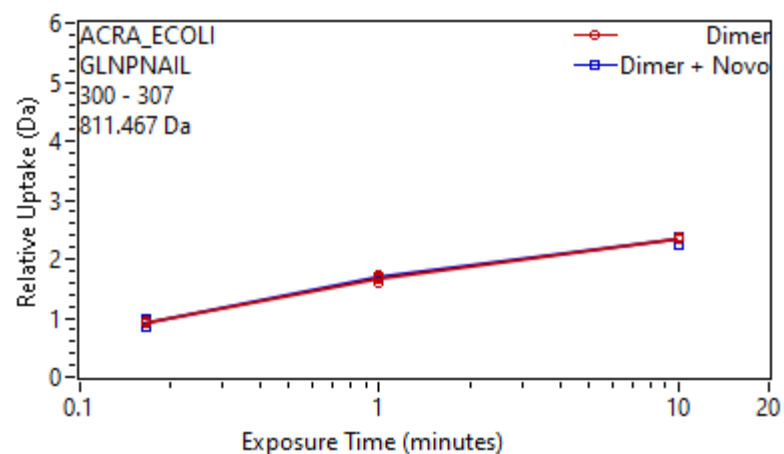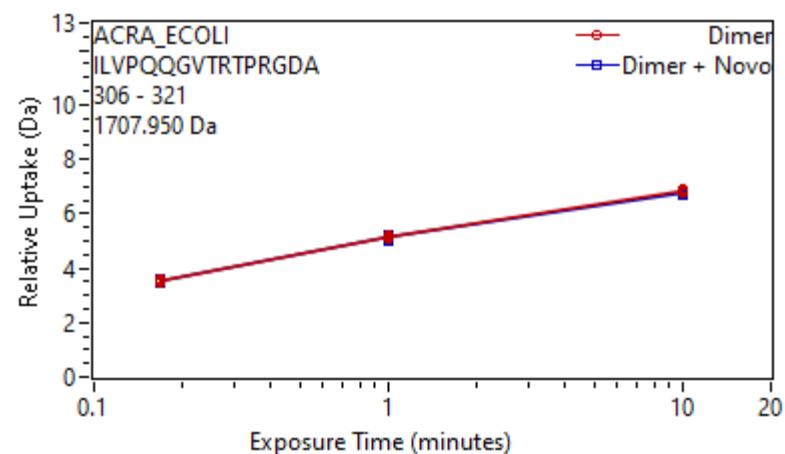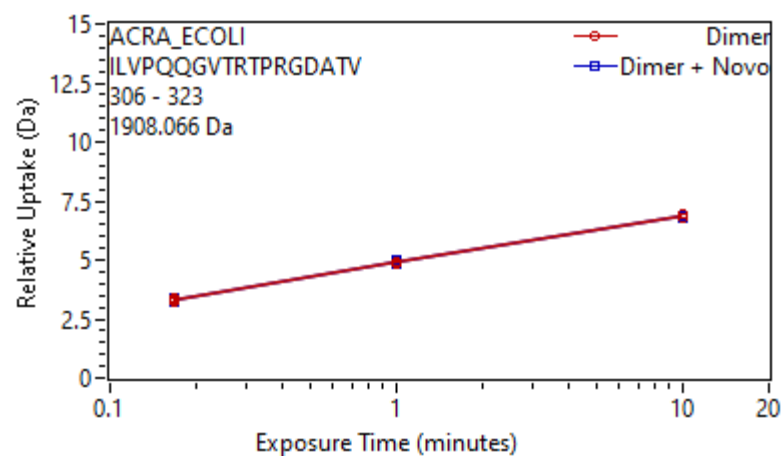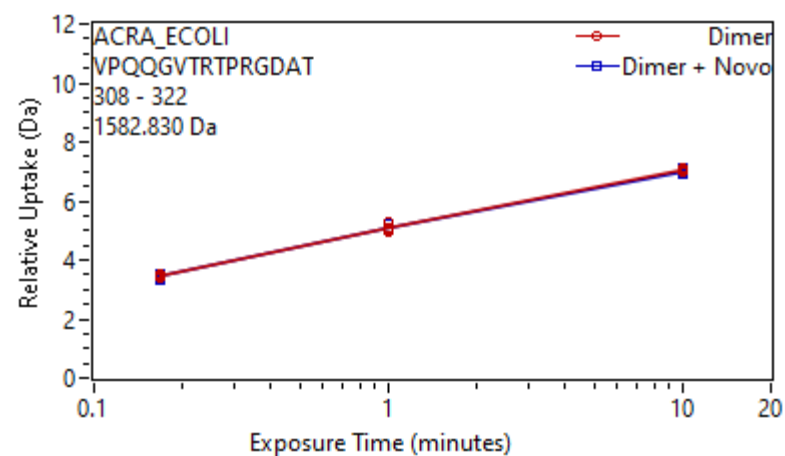

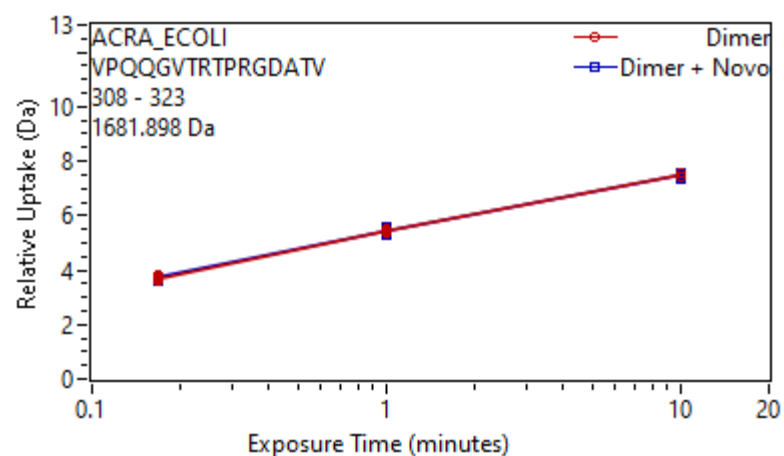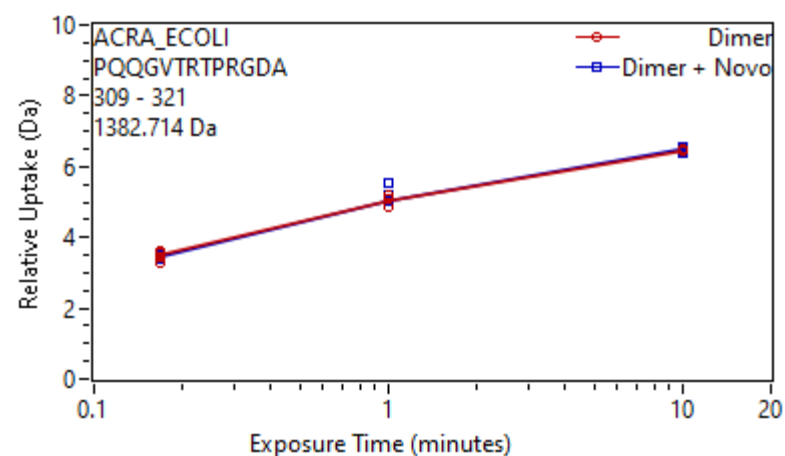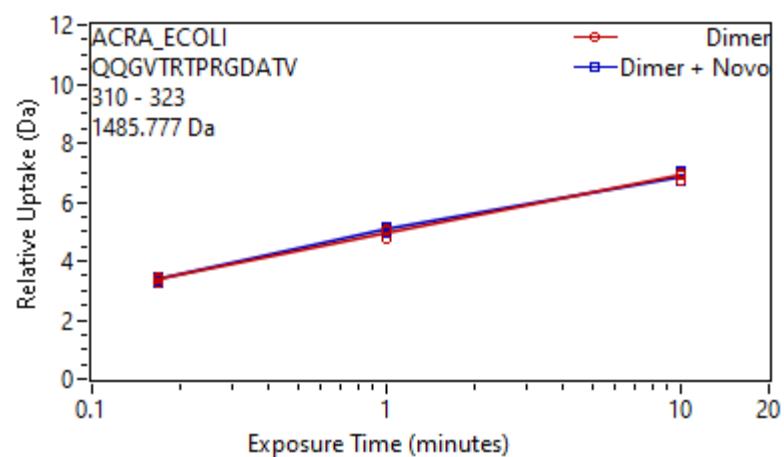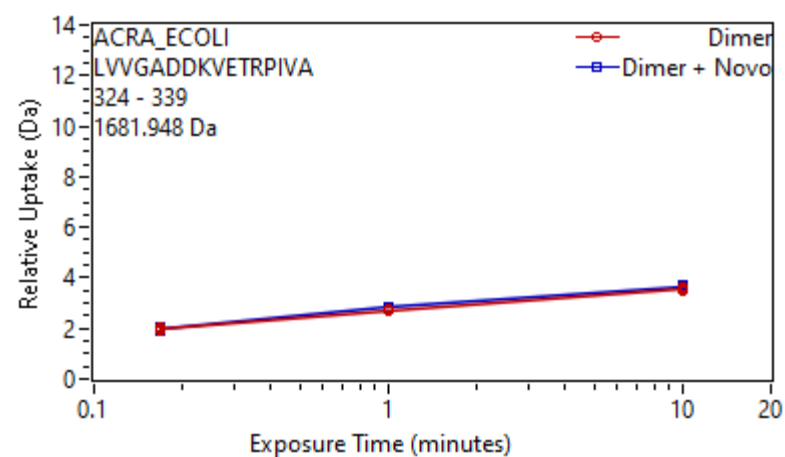

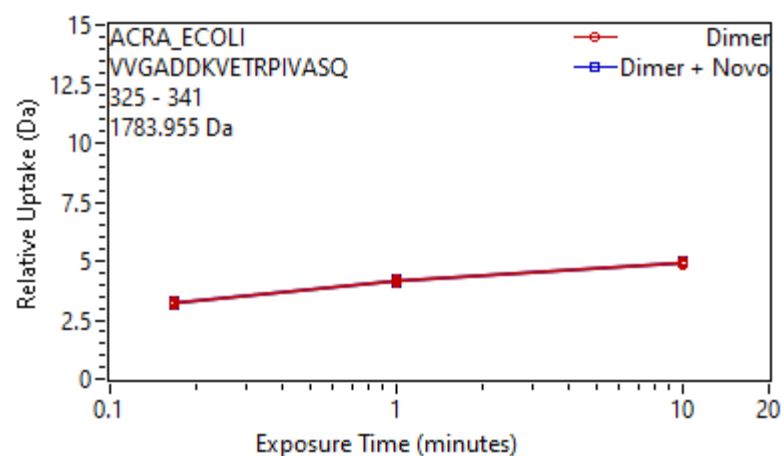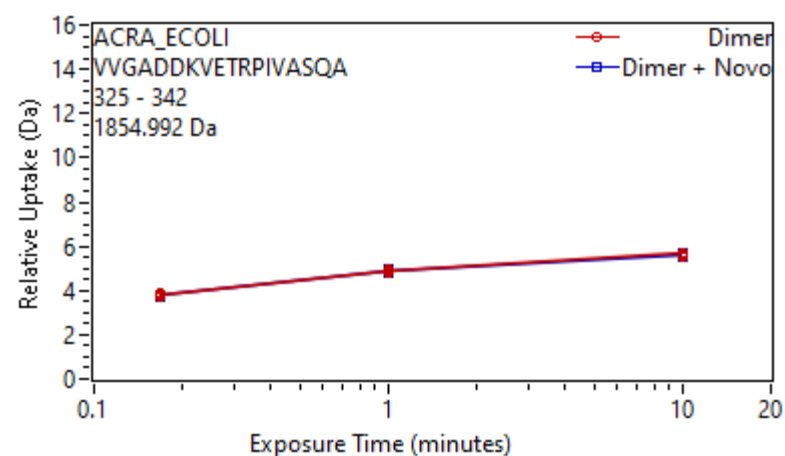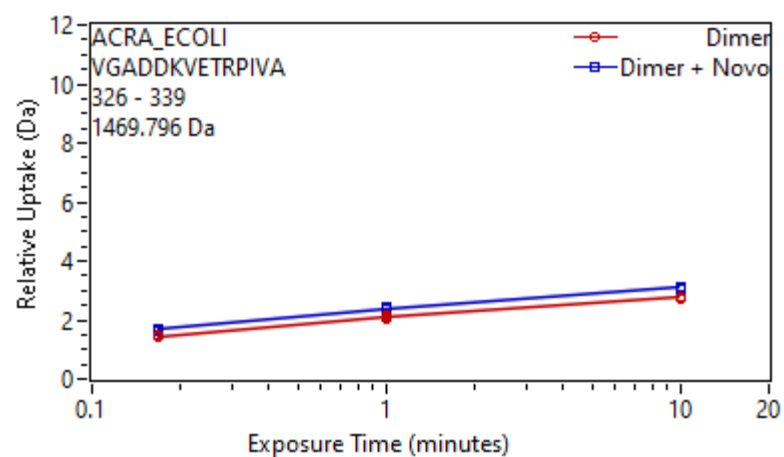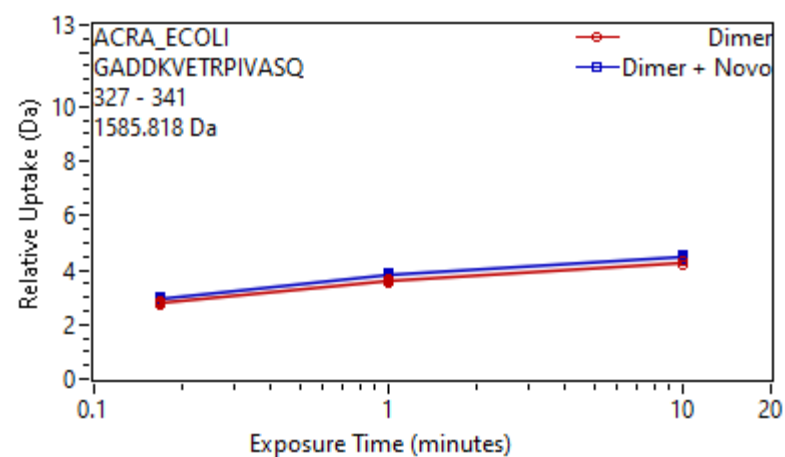

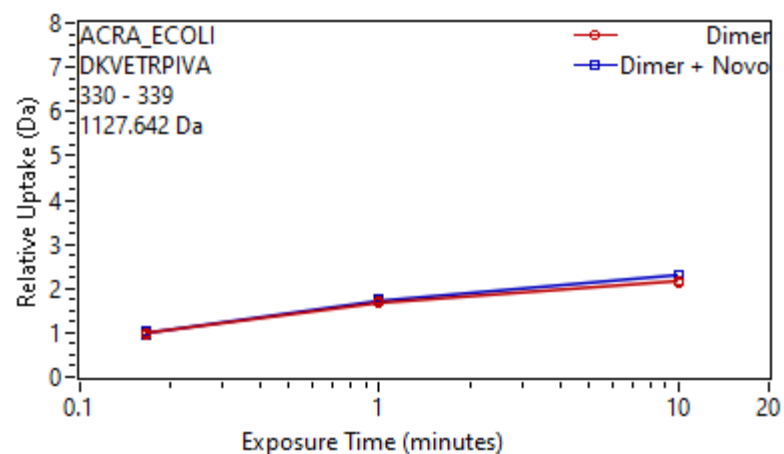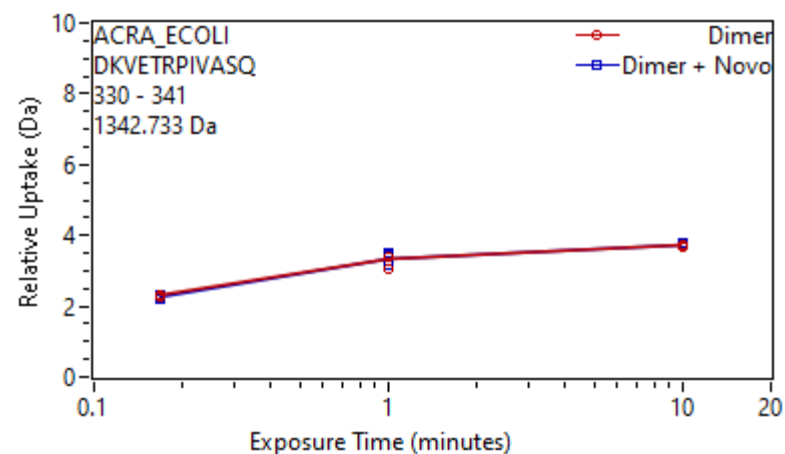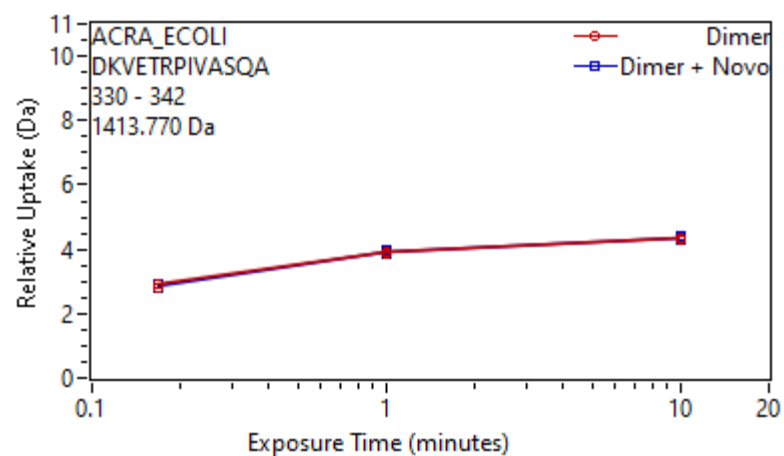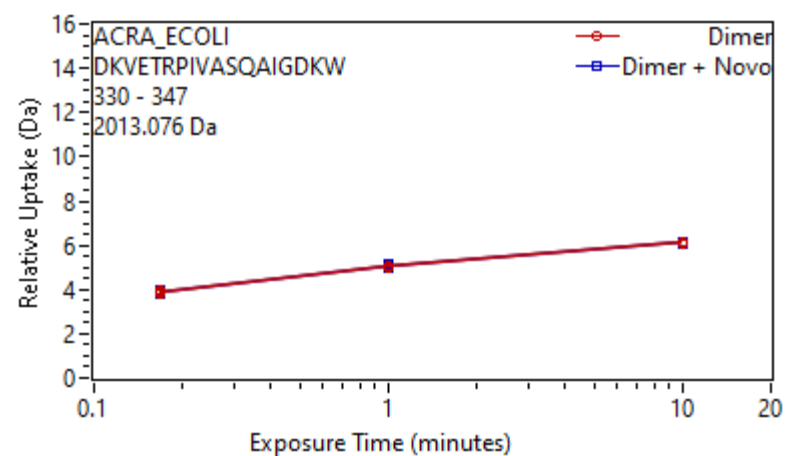

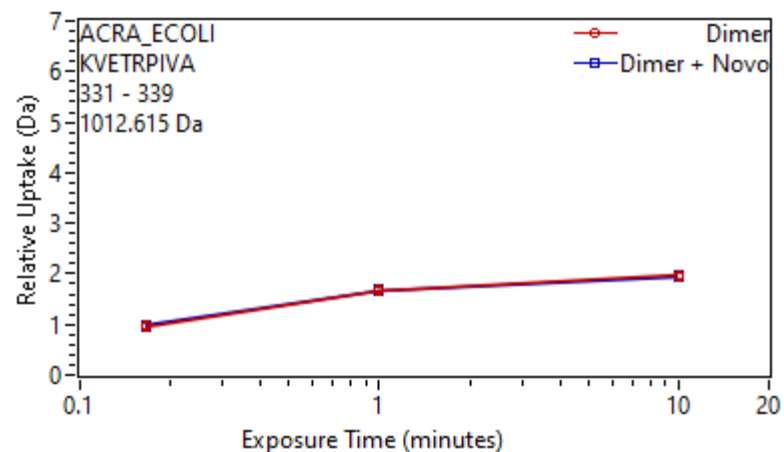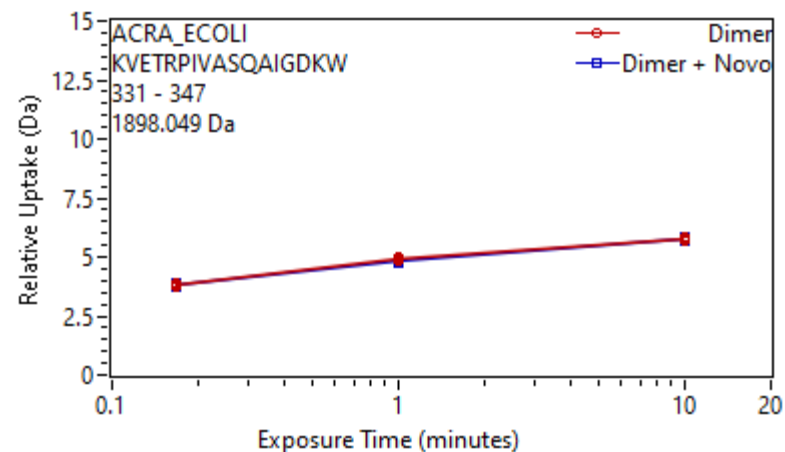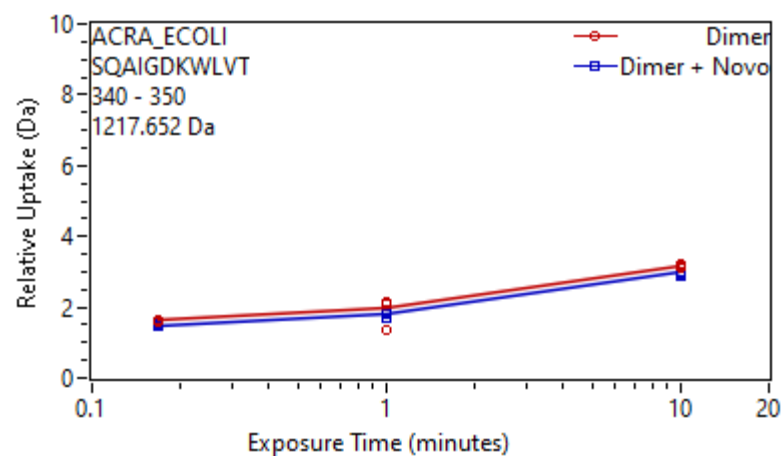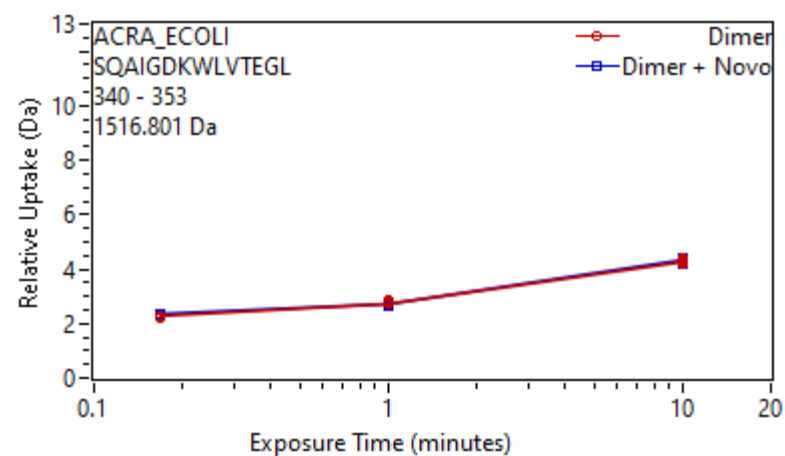

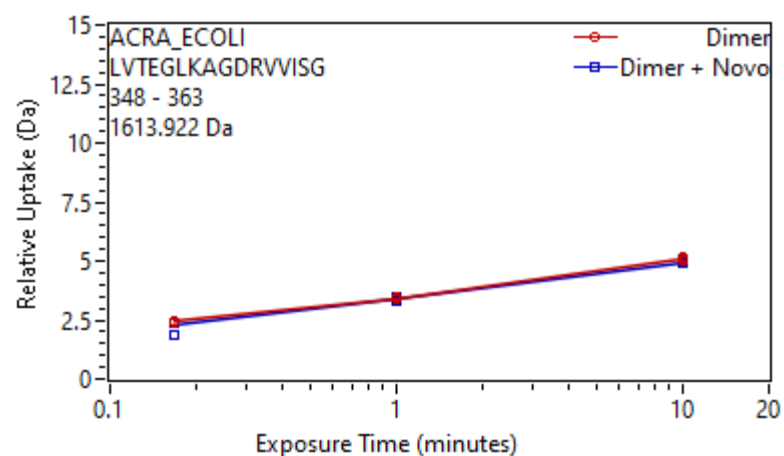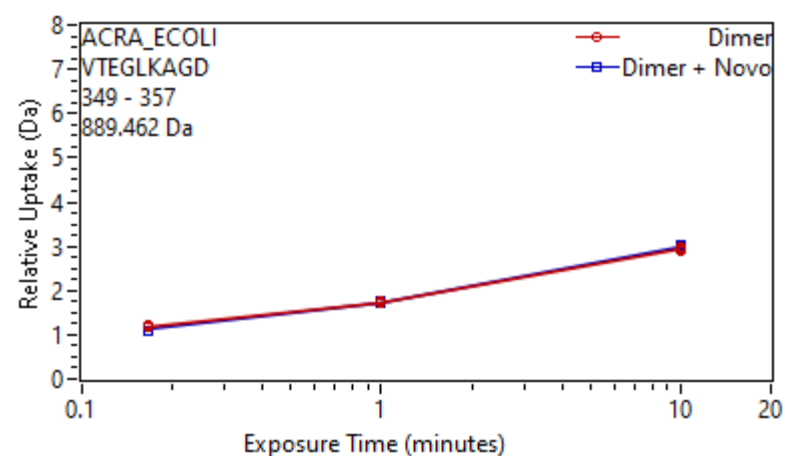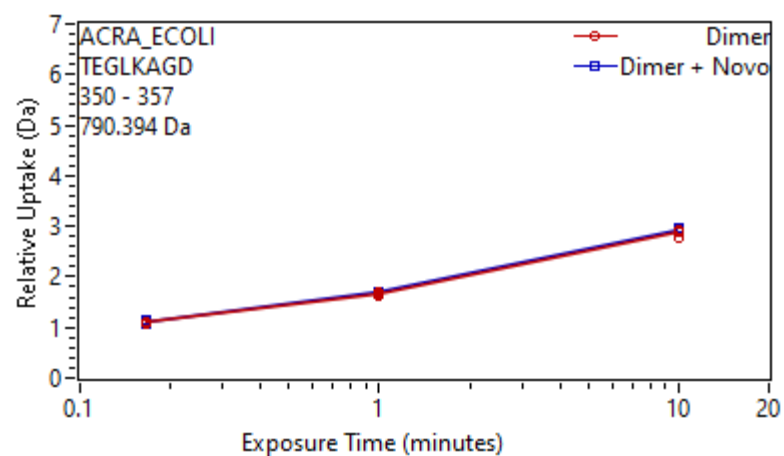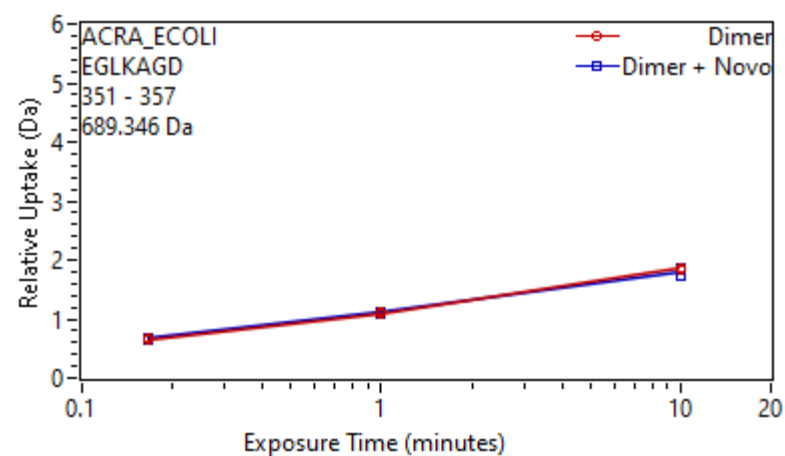

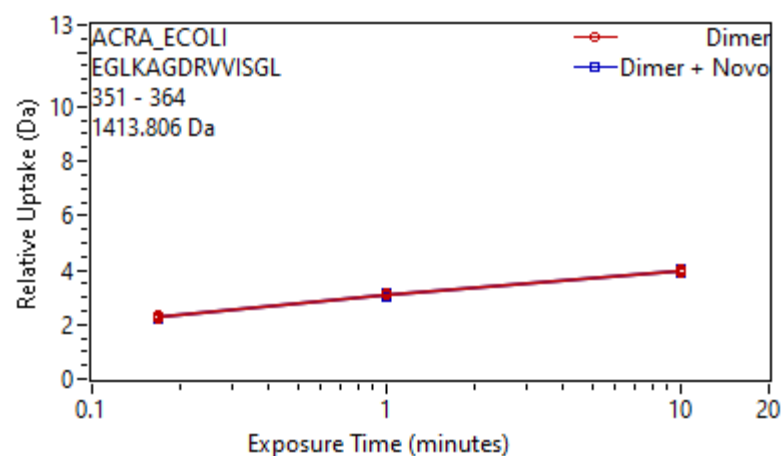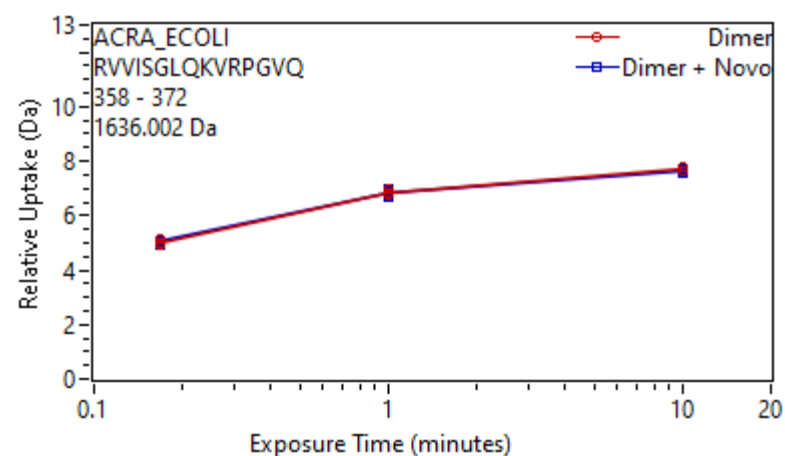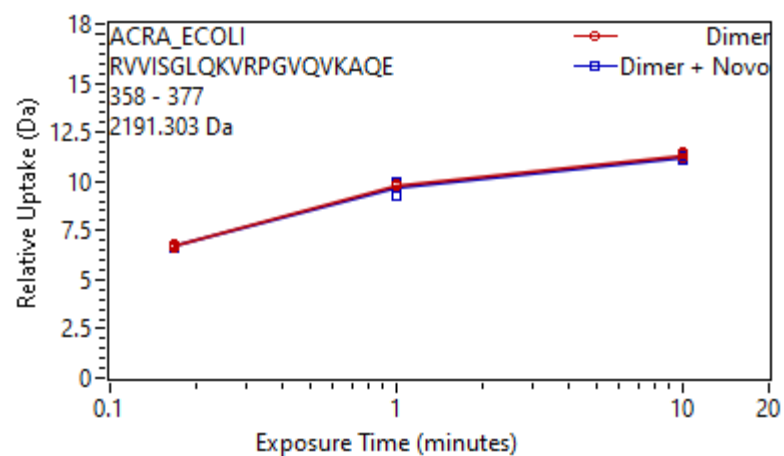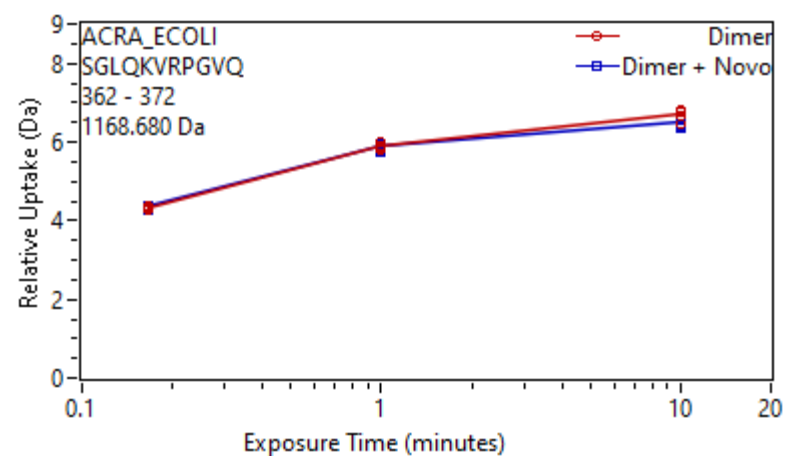

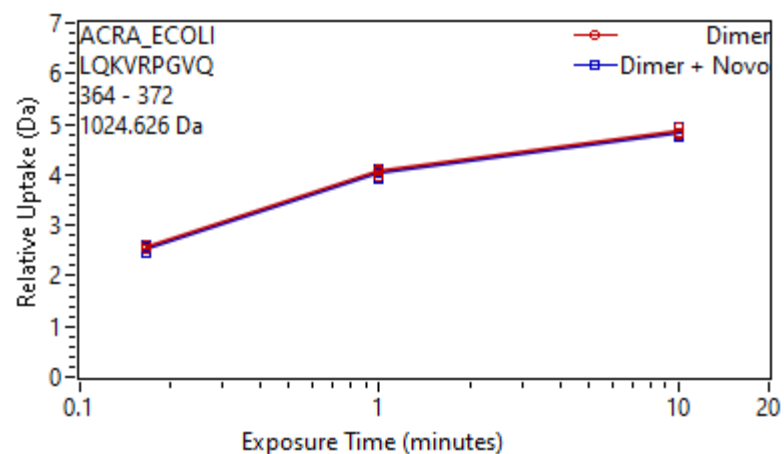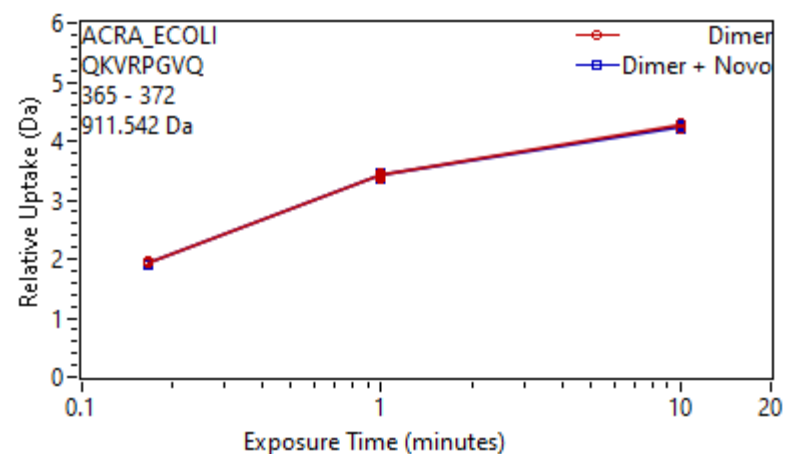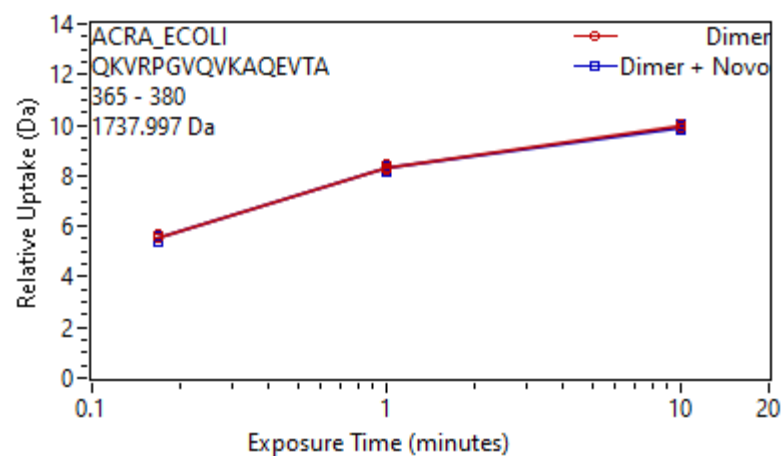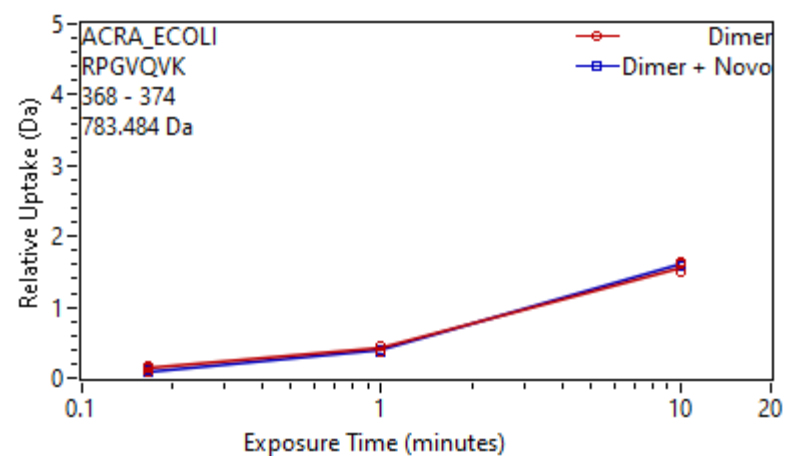

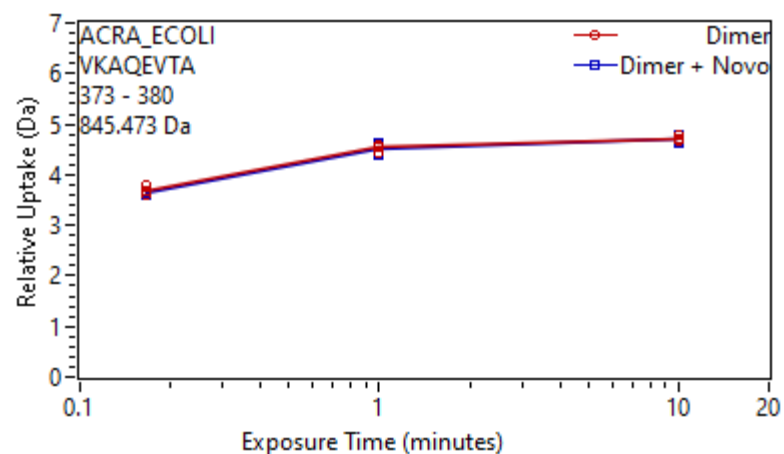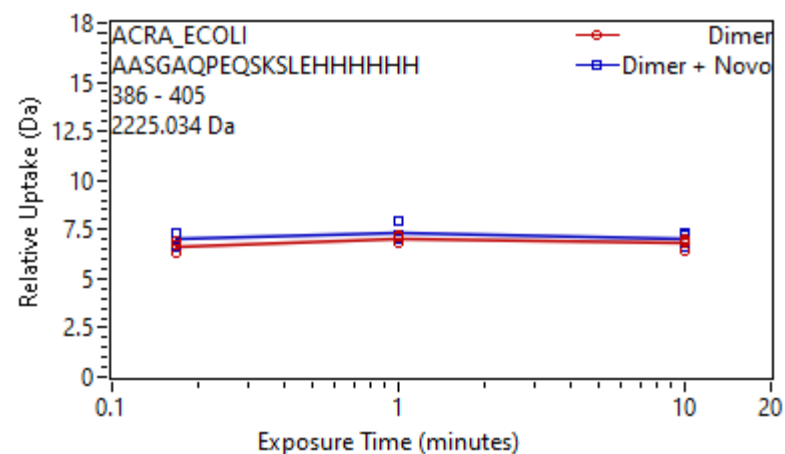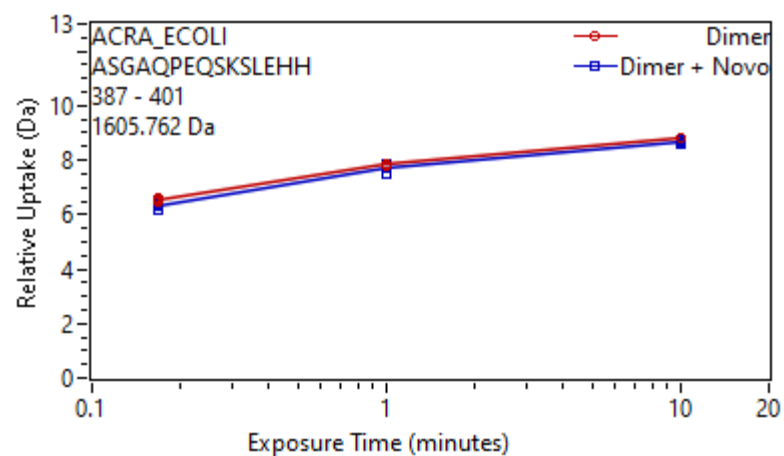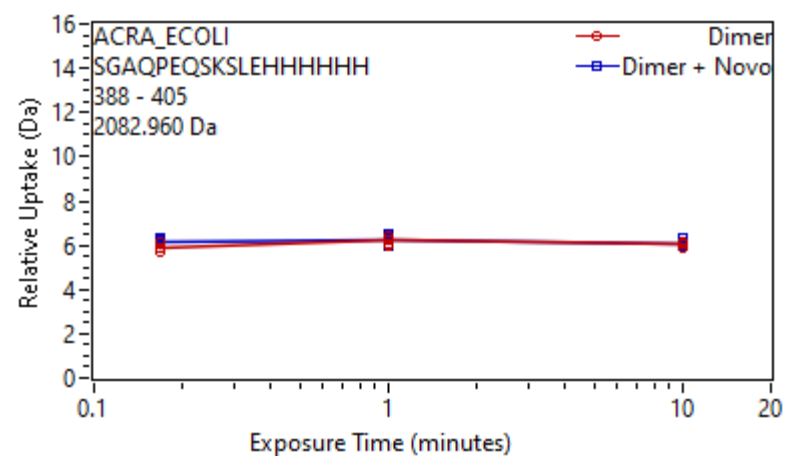

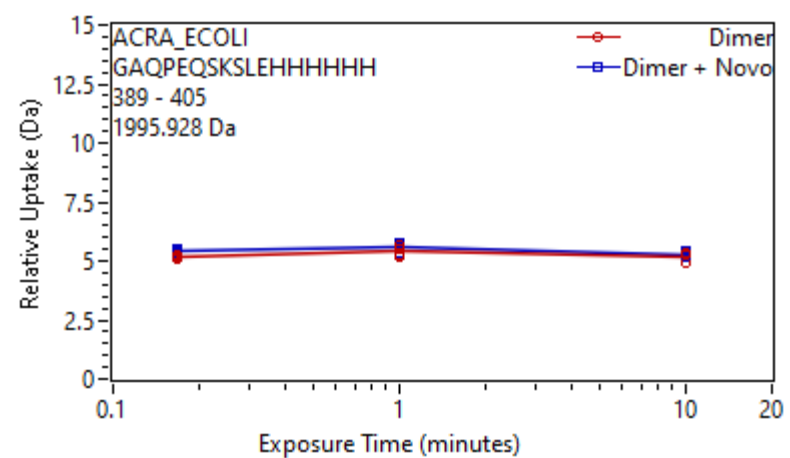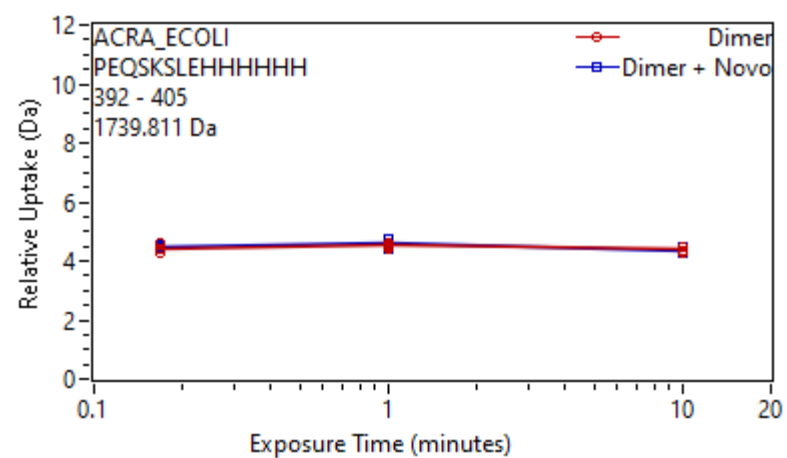

Supplement: Supplementary file 6 — Supplementary Data 3 [file 41467_2023_39615_MOESM6_ESM.zip › Supplementary Data 3/AcrAsd_novobiocin_uptakeplots.pdf]

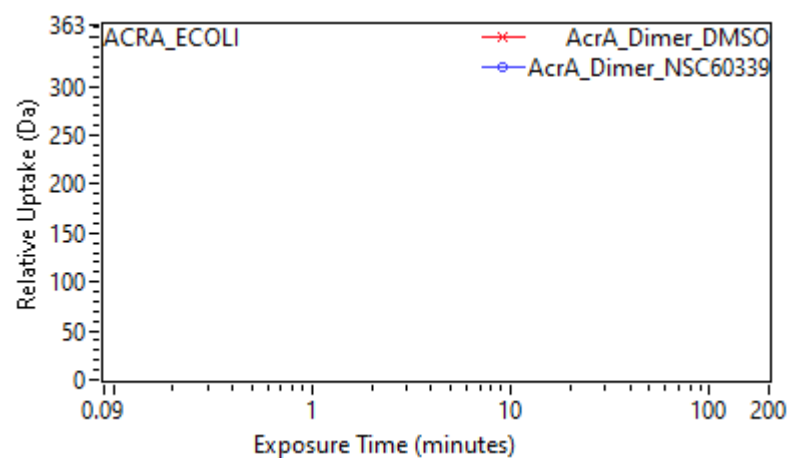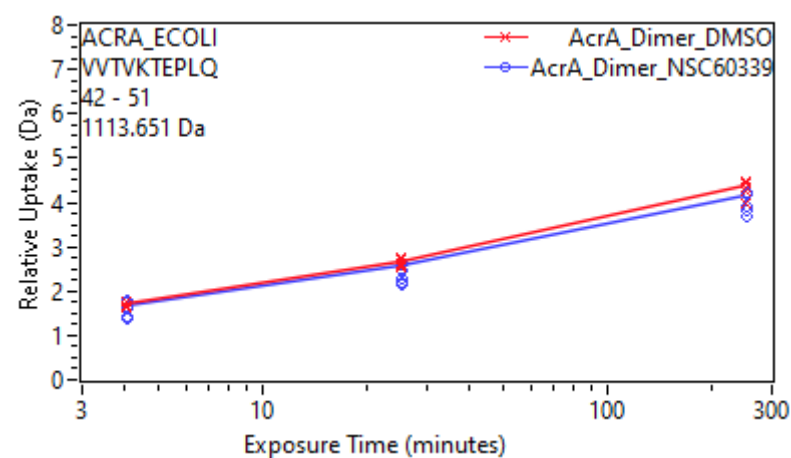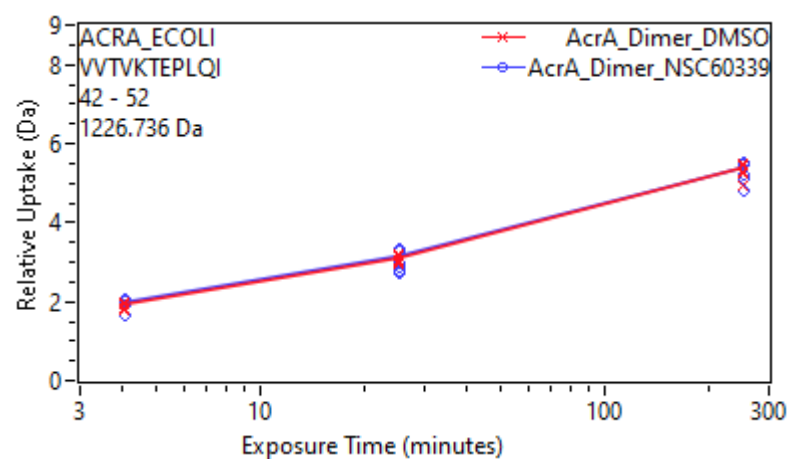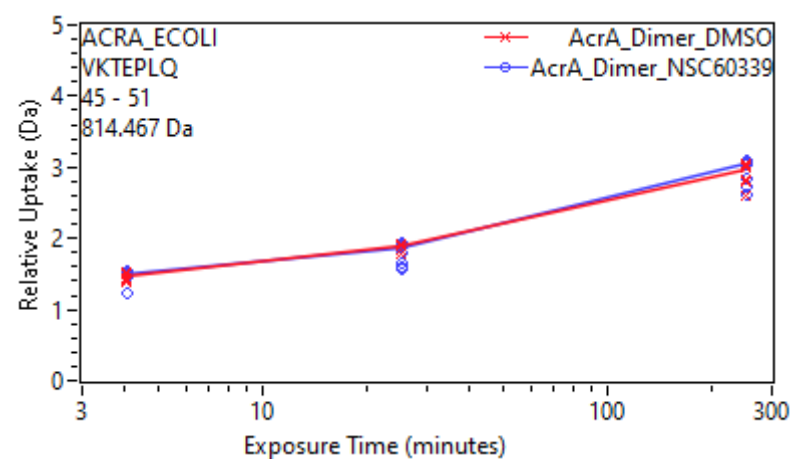

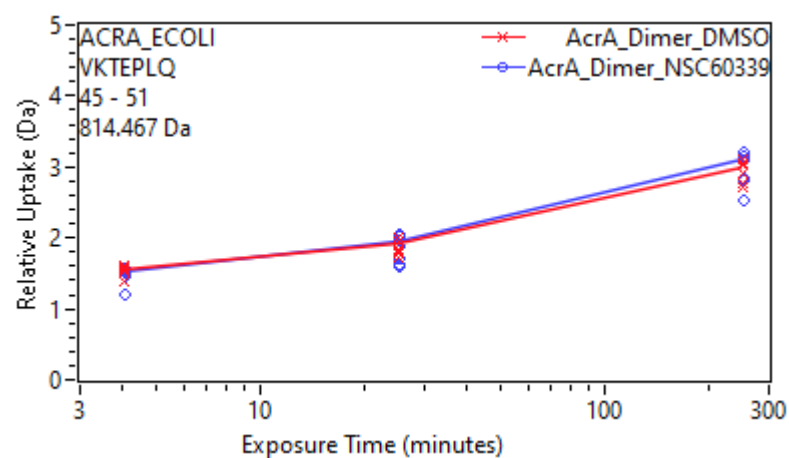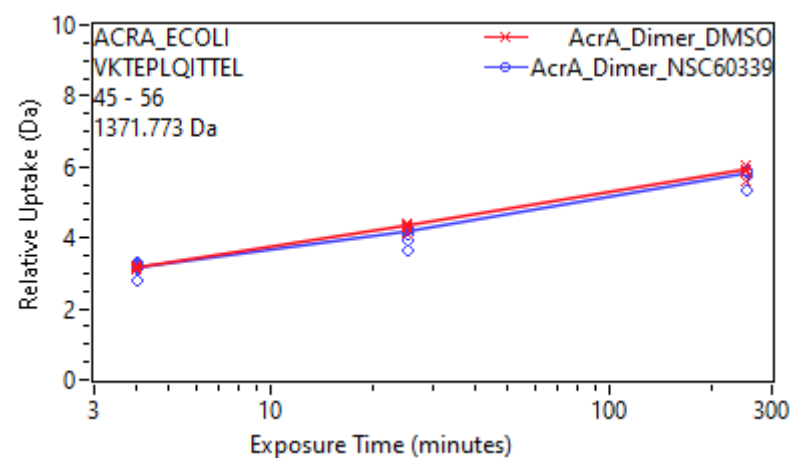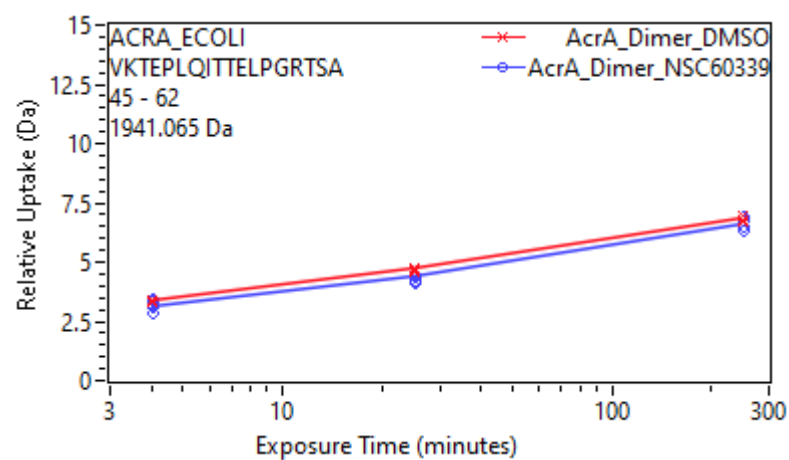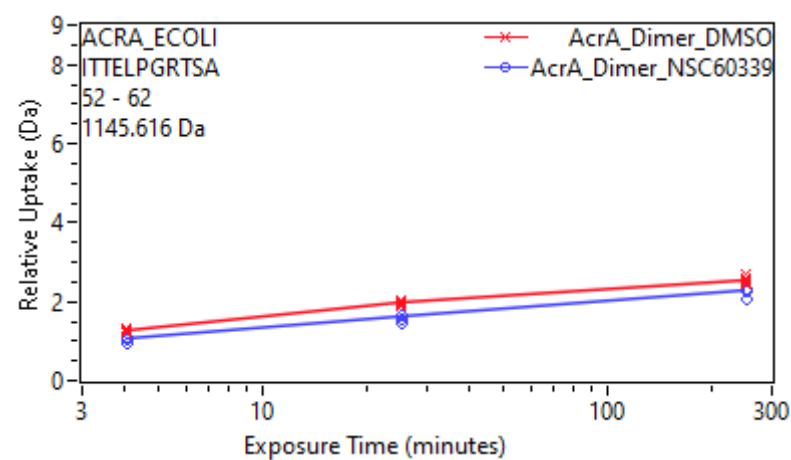

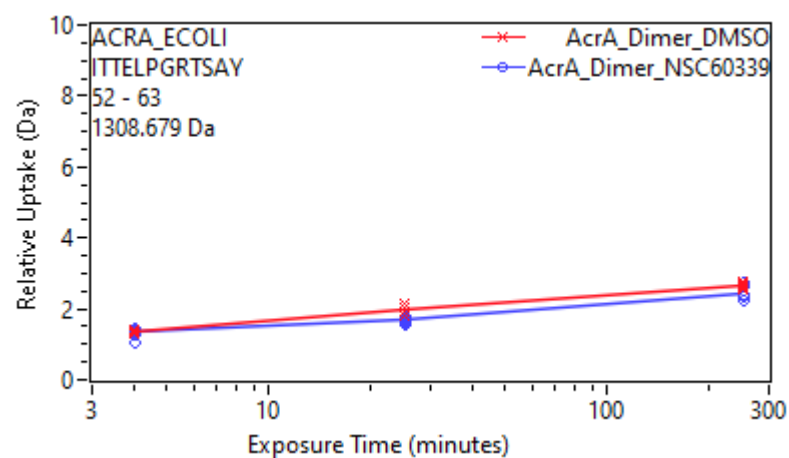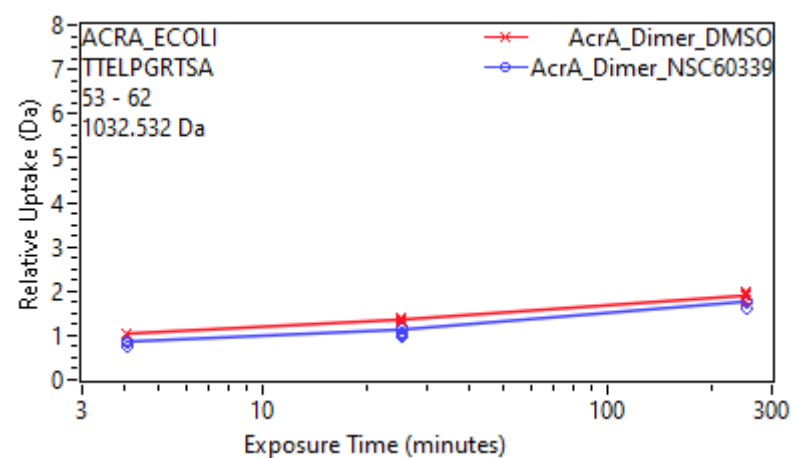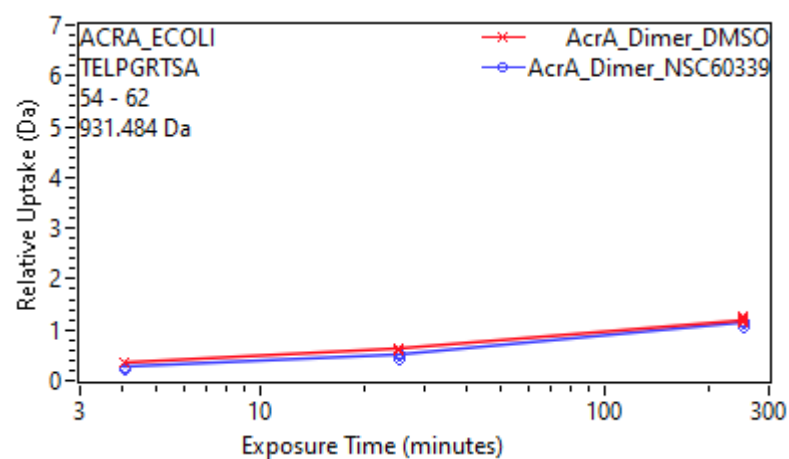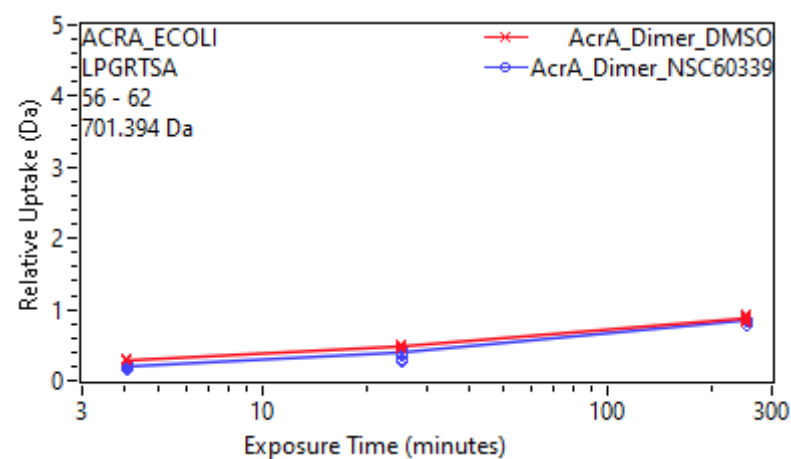

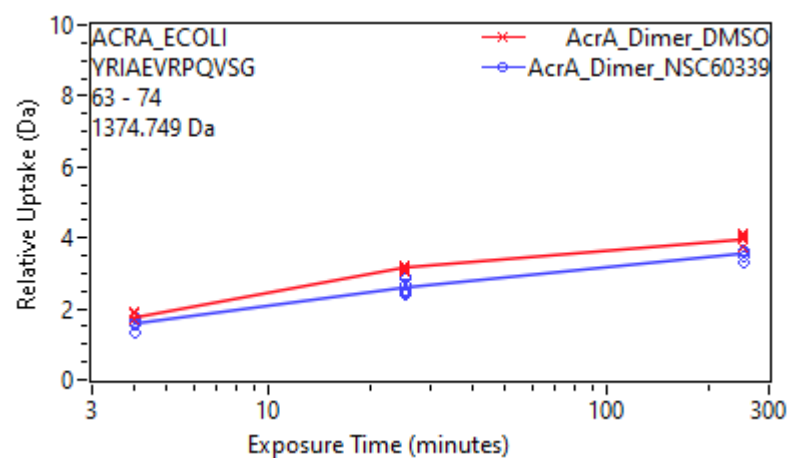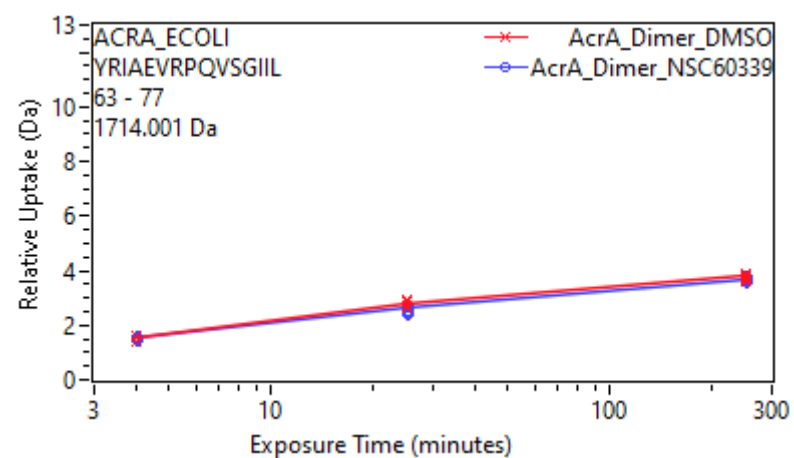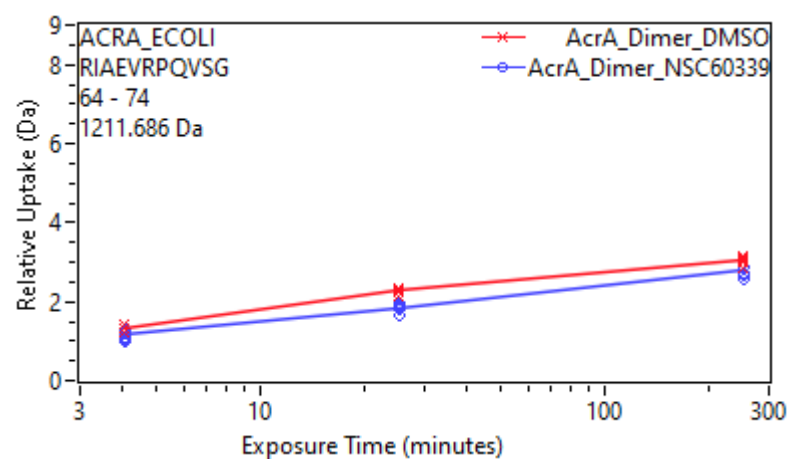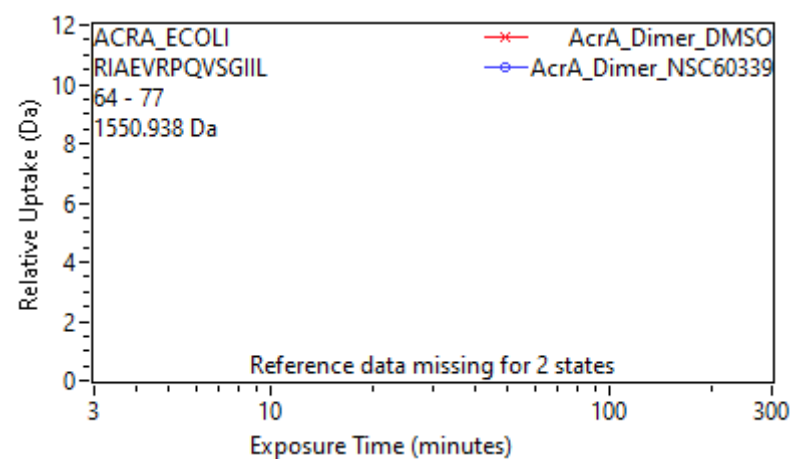

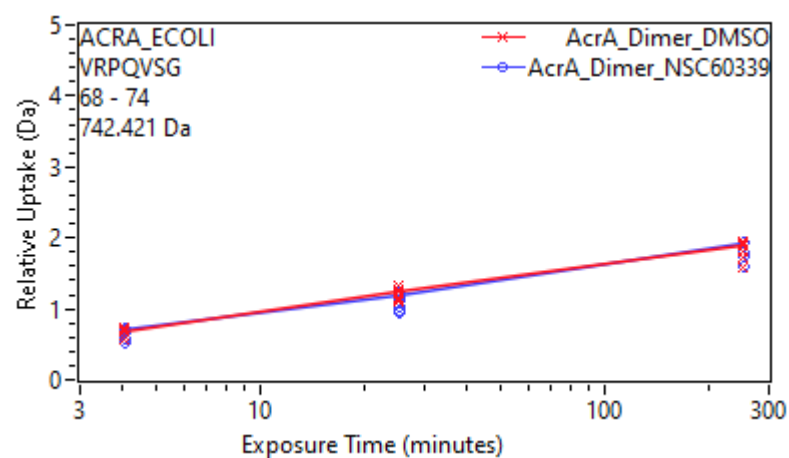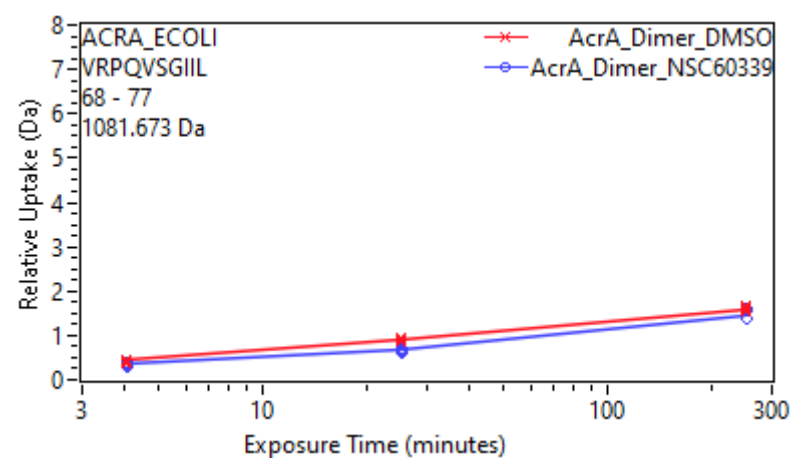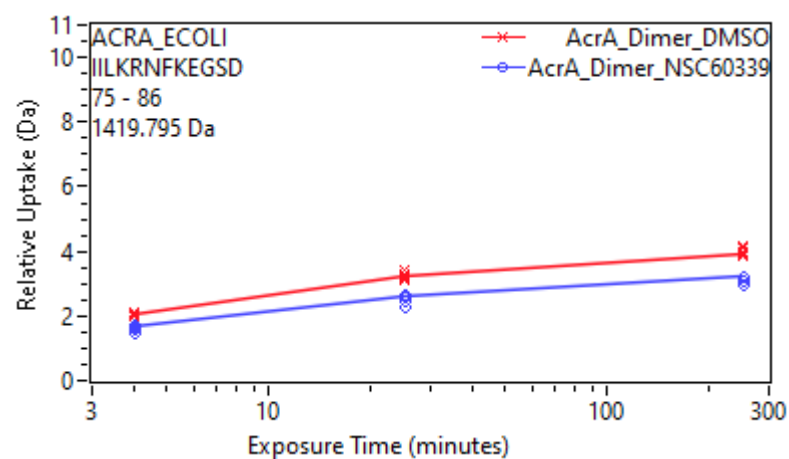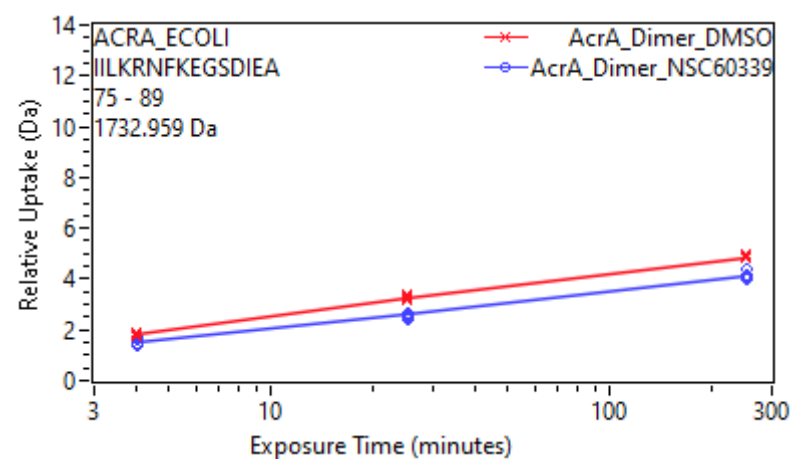

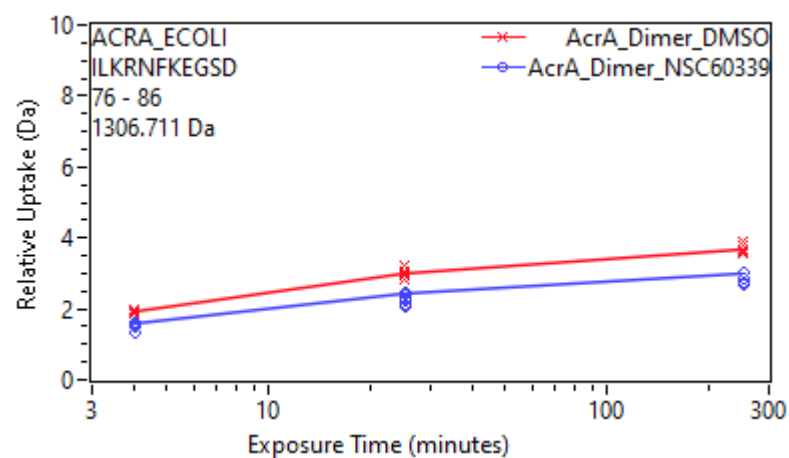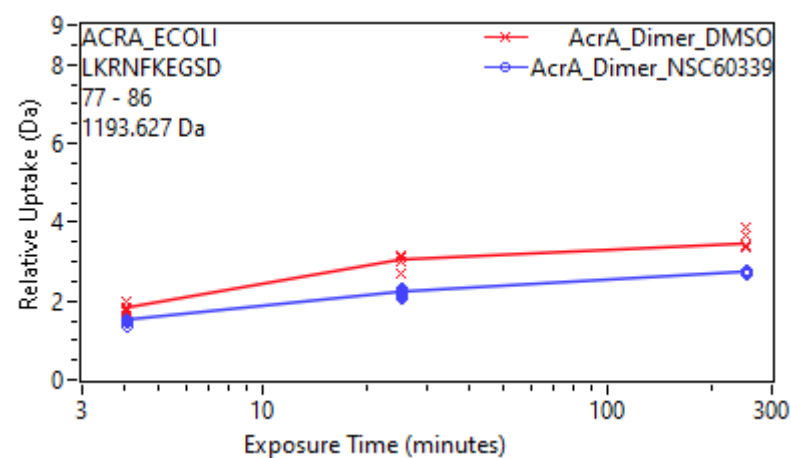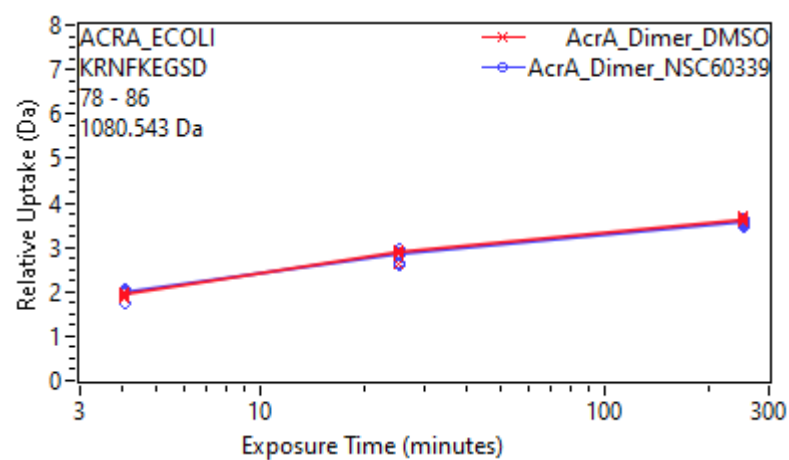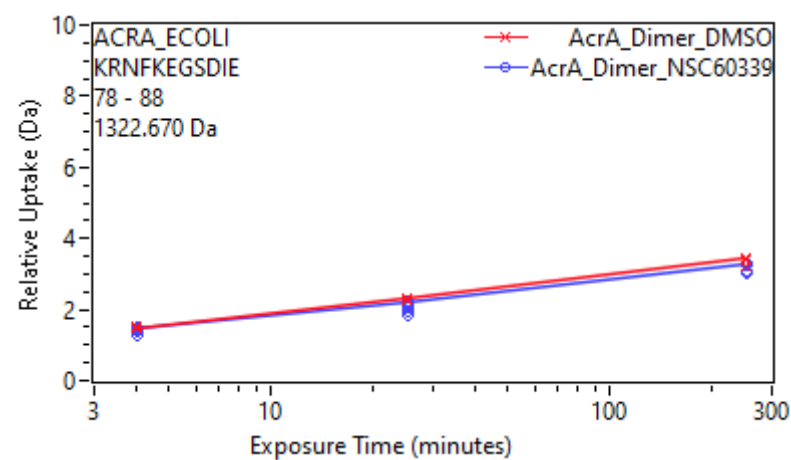

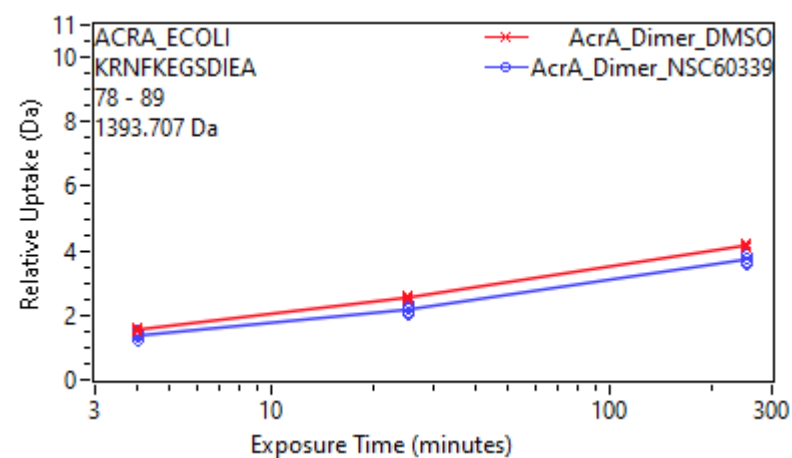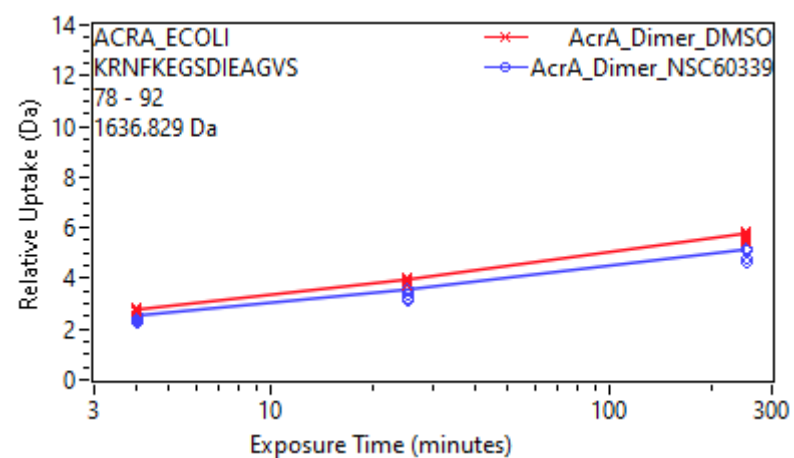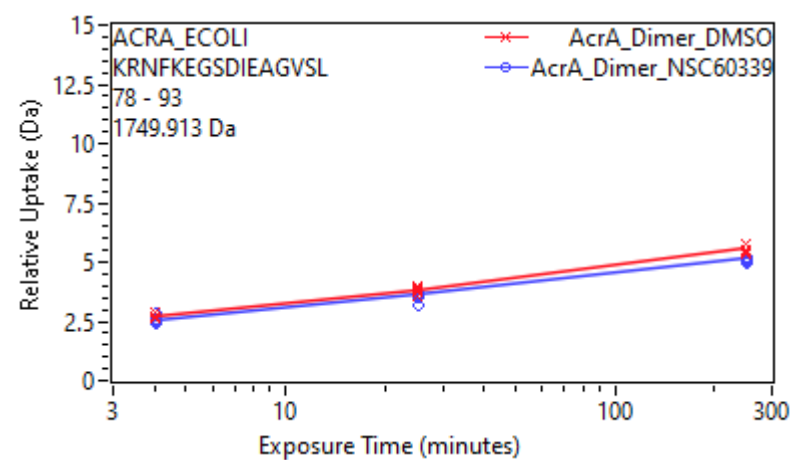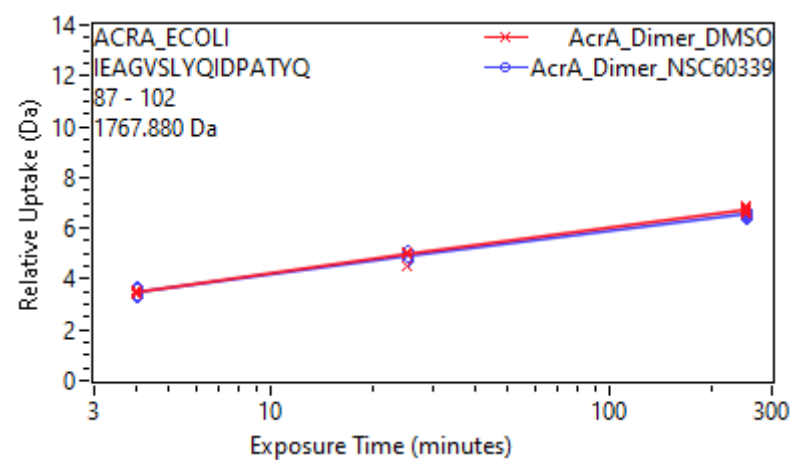

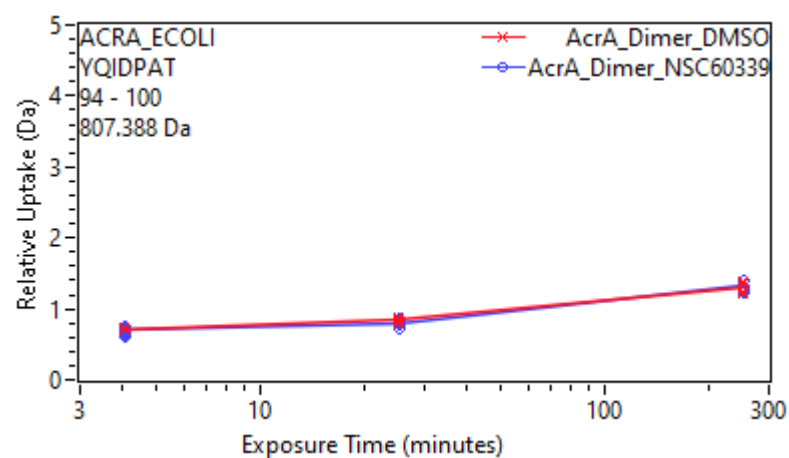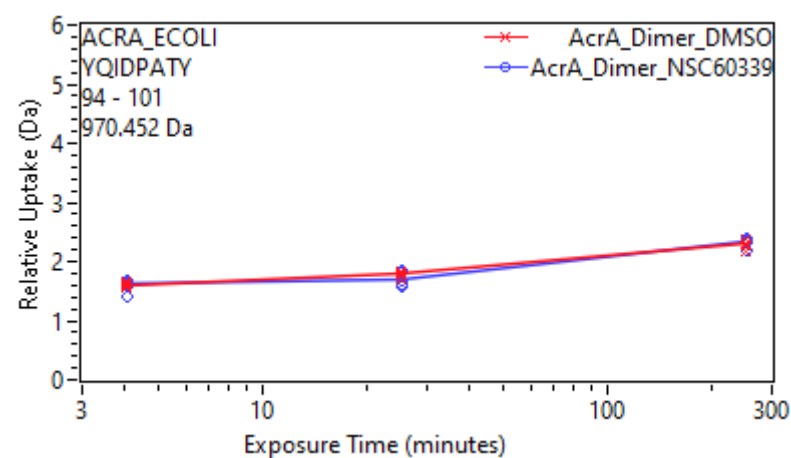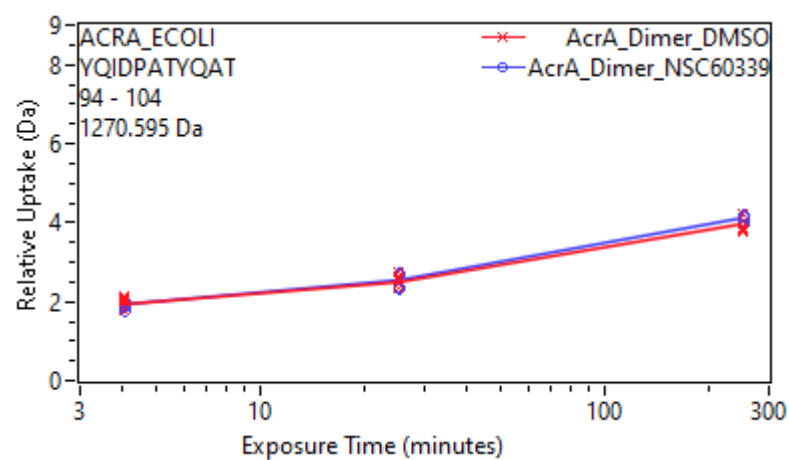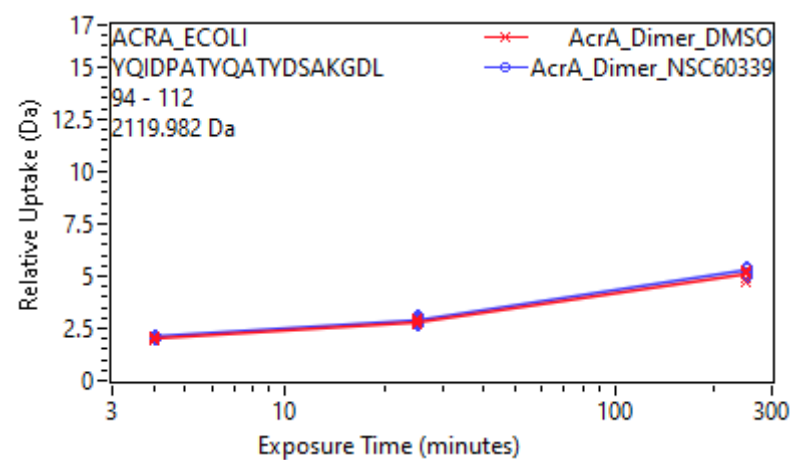

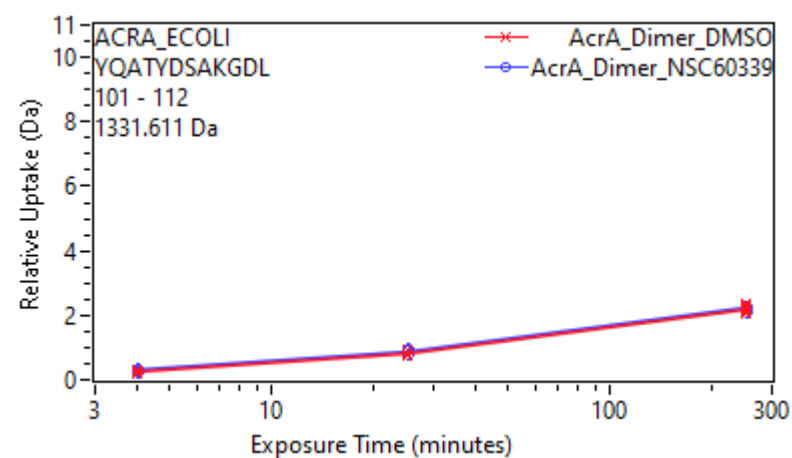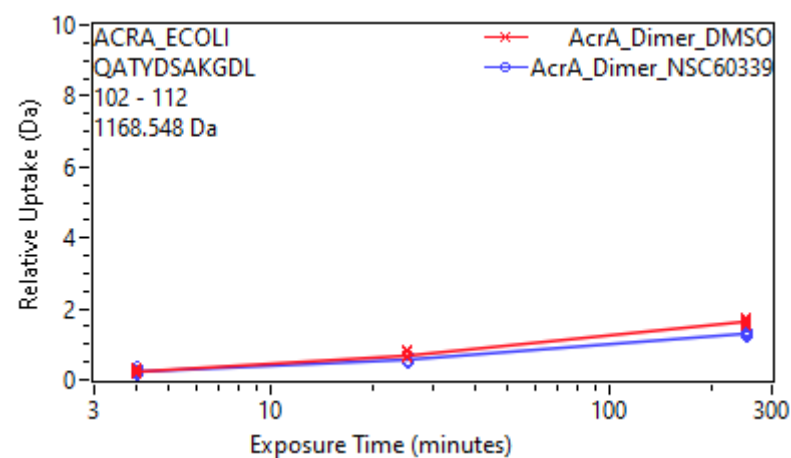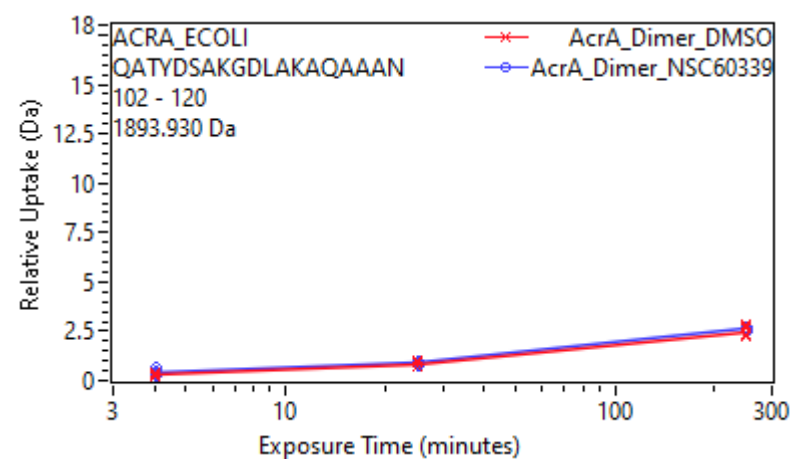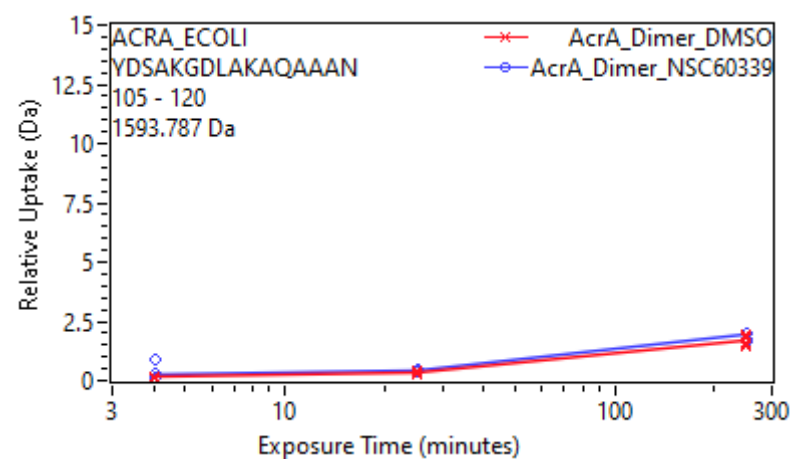

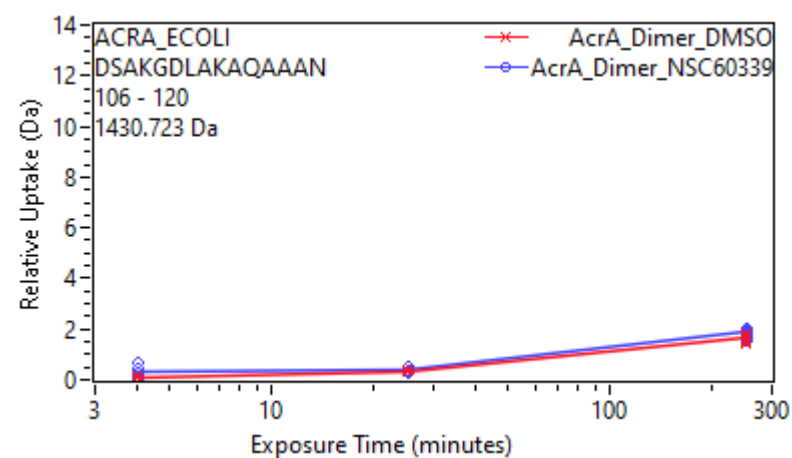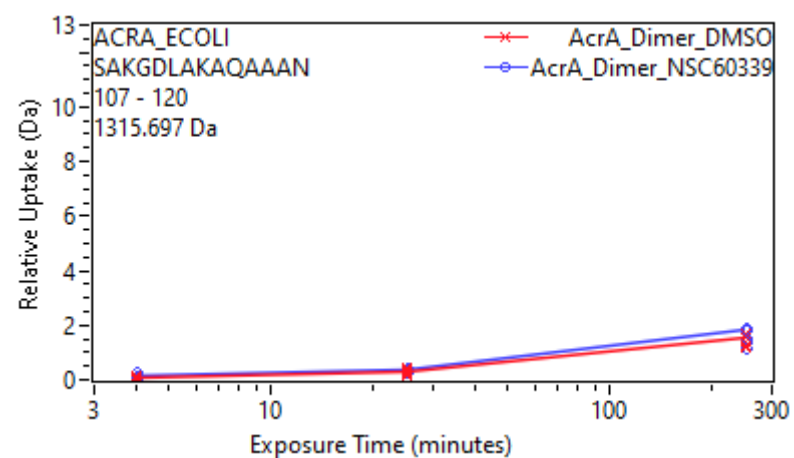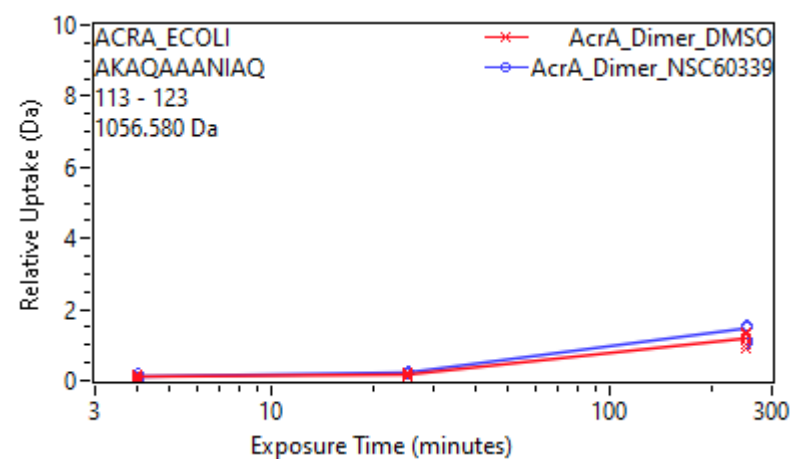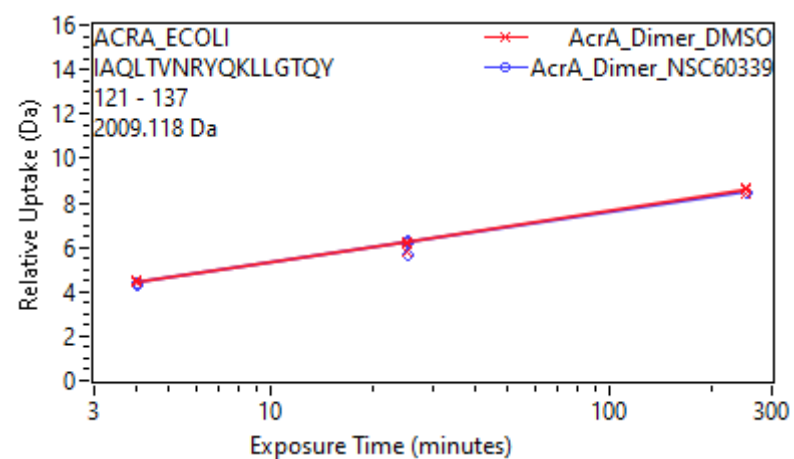

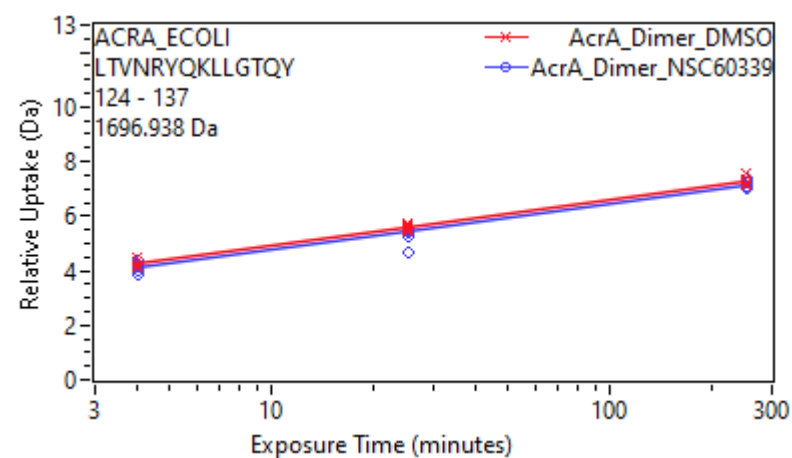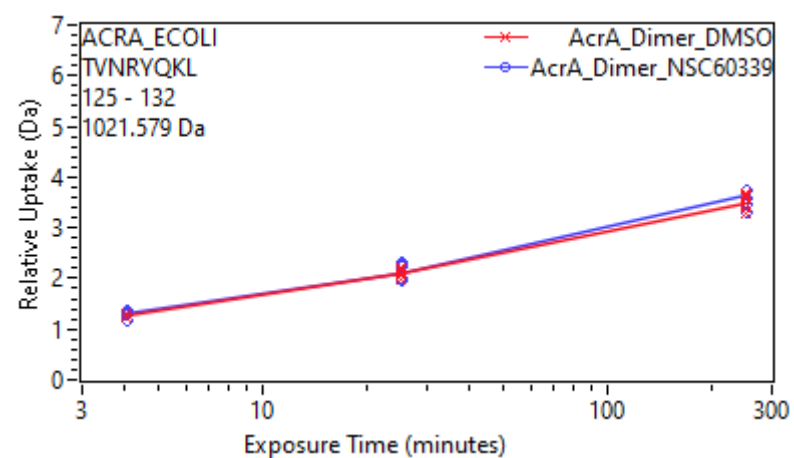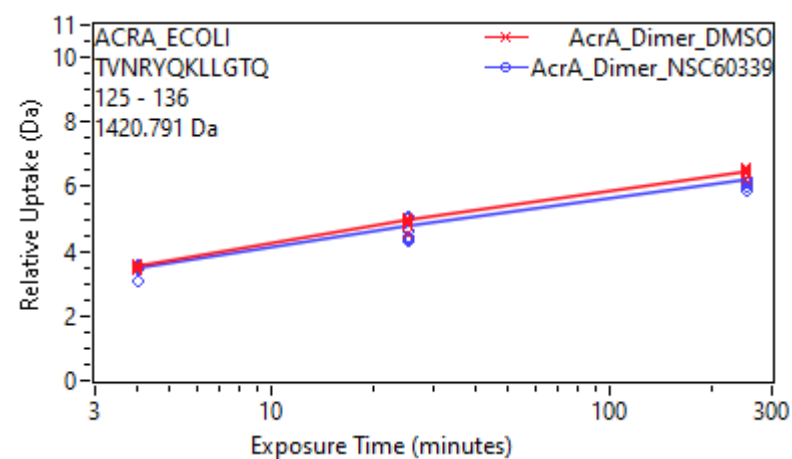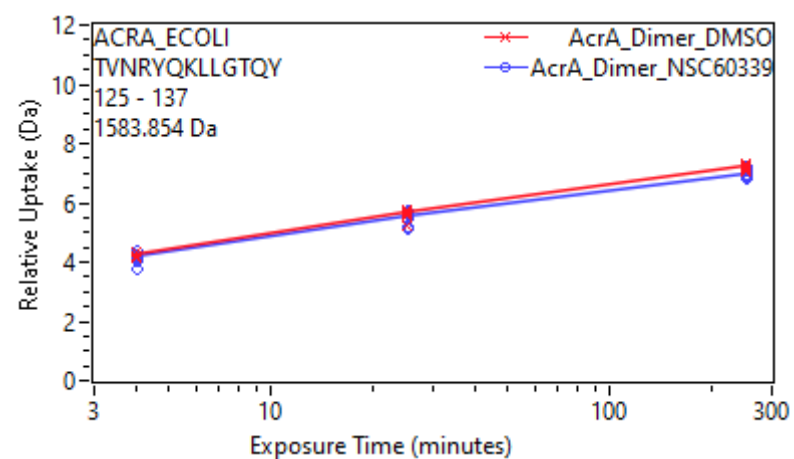

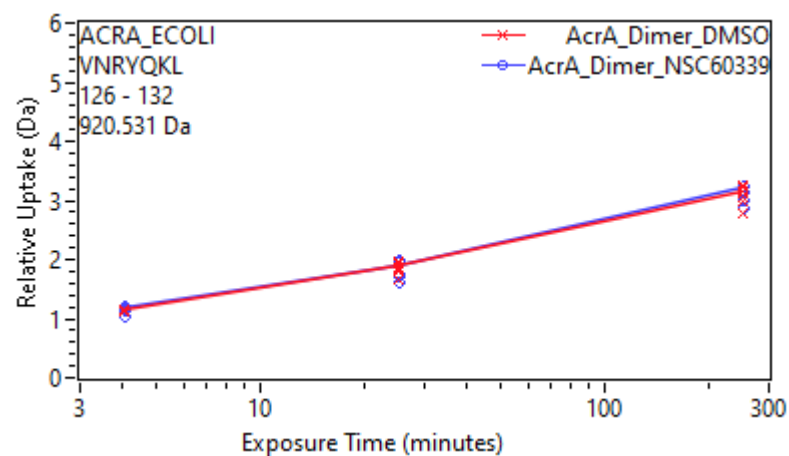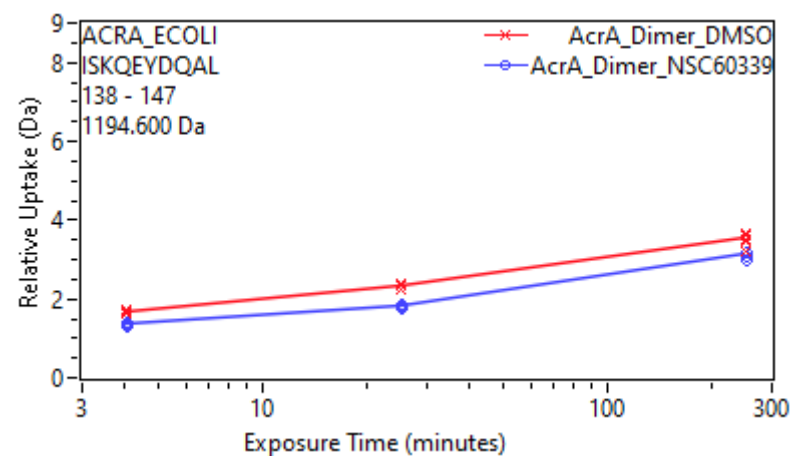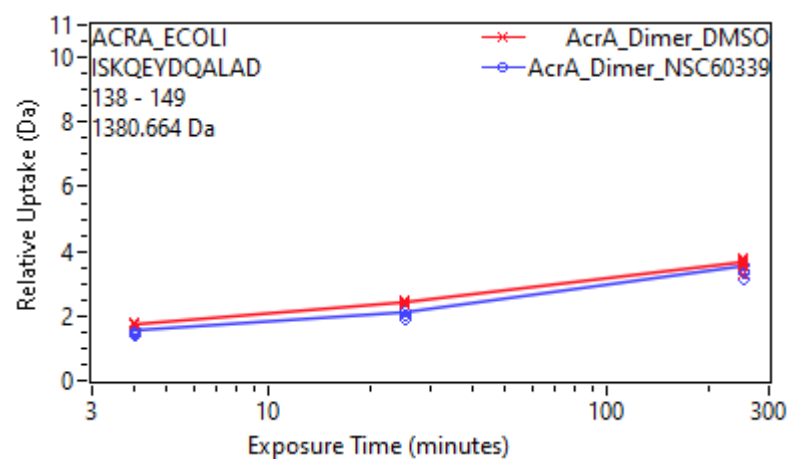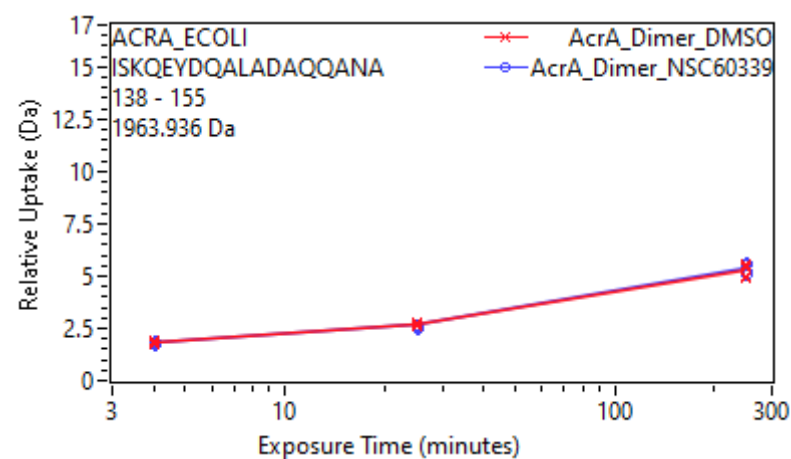

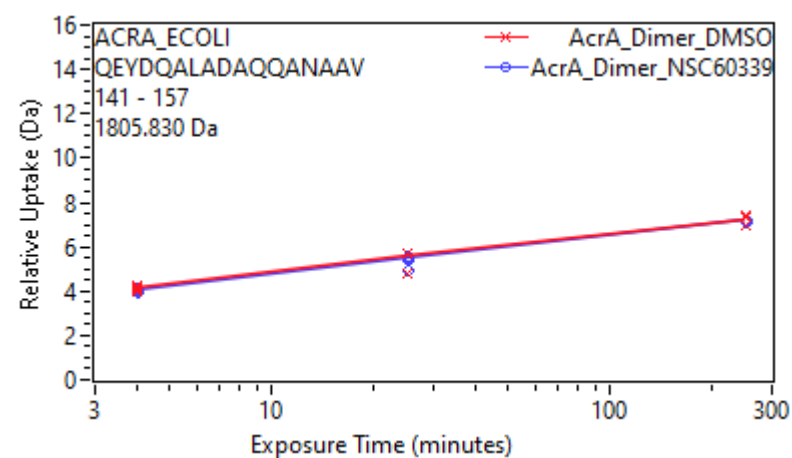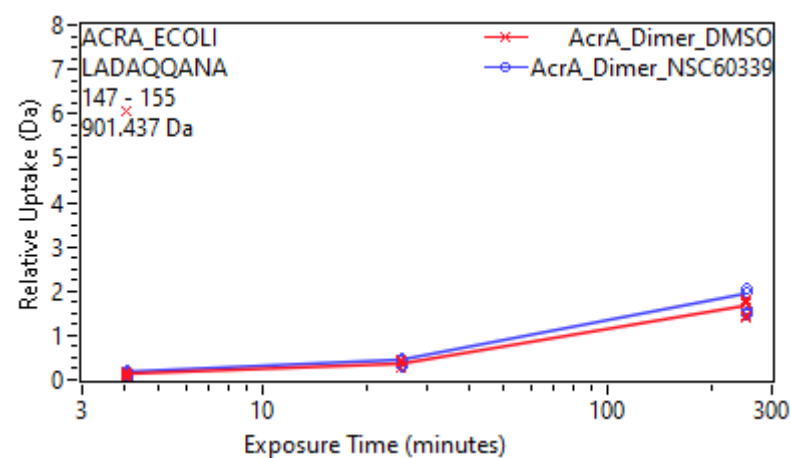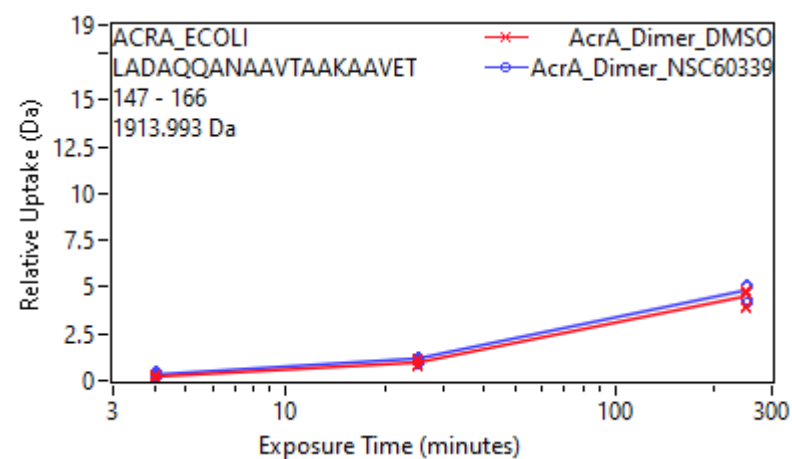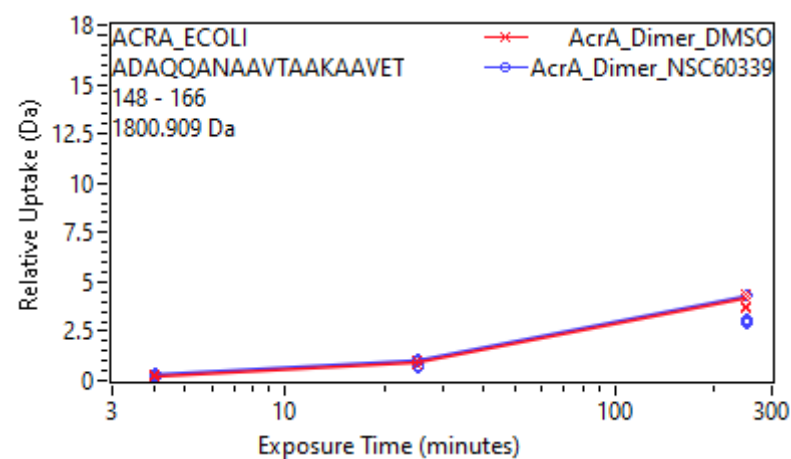

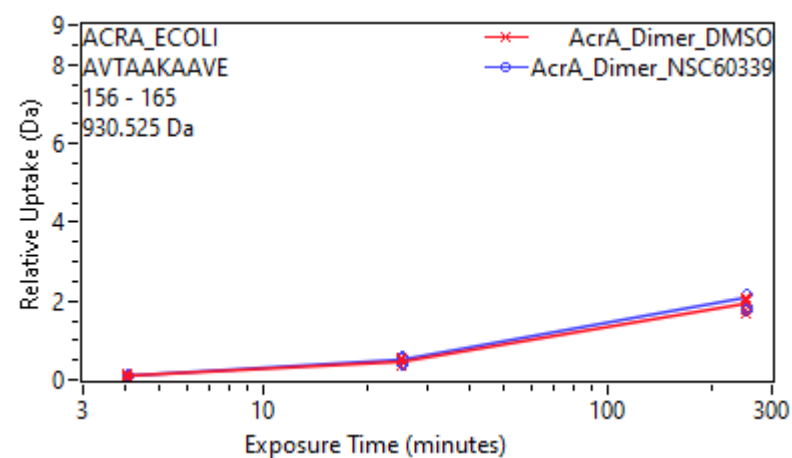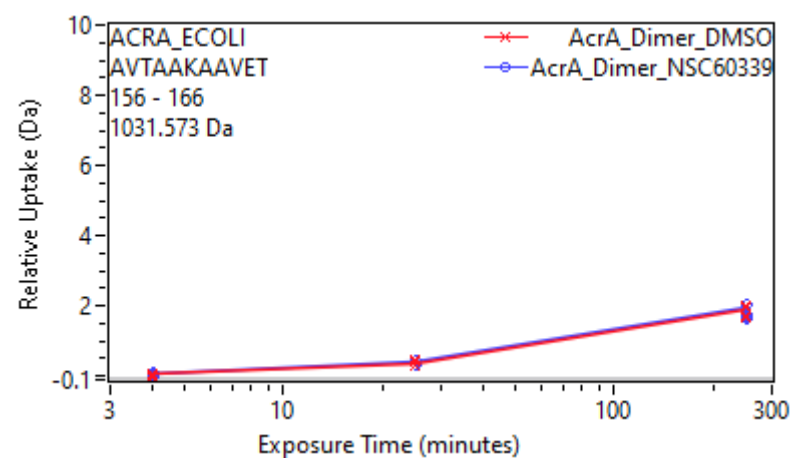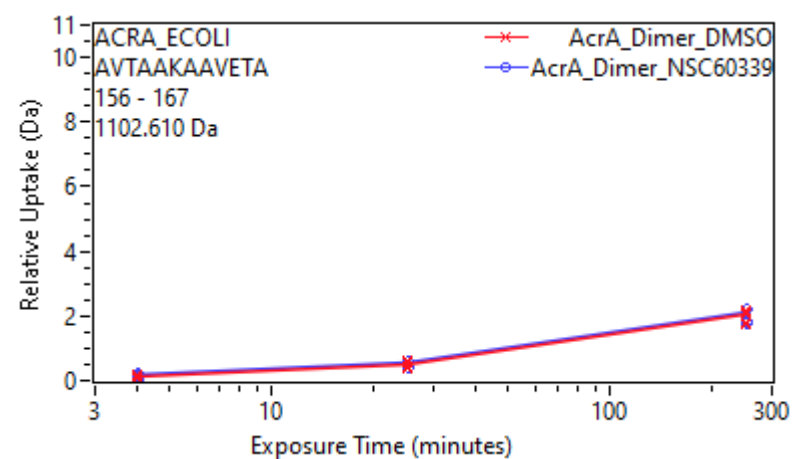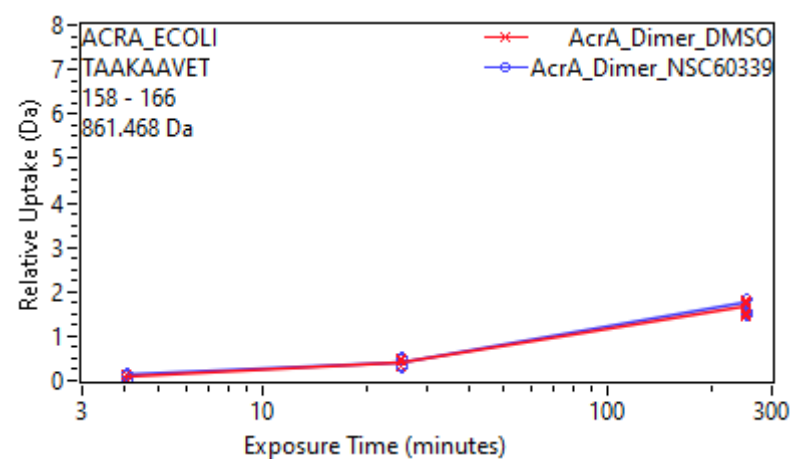

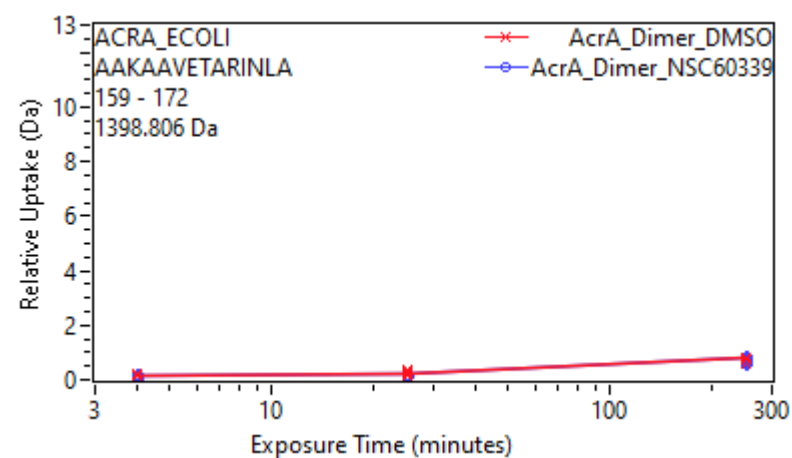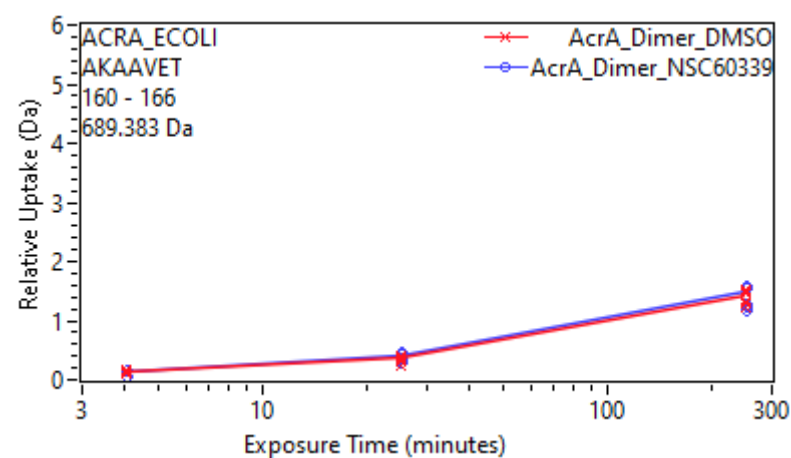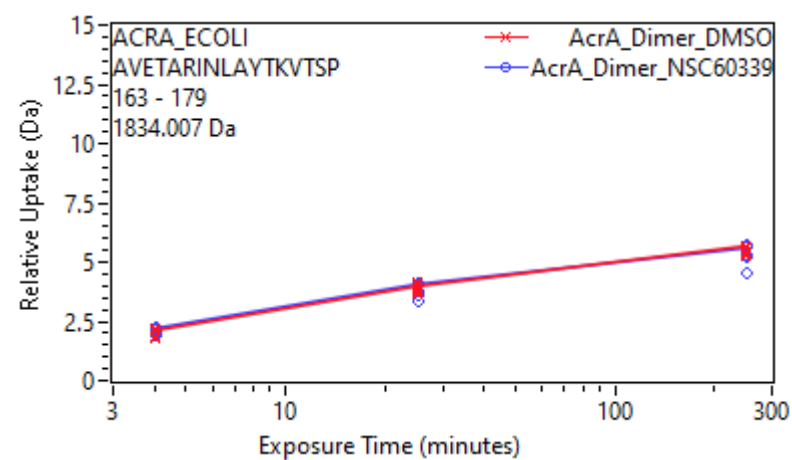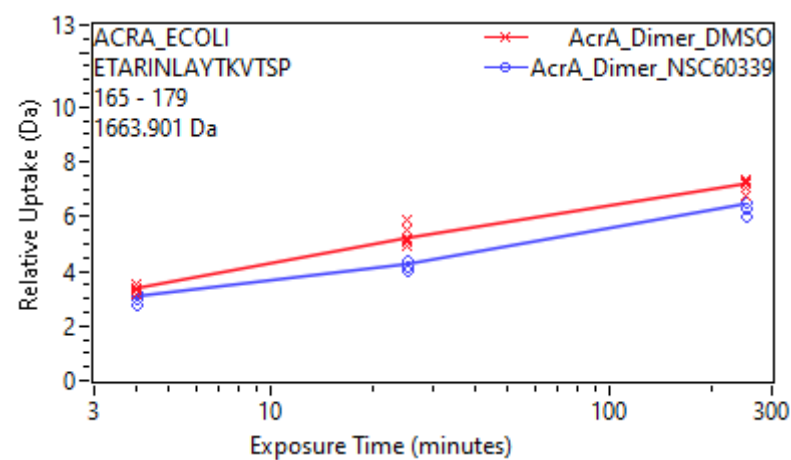

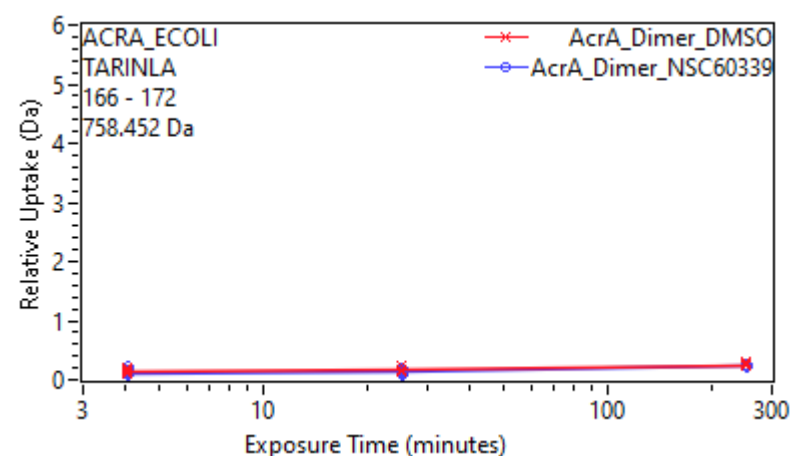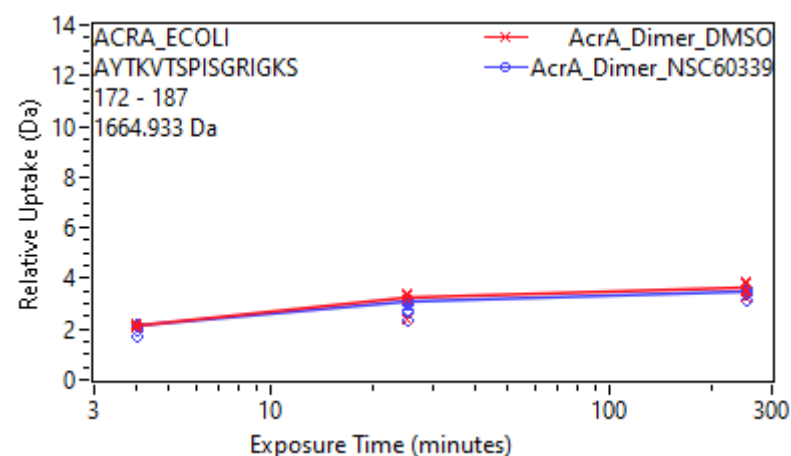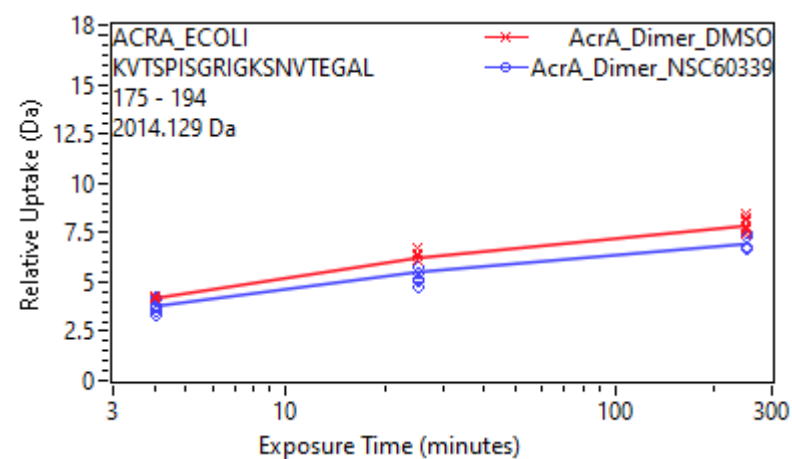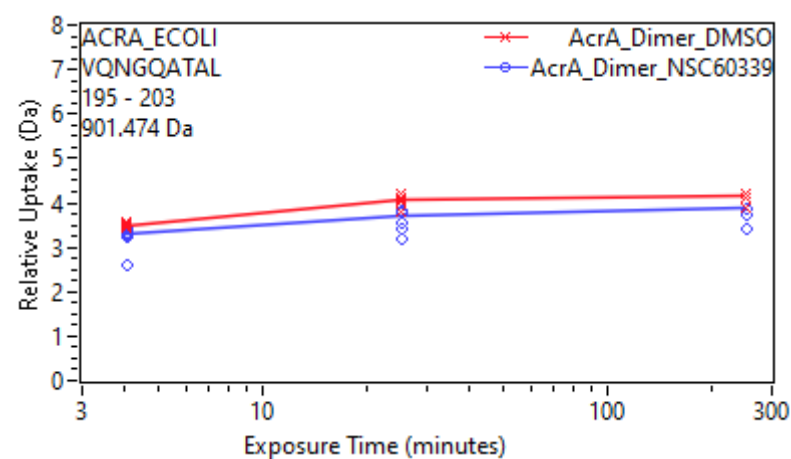

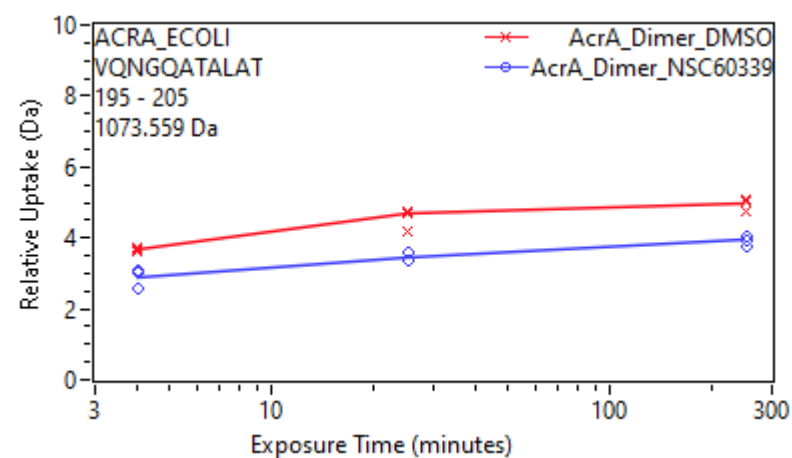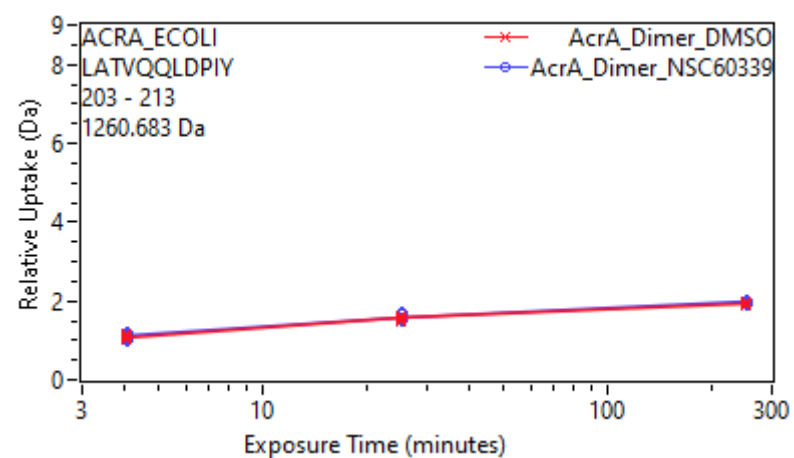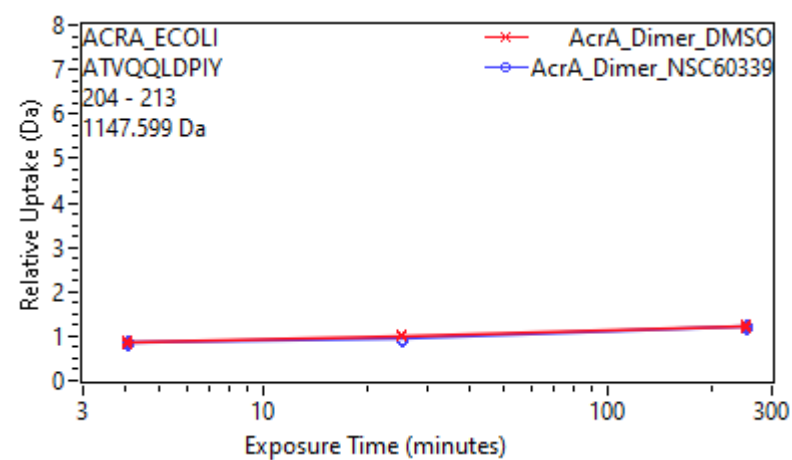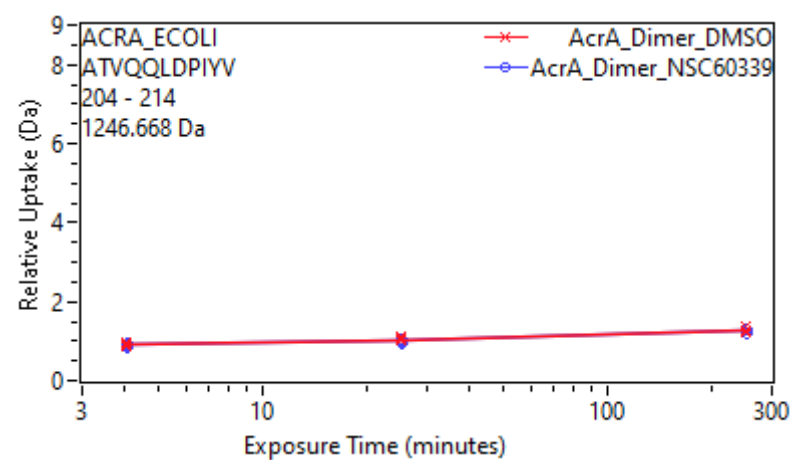

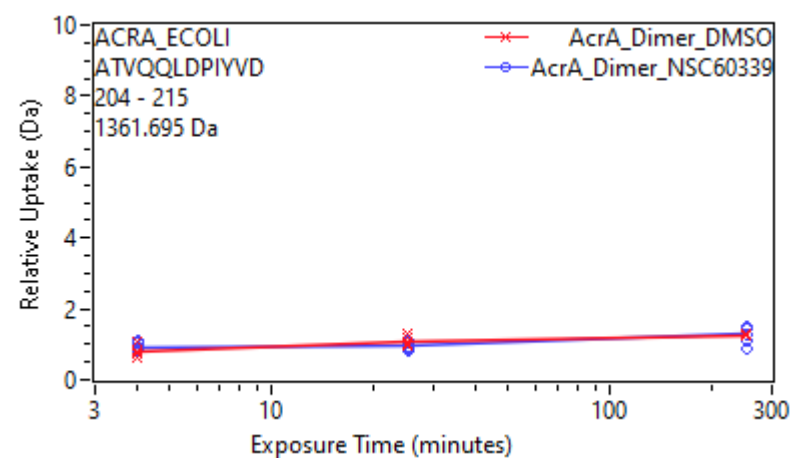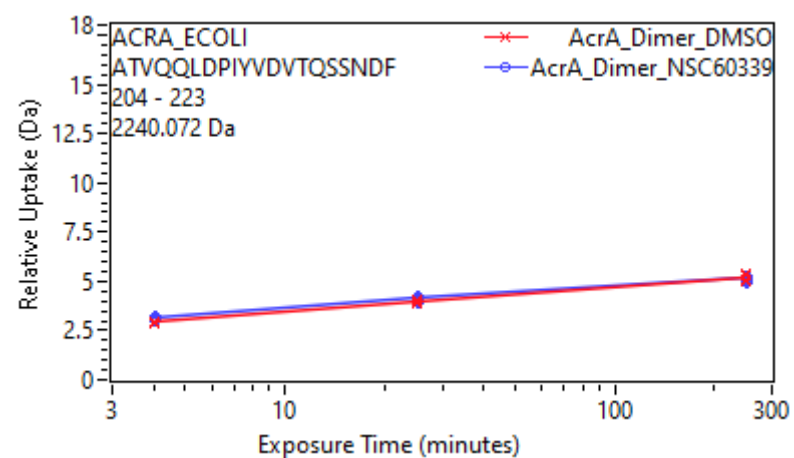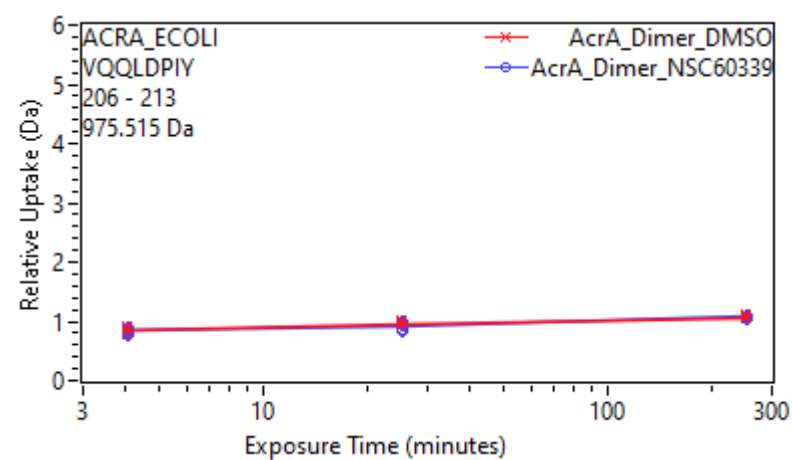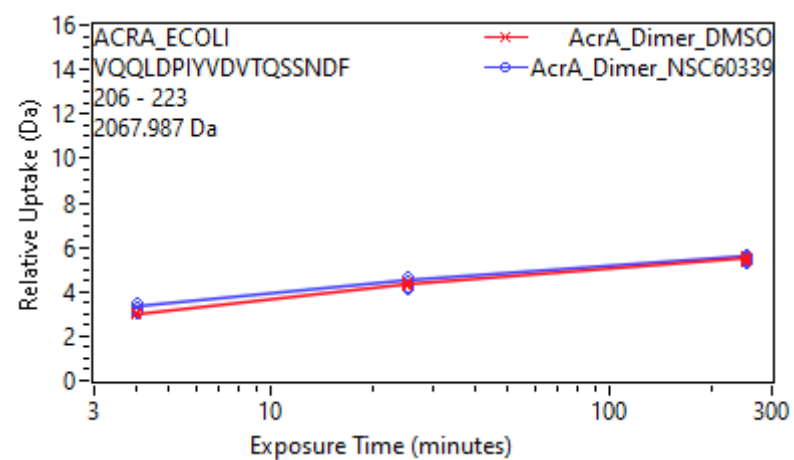

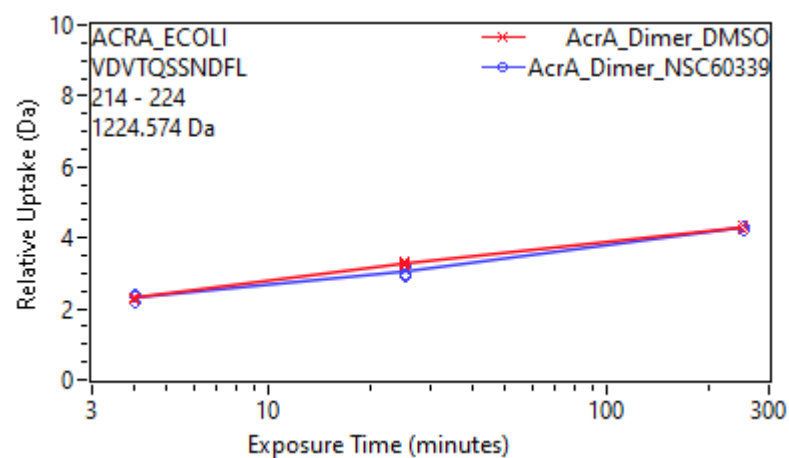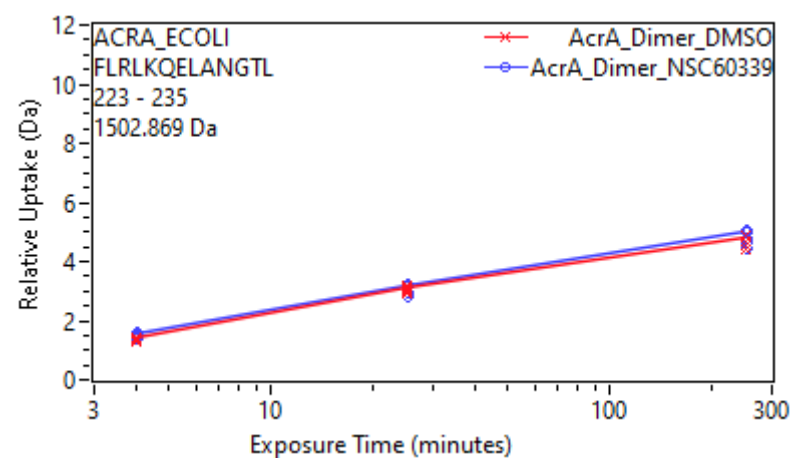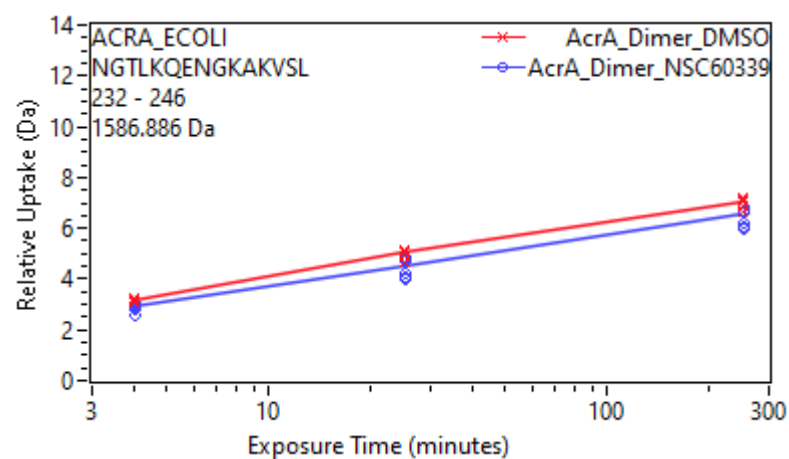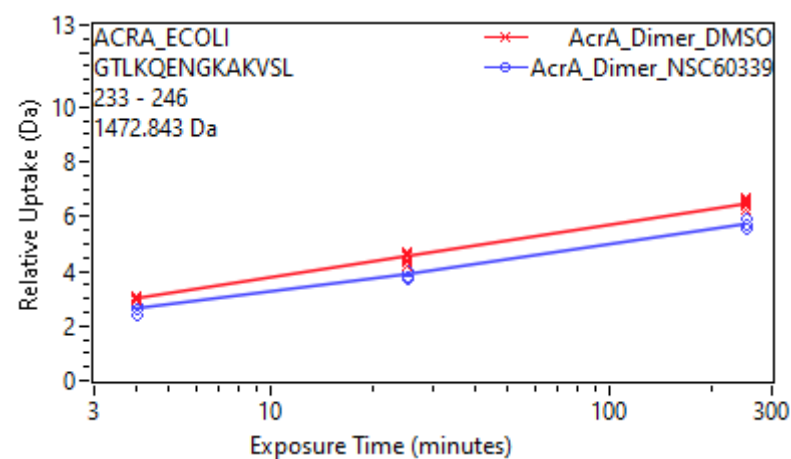

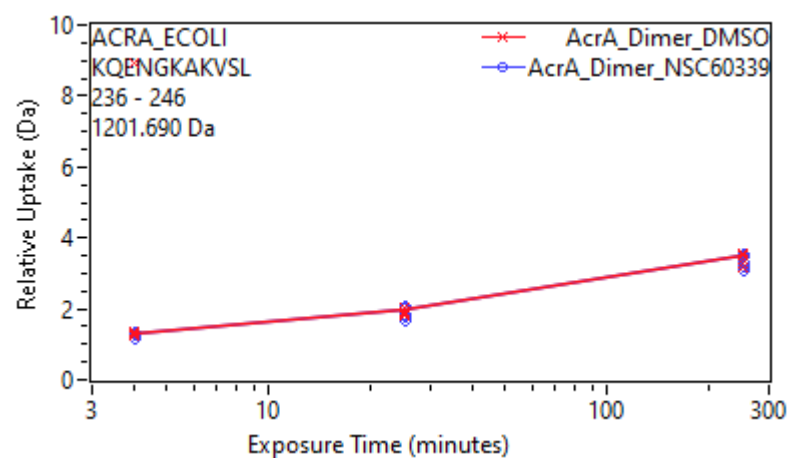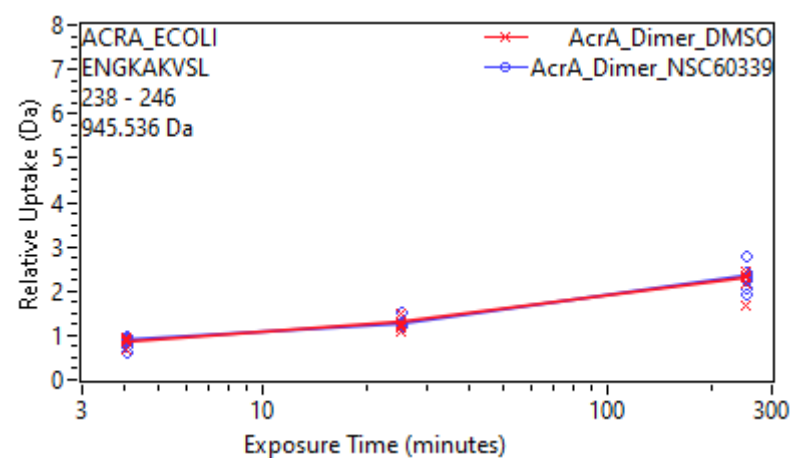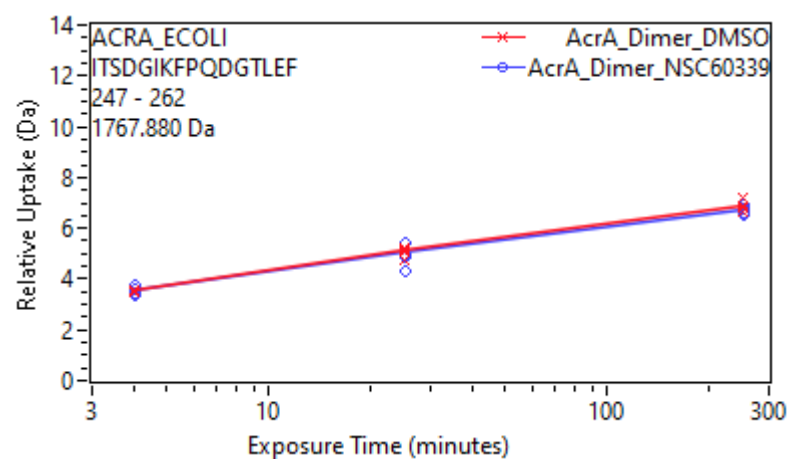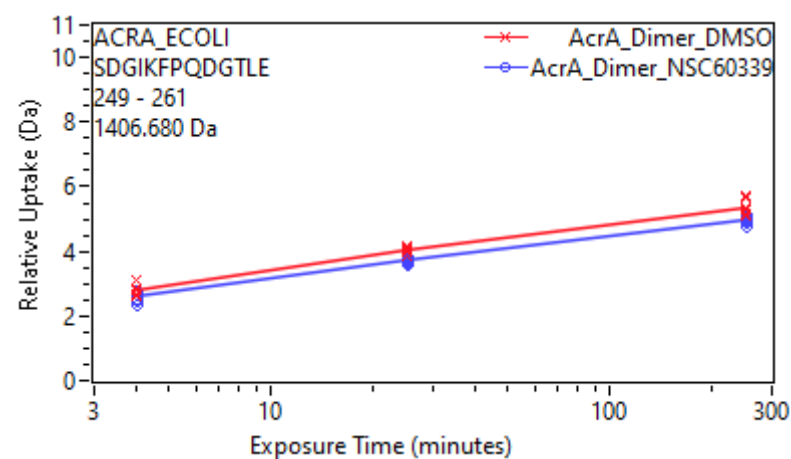

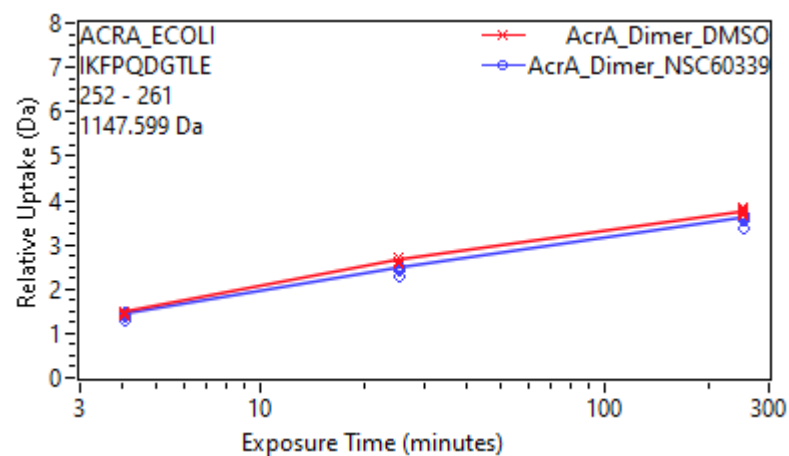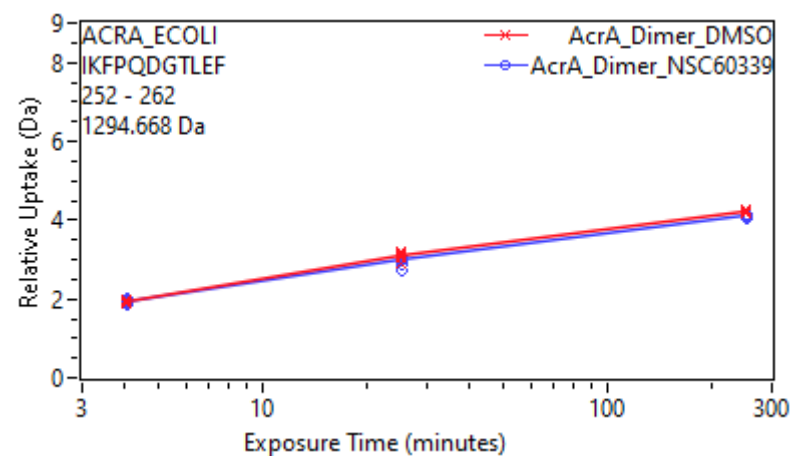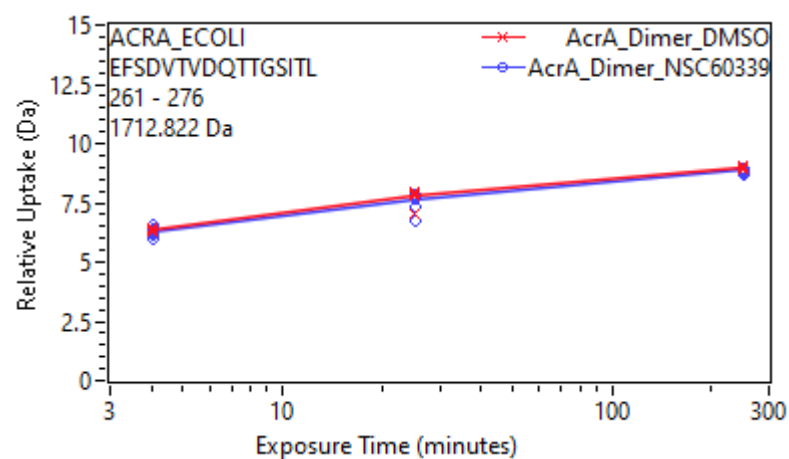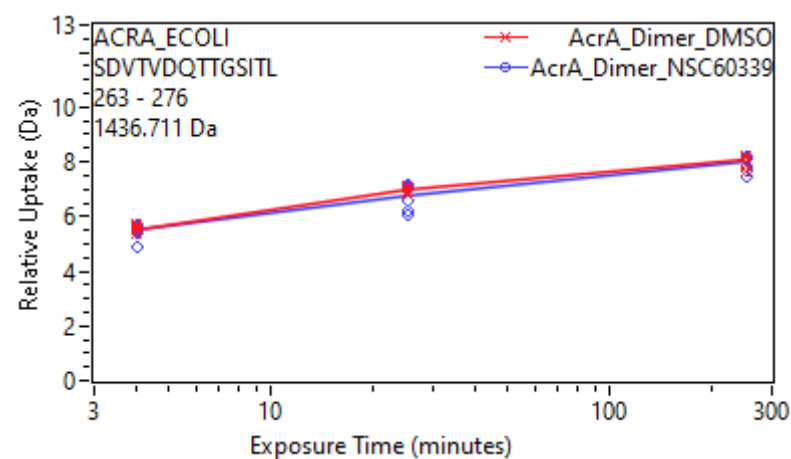

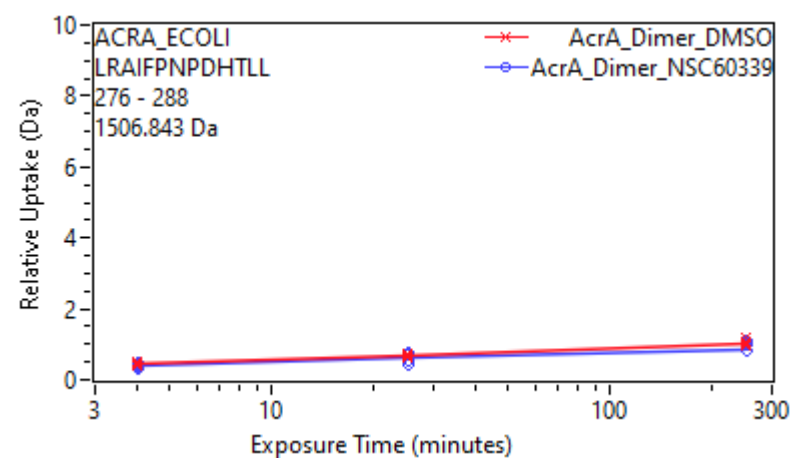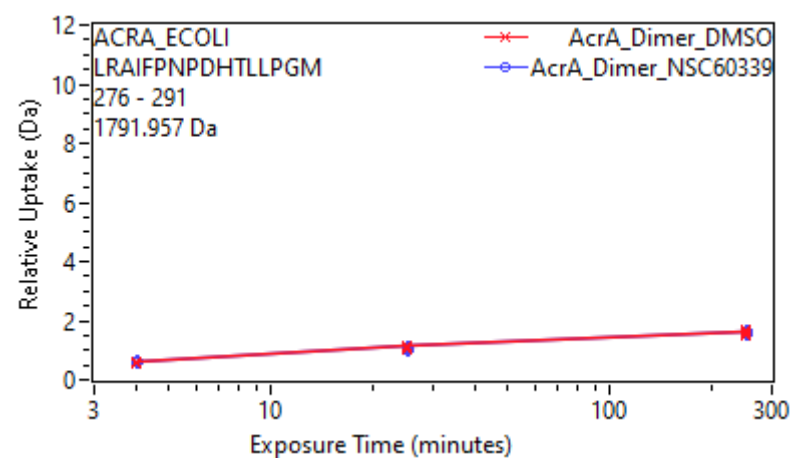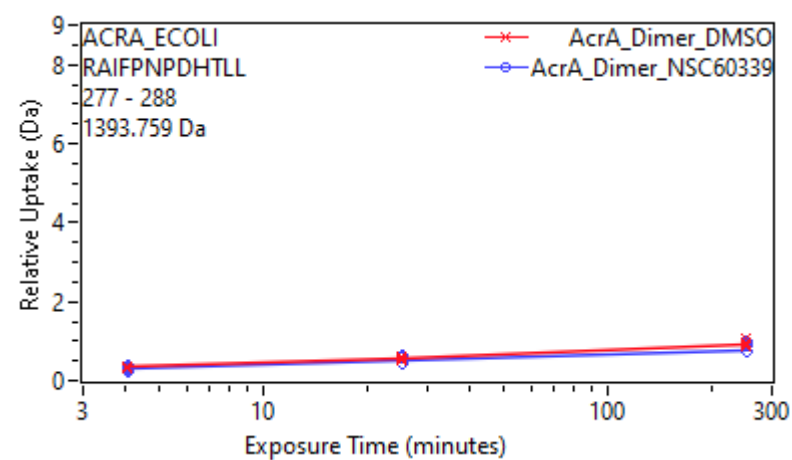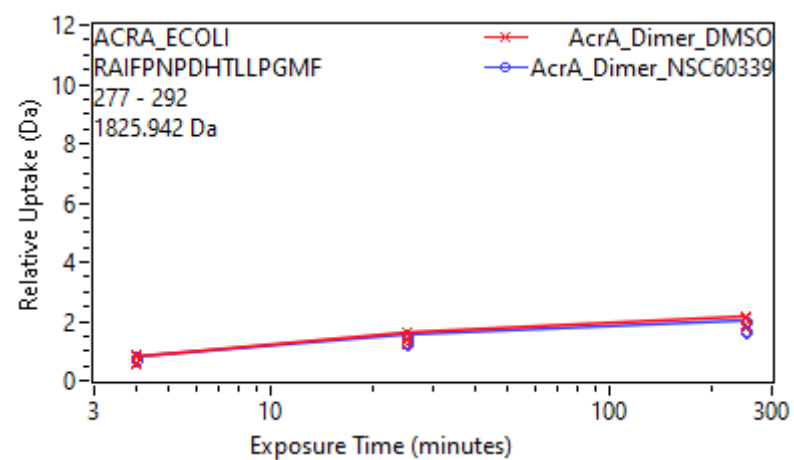

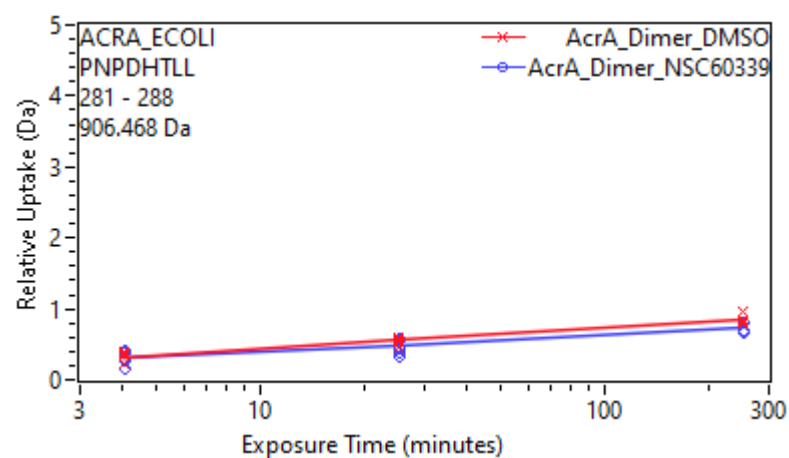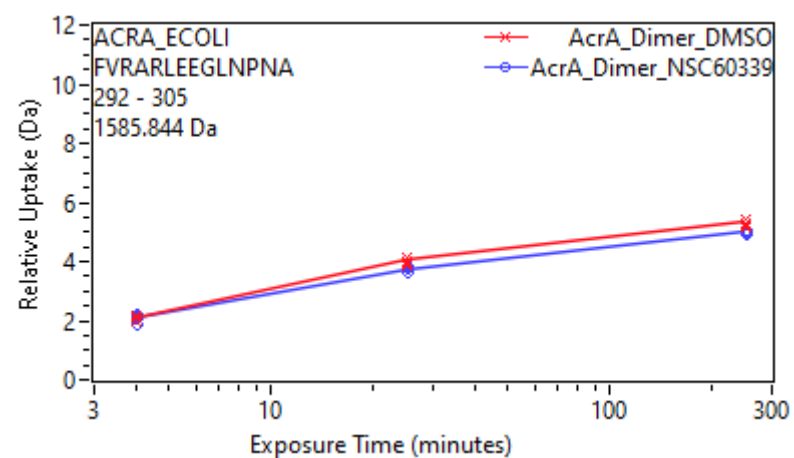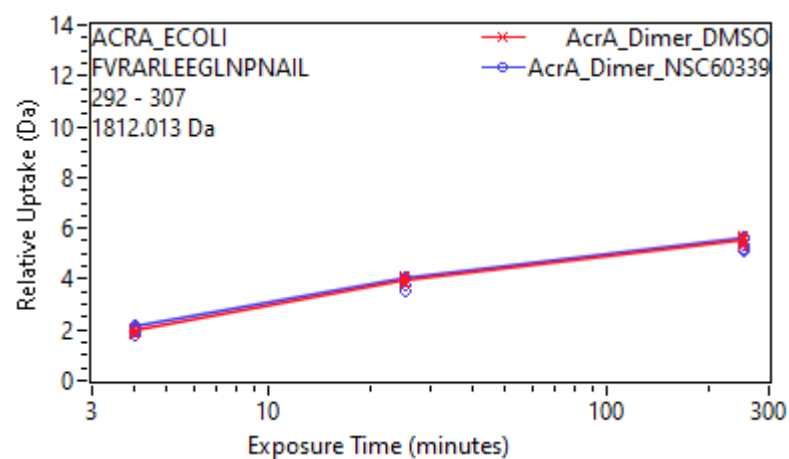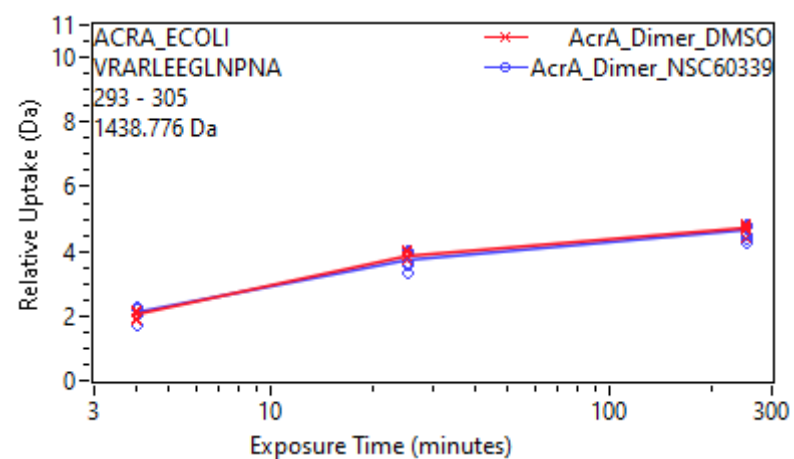

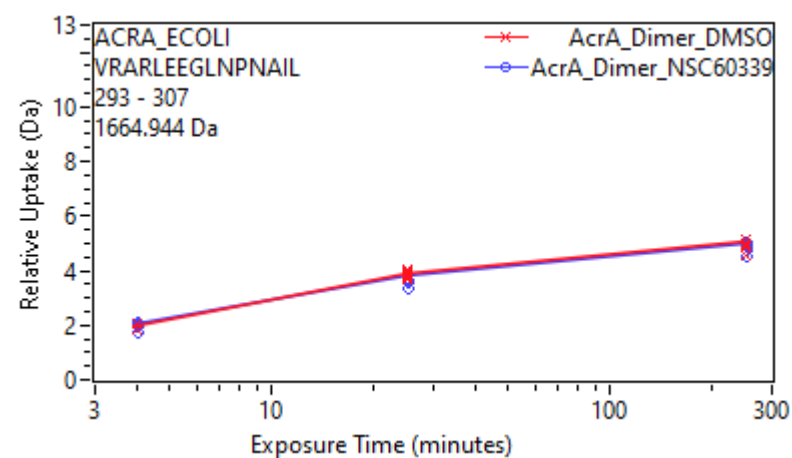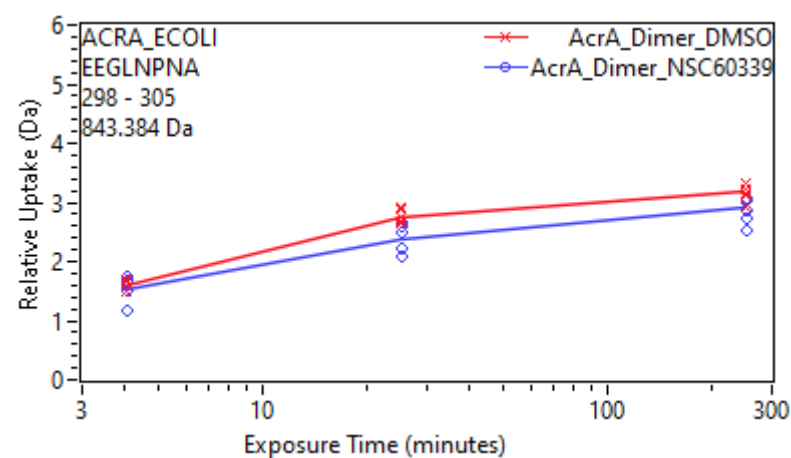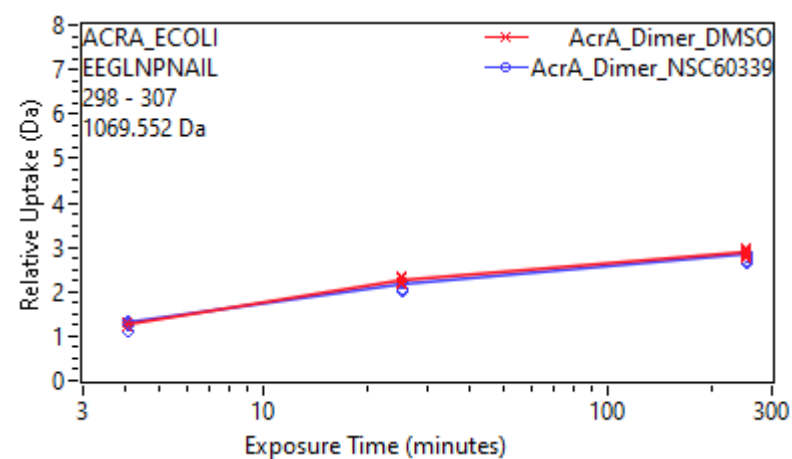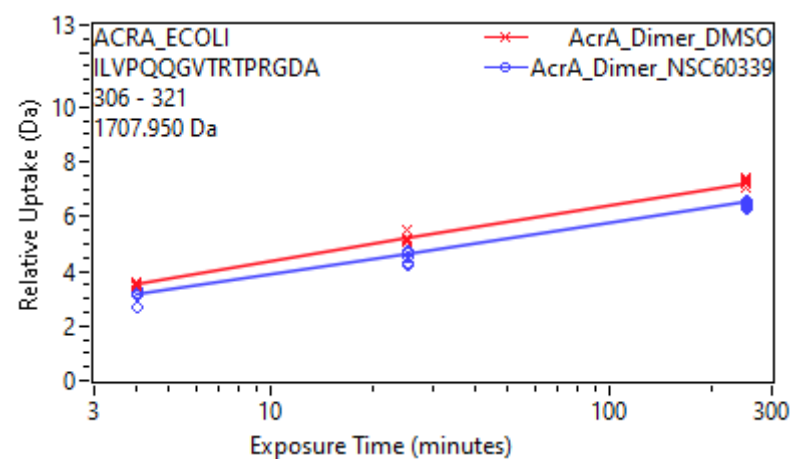

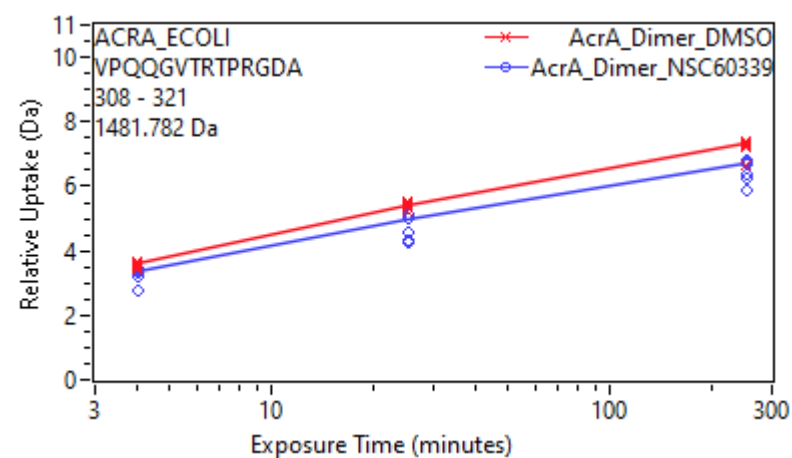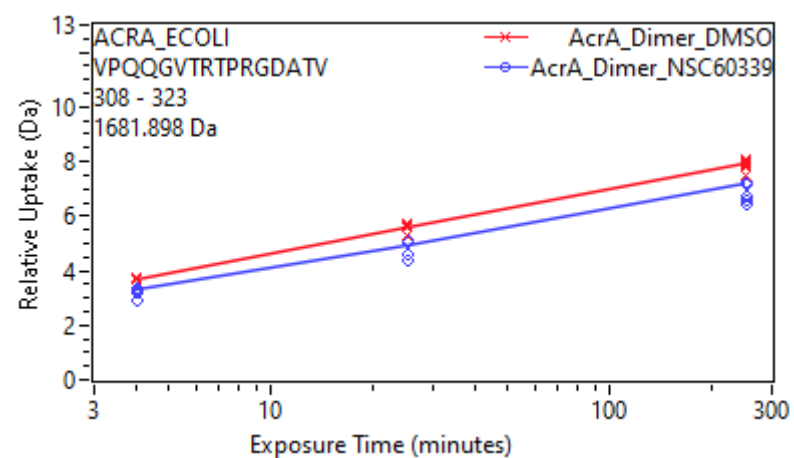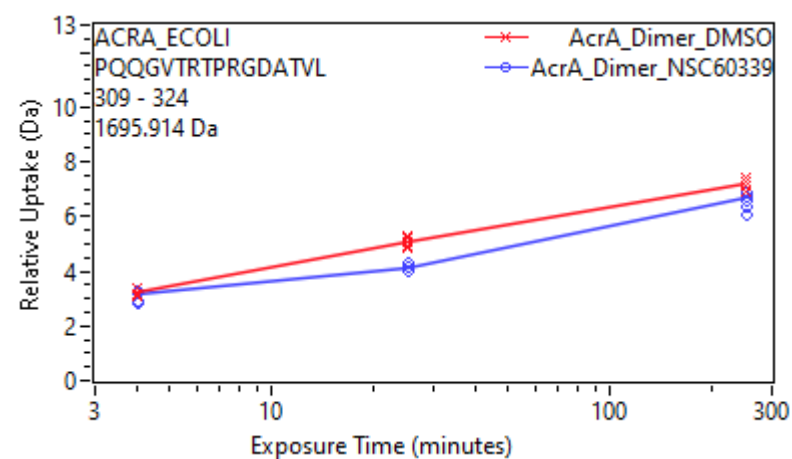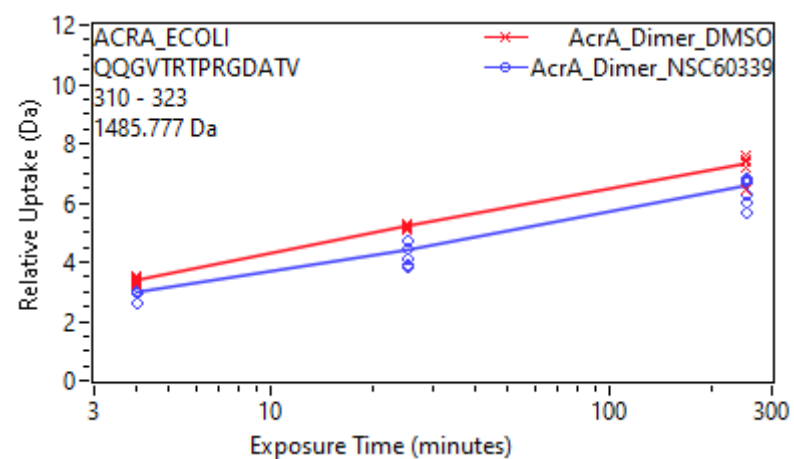

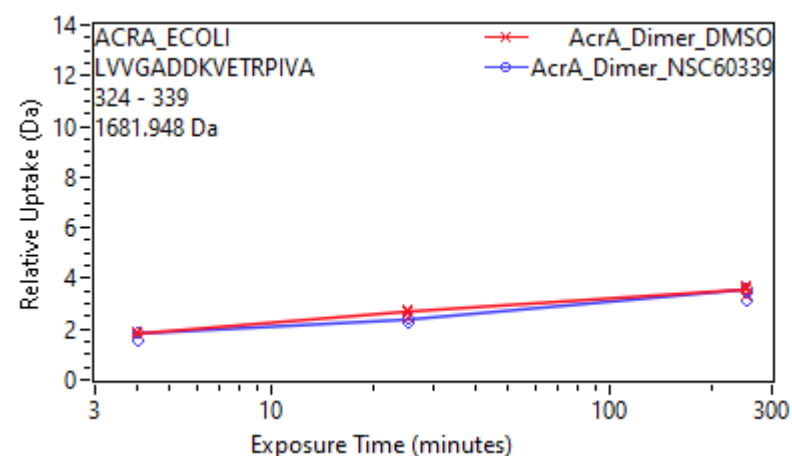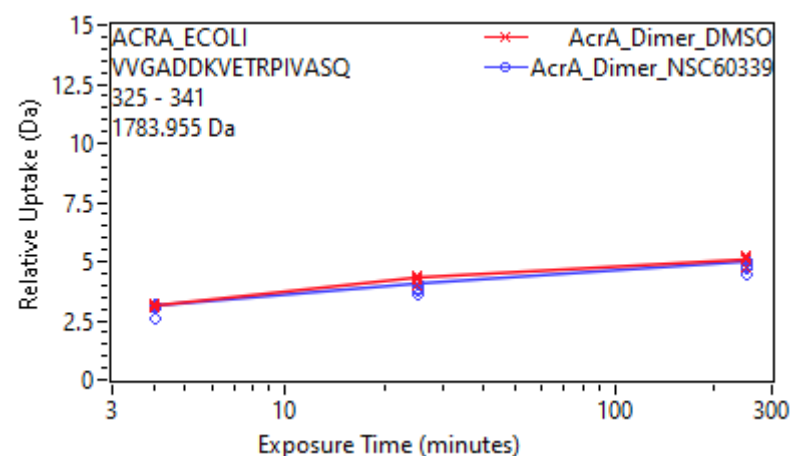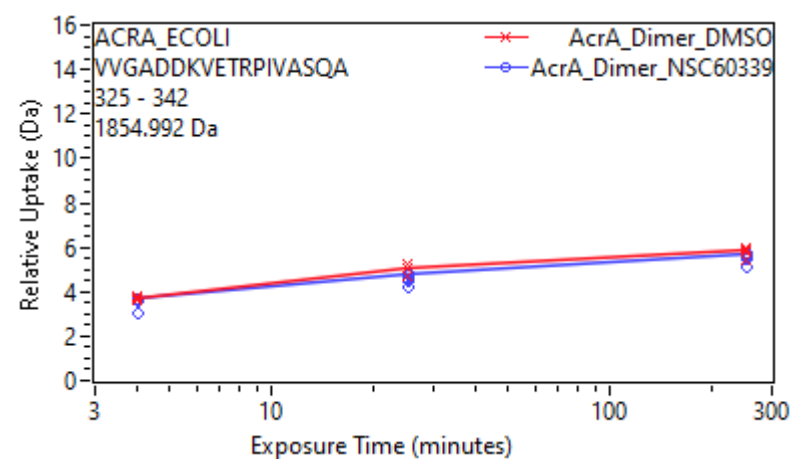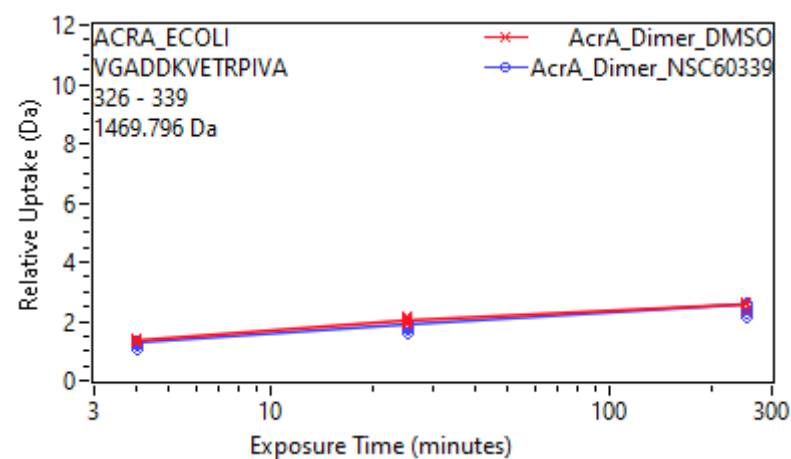

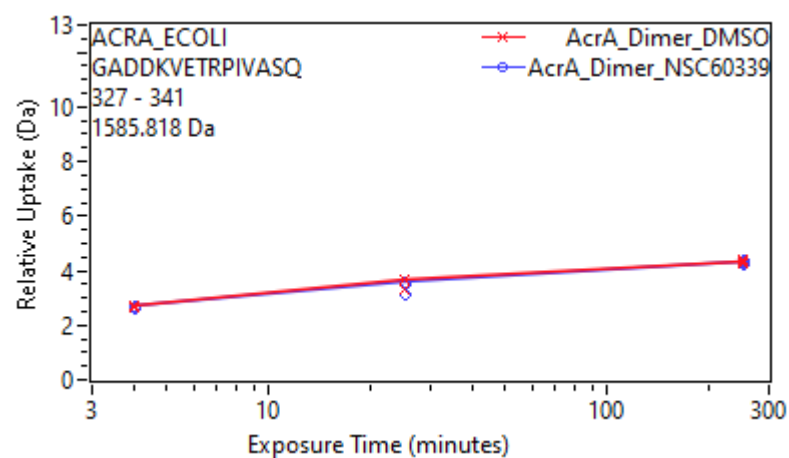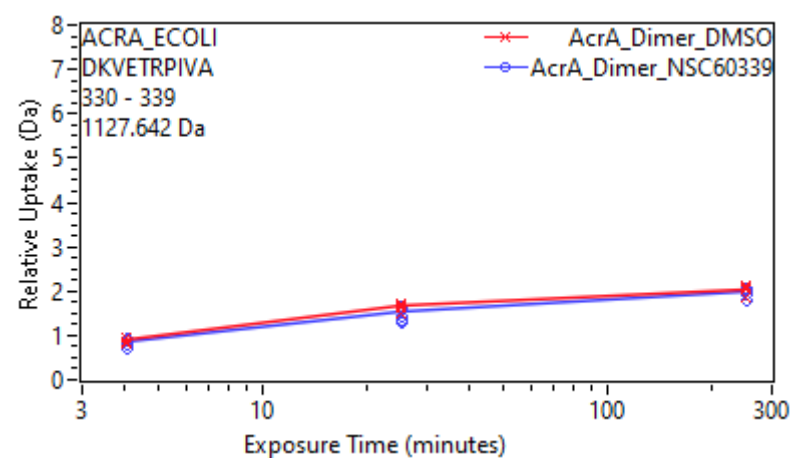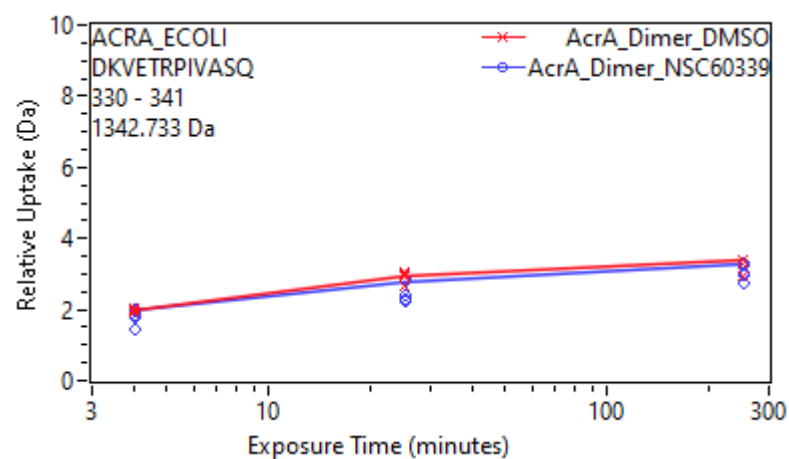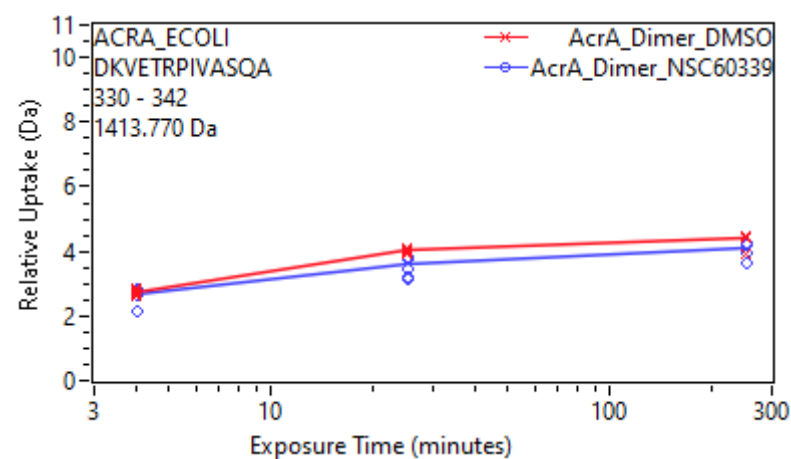

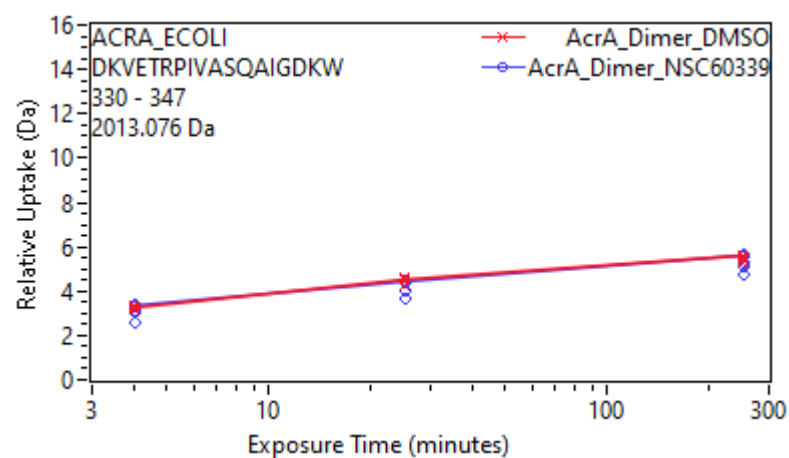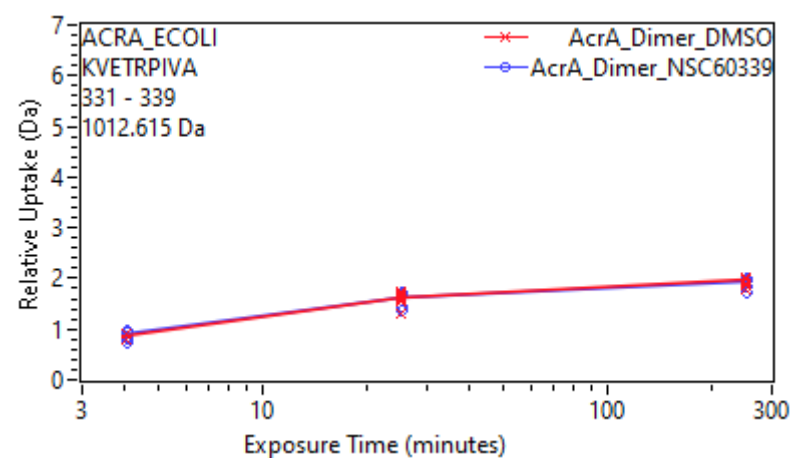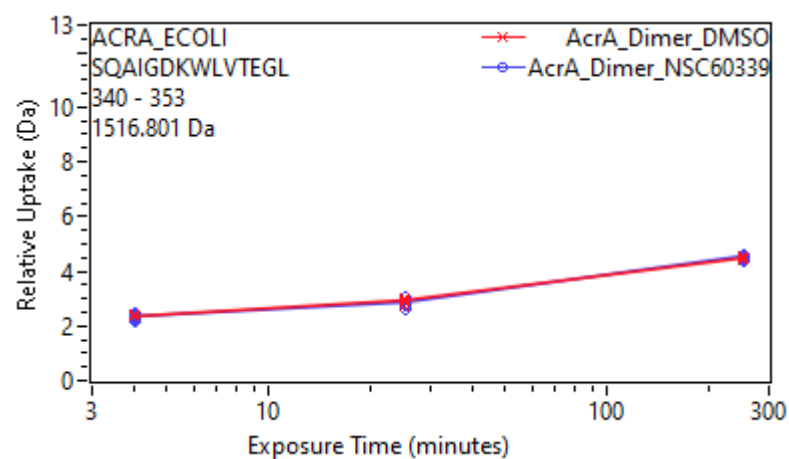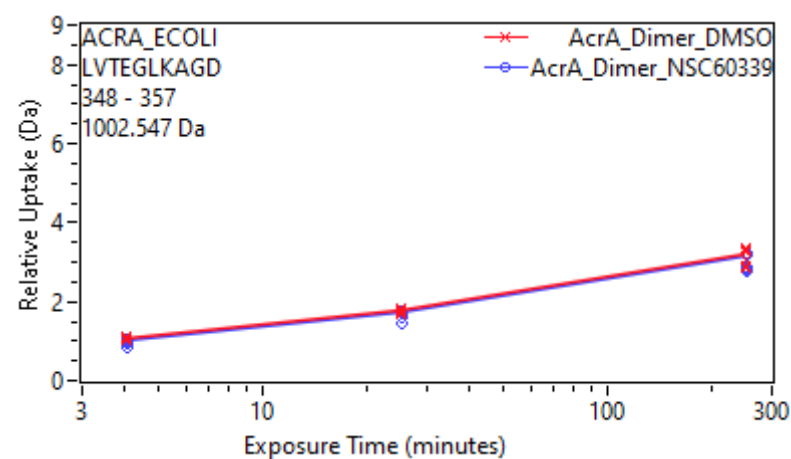

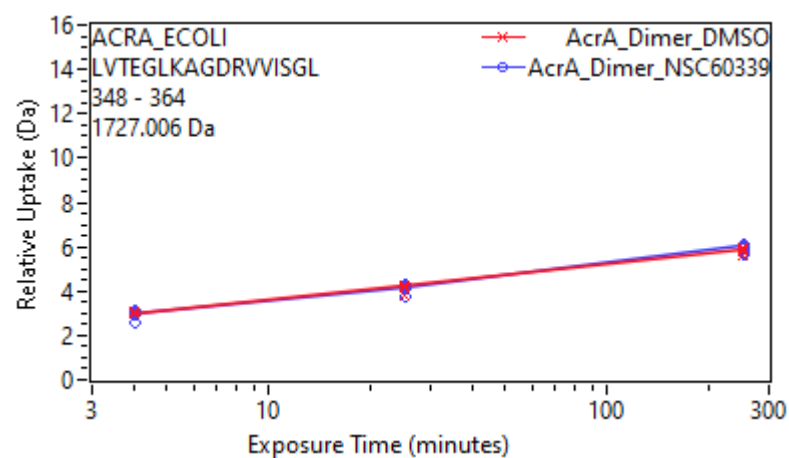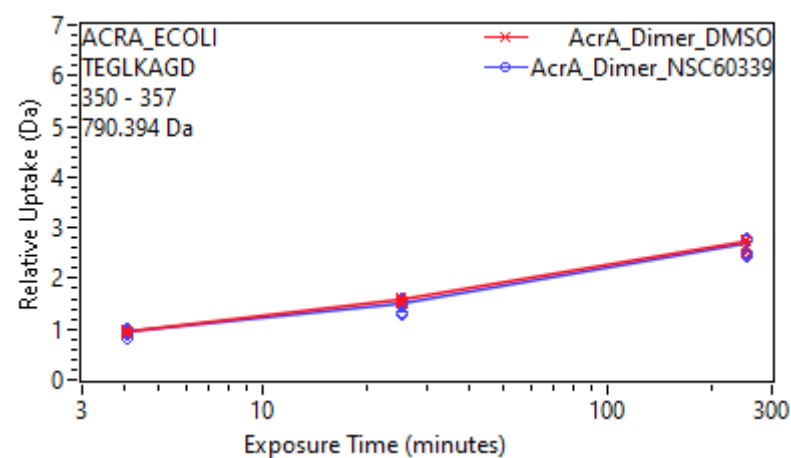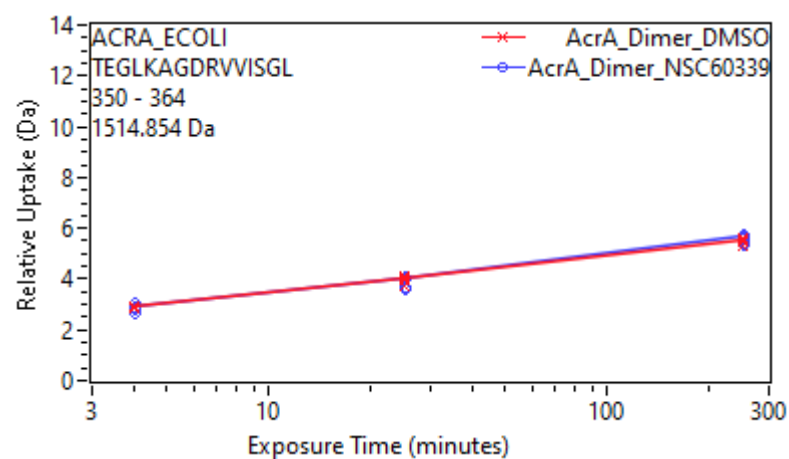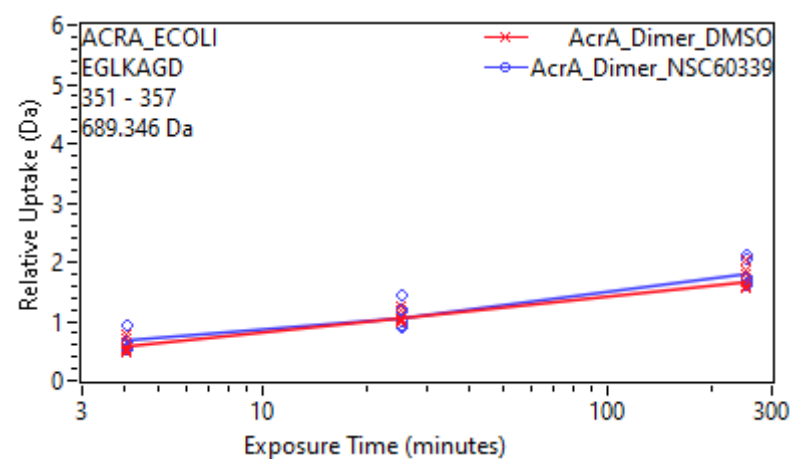

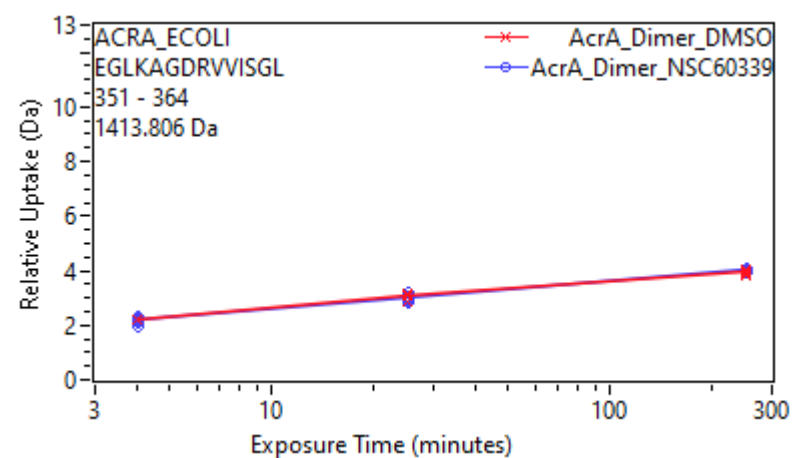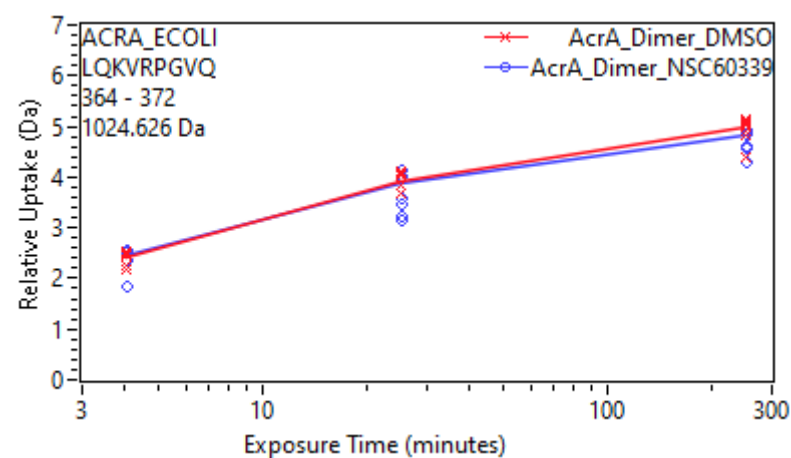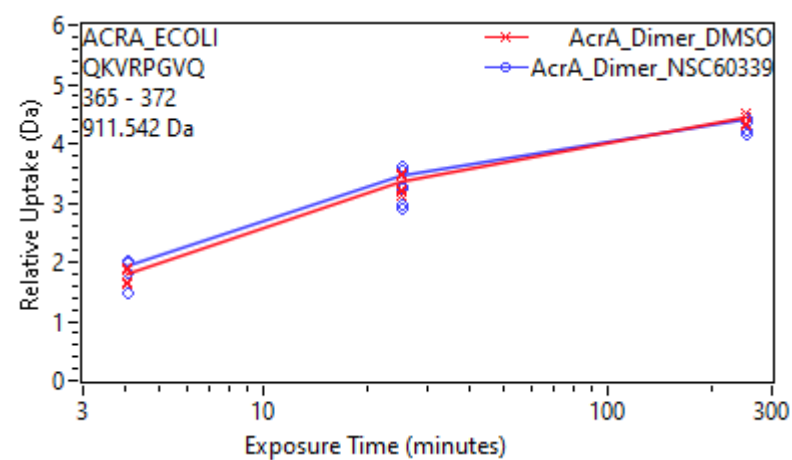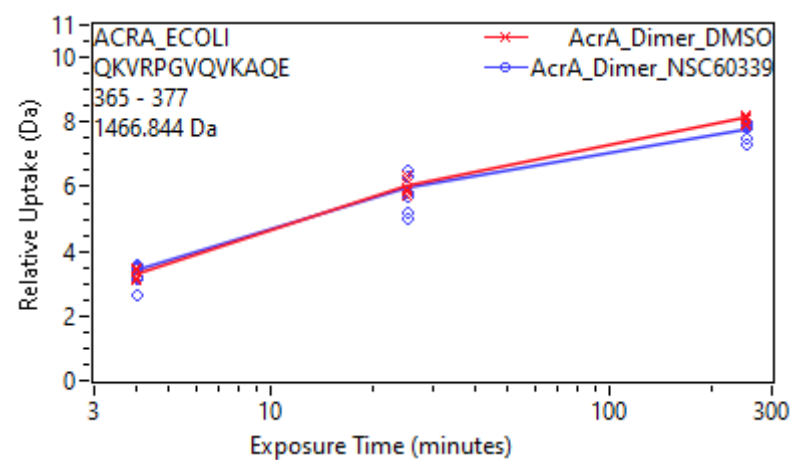

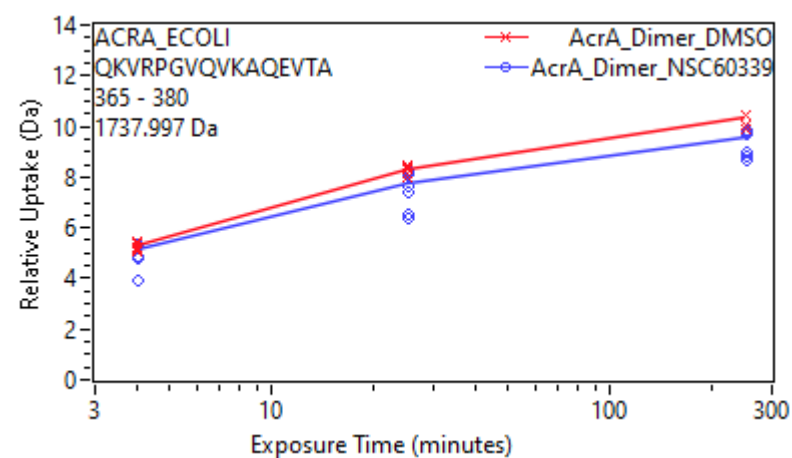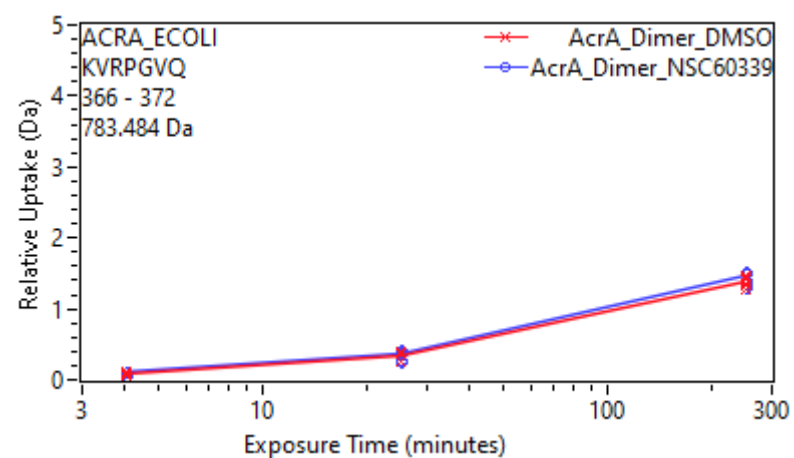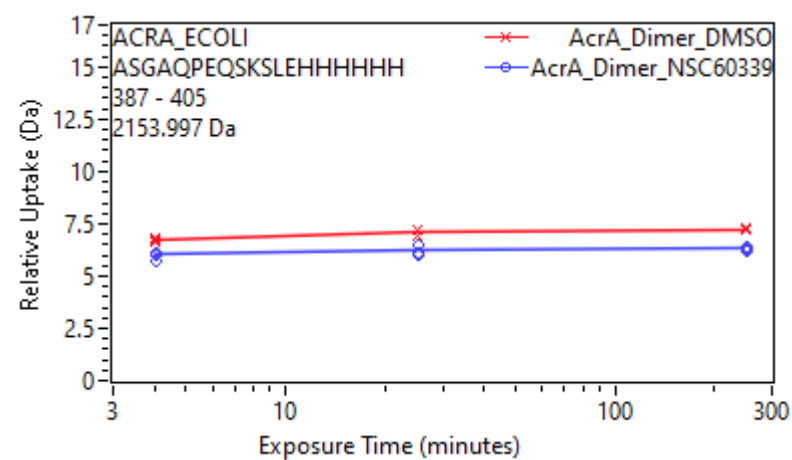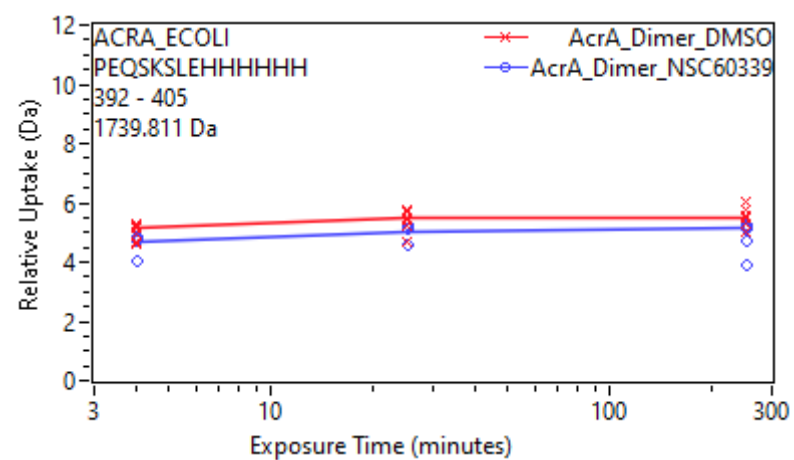

Supplement: Supplementary file 6 — Supplementary Data 3 [file 41467_2023_39615_MOESM6_ESM.zip › Supplementary Data 3/AcrAsd_NSC60339_UptakePlots.pdf]
