# Supplementary material for: Conformational restriction shapes the inhibition of a multidrug efflux adaptor protein
Source: Nat Commun. 2023 Jul 18;14:3900. doi: 10.1038/s41467-023-39615-x (PMC10354078; doi:10.1038/s41467-023-39615-x)
Supplement: Supplementary file 7 — Reporting Summary [file 41467_2023_39615_MOESM7_ESM.pdf]

nature portfolio

Corresponding author(s): Eamonn Reading, Helen Zgurskaya, JC Gumbart

Last updated by author(s): May 31, 2023

Reporting Summary

Nature Portfolio wishes to improve the reproducibility of the work that we publish. This form provides structure for consistency and transparency in reporting. For further information on Nature Portfolio policies, see our Editorial Policies and the Editorial Policy Checklist.

Statistics

For all statistical analyses, confirm that the following items are present in the figure legend, table legend, main text, or Methods section.

n/a	Confirmed	
☐	☒	The exact sample size (*n*) for each experimental group/condition, given as a discrete number and unit of measurement
☐	☒	A statement on whether measurements were taken from distinct samples or whether the same sample was measured repeatedly
☐	☒	The statistical test(s) used AND whether they are one- or two-sided<br>*Only common tests should be described solely by name; describe more complex techniques in the Methods section.*
☒	☐	A description of all covariates tested
☐	☒	A description of any assumptions or corrections, such as tests of normality and adjustment for multiple comparisons
☐	☒	A full description of the statistical parameters including central tendency (e.g. means) or other basic estimates (e.g. regression coefficient) AND variation (e.g. standard deviation) or associated estimates of uncertainty (e.g. confidence intervals)
☐	☒	For null hypothesis testing, the test statistic (e.g. *F*, *t*, *r*) with confidence intervals, effect sizes, degrees of freedom and *P* value noted<br>*Give P values as exact values whenever suitable.*
☒	☐	For Bayesian analysis, information on the choice of priors and Markov chain Monte Carlo settings
☒	☐	For hierarchical and complex designs, identification of the appropriate level for tests and full reporting of outcomes
☒	☐	Estimates of effect sizes (e.g. Cohen's *d*, Pearson's *r*), indicating how they were calculated

Our web collection on statistics for biologists contains articles on many of the points above.

Software and code

Policy information about availability of computer code

Data collection	MassLynx (v. 4.1, Waters) was used for controlling the Xevo G2-XS Q-Tof mass spectrometer and the Synapt G2-Si mass spectrometer for the acquisition of all MS data
Data analysis	ProteinLynX Global Server (v. 3.0.2, Waters) was used for processing MSE data to obtain a peptide list. DynamX (v 3.0, Waters) was used to further filter the peptides and analyze the HDX results of the peptides. HDeXplosion (v.1.2) was used for statistical analysis of HDX data. Chimera UCSF (v.1.15) was used to visualise protein structures. PyHDX (v.0.4.0) was used for the visualsation of heatmaps. ChemDraw (v.21.0) was used to draw chemical structures. MassLynx (v. 4.1) and UniDec (v.4.2.2) were used to analyze Native-MS data. MD simulations: Simulations were run using either NAMD2.1464 or NAMD365 depending on the computational resource used, and the CHARMM36m force field. Force-field parameters for NSC-60339 were generated using the CGenFF webserver67 and are provided as Supplementary Data. MD data analysis. All system preparation and analysis was carried out using Visual Molecular Dynamics (VMD).

For manuscripts utilizing custom algorithms or software that are central to the research but not yet described in published literature, software must be made available to editors and reviewers. We strongly encourage code deposition in a community repository (e.g. GitHub). See the Nature Portfolio guidelines for submitting code & software for further information.

Data

Policy information about availability of data

All manuscripts must include a data availability statement. This statement should provide the following information, where applicable:

- Accession codes, unique identifiers, or web links for publicly available datasets
- A description of any restrictions on data availability
- For clinical datasets or third party data, please ensure that the statement adheres to our policy

HDX-MS and meta-data supporting the findings are published in the Source Data File and the Supporting Data Tables respectively, in line with the suggestions made by Masson, G et al., Nature Methods, 16, 595-602, (2019). HDX-MS proteomics data files have been deposited to the ProteomeXchange Consortium via the PRIDE partner repository with the project accession code: PXD041359. The MD simulations trajectories can be found in Supplementary Data 3. The protein structures from other publications referenced in this paper are accessible under the PDB accession codes 5O66 and 2F1M. The AlphaFold2 structure was generated from the P0AE06 UniProt accession code. The source data underlying Figures. 2a-d, 4a,b, 5, and Supplementary Figures. 3, 7, 9, 10, 11, 14, 15.

Human research participants

Policy information about studies involving human research participants and Sex and Gender in Research.

Reporting on sex and gender	This information has not been collected
Population characteristics	See above
Recruitment	See above
Ethics oversight	See above

Note that full information on the approval of the study protocol must also be provided in the manuscript.

Field-specific reporting

Please select the one below that is the best fit for your research. If you are not sure, read the appropriate sections before making your selection.

[x] Life sciences [ ] Behavioural & social sciences [ ] Ecological, evolutionary & environmental sciences

For a reference copy of the document with all sections, see nature.com/documents/nr-reporting-summary-flat.pdf

Life sciences study design

All studies must disclose on these points even when the disclosure is negative.

Sample size	Samples were measured in singlets with single measuring points measured at least in triplicate/quadruplet. No sample size calculations required, number of samples based upon the field recommendations (Masson, G et al., Nature Methods, 16, 595-602, (2019)).
Data exclusions	Peptides excluded if they were insufficiently fragmented and/or if the mass error was above 10 ppm.
Replication	All deuterium timepoint measurements in HDX-MS experiments were performed in at least triplicate/quadruplet, with some biological repeats. Individual datasets all collected on the same day to minimize instrumental variations, and differential data collected in adjourning days. All replication attempts successful. All other experiments performed in technical triplicates or quadruplets. All attempts at replication successful. Source data found in the Source Data file.
Randomization	Samples injected into the LC-MS system according to the measuring time point, so each timepoint was analyzed to completion before the next one started. The experiments were not planned in order and time points were analysed in series. A clean blank was performed between each LC-MS measurement to mitigate and assess for any carryover between experiments.
Blinding	Blinding not relevant for this study as no live subjects were involved and the sample preparations performed in an experimental laboratory.

Reporting for specific materials, systems and methods

We require information from authors about some types of materials, experimental systems and methods used in many studies. Here, indicate whether each material, system or method listed is relevant to your study. If you are not sure if a list item applies to your research, read the appropriate section before selecting a response.

Materials & experimental systems

n/a	Involved in the study
☒	☐ Antibodies
☒	☐ Eukaryotic cell lines
☒	☐ Palaeontology and archaeology
☒	☐ Animals and other organisms
☒	☐ Clinical data
☒	☐ Dual use research of concern

Methods

n/a	Involved in the study
☒	☐ ChIP-seq
☒	☐ Flow cytometry
☒	☐ MRI-based neuroimaging
